# De novo transcriptome analysis of Tibetan medicinal plant Dysphania schraderiana

**DOI:** 10.1590/1678-4685-GMB-2018-0033

**Published:** 2019-06-13

**Authors:** Suhong Fu, Ming Lei, Yongqun Zhang, Zhaomin Deng, Jing Shi, Doudou Hao

**Affiliations:** 1 Molecular Medical Laboratory, Hospital of Chengdu Office of People’s Government of Tibetan Autonomous Region, Chengdu, China; 2 School of Science, Tibet University, Lhasa, China

**Keywords:** Dysphania schraderiana, *de novo* assembly, transcriptome, annotation, terpenoid biosynthesis

## Abstract

*Dysphania schraderiana* is widely distributed in Lhasa (Tibet, China) and used as a traditional medicine. However, the lack of genetic information hinders the understanding of its physiological processes, such as the biosynthesis of secondary metabolites. Herein, we used Illumina Hiseq4000 platform to sequence the transcriptome of flower and leaf tissues from *D. schraderiana* for the first time. Totally, 40,142 unigenes were assembled from approximately 5.2 million clean reads. All unigenes underwent gene prediction and were subsequently annotated in a NR (NCBI non-redundant protein) database, COG (Clusters of Orthologous Groups of proteins) database, and KEGG (Kyoto Encyclopedia of Genes and Genomes) database. Among the 40,142 unigenes, 2,579 genes were identified as differentially expressed between flowers and leaves, and used in further enrichment analysis. Also, 2,156 unigenes were annotated as transcription factors. Furthermore, our transcriptome analysis resulted in the identification of candidate unigenes annotated to enzymes involved in terpenoid biosynthesis. Taken together, this work has laid the foundation for the investigation of secondary metabolite biosynthesis and other physiological processes of *D. schraderiana*.

## Introduction

*Dysphania schraderiana*, in the Chenopodiaceae family, is widely distributed in the Qinghai-Tibet Plateau of China, Europe, and Africa. The Qinghai-Tibet Plateau is a low oxygen, low temperature, strong ultraviolet radiation, and poor soil environment. The adaptation of *D. schraderiana* to these extreme environments can be a good model for understanding evolutionary ecology. Besides, *D. schraderiana* was used as an indicator of fixed sand because of its high adaption to environmental adversities (Liu, 2013). Apart from its ecological value, the plant is used in a variety of applications such as medicine and insect control. In traditional Chinese medicine, *D. schraderiana* is used to, amongst others, mitigate wheeze, inflammation, spasm, migraine (Xie, 1996). Some recent studies indicated that the essential oil of *D. schraderiana* could be applied to prevent plant mite (Liu, 2015). In addition, the essential oil of *D. schraderiana* also has *in vitro* antibacterial activity against *Escherichia coli*, as well as anti-insect activity against red flour beetle and corn weevil (Lei *et al.*, 2015a,b; Shi, 2015). These findings suggest that *D. schraderiana* can became a promising antibacterial agent considering the increasing episodes of drug resistant bacteria. Moreover, the essential oil can also be utilized in foods, cosmetics, and cigarettes (Han *et al.*, 2013). Despite the importance of *D. schraderiana* and some genomic research on Chenopodiaceae plants such as *Beta vulgaris* ssp. *vulgaris*, and *Chenopodium quinoa* (Chou *et al.*, 2017; Zhang *et al.*, 2017), no genetic information exists for this non-model genus. Therefore, it is necessary to explore genetic data sources of *D. schraderiana* for gene discovery and further functional studies.

In essential oils of *D. schraderiana*, 52 phytochemical compounds were identified by GC-MS (Finnigan Voyager) analysis and the dominant components are sesquiterpenes and oxygen-containing derivatives, followed by monoterpenes (Shi, 2015). The synthesis of terpenoids relies on two key pathways, the 2-C-methyl-derythritol-4-phosphate (MEP) and mevalonate (MVA) pathways, involving diverse enzymes (Goodwin, 1979; Rohdich *et al.*, 2001; Cheng *et al.*, 2007). Isopentenyl pyrophosphate (IPP) and dimethylallyl pyrophosphate (DMAPP), the universal precursors for terpenoids, are synthesized through the MEP and MVA pathways (Rohdich *et al.*, 2001). GPP synthase catalyzes IPP and DMAPP to form geranyl diphosphate (GPP), while FPP synthase converts GPP into farnesyl diphosphate (FPP) (Rohdich *et al.*, 2001). GPP and FPP are catalyzed to form monoterpenes and sesquiterpenes by monoterpene synthase (mono-TPS) and sesquiterpene synthase (sesqui-TPS), respectively (Gershenzon, 2009). The discovery and research of key enzymes related to terpenoid biosynthesis in *D. schraderiana* could help understand the composition of the essential oil.

Nowadays, *de novo* assembly is gaining more attention and has proved to be a rapid and cost-effective method for short reads in non-model organisms (Crawford *et al.*, 2010). Millions of short tags could be generated from RNA-Seq platforms and subsequently assembled, which can help to interpret genome and transcriptome sequences. Collectively, *D. schraderiana* could be a valuable source to identify and discover natural compounds with biological activity for further pharmaceutical research, and be used to study the adaptive genetic mechanism. In this study, we characterized the flower and leaf transcriptomes of *D. schraderiana* using Illumina sequencing platform, and annotated sequences from transcriptome in multiple databases.

## Materials and Methods

### Plant materials

Whole flowers and young leaves of *D. schraderiana* were collected from the new campus of Tibet University (N29°38’, E91°10’), Lhasa, Tibet. Plant materials were quickly frozen in liquid nitrogen upon harvest and stored at -80 °C until RNA extraction.

### RNA extraction and quality determination

Total RNA was isolated using Trizol (Invitrogen Inc, USA). RNA quantity and quality was checked using a Nanodrop2000 spectrophotometer. Agarose gels (1%) were utilized to monitor RNA degradation and contamination. RNA integrity was assessed using an Agilent2100 system (Agilent Technologies, CA, USA) at OD 260/280 – 1.8 to 2.2, RIN ≥ 8, > 12 μg.

### cDNA library construction for Illumina sequencing

The TruseqTM RNA Sample Prep Kit (Illumina, USA) was used to prepare the RNA-seq transcriptome libraries. DNA-free mRNA was captured by magnetic Oligo (dT) beads (Invitrogen) and fragmented to a size of 200 bp with a fragmentation buffer. Using mRNA as a template, double-stranded cDNA was synthesized with a SuperScript double-stranded cDNA Synthesis Kit (Invitrogen) using random hexamer primers (Illumina). Then the end fragments were subjected to end-repair and `A’ base addition. PCR amplification was carried out for 15 PCR cycles. Then, cDNA target fragments were selected on 2% Certified Low Range Ultra Agarose (Bio-Rad, USA) and quantitated by TBS380 Picogreen (Invitrogen).

The clustering of the index-coded samples was performed on a cBot Cluster Generation System using the TruSeq PE Cluster Kit v3-cBot-HS (Illumina) according to the manufacturer’s instructions. After cluster generation, the library preparations were sequenced on an Illumina Hiseq4000 Truseq SBS Kit v3-HS (200 cycles).

### *De novo* assembly and sequence annotation

Raw reads were cleaned by removing: adapters that were added for reverse transcription and sequencing; reads containing N (unknown nucleotides) more than 10%; low quality reads with average phred scores less than 20; and sequences of less than 20 bp after trimming, which was performed with SeqPrep (https://github.com/jstjohn/SeqPres) and Sickle (https://github.com/najoshi/sickle). The Q20, Q30, GC content, and sequence duplication level of the clean data were calculated (Erlich *et al.*, 2008). All clean reads were used for *de novo* assembly into unigenes using Trinity (http://trinityrnaseq.sourceforge.net/) (Cock *et al.*, 2009; Grabherr *et al.*, 2011). Results of assembled unigenes were used to gather statistics from the basic indicators.

All the assembled unigenes of the two tissues were submitted to gene prediction using the ORF prediction procedure implemented in Trinity, then corrected against the Pfam database (http://pfam.sanger.ac.uk/). Finally, they were aligned using BLASTX (Version 2.2.25) (Camacho *et al.*, 2009) against the Non-redundant (Nr, ftp://ftp.ncbi.nih.gov/blast/db/FASTA/nr.gz) (E-value threshold of 1.00e-5) (Deng *et al.*, 2006), the String database, the Swissprot database, the Clusters of Orthologous Groups of proteins (Harris, 2004; COG, http://www.ncbi.nlm.nih.gov/COG/), and the Kyoto Encyclopedia of Genes and Genomes (KEGG, http://www.genome.jp/kegg/) (Kanehisa *et al.*, 2004) database to acquire the corresponding annotation information.

### Identification and functional characterization of differentially expressed genes

To investigate the expression profiles of unigenes from flower and leaf tissues of *D. schraderiana*, high-quality reads from each sample were mapped on the Trinity transcripts assembly using Bowtie (http://bowtie-bio.sourceforge.net/) (Langmead *et al.*, 2012). RESM (http://deweylab.biostat.wisc.edu/rsem/) was used to filter and count the mapped reads (Li *et al.*, 2011). Differential expression values were computed with edgeR (version 2.12) (Robinson *et al.*, 2010), and gene expression levels were presented as FPKM (fragments per kilobase of exon per million fragments mapped) score (Mortazavi *et al.*, 2008). Differentially expressed genes were considered significant at a false discovery rate (FDR) < 0.05, adjusted value of 0.05 (Benjamini and Hochberg, 1995), and |log2 FC| ≥ 1 (FC, posterior fold change). The identified DEGs were used for GO and KEGG enrichment analysis, performed using Goatools (Young *et al.*, 2010) (https://github.com/tanghaibao/GOatools) and KOBAS (http://kobas.cbi.pku.edu.cn/home.do) (Xie *et al.*, 2011) respectively.

### Identification of transcription factors

Transcription factors (TFs) were identified by analyzing InterProScan domain patterns in protein sequences with high coverage and sensitivity using the PlantTFcat analysis tool (http://plantgrn.noble.org/PlantTFcat/) (Dai *et al.*, 2013).

## Results

### Transcriptome sequencing and *de novo* transcriptome assembly

**Figure 1:**
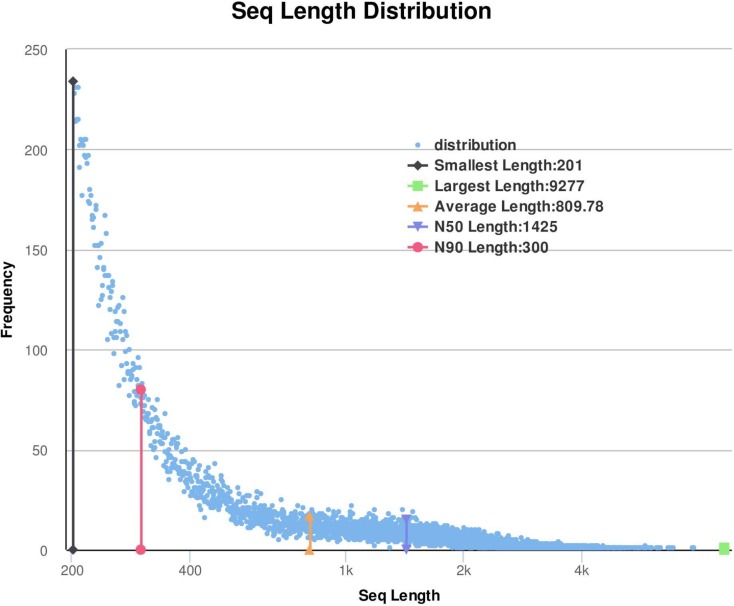
Sequences length distribution.

cDNA samples from leaves and flowers of *D. schraderiana* were sequenced using Illumina sequencing. In total, 28,589,570 raw reads from leaves and 26,126,142 raw reads from flowers were generated. The high-quality raw data was deposited in the NCBI SRA database with accession number SRX3145241 and SRX3145242. The quality of reads is reported in Table S1, including Error, Q20%, Q30%, and GC%. Using the Trinity program, all clean reads were further *de novo* assembled into 48,908 transcripts with an average length of 873.13 bp and an N50 of 1450 bp (Table 1). Then, a total of 40,412 unigenes were generated with the clustering and assembly analysis of transcripts. The length distribution of unigenes is illustrated in Figure 1, indicating the highest concentration in the 1-400 and > 800 length range.

**Table 1:** Summary of sequencing data and *de novo* assembling.

	Total number	Mean Length	GC%	N50	N90
Transcripts	48908	873.13	42.07%	1450	338
Unigenes	40142	809.78	42.22%	1425	300

### Functional annotation and classification

The results of the functional annotation showed that 23,864 (59.45%) of the 40,142 unigenes were annotated against the Nr, Pfam, String, Swissprot, COG, and KEGG databases. As results, 23,723 (59.10%) unigenes had significant marches in the Nr database, 14,572 (36.30%) in the Pfam database, 11,843 (29.50%) in String, 12,978 (32.33%) in Swissprot, 9,950 (24.79%) in KEGG, and 5,921(15.37%) in the COG database (Table 2).

**Table 2:** Summary of functional annotations for unigenes of *D. schraderiana.*

Annotated Database	Unigenes	Frequency
All Annotation	23,864	59.45%
Nr	23,723	59.10%
Pfam	14573	36.30%
String	11,842	29.50%
Swissprot	12,979	32.33%
KEGG	9,950	24.79%
COG	6168	15.37%

**Figure 2:**
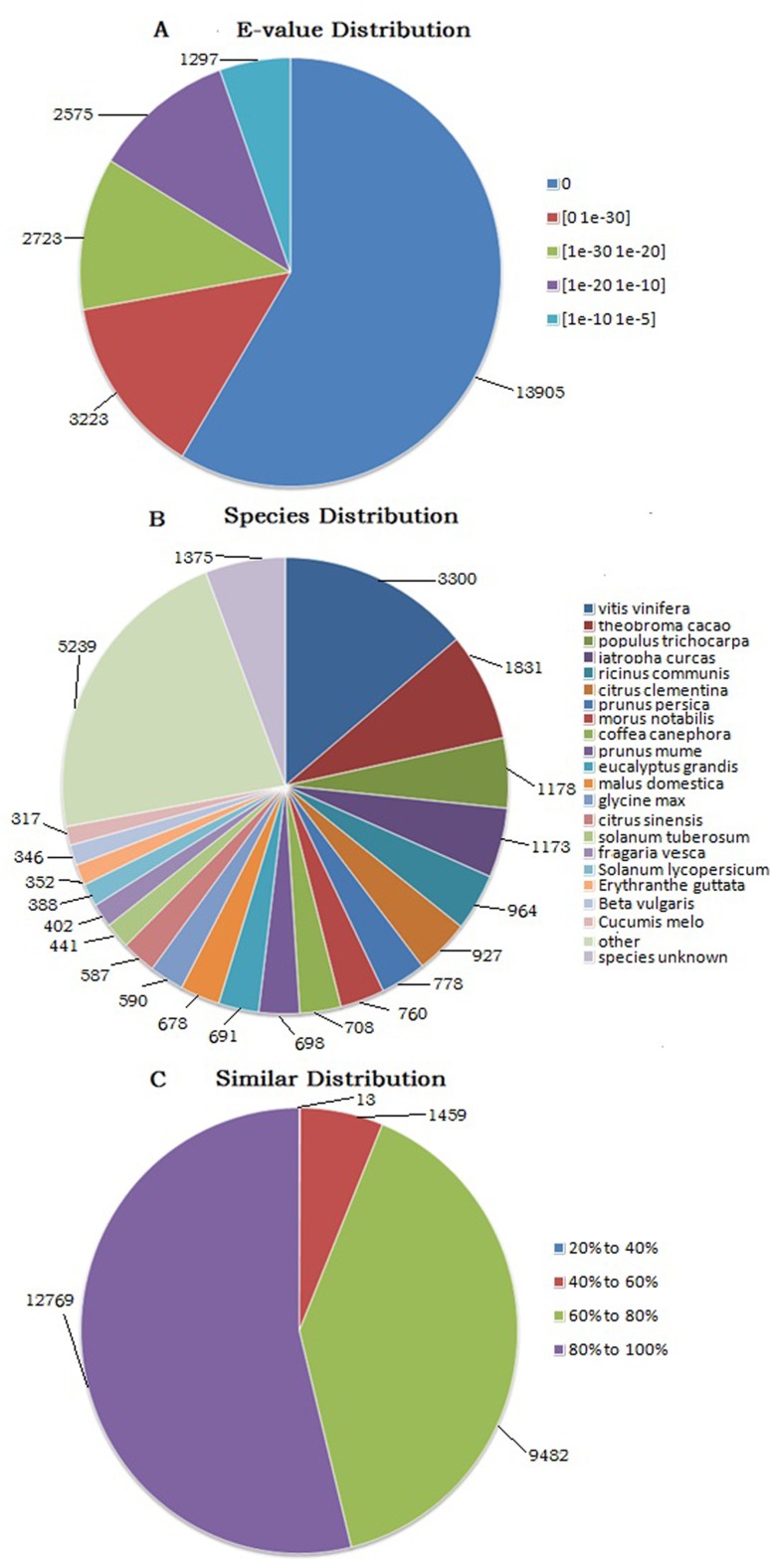
Species distribution of unigenes from *D. schraderiana*. (A) E-value distribution of BLAST hits for each unigene with a cut off E-value of 1.0E-5. (B) Species distribution of top BLAST hits in the Nr database. (C) Similarity distribution of top BLAST hits for each unigene.

Of the annotated sequences in the non-redundant (Nr) protein database, 72.2% displayed significant homology (E value < 1E-30). The E-value distribution is shown in Figure 2A and as shown in Figure 2B, the BLAST search analysis further revealed that a total of 3,300 (13.91%) unigenes had the most similar sequences to proteins from *Vitis vinifera*, followed by *Theobroma cacao* 1,831 (7.72%), and *Populus trichocarpa* 1,178 (4.97%). In Figure 2C, the distribution analysis showed that 53.83% unigenes had a similarity of more than 80%, 39.97% unigenes had a similarity between 60 and 80%, and 6.2% unigenes had a similarity of less than 60%.

**Figure 3:**
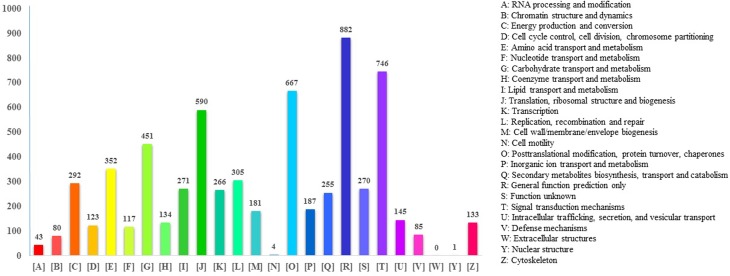
COG functional classification of unigenes of *D. schraderiana*.

All the assembled unigenes were searched against the COG database and classified in clusters of orthologs. Overall, 6,168 unigenes were assigned 25 COG functional classifications with term abbreviations ranging from A to Z (except X). The largest category was the General Function prediction (882, 14.30%); the second category was Signal Transduction mechanisms (746, 12.09%); and the third categories were Post-translational modification, Protein turnover, and Chaperon (667, 10.81%) (Figure 3). Only few unigenes were assigned to Cell motility and Nuclear structure, and no unigene was assigned to the Extracellular structures category.

**Figure 4:**
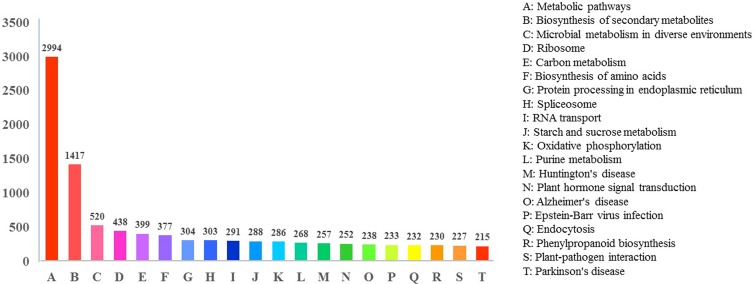
Top 20 KEGG pathways assigned to the assembled transcripts.

The KEGG analysis showed that 9,950 unigenes were assigned to 341 KEGG pathways (Table S2), and these were classified into five larger pathway categories: metabolism (6854), genetic information processing (2,136), environmental information processing (1102), cellular processes (1143), and organism system (1,903) (Figure S1). The top 20 KEGG pathways that contained transcripts are shown in Figure 4. The most highly represented pathway was the Metabolic pathway (2,994 transcripts), followed by Biosynthesis of secondary metabolites (1,417 transcripts).

### Identification and functional characterization of differentially expressed genes (DEGs)

**Figure 5:**
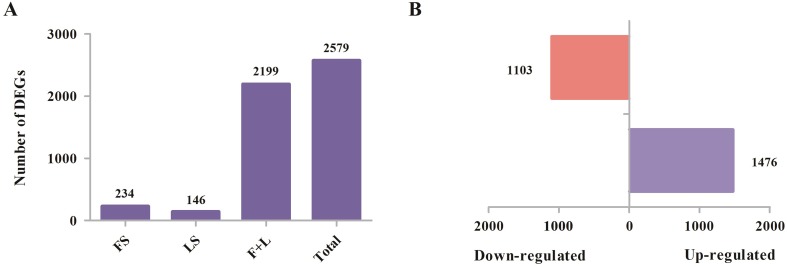
Differential expression gene analysis of *D. schraderiana* transcriptome. (A) Tissue-specific expressed unigenes. FS: flowers-specific; LS: leaves-specific; (F + L): unigenes differentially expressed in common between flowers and leaves. (B) The number of significantly up- and down-regulated unigenes in leaves compared to flowers.

The expression levels of 40,142 unigenes from flower and leaf tissue samples were represented by FPKM scores. As a result, a comparison between the flower and leaf tissue groups resulted in 2,579 significantly (*p*-value < 0.05) DEGs with |log2 FC| ≥ 1 and FDR < 0.05 (Table S3). Statistical analysis of tissue-specifically expressed unigenes indicated that 234 and 146 unigenes were specific to flower and leaf tissues separately. Deep functional studies of these tissue-specific unigenes might provide additional insights into plant development. In addition, 2,199 DEGs commonly exist in both flower and leaf tissues (Figure 5A). In total, 1,476 unigenes exhibited up-regulation and 1,103 unigenes appeared down-regulated in leaves compared to flowers, among these 2,579 DEGs (Figure 5B). The DEGs were further analyzed using the KEGG database to explore their functional categories (Table S4). Among the 2,579 DEGs, 159 were annotated to phenylpropanoid biosynthesis, followed by starch and sucrose metabolism (113), phenylalanine metabolism (106), and carbon metabolism (105).

The GO enrichment analysis and KEGG enrichment analysis of DEGs of *D. schraderiana* was utilized to elucidate functional differences between flower and leaf tissues. In the GO enrichment analysis, the corrected *p*-value of the enriched function should be below 0.05 (Westfall and Young, 1989). Highly enriched DEGs were found as involved in the reductive pentose-phosphate cycle (83.33%), photosynthesis, dark reaction (58.82%), the DNA bending complex (47.22%), nucleosome (47.22%), and protein heterodimerization activity (30.16%) between flowers and leaves (Figure S2). A further study of DEGs focused on the search for significantly enriched biochemical pathways in the KEGG database. Between flowers and leaves, the most significant (*p*-value < 0.05) enriched pathway was related to metabolisms, such as glyoxylate and dicarboxylate metabolism, photosynthesis, and carbon fixation in photosynthetic organisms (Figure S3).

### Transcription Factor (TF) Identification

**Figure 6:**
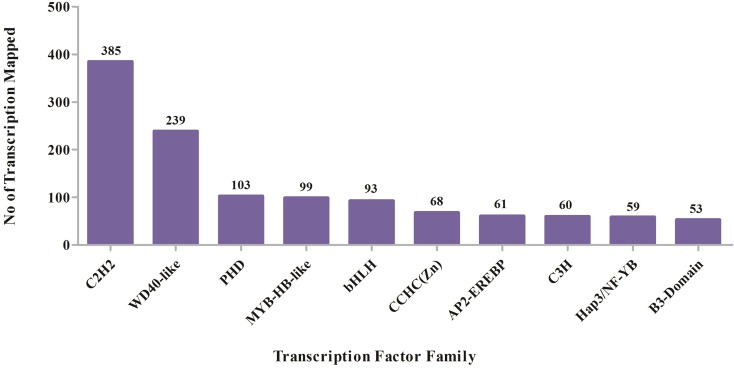
Top 10 transcription factor families.

The biological characteristics corresponding to spatial, temporal, and environmental stimuli are regulated at the transcriptional level via transcription factors, which have been investigated intensively (Yang *et al.*, 2012). In our study, 2,156 unique unigenes with RPKM values were shown to belonged to 97 plant-specific and plant-non-specific transcription factor families (Table S5). Top 10 transcription factor families are shown in Figure 6. The C2H2 transcription factor family had the highest number of members (385) among all unigenes, followed by WD40-like (239), PHD (103), MYB-HB-like (99), and bHLH (93). Among the identified unigenes, we found that 255 unigenes distributed in 55 transcription factor families exhibited significant differential expression levels. Most significant differentially expressed unigenes (26) represented in C2H2 transcription factor family, which acts as trans-regulators of gene expression in cellular processes such as differentiation and development (Razin *et al.*, 2012).

## Discussion

Before this study, no nucleotide sequence from *D. schraderiana* was available in public databases, which limited research on molecular mechanisms of secondary metabolites biosynthesis, physiological adaptation, growth, etc. Nowadays, an increasing number of studies have proved that high-throughput sequence and *de novo* assembly could be an effective technique for non-model plants to identify novel genes and produce massive sequences at a low cost. (Li *et al.*, 2016; Moraortiz *et al.*, 2016).

Here, an Illumina Hiseq4000 sequencing platform was employed to sequence RNA from flower and leaf tissues, providing the first transcriptome information for *D. schraderiana*, which can now be extensively employed in research for novel gene discoveries, comparative genomics, functional genomics, and phylogenetics. In this study, we have obtained 40,142 unigenes with a mean length of 873.13 bp, suggesting that our assembly has high quality according to the criterion for evaluating the accuracy of an assembly. The unigenes were matched with unique known proteins in public databases to predict the potential functions. As a result, 59.45% were successfully annotated, suggesting their relatively conserved functions. The non-annotated unigenes may possess poorly conserved regions (Hou *et al.*, 2011).

Similarity analyses in Nr database indicated that the unigenes of *D. schraderiana* had the highest homologies in cDNA sequences of *Vitis vinifera* (13.93%), *Theobroma cacao* (7.73%), and *Populus trichocarpa* (4.97%). According to the Angiosperm Phylogeny Group III (APG III), *V. vinifera*, *T. cacao*, and *P. trichocarpa* all belong to Dicotyledoneae, as is also the case with *D. schraderiana*. Furthermore, reference genome sequences of *V. vinifera*, *T. cacao*, and *P. trichocarpa* are available in the public databases (Jaillon *et al.*, 2007; Wullschleger *et al.*, 2013; Maximova *et al.*, 2014). Therefore, *D. schraderiana* and these three plant species showed similar genome sequences due to their relatively close relationship.

With respect to the KEGG analysis, *D. schraderiana* unigenes were mainly related to metabolic pathways (2,994 transcripts) and biosynthesis of secondary metabolites (1,417 transcripts). Additionally, the transcriptomic data from flower and leaf tissues showed that 1,476 unigenes exhibited up-regulation, 1,103 unigenes were down-regulated in leaves compared to flowers, and 234 and 146 unigenes were specific to flower and leaf tissues separately. In both the GO and KEGG enrichment analyses, photosynthesis and energy metabolism were enriched, which is likely due to the leaves vital function of photosynthesis performed by chlorophyll. Besides, 2,156 unique unigenes belonged to 97 transcription factor families.

Based on the KEGG analysis, 28 unigenes related to terpenoid backbone biosynthesis of *D. schraderiana* were identified, involving all enzymes in the MVA and MEP pathways, except for 1-hydroxy-2-methyl-2-(E)-butenyl-4-diphosphate reductase (HDR) (Table S6). Additionally, 10 unigenes were predicted to encode two known monoterpene synthases, including (3S)-linalool synthase and (+)-neomenthol dehydrogenase (Table S7). Eight other unigenes were annotated to encode three enzymes participating in sesquiterpene biosynthesis, including valencene/7-epi-alpha-selinene synthase, (-)-germacrene D synthase, and NAD^+^-dependent farnesol dehydrogenase. The expression levels of these unigenes and their FPKM values were identified and estimated. Unigene c12686_g1 annotated to (+)-neomenthol dehydrogenase and unigene c7041_g2 annotated to (-)-germacrene D synthase showed significant differential expression between flowers and leaves (Table S7). And unigene c12686_g1 showed a higher RPKM value in leaf tissue compared to flower tissue, while unigene c7041_g2 had high abundance in flowers. Additional studies involving intensive molecular and proteomic analyses should now be carried out to validate these gene function predictions.

## Conclusion

*D. schraderiana* is a potential medicinal plant that can adapt to alpine hypoxia and high ambient ultraviolet radiation. In this study, our transcriptome analysis generated 40,142 unigenes, of which 59.45% were aligned to sequences in databases. This first comprehensive transcriptome analysis markedly expands our understanding of molecular mechanism in *D. schraderiana*. It also provides a large number of candidate genes potentially involved in the biosynthesis of secondary metabolites and could be used in research on the adaptation to the extreme environment of the Qinghai-Tibet Plateau.

## Supplementary material

The following online material is available for this study:

Table S1 Quality of sequencing.

Table S2 KEGG classification for unigenes of *D. schraderiana*.

Table S3 Unigenes (2579) with detectable expression level.

Table S4 KEGG annotation of DEGs

Table S5 List of predicted transcription factors.

Table S6 Unigenes involved in terpenoid backbone biosynthesis.

Table S7 Unigenes annotated to mono-TPS and sesqui-TPS in *D. schraderiana*.

Figure S1 Pathway assignment based on the KEGG database.

Figure S2 Flowers vs Leaves.DE.list.GO.enrichment.details.

Figure S3 Flowers vs Leaves.DE.list.KEGG.enrichment.details.

*Associate Editor: Houtan Noushmehr*
