# Supplementary material for: De novo transcriptome analysis of Tibetan medicinal plant Dysphania schraderiana
Source: Genet Mol Biol. 2019 Jun 13;42(2):480–7. doi: 10.1590/1678-4685-GMB-2018-0033 (PMC6726160; doi:10.1590/1678-4685-GMB-2018-0033)
Supplement: Supplementary file 1 [file 1415-4757-GMB-1678-4685-GMB-2018-0033-20190513-suppl1.pdf]

Supplementary Material to "*De novo* transcriptome analysis of Tibetan medicinal plant *Dysphania schraderiana*"

Table S1 - Quality of sequencing.

sample	Raw data		Clean data	
	flowers	leaves	flowers	leaves
reads	26,126,142	28,589,570	24,187,800	27,832,004
Error	0.0217	0.0146	0.0147	0.0125
Q20%	92.68	96.33	96.84	97.86
Q30%	96.33	90.81	90.94	93.20
GC%	45.92	46.83	45.57	46.51
