# Supplementary material for: De novo transcriptome analysis of Tibetan medicinal plant Dysphania schraderiana
Source: Genet Mol Biol. 2019 Jun 13;42(2):480–7. doi: 10.1590/1678-4685-GMB-2018-0033 (PMC6726160; doi:10.1590/1678-4685-GMB-2018-0033)
Supplement: Supplementary file 2 [file 1415-4757-GMB-1678-4685-GMB-2018-0033-20190513-suppl2.pdf]

Supplementary Material to "*De novo* transcriptome analysis of Tibetan medicinal plant *Dysphania schraderiana*"

Table S2 - KEGG classification for unigenes of *D. schraderiana*.

Pathway ID	Pathway	Number of seqs
path:ko04977	Vitamin digestion and absorption	6
path:ko04664	Fc epsilon RI signaling pathway	25
path:ko00563	Glycosylphosphatidylinositol(GPI)-anchor biosynthesis	63
path:ko00830	Retinol metabolism	28
path:ko05222	Small cell lung cancer	19
path:ko04660	T cell receptor signaling pathway	33
path:ko00650	Butanoate metabolism	36
path:ko04911	Insulin secretion	7
path:ko05030	Cocaine addiction	2
path:ko00945	Stilbenoid, diarylheptanoid and gingerol biosynthesis	34
path:ko03430	Mismatch repair	73
path:ko04745	Phototransduction - fly	18
path:ko00760	Nicotinate and nicotinamide metabolism	38
path:ko00514	Other types of O-glycan biosynthesis	7
path:ko04261	Adrenergic signaling in cardiomyocytes	65
path:ko05203	Viral carcinogenesis	179
path:ko04974	Protein digestion and absorption	40
path:ko05131	Shigellosis	72
path:ko04145	Phagosome	145
path:ko03060	Protein export	65
path:ko00072	Synthesis and degradation of ketone bodies	11
path:ko04666	Fc gamma R-mediated phagocytosis	113
path:ko03440	Homologous recombination	109
path:ko05145	Toxoplasmosis	168
path:ko04112	Cell cycle - Caulobacter	26
path:ko00930	Caprolactam degradation	3
path:ko04060	Cytokine-cytokine receptor interaction	1
path:ko00430	Taurine and hypotaurine metabolism	16
path:ko01200	Carbon metabolism	399
path:ko04932	Non-alcoholic fatty liver disease (NAFLD)	174
path:ko04750	Inflammatory mediator regulation of TRP channels	24
path:ko00513	Various types of N-glycan biosynthesis	51
path:ko00130	Ubiquinone and other terpenoid-quinone biosynthesis	67
path:ko04966	Collecting duct acid secretion	27
path:ko03410	Base excision repair	77
path:ko04740	Olfactory transduction	12
path:ko05160	Hepatitis C	40
path:ko05020	Prion diseases	30
path:ko04391	Hippo signaling pathway - fly	43
path:ko00240	Pyrimidine metabolism	217

Pathway ID	Pathway	Number of seqs
path:ko00900	Terpenoid backbone biosynthesis	85
path:ko05223	Non-small cell lung cancer	22
path:ko04962	Vasopressin-regulated water reabsorption	45
path:ko04340	Hedgehog signaling pathway	15
path:ko04130	SNARE interactions in vesicular transport	54
path:ko00624	Polycyclic aromatic hydrocarbon degradation	6
path:ko05218	Melanoma	23
path:ko00909	Sesquiterpenoid and triterpenoid biosynthesis	21
path:ko00051	Fructose and mannose metabolism	100
path:ko04621	NOD-like receptor signaling pathway	29
path:ko05152	Tuberculosis	203
path:ko05012	Parkinson's disease	215
path:ko04921	Oxytocin signaling pathway	91
path:ko04011	MAPK signaling pathway - yeast	16
path:ko03050	Proteasome	76
path:ko04120	Ubiquitin mediated proteolysis	190
path:ko05144	Malaria	1
path:ko04920	Adipocytokine signaling pathway	47
path:ko00520	Amino sugar and nucleotide sugar metabolism	161
path:ko05215	Prostate cancer	55
path:ko00040	Pentose and glucuronate interconversions	127
path:ko00450	Selenocompound metabolism	24
path:ko00620	Pyruvate metabolism	133
path:ko00310	Lysine degradation	77
path:ko04973	Carbohydrate digestion and absorption	33
path:ko05031	Amphetamine addiction	39
path:ko04022	cGMP-PKG signaling pathway	76
path:ko05323	Rheumatoid arthritis	34
path:ko04270	Vascular smooth muscle contraction	40
path:ko00362	Benzoate degradation	11
path:ko00010	Glycolysis / Gluconeogenesis	209
path:ko04612	Antigen processing and presentation	98
path:ko00640	Propanoate metabolism	64
path:ko03013	RNA transport	291
path:ko03070	Bacterial secretion system	29
path:ko03030	DNA replication	77
path:ko05169	Epstein-Barr virus infection	233
path:ko04940	Type I diabetes mellitus	20
path:ko04380	Osteoclast differentiation	38
path:ko01120	Microbial metabolism in diverse environments	520
path:ko04918	Thyroid hormone synthesis	32
path:ko00904	Diterpenoid biosynthesis	18
path:ko00966	Glucosinolate biosynthesis	2
path:ko00591	Linoleic acid metabolism	28

Pathway ID	Pathway	Number of seqs
path:ko04725	Cholinergic synapse	16
path:ko04210	Apoptosis	113
path:ko00790	Folate biosynthesis	41
path:ko05220	Chronic myeloid leukemia	23
path:ko01110	Biosynthesis of secondary metabolites	1417
path:ko04070	Phosphatidylinositol signaling system	112
path:ko04728	Dopaminergic synapse	49
path:ko00052	Galactose metabolism	134
path:ko04620	Toll-like receptor signaling pathway	112
path:ko04012	ErbB signaling pathway	26
path:ko00660	C5-Branched dibasic acid metabolism	12
path:ko00523	Polyketide sugar unit biosynthesis	1
path:ko04711	Circadian rhythm - fly	4
path:ko04113	Meiosis - yeast	144
path:ko00970	Aminoacyl-tRNA biosynthesis	95
path:ko00643	Styrene degradation	5
path:ko00983	Drug metabolism - other enzymes	35
path:ko04111	Cell cycle - yeast	173
path:ko00380	Tryptophan metabolism	63
path:ko04370	VEGF signaling pathway	42
path:ko00260	Glycine, serine and threonine metabolism	95
path:ko00300	Lysine biosynthesis	14
path:ko00053	Ascorbate and aldarate metabolism	71
path:ko03020	RNA polymerase	81
path:ko00531	Glycosaminoglycan degradation	36
path:ko01100	Metabolic pathways	2994
path:ko01210	2-Oxocarboxylic acid metabolism	95
path:ko05132	Salmonella infection	68
path:ko00942	Anthocyanin biosynthesis	2
path:ko05214	Glioma	37
path:ko04720	Long-term potentiation	55
path:ko04976	Bile secretion	62
path:ko05322	Systemic lupus erythematosus	56
path:ko04020	Calcium signaling pathway	78
path:ko04914	Progesterone-mediated oocyte maturation	89
path:ko00562	Inositol phosphate metabolism	122
path:ko04622	RIG-I-like receptor signaling pathway	15
path:ko00071	Fatty acid degradation	80
path:ko00903	Limonene and pinene degradation	24
path:ko00710	Carbon fixation in photosynthetic organisms	124
path:ko00600	Sphingolipid metabolism	50
path:ko02020	Two-component system	46
path:ko04919	Thyroid hormone signaling pathway	89
path:ko05168	Herpes simplex infection	119

Pathway ID	Pathway	Number of seqs
path:ko00670	One carbon pool by folate	32
path:ko04970	Salivary secretion	17
path:ko05110	Vibrio cholerae infection	72
path:ko04540	Gap junction	62
path:ko02030	Bacterial chemotaxis	3
path:ko04910	Insulin signaling pathway	127
path:ko04742	Taste transduction	2
path:ko01051	Biosynthesis of ansamycins	7
path:ko00590	Arachidonic acid metabolism	21
path:ko00603	Glycosphingolipid biosynthesis - globo series	16
path:ko04110	Cell cycle	204
path:ko00623	Toluene degradation	4
path:ko04068	FoxO signaling pathway	85
path:ko03022	Basal transcription factors	94
path:ko00965	Betalain biosynthesis	5
path:ko05217	Basal cell carcinoma	9
path:ko04142	Lysosome	125
path:ko00550	Peptidoglycan biosynthesis	5
path:ko05130	Pathogenic Escherichia coli infection	76
path:ko00950	Isoquinoline alkaloid biosynthesis	31
path:ko03010	Ribosome	438
path:ko00510	N-Glycan biosynthesis	81
path:ko00750	Vitamin B6 metabolism	16
path:ko05034	Alcoholism	100
path:ko04013	MAPK signaling pathway - fly	15
path:ko04310	Wnt signaling pathway	69
path:ko00626	Naphthalene degradation	14
path:ko00540	Lipopolysaccharide biosynthesis	17
path:ko05142	Chagas disease (American trypanosomiasis)	119
path:ko04930	Type II diabetes mellitus	48
path:ko00340	Histidine metabolism	41
path:ko00860	Porphyrin and chlorophyll metabolism	75
path:ko04512	ECM-receptor interaction	27
path:ko03018	RNA degradation	163
path:ko04913	Ovarian steroidogenesis	9
path:ko04611	Platelet activation	58
path:ko04721	Synaptic vesicle cycle	77
path:ko05206	MicroRNAs in cancer	128
path:ko04150	mTOR signaling pathway	65
path:ko04964	Proximal tubule bicarbonate reclamation	11
path:ko04520	Adherens junction	52
path:ko05213	Endometrial cancer	30
path:ko04670	Leukocyte transendothelial migration	23
path:ko01212	Fatty acid metabolism	118

Pathway ID	Pathway	Number of seqs
path:ko03008	Ribosome biogenesis in eukaryotes	147
path:ko00680	Methane metabolism	95
path:ko00561	Glycerolipid metabolism	90
path:ko04810	Regulation of actin cytoskeleton	122
path:ko00982	Drug metabolism - cytochrome P450	71
path:ko04064	NF-kappa B signaling pathway	102
path:ko04726	Serotonergic synapse	16
path:ko00410	beta-Alanine metabolism	89
path:ko00250	Alanine, aspartate and glutamate metabolism	82
path:ko01040	Biosynthesis of unsaturated fatty acids	54
path:ko00312	beta-Lactam resistance	2
path:ko04712	Circadian rhythm - plant	62
path:ko04662	B cell receptor signaling pathway	42
path:ko05014	Amyotrophic lateral sclerosis (ALS)	43
path:ko04062	Chemokine signaling pathway	33
path:ko00440	Phosphonate and phosphinate metabolism	11
path:ko03450	Non-homologous end-joining	21
path:ko04626	Plant-pathogen interaction	227
path:ko04972	Pancreatic secretion	49
path:ko00780	Biotin metabolism	21
path:ko05210	Colorectal cancer	53
path:ko05410	Hypertrophic cardiomyopathy (HCM)	31
path:ko04668	TNF signaling pathway	24
path:ko05010	Alzheimer's disease	238
path:ko05216	Thyroid cancer	22
path:ko05162	Measles	149
path:ko04723	Retrograde endocannabinoid signaling	22
path:ko04917	Prolactin signaling pathway	22
path:ko04014	Ras signaling pathway	92
path:ko04514	Cell adhesion molecules (CAMs)	1
path:ko00253	Tetracycline biosynthesis	9
path:ko00471	D-Glutamine and D-glutamate metabolism	3
path:ko04144	Endocytosis	232
path:ko04260	Cardiac muscle contraction	64
path:ko00740	Riboflavin metabolism	15
path:ko00401	Novobiocin biosynthesis	9
path:ko00532	Glycosaminoglycan biosynthesis - chondroitin sulfate / dermatan sulfate	4
path:ko04623	Cytosolic DNA-sensing pathway	48
path:ko04024	cAMP signaling pathway	100
path:ko00908	Zeatin biosynthesis	47
path:ko05120	Epithelial cell signaling in Helicobacter pylori infection	42
path:ko00604	Glycosphingolipid biosynthesis - ganglio series	12
path:ko04360	Axon guidance	49
path:ko04530	Tight junction	52

Pathway ID	Pathway	Number of seqs
path:ko00730	Thiamine metabolism	16
path:ko04320	Dorso-ventral axis formation	25
path:ko00254	Aflatoxin biosynthesis	5
path:ko04630	Jak-STAT signaling pathway	8
path:ko00940	Phenylpropanoid biosynthesis	230
path:ko00981	Insect hormone biosynthesis	2
path:ko04915	Estrogen signaling pathway	84
path:ko00627	Aminobenzoate degradation	19
path:ko00364	Fluorobenzoate degradation	4
path:ko00910	Nitrogen metabolism	44
path:ko00902	Monoterpenoid biosynthesis	19
path:ko00062	Fatty acid elongation	45
path:ko04610	Complement and coagulation cascades	1
path:ko00195	Photosynthesis	77
path:ko05414	Dilated cardiomyopathy	11
path:ko04722	Neurotrophin signaling pathway	149
path:ko00960	Tropane, piperidine and pyridine alkaloid biosynthesis	35
path:ko05133	Pertussis	118
path:ko05033	Nicotine addiction	8
path:ko00511	Other glycan degradation	44
path:ko04614	Renin-angiotensin system	7
path:ko04141	Protein processing in endoplasmic reticulum	304
path:ko03015	mRNA surveillance pathway	197
path:ko00524	Butirosin and neomycin biosynthesis	16
path:ko00906	Carotenoid biosynthesis	37
path:ko05340	Primary immunodeficiency	3
path:ko00480	Glutathione metabolism	121
path:ko04730	Long-term depression	20
path:ko04710	Circadian rhythm	36
path:ko00030	Pentose phosphate pathway	86
path:ko00361	Chlorocyclohexane and chlorobenzene degradation	4
path:ko04114	Oocyte meiosis	163
path:ko05164	Influenza A	177
path:ko05166	HTLV-I infection	181
path:ko05100	Bacterial invasion of epithelial cells	59
path:ko04390	Hippo signaling pathway	54
path:ko04350	TGF-beta signaling pathway	42
path:ko05416	Viral myocarditis	32
path:ko05146	Amoebiasis	50
path:ko04960	Aldosterone-regulated sodium reabsorption	15
path:ko00281	Geraniol degradation	7
path:ko05205	Proteoglycans in cancer	85
path:ko00140	Steroid hormone biosynthesis	19
path:ko04330	Notch signaling pathway	22

Pathway ID	Pathway	Number of seqs
path:ko04510	Focal adhesion	84
path:ko00943	Isoflavonoid biosynthesis	2
path:ko05161	Hepatitis B	52
path:ko00770	Pantothenate and CoA biosynthesis	48
path:ko05204	Chemical carcinogenesis	69
path:ko00565	Ether lipid metabolism	61
path:ko04727	GABAergic synapse	44
path:ko04724	Glutamatergic synapse	76
path:ko05221	Acute myeloid leukemia	21
path:ko05200	Pathways in cancer	118
path:ko04075	Plant hormone signal transduction	252
path:ko04912	GnRH signaling pathway	63
path:ko00785	Lipoic acid metabolism	8
path:ko00400	Phenylalanine, tyrosine and tryptophan biosynthesis	78
path:ko00460	Cyanoamino acid metabolism	73
path:ko04146	Peroxisome	155
path:ko04713	Circadian entrainment	26
path:ko00280	Valine, leucine and isoleucine degradation	110
path:ko00625	Chloroalkane and chloroalkene degradation	31
path:ko00290	Valine, leucine and isoleucine biosynthesis	31
path:ko05202	Transcriptional misregulation in cancer	65
path:ko00534	Glycosaminoglycan biosynthesis - heparan sulfate / heparin	7
path:ko03420	Nucleotide excision repair	106
path:ko00564	Glycerophospholipid metabolism	150
path:ko03040	Spliceosome	303
path:ko00073	Cutin, suberine and wax biosynthesis	41
path:ko00500	Starch and sucrose metabolism	288
path:ko04152	AMPK signaling pathway	130
path:ko00944	Flavone and flavonol biosynthesis	5
path:ko04015	Rap1 signaling pathway	56
path:ko05219	Bladder cancer	19
path:ko00941	Flavonoid biosynthesis	49
path:ko00190	Oxidative phosphorylation	286
path:ko05211	Renal cell carcinoma	34
path:ko04140	Regulation of autophagy	57
path:ko04971	Gastric acid secretion	15
path:ko00061	Fatty acid biosynthesis	59
path:ko04975	Fat digestion and absorption	11
path:ko04115	p53 signaling pathway	60
path:ko00330	Arginine and proline metabolism	136
path:ko00905	Brassinosteroid biosynthesis	13
path:ko04010	MAPK signaling pathway	84
path:ko00270	Cysteine and methionine metabolism	128
path:ko00100	Steroid biosynthesis	41

Pathway ID	Pathway	Number of seqs
path:ko04650	Natural killer cell mediated cytotoxicity	38
path:ko00020	Citrate cycle (TCA cycle)	85
path:ko00901	Indole alkaloid biosynthesis	1
path:ko03460	Fanconi anemia pathway	146
path:ko00196	Photosynthesis - antenna proteins	23
path:ko05134	Legionellosis	120
path:ko01220	Degradation of aromatic compounds	16
path:ko05032	Morphine addiction	11
path:ko04151	PI3K-Akt signaling pathway	154
path:ko00350	Tyrosine metabolism	60
path:ko04080	Neuroactive ligand-receptor interaction	7
path:ko00630	Glyoxylate and dicarboxylate metabolism	96
path:ko00232	Caffeine metabolism	9
path:ko05016	Huntington's disease	257
path:ko04744	Phototransduction	13
path:ko02010	ABC transporters	78
path:ko03320	PPAR signaling pathway	56
path:ko00720	Carbon fixation pathways in prokaryotes	44
path:ko00920	Sulfur metabolism	33
path:ko00360	Phenylalanine metabolism	155
path:ko04978	Mineral absorption	26
path:ko00363	Bisphenol degradation	6
path:ko01230	Biosynthesis of amino acids	377
path:ko04961	Endocrine and other factor-regulated calcium reabsorption	39
path:ko04066	HIF-1 signaling pathway	85
path:ko00120	Primary bile acid biosynthesis	1
path:ko05412	Arrhythmogenic right ventricular cardiomyopathy (ARVC)	11
path:ko04916	Melanogenesis	38
path:ko04122	Sulfur relay system	20
path:ko00230	Purine metabolism	268
path:ko00980	Metabolism of xenobiotics by cytochrome P450	77
path:ko00592	alpha-Linolenic acid metabolism	53
path:ko01053	Biosynthesis of siderophore group nonribosomal peptides	8
path:ko05140	Leishmaniasis	101
path:ko05212	Pancreatic cancer	35
path:ko05143	African trypanosomiasis	13
path:ko00521	Streptomycin biosynthesis	21
