# Supplementary material for: De novo transcriptome analysis of Tibetan medicinal plant Dysphania schraderiana
Source: Genet Mol Biol. 2019 Jun 13;42(2):480–7. doi: 10.1590/1678-4685-GMB-2018-0033 (PMC6726160; doi:10.1590/1678-4685-GMB-2018-0033)
Supplement: Supplementary file 4 [file 1415-4757-GMB-1678-4685-GMB-2018-0033-20190513-suppl3.pdf]

Supplementary Material to "*De novo* transcriptome analysis of Tibetan medicinal plant *Dysphania schraderiana*"

Table S3 - 2579 unigenes with detectable expression level.

seq_id	Fpkm of flower	Fpkm of leaf	log2FC (leaf/flower)	p-value	FDR	regulate
c17697_g1	3.813	12468.88	11.64	1.85E-14	3.48E-10	up
c3959_g1	10.775	21514.206	10.95	1.36E-13	1.28E-09	up
c1833_g1	2.088	3024.74	10.43	1.28E-12	7.99E-09	up
c34837_g1	12.662	12814.858	9.97	3.26E-12	1.53E-08	up
c17833_g1	2.669	2693.668	9.93	6.59E-12	2.47E-08	up
c275_g1	5.159	3158.492	9.23	3.08E-11	8.89E-08	up
c7221_g1	14.07	8086.033	9.16	3.65E-11	8.89E-08	up
c30011_g1	51.145	28848.026	9.14	3.83E-11	8.89E-08	up
c3120_g1	31.173	17223.561	9.11	4.26E-11	8.89E-08	up
c30892_g1	0	676.739	12.72	5.19E-11	9.73E-08	up
c30268_g1	1.044	992.957	9.76	6.51E-11	1.09E-07	up
c30735_g1	4.378	2234.661	8.96	7.27E-11	1.09E-07	up
c26783_g1	20.924	12881.915	9.26	7.94E-11	1.09E-07	up
c277_g1	0.758	474.611	9.11	8.51E-11	1.09E-07	up
c26056_g1	9.36	4482.017	8.89	8.68E-11	1.09E-07	up
c34475_g1	18.998	8402.354	8.78	1.25E-10	1.47E-07	up
c21696_g1	13.846	6019.034	8.75	1.40E-10	1.54E-07	up
c9654_g1	0.193	104.38	8.48	1.68E-10	1.75E-07	up
c17644_g1	31.815	12294.495	8.59	2.05E-10	2.02E-07	up
c13183_g1	45.166	0	-8.82	3.20E-10	3.00E-07	down
c30125_g1	1.122	431.16	8.46	3.92E-10	3.50E-07	up
c7592_g1	0	51.337	9.01	6.44E-10	5.50E-07	up
c34496_g1	4.494	1283.448	8.13	1.04E-09	8.47E-07	up
c26017_g1	4.177	1152.824	8.07	1.09E-09	8.54E-07	up
c13151_g1	144.563	0.129	-9.3	1.19E-09	8.95E-07	down
c1163_g1	0.101	55.021	8.1	1.39E-09	1.00E-06	up
c21785_g1	8.455	2035.467	7.89	1.79E-09	1.24E-06	up
c22449_g1	0.642	173.338	7.87	2.39E-09	1.60E-06	up
c18347_g1	4.2	986.816	7.84	2.50E-09	1.62E-06	up
c30414_g1	0.309	121.691	8.22	3.02E-09	1.89E-06	up
c7488_g1	0.425	145.284	8.11	3.15E-09	1.91E-06	up
c17990_g1	4.502	1018.994	7.79	3.31E-09	1.94E-06	up
c30040_g1	6.033	1247.534	7.67	3.85E-09	2.19E-06	up
c34942_g1	0.062	52.436	8.34	5.26E-09	2.90E-06	up
c5415_g1	0.727	150.275	7.51	1.16E-08	6.23E-06	up
c13324_g1	13.235	2090.851	7.29	1.24E-08	6.45E-06	up
c30210_g1	8.687	1492.933	7.41	1.40E-08	7.08E-06	up

seq_id	Fpkm of flower	Fpkm of leaf	log2FC (leaf/flower)	p-value	FDR	regulate
c1881_g1	291.021	43061.726	7.21	1.46E-08	7.22E-06	up
c7174_g1	0.309	87.225	7.74	1.53E-08	7.36E-06	up
c25987_g1	19.616	2881.925	7.19	1.64E-08	7.70E-06	up
c373_g1	0	17.052	7.42	2.28E-08	1.04E-05	up
c10262_g2	0.379	111.025	7.86	2.33E-08	1.04E-05	up
c11154_g1	0.294	79.804	7.66	2.46E-08	1.07E-05	up
c34883_g1	11.781	1560.947	7.04	2.72E-08	1.16E-05	up
c26376_g1	3.775	503.957	7.02	2.99E-08	1.25E-05	up
c14473_g1	1.655	244.364	7.12	3.48E-08	1.40E-05	up
c15072_g1	0.634	104.277	7.15	3.57E-08	1.40E-05	up
c6064_g1	0.108	28.157	7.09	3.58E-08	1.40E-05	up
c5719_g1	0	17.556	7.46	4.23E-08	1.62E-05	up
c8183_g1	0.572	84.975	6.98	4.64E-08	1.74E-05	up
c29963_g1	0.89	159.221	7.33	4.87E-08	1.79E-05	up
c21900_g1	0.07	33.522	7.63	5.06E-08	1.83E-05	up
c28495_g1	0	55.978	9.13	5.20E-08	1.84E-05	up
c6943_g1	0	96.042	9.91	6.16E-08	2.14E-05	up
c19312_g1	0.333	65.273	7.24	6.81E-08	2.32E-05	up
c21975_g1	27.011	2814.958	6.7	7.79E-08	2.61E-05	up
c9585_g1	0.093	16.871	6.46	8.09E-08	2.66E-05	up
c7937_g1	0.124	26.089	6.87	8.95E-08	2.89E-05	up
c30179_g1	13.97	1385.062	6.62	9.83E-08	3.12E-05	up
c26438_g1	6.049	583.516	6.57	1.10E-07	3.37E-05	up
c12681_g2	0	23.852	7.9	1.11E-07	3.37E-05	up
c10155_g1	0.874	92.861	6.58	1.11E-07	3.37E-05	up
c27021_g1	0.255	38.9	6.78	1.17E-07	3.49E-05	up
c31412_g1	0.425	50.626	6.59	1.26E-07	3.64E-05	up
c36392_g1	5.933	593.755	6.62	1.28E-07	3.64E-05	up
c10424_g1	0	17.621	7.47	1.28E-07	3.64E-05	up
c14452_g1	54.402	0.142	-7.82	1.63E-07	4.57E-05	down
c8317_g1	0.518	56.482	6.52	1.77E-07	4.76E-05	up
c13431_g1	45.839	3879.446	6.4	1.77E-07	4.76E-05	up
c14903_g1	0	22.327	7.81	1.77E-07	4.76E-05	up
c30643_g1	0.124	22.159	6.63	1.89E-07	5.00E-05	up
c31555_g1	2.591	261.106	6.6	1.95E-07	5.07E-05	up
c22006_g1	148.144	12197.807	6.36	1.98E-07	5.09E-05	up
c35591_g1	0	16.418	7.37	2.04E-07	5.14E-05	up
c4010_g1	0.456	81.64	7.2	2.06E-07	5.14E-05	up
c12708_g1	17.319	0	-7.44	2.09E-07	5.15E-05	down
c7538_g1	0.116	42.998	7.64	2.24E-07	5.42E-05	up
c3868_g1	0	16.936	7.41	2.27E-07	5.42E-05	up
c21867_g1	74.08	0.556	-6.82	2.33E-07	5.42E-05	down

seq_id	Fpkm of flower	Fpkm of leaf	log2FC (leaf/flower)	p-value	FDR	regulate
c13263_g1	16.197	1291.321	6.31	2.34E-07	5.42E-05	up
c30678_g1	0.897	81.084	6.35	2.36E-07	5.42E-05	up
c18689_g1	0.472	49.139	6.43	2.37E-07	5.42E-05	up
c21723_g1	2.908	235.172	6.29	2.42E-07	5.47E-05	up
c31395_g1	2.529	202.064	6.26	2.92E-07	6.53E-05	up
c10677_g1	242.946	2.353	-6.63	3.12E-07	6.83E-05	down
c8255_g1	0.394	55.616	6.82	3.13E-07	6.83E-05	up
c22245_g1	0	32.591	8.35	3.29E-07	7.09E-05	up
c247_g1	106.212	7748.251	6.19	3.37E-07	7.19E-05	up
c14782_g1	77.452	0.401	-7.27	3.59E-07	7.57E-05	down
c19552_g1	5.956	476.783	6.3	3.66E-07	7.63E-05	up
c14560_g1	0	15.152	7.25	3.96E-07	8.17E-05	up
c1092_g1	0.162	19.263	6.21	4.14E-07	8.38E-05	up
c35017_g1	0.333	50.393	6.87	4.20E-07	8.38E-05	up
c9488_g1	1.191	103.023	6.32	4.24E-07	8.38E-05	up
c30411_g1	12.67	886.327	6.12	4.24E-07	8.38E-05	up
c1827_g1	0.394	46.618	6.56	4.40E-07	8.61E-05	up
c15077_g1	0	15.591	7.29	4.47E-07	8.64E-05	up
c31263_g1	0.572	85.247	6.99	4.56E-07	8.74E-05	up
c8955_g1	0	34.815	8.45	4.93E-07	9.35E-05	up
c35119_g1	4.718	338.867	6.14	5.10E-07	9.56E-05	up
c21672_g1	5.167	351.382	6.06	5.16E-07	9.59E-05	up
c13352_g1	3.024	204.83	6.04	5.51E-07	1.01E-04	up
c8615_g1	2.042	138.872	6.02	5.70E-07	1.04E-04	up
c35475_g1	0.603	52.397	6.22	5.77E-07	1.04E-04	up
c12664_g1	33.138	0	-8.38	5.89E-07	1.04E-04	down
c2030_g1	0.92	71.608	6.14	5.91E-07	1.04E-04	up
c7763_g2	32.031	0.181	-6.84	5.96E-07	1.04E-04	down
c1460_g1	0.116	32.346	7.23	6.13E-07	1.06E-04	up
c8338_g2	0.82	68.583	6.22	6.39E-07	1.10E-04	up
c34461_g1	1.361	91.323	5.97	6.53E-07	1.11E-04	up
c1488_g1	0.866	65.26	6.08	6.60E-07	1.12E-04	up
c4118_g1	1.632	105.285	5.93	6.82E-07	1.14E-04	up
c35171_g1	236.635	2.792	-6.36	6.89E-07	1.14E-04	down
c11604_g1	0.17	21.9	6.35	7.22E-07	1.19E-04	up
c11606_g1	56.126	0	-9.14	7.29E-07	1.19E-04	down
c1751_g1	0.309	24.951	5.94	8.10E-07	1.31E-04	up
c167_g1	0.209	23.283	6.24	8.21E-07	1.32E-04	up
c8781_g1	29.177	0	-8.19	8.37E-07	1.33E-04	down
c35376_g1	13.15	795.121	5.91	8.43E-07	1.33E-04	up
c1142_g1	9.321	562.586	5.9	8.48E-07	1.33E-04	up
c22088_g1	12.407	750.881	5.91	8.56E-07	1.33E-04	up

seq_id	Fpkm of flower	Fpkm of leaf	log2FC (leaf/flower)	p-value	FDR	regulate
c19796_g1	4.061	261.093	5.97	9.90E-07	1.52E-04	up
c1163_g2	1.214	84.032	6	1.00E-06	1.53E-04	up
c28498_g1	7.341	475.826	6	1.13E-06	1.70E-04	up
c18543_g1	2.282	136.17	5.84	1.16E-06	1.74E-04	up
c4886_g1	0.804	58.926	6.03	1.17E-06	1.75E-04	up
c30122_g1	0.89	54.052	5.77	1.20E-06	1.76E-04	up
c1335_g2	629.721	8.351	-6.22	1.22E-06	1.76E-04	down
c584_g1	0	16.005	7.33	1.22E-06	1.76E-04	up
c8848_g1	0.054	13.497	6.46	1.23E-06	1.76E-04	up
c18933_g1	0.077	8.649	5.63	1.23E-06	1.76E-04	up
c21677_g1	1.307	86.048	5.94	1.24E-06	1.76E-04	up
c13940_g1	0.302	28.493	6.15	1.25E-06	1.76E-04	up
c28001_g1	0	11.028	6.8	1.26E-06	1.77E-04	up
c10726_g1	57.171	0.75	-6.07	1.28E-06	1.78E-04	down
c7045_g1	0.286	29.928	6.28	1.34E-06	1.85E-04	up
c3472_g1	0.441	44.201	6.36	1.35E-06	1.85E-04	up
c1335_g1	616.385	9.075	-6.07	1.37E-06	1.86E-04	down
c9820_g1	1.106	66.527	5.79	1.48E-06	1.96E-04	up
c8138_g1	0	6.606	6.07	1.48E-06	1.96E-04	up
c18230_g1	0	22.74	7.84	1.48E-06	1.96E-04	up
c27991_g1	0.557	50.251	6.26	1.49E-06	1.96E-04	up
c1600_g1	0	33.509	8.39	1.52E-06	2.00E-04	up
c21989_g1	2.723	149.848	5.73	1.59E-06	2.07E-04	up
c30514_g1	18.928	1049.388	5.79	1.60E-06	2.08E-04	up
c7404_g1	0.503	34.505	5.84	1.63E-06	2.09E-04	up
c26820_g1	2.653	148.348	5.75	1.78E-06	2.27E-04	up
c13016_g2	49.126	0.22	-7.27	1.80E-06	2.27E-04	down
c30114_g1	58.965	2960.708	5.65	1.81E-06	2.27E-04	up
c29986_g1	257.612	12719.656	5.63	1.83E-06	2.28E-04	up
c23569_g1	0.131	11.493	5.65	1.83E-06	2.28E-04	up
c21684_g1	83.919	4134.475	5.62	1.85E-06	2.29E-04	up
c11909_g1	0.425	37.271	6.15	2.01E-06	2.47E-04	up
c9237_g1	0.07	14.26	6.4	2.05E-06	2.49E-04	up
c26528_g1	0.681	42.934	5.78	2.07E-06	2.51E-04	up
c32894_g1	4.146	395.143	6.54	2.15E-06	2.59E-04	up
c8209_g1	0.565	36.741	5.79	2.17E-06	2.59E-04	up
c8731_g1	0.201	35.423	6.88	2.20E-06	2.61E-04	up
c21664_g1	7.031	338.777	5.57	2.22E-06	2.62E-04	up
c19779_g1	0.178	36.328	7.03	2.36E-06	2.75E-04	up
c23763_g1	22.54	0	-7.82	2.36E-06	2.75E-04	down
c7357_g1	0.588	35.746	5.7	2.40E-06	2.77E-04	up
c3095_g1	0.92	48.182	5.56	2.46E-06	2.83E-04	up

seq_id	Fpkm of flower	Fpkm of leaf	log2FC (leaf/flower)	p-value	FDR	regulate
c821_g1	18.588	0	-7.55	2.63E-06	3.01E-04	down
c26592_g1	3.264	154.218	5.52	2.67E-06	3.04E-04	up
c9413_g2	14.016	0	-7.14	2.73E-06	3.08E-04	down
c30767_g1	0.054	9.812	6.01	2.80E-06	3.14E-04	up
c34692_g1	44.013	0.569	-6.04	2.81E-06	3.14E-04	down
c8224_g1	0	16.238	7.35	2.83E-06	3.14E-04	up
c12322_g1	1.462	69.798	5.48	2.86E-06	3.15E-04	up
c34834_g1	814.073	14.841	-5.77	2.93E-06	3.18E-04	down
c485_g1	15.857	0	-7.32	2.93E-06	3.18E-04	down
c12666_g1	51.176	0	-9	2.93E-06	3.18E-04	down
c3841_g1	1.323	78.938	5.8	2.95E-06	3.18E-04	up
c3287_g1	0.07	14.001	6.37	3.03E-06	3.23E-04	up
c17779_g1	0	21.124	7.73	3.05E-06	3.23E-04	up
c12551_g1	50.812	0	-8.99	3.05E-06	3.23E-04	down
c26452_g1	8.431	375.415	5.46	3.22E-06	3.33E-04	up
c3017_g1	0.433	32.734	5.94	3.23E-06	3.33E-04	up
c30654_g1	17.443	772.471	5.46	3.24E-06	3.33E-04	up
c13665_g1	108.269	1.81	-5.83	3.24E-06	3.33E-04	down
c3688_g1	0	20.943	7.72	3.28E-06	3.33E-04	up
c12896_g2	0	13.264	7.06	3.28E-06	3.33E-04	up
c8973_g1	25.07	0	-7.98	3.28E-06	3.33E-04	down
c5633_g1	20.715	0	-7.7	3.28E-06	3.33E-04	down
c35761_g1	2.174	104.148	5.52	3.32E-06	3.35E-04	up
c5005_g1	0.627	39.185	5.76	3.39E-06	3.37E-04	up
c6933_g1	291.423	3.297	-6.42	3.40E-06	3.37E-04	down
c21761_g1	1.856	84.264	5.43	3.40E-06	3.37E-04	up
c8891_g2	0	16.173	7.35	3.41E-06	3.37E-04	up
c11909_g2	7.333	329.482	5.47	3.44E-06	3.38E-04	up
c7267_g1	0.286	21.292	5.79	3.51E-06	3.43E-04	up
c23158_g1	0.634	59.52	6.34	3.54E-06	3.43E-04	up
c3905_g1	0	17.634	7.47	3.55E-06	3.43E-04	up
c12744_g1	0.093	17.168	6.48	3.63E-06	3.49E-04	up
c13048_g1	23.113	0	-7.86	3.69E-06	3.53E-04	down
c12099_g1	0.433	25.752	5.6	3.70E-06	3.53E-04	up
c16038_g1	37.423	0.427	-6.15	3.75E-06	3.55E-04	down
c34667_g1	58.114	2431.347	5.38	3.77E-06	3.55E-04	up
c12533_g1	34.661	0	-8.44	3.99E-06	3.74E-04	down
c656_g1	0.093	16.328	6.41	4.16E-06	3.88E-04	up
c21737_g1	9.855	413.72	5.38	4.19E-06	3.89E-04	up
c8009_g1	1.539	61.731	5.24	4.33E-06	4.00E-04	up
c6496_g1	0.541	29.553	5.53	4.35E-06	4.00E-04	up
c35457_g1	62.578	2548.28	5.35	4.49E-06	4.09E-04	up

seq_id	Fpkm of flower	Fpkm of leaf	log2FC (leaf/flower)	p-value	FDR	regulate
c14399_g1	0	16.806	7.4	4.50E-06	4.09E-04	up
c15648_g1	0.093	15.94	6.38	4.52E-06	4.09E-04	up
c13854_g1	60.342	1.06	-5.7	4.58E-06	4.13E-04	down
c4446_g1	0	22.727	7.83	4.69E-06	4.19E-04	up
c34390_g1	0	7.136	6.18	4.69E-06	4.19E-04	up
c21780_g1	0.147	10.795	5.46	4.77E-06	4.23E-04	up
c11998_g2	0.224	19.172	5.89	4.78E-06	4.23E-04	up
c31701_g1	4.015	186.02	5.5	4.81E-06	4.24E-04	up
c25956_g1	447.364	17639.505	5.3	4.85E-06	4.25E-04	up
c3954_g1	1.648	69.462	5.31	4.91E-06	4.28E-04	up
c12228_g1	276.803	5.3	-5.68	4.97E-06	4.32E-04	down
c30051_g1	5.353	222.309	5.35	5.00E-06	4.32E-04	up
c8735_g1	0.379	25.921	5.76	5.04E-06	4.33E-04	up
c27341_g1	0	20.788	7.71	5.09E-06	4.36E-04	up
c2994_g1	1.029	48.57	5.43	5.14E-06	4.38E-04	up
c13568_g1	11.077	437.547	5.29	5.17E-06	4.39E-04	up
c8530_g1	0	22.417	7.81	5.31E-06	4.45E-04	up
c8111_g1	0	29.295	8.2	5.31E-06	4.45E-04	up
c5344_g1	14.48	0	-7.19	5.31E-06	4.45E-04	down
c2700_g1	0.294	24.317	5.95	5.52E-06	4.60E-04	up
c13967_g1	3.798	159.415	5.35	5.60E-06	4.65E-04	up
c33141_g1	0	46.825	8.87	5.79E-06	4.78E-04	up
c9078_g1	0.998	45.338	5.37	5.88E-06	4.84E-04	up
c15836_g1	1.454	72.733	5.55	5.93E-06	4.86E-04	up
c11658_g1	55.082	0.349	-6.94	5.97E-06	4.87E-04	down
c13605_g1	29.711	0.427	-5.82	6.06E-06	4.92E-04	down
c7346_g1	1.307	53.884	5.26	6.12E-06	4.95E-04	up
c31312_g1	30.809	0	-8.27	6.31E-06	5.08E-04	down
c22212_g1	28.202	1060.57	5.23	6.37E-06	5.10E-04	up
c13417_g1	25.24	0.362	-5.78	6.49E-06	5.18E-04	down
c32541_g1	4.015	160.216	5.28	6.55E-06	5.19E-04	up
c13638_g1	32.495	0.478	-5.82	6.58E-06	5.19E-04	down
c8392_g1	0	27.265	8.1	6.60E-06	5.19E-04	up
c14271_g1	52.197	0.931	-5.66	6.61E-06	5.19E-04	down
c6638_g1	0.704	44.886	5.81	6.65E-06	5.20E-04	up
c9103_g1	14.086	0.065	-6.43	6.86E-06	5.32E-04	down
c375_g1	0.487	33.302	5.83	6.86E-06	5.32E-04	up
c2990_g1	0.936	39.986	5.27	7.03E-06	5.43E-04	up
c8325_g1	18.634	685.686	5.19	7.17E-06	5.50E-04	up
c1282_g1	0.317	19.625	5.56	7.18E-06	5.50E-04	up
c10288_g1	11.912	0	-6.91	7.22E-06	5.51E-04	down
c10418_g2	2.63	96.287	5.14	7.62E-06	5.78E-04	up

seq_id	Fpkm of flower	Fpkm of leaf	log2FC (leaf/flower)	p-value	FDR	regulate
c12510_g1	1.276	81.317	5.89	7.64E-06	5.78E-04	up
c36408_g1	0.712	55.668	6.1	7.70E-06	5.80E-04	up
c11194_g1	1.601	64.717	5.25	7.82E-06	5.87E-04	up
c92_g1	6.838	0	-6.12	7.91E-06	5.91E-04	down
c26744_g1	0.232	35.099	6.73	7.95E-06	5.92E-04	up
c5540_g2	0.209	12.708	5.37	8.08E-06	5.99E-04	up
c21679_g1	8.578	307.091	5.15	8.11E-06	5.99E-04	up
c28661_g1	0	13.626	7.1	8.29E-06	6.10E-04	up
c7763_g1	63.962	1.215	-5.61	8.38E-06	6.11E-04	down
c17938_g1	2.429	150.973	5.9	8.41E-06	6.11E-04	up
c915_g1	26.532	0.362	-5.85	8.42E-06	6.11E-04	down
c21942_g2	4.989	178.509	5.13	8.44E-06	6.11E-04	up
c526_g1	0.116	8.636	5.34	8.47E-06	6.11E-04	up
c34443_g1	0	15.307	7.27	8.69E-06	6.19E-04	up
c13857_g1	0	8.313	6.39	8.69E-06	6.19E-04	up
c12799_g1	11.796	0	-6.89	8.69E-06	6.19E-04	down
c2277_g1	29.448	0.194	-6.65	8.74E-06	6.19E-04	down
c11495_g1	0.131	21.551	6.55	8.75E-06	6.19E-04	up
c262_g1	0.975	40.426	5.24	8.92E-06	6.29E-04	up
c6089_g2	133.857	3.129	-5.37	9.04E-06	6.35E-04	down
c6569_g1	0.278	18.927	5.65	9.13E-06	6.39E-04	up
c4616_g1	651.549	15.552	-5.38	9.30E-06	6.49E-04	down
c12717_g1	0.108	8.132	5.31	9.34E-06	6.49E-04	up
c17738_g1	82.604	2823.051	5.09	9.39E-06	6.49E-04	up
c3497_g2	0.178	11.209	5.35	9.42E-06	6.49E-04	up
c6846_g1	496.111	11.377	-5.43	9.45E-06	6.49E-04	down
c13277_g1	3384.171	82.972	-5.35	9.58E-06	6.56E-04	down
c22601_g1	1.593	64.317	5.25	9.95E-06	6.79E-04	up
c6838_g1	17.087	0.194	-5.87	1.01E-05	6.88E-04	down
c9193_g1	0.657	24.667	5.03	1.02E-05	6.89E-04	up
c5496_g1	0.774	33.91	5.28	1.03E-05	6.94E-04	up
c26114_g1	1.323	53.224	5.23	1.03E-05	6.95E-04	up
c30625_g1	3.365	113.779	5.04	1.04E-05	6.99E-04	up
c13329_g1	400.056	9.683	-5.35	1.05E-05	6.99E-04	down
c12955_g1	2834.981	90958.39	5	1.06E-05	7.01E-04	up
c1235_g1	26.887	0.44	-5.64	1.06E-05	7.01E-04	down
c13858_g1	1.276	47.989	5.13	1.06E-05	7.01E-04	up
c34121_g1	2.452	91.233	5.16	1.07E-05	7.02E-04	up
c21654_g1	8.555	286.302	5.05	1.08E-05	7.04E-04	up
c15167_g1	2.166	76.689	5.08	1.08E-05	7.04E-04	up
c12863_g1	9.205	0.103	-5.52	1.10E-05	7.16E-04	down
c23557_g1	185.203	3.917	-5.53	1.10E-05	7.16E-04	down

seq_id	Fpkm of flower	Fpkm of leaf	log2FC (leaf/flower)	p-value	FDR	regulate
c8530_g2	7.472	244.881	5.02	1.13E-05	7.29E-04	up
c1760_g1	0.286	15.992	5.38	1.15E-05	7.40E-04	up
c21942_g3	5.399	177.54	5.01	1.15E-05	7.40E-04	up
c8969_g1	0.263	15.1	5.39	1.16E-05	7.40E-04	up
c34779_g1	13.25	0	-7.06	1.16E-05	7.40E-04	down
c20355_g1	38.846	0	-8.61	1.16E-05	7.40E-04	down
c32364_g1	197.525	3.025	-5.98	1.20E-05	7.58E-04	down
c9226_g1	17.977	0.478	-4.97	1.20E-05	7.58E-04	down
c21708_g1	1205.303	30.988	-5.28	1.21E-05	7.59E-04	down
c6050_g1	18.216	0.259	-5.67	1.25E-05	7.79E-04	down
c9128_g2	0.24	12.475	5.21	1.25E-05	7.79E-04	up
c11491_g1	5.616	178.897	4.97	1.25E-05	7.80E-04	up
c14892_g1	0.186	12.411	5.45	1.27E-05	7.88E-04	up
c35086_g1	2.282	75.887	5	1.28E-05	7.93E-04	up
c35590_g1	48.314	1.086	-5.35	1.34E-05	8.24E-04	down
c30175_g1	0.294	12.488	5	1.34E-05	8.24E-04	up
c11941_g1	13.985	0	-7.14	1.36E-05	8.32E-04	down
c21901_g1	30.6	949.998	4.95	1.43E-05	8.61E-04	up
c168_g1	46.233	1.112	-5.26	1.43E-05	8.61E-04	down
c12888_g1	6.08	0	-5.95	1.43E-05	8.61E-04	down
c13189_g1	8.718	0	-6.46	1.43E-05	8.61E-04	down
c35745_g1	6.737	0	-6.1	1.43E-05	8.61E-04	down
c11837_g1	0.843	33.949	5.17	1.43E-05	8.61E-04	up
c34706_g1	1153.593	30.885	-5.22	1.44E-05	8.61E-04	down
c799_g1	0.294	16.832	5.43	1.44E-05	8.61E-04	up
c12851_g1	1.431	41.033	4.75	1.45E-05	8.64E-04	up
c6230_g2	0.657	26.929	5.16	1.46E-05	8.64E-04	up
c21775_g1	44.547	1367.493	4.94	1.49E-05	8.79E-04	up
c12325_g1	2.065	77.283	5.16	1.49E-05	8.80E-04	up
c15226_g1	105.09	1.913	-5.71	1.50E-05	8.82E-04	down
c35526_g1	21.133	0.323	-5.65	1.51E-05	8.82E-04	down
c13200_g1	8.516	0	-6.43	1.51E-05	8.82E-04	down
c28549_g1	0.248	33.16	6.58	1.52E-05	8.84E-04	up
c34848_g1	11.873	362.008	4.92	1.52E-05	8.84E-04	up
c7137_g1	159.639	2.586	-5.89	1.57E-05	9.07E-04	down
c15058_g1	0.627	32.604	5.49	1.57E-05	9.08E-04	up
c14134_g1	43.456	1521.788	5.13	1.59E-05	9.12E-04	up
c22920_g1	3.071	107.431	5.08	1.59E-05	9.12E-04	up
c36700_g1	39.828	0.711	-5.62	1.60E-05	9.13E-04	down
c13497_g1	30.747	0.388	-5.98	1.61E-05	9.18E-04	down
c11976_g2	15.254	281.105	4.19	1.62E-05	9.20E-04	up
c10605_g1	11.332	0.155	-5.49	1.63E-05	9.23E-04	down

seq_id	Fpkm of flower	Fpkm of leaf	log2FC (leaf/flower)	p-value	FDR	regulate
c30838_g1	10.705	324.97	4.91	1.66E-05	9.33E-04	up
c12862_g1	0.201	11.234	5.23	1.66E-05	9.33E-04	up
c8704_g1	0.263	10.51	4.87	1.67E-05	9.33E-04	up
c27354_g1	2.003	66.372	4.98	1.67E-05	9.33E-04	up
c12472_g1	9.909	223.641	4.48	1.68E-05	9.33E-04	up
c4976_g1	0	14.221	7.16	1.68E-05	9.33E-04	up
c18705_g1	0	4.098	5.39	1.68E-05	9.33E-04	up
c13033_g1	56.088	0.724	-6.09	1.73E-05	9.56E-04	down
c10307_g1	17.327	0.414	-5.08	1.74E-05	9.59E-04	down
c30075_g1	339.505	9997.172	4.88	1.74E-05	9.59E-04	up
c2820_g1	52.065	1.228	-5.3	1.76E-05	9.62E-04	down
c22460_g1	15.2	451.522	4.88	1.76E-05	9.62E-04	up
c11127_g1	9.476	0.142	-5.31	1.76E-05	9.62E-04	down
c3405_g1	6.343	0	-6.01	1.78E-05	9.66E-04	down
c3911_g1	248.291	6.8	-5.17	1.79E-05	9.71E-04	down
c13014_g1	1.129	37.827	4.95	1.80E-05	9.71E-04	up
c2662_g1	0.201	66.902	7.8	1.83E-05	9.86E-04	up
c3407_g1	906.501	25.455	-5.15	1.84E-05	9.86E-04	down
c18243_g1	72.796	1.81	-5.25	1.84E-05	9.86E-04	down
c35565_g1	5.794	174.476	4.89	1.84E-05	9.86E-04	up
c14145_g1	56.343	1.37	-5.26	1.86E-05	9.91E-04	down
c850_g1	62.338	1791.814	4.84	1.89E-05	1.01E-03	up
c19706_g1	43.742	0.944	-5.39	1.90E-05	1.01E-03	down
c12909_g1	172.618	4.292	-5.3	1.96E-05	1.03E-03	down
c21828_g1	1.199	39.12	4.92	1.96E-05	1.04E-03	up
c18115_g1	34.035	974.069	4.83	1.98E-05	1.04E-03	up
c11837_g2	0.193	10.291	5.15	1.98E-05	1.04E-03	up
c7767_g1	3.349	103.217	4.9	1.98E-05	1.04E-03	up
c18667_g1	78.984	2365.001	4.9	2.02E-05	1.05E-03	up
c11980_g1	30.345	0.246	-6.46	2.04E-05	1.06E-03	down
c8611_g1	45.042	1.189	-5.13	2.07E-05	1.07E-03	down
c4618_g1	5.422	155.407	4.82	2.08E-05	1.08E-03	up
c6229_g2	54.239	1.125	-5.47	2.11E-05	1.09E-03	down
c1306_g1	1.083	33.302	4.82	2.15E-05	1.10E-03	up
c9683_g1	1.617	54.646	4.99	2.16E-05	1.10E-03	up
c26599_g1	0.967	29.295	4.78	2.16E-05	1.10E-03	up
c10171_g1	3.543	101.045	4.8	2.20E-05	1.12E-03	up
c23125_g1	2.119	61.615	4.8	2.21E-05	1.12E-03	up
c13064_g3	0	11.092	6.81	2.23E-05	1.13E-03	up
c14598_g1	2.862	96.766	5.03	2.23E-05	1.13E-03	up
c11931_g1	1.307	34.582	4.62	2.25E-05	1.13E-03	up
c19851_g1	0.843	40.477	5.43	2.26E-05	1.13E-03	up

seq_id	Fpkm of flower	Fpkm of leaf	log2FC (leaf/flower)	p-value	FDR	regulate
c27440_g1	26.601	0.452	-5.6	2.26E-05	1.13E-03	down
c6984_g1	3.187	93.805	4.84	2.27E-05	1.14E-03	up
c30235_g1	2.212	63.825	4.79	2.29E-05	1.14E-03	up
c26950_g1	45.003	1.073	-5.26	2.30E-05	1.15E-03	down
c11998_g1	0.162	9.58	5.21	2.31E-05	1.15E-03	up
c8473_g1	19.678	0	-7.63	2.36E-05	1.17E-03	down
c9314_g2	16.221	0.22	-5.67	2.37E-05	1.17E-03	down
c26365_g1	22.718	628.221	4.78	2.38E-05	1.17E-03	up
c5886_g2	3.744	106.992	4.8	2.42E-05	1.19E-03	up
c9646_g1	56.065	1.538	-5.1	2.43E-05	1.19E-03	down
c34591_g1	1747.129	53.108	-5.04	2.43E-05	1.19E-03	down
c23072_g1	48.082	1.034	-5.41	2.44E-05	1.19E-03	down
c6060_g1	0.526	13.38	4.43	2.45E-05	1.19E-03	up
c1710_g1	1.044	37.09	5.02	2.47E-05	1.19E-03	up
c15157_g1	7.008	194.087	4.77	2.47E-05	1.19E-03	up
c9714_g1	3.264	89.901	4.74	2.49E-05	1.20E-03	up
c13253_g1	1635.333	50.329	-5.02	2.55E-05	1.23E-03	down
c4826_g1	1275.206	37.142	-5.1	2.56E-05	1.23E-03	down
c5887_g1	0.124	14.117	5.99	2.61E-05	1.25E-03	up
c34617_g1	2.986	81.162	4.72	2.63E-05	1.26E-03	up
c12917_g1	2.127	59.288	4.74	2.64E-05	1.26E-03	up
c3972_g1	72.131	2.056	-5.07	2.66E-05	1.26E-03	down
c19155_g1	4.672	0	-5.58	2.66E-05	1.26E-03	down
c20612_g1	3.636	130.973	5.13	2.67E-05	1.26E-03	up
c2042_g2	0.224	11.118	5.11	2.69E-05	1.27E-03	up
c13016_g1	27.483	0.388	-5.82	2.70E-05	1.27E-03	down
c29513_g1	0.936	54.556	5.72	2.72E-05	1.28E-03	up
c1842_g1	329.163	8722.36	4.73	2.81E-05	1.31E-03	up
c4910_g1	0	17.543	7.46	2.83E-05	1.32E-03	up
c34593_g1	17.458	456.654	4.7	2.85E-05	1.33E-03	up
c32562_g1	0.48	26.683	5.53	2.96E-05	1.37E-03	up
c5117_g1	18.51	0.388	-5.25	2.97E-05	1.37E-03	down
c26226_g1	18.526	484.953	4.7	2.98E-05	1.38E-03	up
c6921_g1	13.676	0	-7.11	3.01E-05	1.39E-03	down
c12380_g1	26.392	0.633	-5.18	3.01E-05	1.39E-03	down
c35967_g1	4.703	125.246	4.71	3.04E-05	1.39E-03	up
c3272_g1	1.106	32.449	4.75	3.07E-05	1.40E-03	up
c12327_g1	2.746	73.146	4.69	3.07E-05	1.40E-03	up
c7801_g1	3.164	86.087	4.72	3.11E-05	1.42E-03	up
c34393_g1	93.333	2382.596	4.67	3.16E-05	1.43E-03	up
c26494_g1	7.426	197.914	4.72	3.17E-05	1.43E-03	up
c23685_g1	2.498	85.286	5.04	3.17E-05	1.43E-03	up

seq_id	Fpkm of flower	Fpkm of leaf	log2FC (leaf/flower)	p-value	FDR	regulate
c13219_g1	20.854	0.362	-5.5	3.17E-05	1.43E-03	down
c11803_g1	34.429	0.582	-5.66	3.22E-05	1.45E-03	down
c8286_g1	14.163	0.31	-5.12	3.24E-05	1.45E-03	down
c3541_g1	6.211	158.923	4.66	3.26E-05	1.46E-03	up
c3143_g1	84.53	2.547	-5	3.36E-05	1.50E-03	down
c5923_g1	1.338	45.08	4.97	3.37E-05	1.50E-03	up
c35130_g1	0.232	25.261	6.26	3.37E-05	1.50E-03	up
c11948_g3	34.22	0.892	-5.11	3.38E-05	1.50E-03	down
c6796_g1	0.549	17.996	4.8	3.45E-05	1.53E-03	up
c13259_g1	64.759	1599.95	4.62	3.59E-05	1.59E-03	up
c22294_g1	2.406	62.649	4.65	3.63E-05	1.60E-03	up
c9668_g1	2.065	52.746	4.61	3.63E-05	1.60E-03	up
c22154_g1	261.774	8.649	-4.9	3.72E-05	1.63E-03	down
c35140_g1	0.936	31.131	4.91	3.73E-05	1.63E-03	up
c2188_g1	5.19	137.592	4.7	3.87E-05	1.68E-03	up
c7338_g1	24.273	0.22	-6.25	3.89E-05	1.68E-03	down
c15023_g1	0	16.302	7.36	3.89E-05	1.68E-03	up
c7715_g1	0	37.31	8.55	3.89E-05	1.68E-03	up
c10183_g2	21.125	0	-7.73	3.89E-05	1.68E-03	down
c2681_g1	7.967	0	-6.33	3.89E-05	1.68E-03	down
c6529_g1	4.015	102.687	4.64	3.90E-05	1.68E-03	up
c34888_g1	4.177	102.545	4.58	3.92E-05	1.68E-03	up
c30501_g1	74.838	1809.422	4.59	3.95E-05	1.69E-03	up
c26498_g1	1.207	39.107	4.91	3.96E-05	1.69E-03	up
c7069_g1	51.153	1.008	-5.53	3.97E-05	1.69E-03	down
c261_g1	7.372	181.896	4.61	4.00E-05	1.70E-03	up
c8534_g1	20.382	0.556	-4.96	4.01E-05	1.70E-03	down
c15293_g1	0.201	10.497	5.14	4.03E-05	1.71E-03	up
c27208_g1	3.682	96.727	4.68	4.10E-05	1.73E-03	up
c26156_g1	19.864	478.295	4.58	4.11E-05	1.73E-03	up
c966_g1	7.697	0	-6.28	4.16E-05	1.75E-03	down
c9336_g1	11.402	0.181	-5.36	4.19E-05	1.76E-03	down
c26541_g1	19.694	0.194	-6.07	4.32E-05	1.81E-03	down
c3998_g1	427.794	14.777	-4.85	4.32E-05	1.81E-03	down
c9935_g1	24.954	0.685	-5	4.39E-05	1.83E-03	down
c12669_g1	9.654	0	-6.61	4.45E-05	1.85E-03	down
c32227_g1	34.36	0.336	-6.3	4.47E-05	1.85E-03	down
c17696_g1	12.454	295.249	4.56	4.48E-05	1.85E-03	up
c22917_g1	1.965	55.719	4.76	4.51E-05	1.86E-03	up
c13450_g1	285.111	9.683	-4.87	4.56E-05	1.88E-03	down
c19564_g1	123.523	4.021	-4.91	4.60E-05	1.89E-03	down
c7329_g1	0.093	8.584	5.49	4.63E-05	1.89E-03	up

seq_id	Fpkm of flower	Fpkm of leaf	log2FC (leaf/flower)	p-value	FDR	regulate
c16460_g1	0.348	17.349	5.28	4.63E-05	1.89E-03	up
c15374_g1	9.391	225.127	4.57	4.63E-05	1.89E-03	up
c30384_g1	32.782	767.985	4.55	4.64E-05	1.89E-03	up
c1734_g1	0.325	10.187	4.6	4.71E-05	1.91E-03	up
c31573_g1	86.75	2.948	-4.83	4.73E-05	1.91E-03	down
c1378_g1	1.702	41.861	4.54	4.75E-05	1.91E-03	up
c25992_g1	26.671	623.554	4.54	4.76E-05	1.91E-03	up
c25356_g1	0	11.377	6.84	4.76E-05	1.91E-03	up
c26047_g1	0	4.305	5.46	4.76E-05	1.91E-03	up
c11218_g1	11.92	0	-6.91	4.76E-05	1.91E-03	down
c8383_g1	0.588	24.059	5.13	4.81E-05	1.93E-03	up
c30014_g1	169.447	3908.34	4.53	4.82E-05	1.93E-03	up
c9844_g1	0.487	15.035	4.69	4.92E-05	1.96E-03	up
c8495_g1	0.24	7.369	4.46	4.92E-05	1.96E-03	up
c20048_g1	8.702	210.118	4.58	4.96E-05	1.97E-03	up
c11459_g3	2569.069	93.883	-4.77	5.01E-05	1.99E-03	down
c18226_g1	0.07	7.123	5.41	5.09E-05	2.01E-03	up
c12269_g1	51.833	0	-9.02	5.10E-05	2.01E-03	down
c26832_g1	2.754	65.984	4.53	5.11E-05	2.01E-03	up
c6400_g1	1.199	36.004	4.8	5.13E-05	2.02E-03	up
c16011_g1	1.106	34.22	4.83	5.14E-05	2.02E-03	up
c3519_g1	42.443	1.383	-4.84	5.18E-05	2.03E-03	down
c12265_g1	3.69	84.058	4.47	5.25E-05	2.05E-03	up
c8441_g1	1.33	34.621	4.6	5.26E-05	2.05E-03	up
c85_g1	0.92	27.976	4.78	5.27E-05	2.05E-03	up
c7668_g3	0.248	24.098	6.12	5.34E-05	2.07E-03	up
c14993_g1	139.806	4.589	-4.9	5.36E-05	2.08E-03	down
c17843_g1	229.062	8.248	-4.78	5.40E-05	2.09E-03	down
c31734_g1	8.996	207.519	4.51	5.41E-05	2.09E-03	up
c3623_g1	27.328	0.763	-4.99	5.44E-05	2.09E-03	down
c1029_g1	0	6.49	6.04	5.48E-05	2.10E-03	up
c10328_g1	6.722	0	-6.09	5.48E-05	2.10E-03	down
c21740_g1	65.2	1446.832	4.47	5.71E-05	2.18E-03	up
c6193_g1	6.49	148.516	4.5	5.72E-05	2.18E-03	up
c10254_g1	22.37	0.478	-5.28	5.79E-05	2.21E-03	down
c8603_g1	14.032	0.194	-5.59	5.81E-05	2.21E-03	down
c16158_g1	8.834	0	-6.48	5.88E-05	2.23E-03	down
c26487_g1	0.224	10.51	5.03	5.89E-05	2.23E-03	up
c10965_g1	0.905	21.396	4.42	5.91E-05	2.24E-03	up
c13116_g1	22.339	0.233	-6.07	6.01E-05	2.27E-03	down
c7048_g1	210.675	7.77	-4.74	6.09E-05	2.29E-03	down
c9073_g1	1.261	30.109	4.47	6.18E-05	2.32E-03	up

seq_id	Fpkm of flower	Fpkm of leaf	log2FC (leaf/flower)	p-value	FDR	regulate
c18118_g1	9.468	209.381	4.45	6.20E-05	2.33E-03	up
c30698_g1	1.856	46.566	4.58	6.27E-05	2.35E-03	up
c22933_g1	44.06	1.125	-5.17	6.30E-05	2.35E-03	down
c19236_g1	0	4.564	5.54	6.33E-05	2.35E-03	up
c27532_g1	0	107.056	10.07	6.33E-05	2.35E-03	up
c9776_g1	0	6.386	6.02	6.33E-05	2.35E-03	up
c7217_g1	0	14.13	7.15	6.33E-05	2.35E-03	up
c13778_g1	14.349	316.709	4.45	6.35E-05	2.35E-03	up
c49_g1	47.448	1.5	-4.89	6.41E-05	2.37E-03	down
c21834_g1	13.072	281.778	4.42	6.44E-05	2.38E-03	up
c34433_g1	3.048	71.789	4.51	6.48E-05	2.38E-03	up
c3902_g1	234.863	5054.131	4.43	6.54E-05	2.40E-03	up
c28507_g1	1.996	68.454	5.03	6.60E-05	2.42E-03	up
c9496_g1	0.596	16.276	4.56	6.67E-05	2.44E-03	up
c26058_g1	1.276	35.888	4.71	6.75E-05	2.45E-03	up
c17871_g1	0.263	8.688	4.6	6.75E-05	2.45E-03	up
c7533_g1	0.286	9.696	4.67	6.75E-05	2.45E-03	up
c35339_g1	66.886	2.275	-4.82	6.76E-05	2.45E-03	down
c14222_g1	47.966	1.668	-4.76	6.77E-05	2.45E-03	down
c34615_g1	40.014	0.711	-5.63	6.80E-05	2.45E-03	down
c1230_g1	15.099	323.897	4.41	6.80E-05	2.45E-03	up
c16420_g1	16.847	0	-7.4	6.82E-05	2.45E-03	down
c3672_g1	12.384	266.057	4.41	6.84E-05	2.46E-03	up
c32163_g1	63.684	2.314	-4.72	6.90E-05	2.48E-03	down
c30142_g1	167.057	6.335	-4.7	6.99E-05	2.50E-03	down
c13015_g1	0.425	16.806	5.01	7.03E-05	2.51E-03	up
c13885_g1	93.658	3.297	-4.79	7.05E-05	2.51E-03	down
c17956_g1	8.362	178.07	4.4	7.08E-05	2.52E-03	up
c13424_g2	3.14	80.373	4.63	7.13E-05	2.53E-03	up
c26220_g1	5.183	117.231	4.47	7.20E-05	2.55E-03	up
c12399_g2	0.131	6.024	4.73	7.21E-05	2.55E-03	up
c6131_g2	0.325	11.674	4.79	7.26E-05	2.56E-03	up
c14609_g1	0.17	6.658	4.65	7.31E-05	2.56E-03	up
c22191_g1	8.578	185.128	4.42	7.32E-05	2.56E-03	up
c6717_g1	0.627	17.246	4.58	7.35E-05	2.56E-03	up
c27000_g1	0	7.744	6.29	7.35E-05	2.56E-03	up
c21904_g1	0	10.148	6.68	7.35E-05	2.56E-03	up
c3858_g1	0	6.05	5.94	7.35E-05	2.56E-03	up
c32994_g1	0	9.101	6.52	7.35E-05	2.56E-03	up
c14040_g1	4.363	94.852	4.41	7.41E-05	2.57E-03	up
c23135_g1	4.409	96.313	4.42	7.43E-05	2.57E-03	up
c22841_g1	73.043	2.624	-4.75	7.43E-05	2.57E-03	down

seq_id	Fpkm of flower	Fpkm of leaf	log2FC (leaf/flower)	p-value	FDR	regulate
c35061_g1	572.937	22.508	-4.66	7.44E-05	2.57E-03	down
c18536_g1	21.72	455.103	4.38	7.47E-05	2.58E-03	up
c6162_g1	0.255	9.05	4.69	7.50E-05	2.59E-03	up
c19699_g1	47.865	1.642	-4.78	7.55E-05	2.60E-03	down
c12260_g1	3471.795	138.394	-4.65	7.56E-05	2.60E-03	down
c1769_g1	0.193	8.701	4.91	7.59E-05	2.60E-03	up
c10807_g2	0.162	8.132	4.97	7.59E-05	2.60E-03	up
c22318_g1	88.382	3.245	-4.73	7.60E-05	2.60E-03	down
c21923_g1	5.817	123.19	4.38	7.82E-05	2.67E-03	up
c22724_g1	31.737	1.06	-4.78	7.87E-05	2.67E-03	down
c1298_g1	2.893	61.718	4.37	7.88E-05	2.67E-03	up
c31023_g1	237.485	9.218	-4.67	7.91E-05	2.67E-03	down
c37220_g1	165.92	6.412	-4.67	7.91E-05	2.67E-03	down
c34830_g1	13.707	285.436	4.37	7.93E-05	2.67E-03	up
c15965_g1	0	8.106	6.36	7.93E-05	2.67E-03	up
c36948_g1	16.112	0	-7.34	7.93E-05	2.67E-03	down
c30902_g1	11.092	0	-6.81	7.93E-05	2.67E-03	down
c13295_g1	577.702	11930.159	4.37	7.97E-05	2.67E-03	up
c5115_g1	23.236	0.427	-5.47	8.01E-05	2.68E-03	down
c502_g1	1.245	28.726	4.42	8.02E-05	2.68E-03	up
c9926_g1	22.734	468.121	4.36	8.03E-05	2.68E-03	up
c5428_g1	13.513	0.349	-4.92	8.06E-05	2.68E-03	down
c19470_g1	0.982	26.657	4.63	8.06E-05	2.68E-03	up
c14400_g1	317.73	12.217	-4.69	8.17E-05	2.71E-03	down
c6572_g1	8.919	0.233	-4.76	8.18E-05	2.71E-03	down
c14971_g1	1.648	35.991	4.37	8.25E-05	2.73E-03	up
c3157_g1	78.319	2.767	-4.77	8.30E-05	2.74E-03	down
c10182_g1	0.139	14.958	5.98	8.44E-05	2.77E-03	up
c24112_g1	0.209	8.998	4.88	8.44E-05	2.77E-03	up
c30152_g1	3.048	71.388	4.51	8.45E-05	2.77E-03	up
c7189_g1	9.081	190.429	4.38	8.49E-05	2.79E-03	up
c21990_g1	113.815	2297.853	4.33	8.53E-05	2.79E-03	up
c318_g1	2.614	53.147	4.29	8.57E-05	2.79E-03	up
c15789_g1	18.309	0	-7.52	8.58E-05	2.79E-03	down
c31090_g1	27.7	0	-8.12	8.58E-05	2.79E-03	down
c8834_g1	14.225	0	-7.16	8.58E-05	2.79E-03	down
c8606_g1	4.595	0.052	-4.95	8.60E-05	2.79E-03	down
c22226_g1	40.826	1.538	-4.64	8.61E-05	2.79E-03	down
c7256_g1	0.688	16.625	4.41	8.63E-05	2.79E-03	up
c34471_g1	1218.097	50.16	-4.6	8.74E-05	2.82E-03	down
c8625_g1	54.03	1.719	-4.9	8.82E-05	2.84E-03	down
c30028_g1	58.308	1170.29	4.32	8.82E-05	2.84E-03	up

seq_id	Fpkm of flower	Fpkm of leaf	log2FC (leaf/flower)	p-value	FDR	regulate
c26061_g1	358.433	13.962	-4.67	8.84E-05	2.84E-03	down
c21956_g1	162.609	3242.485	4.32	8.89E-05	2.85E-03	up
c14214_g1	3.79	77.025	4.31	8.90E-05	2.85E-03	up
c11538_g1	13.544	0.44	-4.66	8.92E-05	2.85E-03	down
c9429_g1	6.706	0.078	-5.26	8.97E-05	2.85E-03	down
c13574_g1	0.619	23.18	5.02	8.97E-05	2.85E-03	up
c29967_g1	27.328	543.258	4.31	8.97E-05	2.85E-03	up
c8646_g1	4.486	90.198	4.3	8.98E-05	2.85E-03	up
c34751_g1	39.31	789.652	4.32	9.14E-05	2.90E-03	up
c11958_g2	3.326	69.035	4.33	9.26E-05	2.92E-03	up
c13554_g1	0	10.097	6.67	9.28E-05	2.92E-03	up
c1137_g1	14.287	0	-7.17	9.28E-05	2.92E-03	down
c4354_g1	4.301	0	-5.46	9.28E-05	2.92E-03	down
c29145_g1	65.85	0	-9.37	9.28E-05	2.92E-03	down
c6321_g1	0.178	7.55	4.78	9.40E-05	2.95E-03	up
c33292_g1	3.063	73.689	4.54	9.48E-05	2.97E-03	up
c34453_g1	11.804	234.487	4.3	9.52E-05	2.98E-03	up
c26031_g1	85.164	1669.748	4.29	9.61E-05	3.00E-03	up
c24087_g1	64.844	2.392	-4.7	9.67E-05	3.01E-03	down
c9447_g1	0.34	8.623	4.31	9.68E-05	3.01E-03	up
c15007_g1	4.432	97.709	4.43	9.72E-05	3.02E-03	up
c26381_g1	3.403	69.371	4.31	9.73E-05	3.02E-03	up
c529_g1	13.745	0.478	-4.58	9.74E-05	3.02E-03	down
c13432_g1	1065.474	44.976	-4.56	9.75E-05	3.02E-03	down
c7709_g1	41.94	1.629	-4.6	9.80E-05	3.02E-03	down
c3561_g1	28.566	0.866	-4.89	9.92E-05	3.06E-03	down
c12464_g1	0.998	20.31	4.22	9.99E-05	3.06E-03	up
c9002_g1	1.872	42.753	4.44	1.00E-04	3.06E-03	up
c14111_g1	0	5.43	5.79	1.01E-04	3.06E-03	up
c8842_g1	0	32.346	8.34	1.01E-04	3.06E-03	up
c7362_g1	0	9.14	6.53	1.01E-04	3.06E-03	up
c9843_g1	0	14.518	7.19	1.01E-04	3.06E-03	up
c8715_g1	0	9.605	6.6	1.01E-04	3.06E-03	up
c12616_g1	9.398	0	-6.57	1.01E-04	3.06E-03	down
c12582_g1	9.731	0	-6.62	1.01E-04	3.06E-03	down
c11149_g1	66.383	2.03	-4.96	1.01E-04	3.06E-03	down
c11188_g1	6.931	0.078	-5.3	1.02E-04	3.08E-03	down
c32838_g1	1.4	32.514	4.44	1.03E-04	3.13E-03	up
c35246_g1	24.606	478.632	4.28	1.05E-04	3.17E-03	up
c25986_g1	48.554	924.93	4.25	1.07E-04	3.21E-03	up
c35695_g1	9.205	177.772	4.26	1.07E-04	3.21E-03	up
c13086_g2	0.982	22.34	4.37	1.07E-04	3.21E-03	up

seq_id	Fpkm of flower	Fpkm of leaf	log2FC (leaf/flower)	p-value	FDR	regulate
c14929_g1	35.566	1.021	-4.99	1.08E-04	3.24E-03	down
c36446_g1	0	16.548	7.38	1.09E-04	3.25E-03	up
c6797_g1	0	17.802	7.48	1.09E-04	3.25E-03	up
c1738_g1	0	8.222	6.38	1.09E-04	3.25E-03	up
c7089_g1	10.234	0	-6.69	1.09E-04	3.25E-03	down
c8840_g1	6.072	0	-5.95	1.09E-04	3.25E-03	down
c21918_g1	2.212	44.601	4.27	1.10E-04	3.26E-03	up
c30064_g1	6.977	134.063	4.24	1.10E-04	3.26E-03	up
c31359_g1	67.868	2.573	-4.67	1.10E-04	3.26E-03	down
c8627_g2	40.996	1.487	-4.69	1.10E-04	3.26E-03	down
c9841_g1	1.524	31.583	4.29	1.11E-04	3.26E-03	up
c19691_g1	30.461	0.892	-4.95	1.11E-04	3.26E-03	down
c33407_g1	0.433	35.81	6.07	1.11E-04	3.27E-03	up
c23197_g1	62.168	2.456	-4.61	1.12E-04	3.29E-03	down
c5962_g1	1.067	22.637	4.28	1.12E-04	3.29E-03	up
c13309_g1	6.993	137.308	4.28	1.12E-04	3.29E-03	up
c7717_g1	8.942	0.194	-4.94	1.13E-04	3.30E-03	down
c4206_g1	0.379	12.178	4.68	1.13E-04	3.30E-03	up
c2878_g1	0.495	12.385	4.39	1.13E-04	3.30E-03	up
c6574_g1	1.006	25.52	4.53	1.15E-04	3.36E-03	up
c23470_g1	54.719	1.629	-4.99	1.17E-04	3.39E-03	down
c14064_g1	152.607	6.554	-4.52	1.17E-04	3.40E-03	down
c27157_g1	0.108	8.377	5.35	1.18E-04	3.41E-03	up
c22739_g1	12.191	241.339	4.3	1.18E-04	3.41E-03	up
c34587_g1	36.1	1.332	-4.66	1.19E-04	3.42E-03	down
c18188_g1	1.942	41.744	4.36	1.19E-04	3.42E-03	up
c14639_g1	39.349	1.603	-4.53	1.19E-04	3.42E-03	down
c18166_g1	9.522	178.289	4.21	1.20E-04	3.45E-03	up
c10465_g1	25.039	0.724	-4.93	1.21E-04	3.46E-03	down
c26347_g1	6.49	126.06	4.26	1.21E-04	3.46E-03	up
c6888_g1	8.988	0.233	-4.77	1.21E-04	3.46E-03	down
c11927_g1	15.068	0.194	-5.69	1.21E-04	3.46E-03	down
c12107_g4	0.193	6.192	4.42	1.22E-04	3.47E-03	up
c11820_g1	18.278	0.621	-4.67	1.22E-04	3.47E-03	down
c13153_g1	2.738	55.707	4.3	1.22E-04	3.48E-03	up
c8165_g1	2.027	41.098	4.28	1.23E-04	3.48E-03	up
c8374_g1	2.599	50.6	4.23	1.23E-04	3.49E-03	up
c11244_g1	0.147	11.687	5.58	1.25E-04	3.53E-03	up
c24210_g1	0.302	9.644	4.6	1.26E-04	3.56E-03	up
c18070_g1	21.194	393.1	4.21	1.26E-04	3.56E-03	up
c1562_g1	1.253	26.464	4.3	1.29E-04	3.63E-03	up
c8829_g1	3.342	70.548	4.36	1.29E-04	3.63E-03	up

seq_id	Fpkm of flower	Fpkm of leaf	log2FC (leaf/flower)	p-value	FDR	regulate
c26912_g1	3.396	0	-5.13	1.29E-04	3.63E-03	down
c34739_g1	54.68	996.215	4.18	1.31E-04	3.68E-03	up
c9183_g1	3.171	45.429	3.8	1.32E-04	3.69E-03	up
c14372_g1	0.147	5.572	4.52	1.32E-04	3.69E-03	up
c34679_g1	0.139	5.365	4.52	1.32E-04	3.69E-03	up
c35727_g1	0.139	5.507	4.55	1.32E-04	3.69E-03	up
c9162_g1	0.58	27.64	5.35	1.33E-04	3.69E-03	up
c22617_g1	31.683	1.345	-4.46	1.33E-04	3.69E-03	down
c6051_g1	103.21	4.564	-4.47	1.33E-04	3.69E-03	down
c4781_g1	57.612	1042.536	4.18	1.33E-04	3.69E-03	up
c12010_g1	14.604	0.556	-4.49	1.34E-04	3.71E-03	down
c22188_g1	3.017	56.392	4.18	1.35E-04	3.73E-03	up
c26272_g1	2.212	43.179	4.23	1.35E-04	3.73E-03	up
c200_g1	2.351	44.369	4.18	1.35E-04	3.73E-03	up
c4916_g1	43.611	1.784	-4.54	1.37E-04	3.77E-03	down
c8668_g1	11.533	0.362	-4.65	1.37E-04	3.77E-03	down
c22259_g1	61.943	1151.402	4.21	1.38E-04	3.79E-03	up
c4900_g1	29.154	1.164	-4.53	1.38E-04	3.79E-03	down
c12996_g1	3.929	52.888	3.72	1.39E-04	3.79E-03	up
c15443_g1	0.472	15.772	4.79	1.39E-04	3.80E-03	up
c5729_g2	30.229	545.417	4.17	1.40E-04	3.80E-03	up
c6339_g1	3.102	58.732	4.2	1.40E-04	3.80E-03	up
c22506_g1	0.093	6.929	5.19	1.41E-04	3.80E-03	up
c30555_g1	0.046	3.594	4.66	1.41E-04	3.80E-03	up
c8290_g1	3.164	63.903	4.29	1.41E-04	3.80E-03	up
c13696_g1	49.466	2.056	-4.52	1.41E-04	3.80E-03	down
c25144_g1	0	6.735	6.09	1.41E-04	3.80E-03	up
c23928_g1	0	7.615	6.27	1.41E-04	3.80E-03	up
c1216_g1	8.176	0	-6.37	1.41E-04	3.80E-03	down
c12908_g1	6.482	0	-6.04	1.41E-04	3.80E-03	down
c7647_g1	8.996	0.297	-4.52	1.42E-04	3.83E-03	down
c13374_g1	27.344	486.13	4.15	1.44E-04	3.87E-03	up
c7836_g1	17.814	0.246	-5.69	1.46E-04	3.90E-03	down
c26337_g1	48.685	2.146	-4.44	1.47E-04	3.92E-03	down
c32327_g1	13.807	282.812	4.35	1.47E-04	3.92E-03	up
c6524_g1	1.238	30.536	4.52	1.49E-04	3.98E-03	up
c8599_g1	25.882	456.447	4.14	1.50E-04	3.98E-03	up
c10262_g3	3.891	79.481	4.32	1.51E-04	4.00E-03	up
c26099_g1	25.186	443.584	4.13	1.51E-04	4.00E-03	up
c9384_g1	19.632	0.556	-4.91	1.51E-04	4.00E-03	down
c22773_g1	0.418	12.579	4.61	1.52E-04	4.03E-03	up
c13283_g1	14.372	252.987	4.13	1.54E-04	4.06E-03	up

seq_id	Fpkm of flower	Fpkm of leaf	log2FC (leaf/flower)	p-value	FDR	regulate
c28663_g1	0	6.761	6.1	1.54E-04	4.06E-03	up
c336_g1	6.675	0	-6.08	1.54E-04	4.06E-03	down
c5904_g2	10.644	0	-6.75	1.54E-04	4.06E-03	down
c21881_g1	8.586	0	-6.44	1.54E-04	4.06E-03	down
c11801_g1	19.075	0.75	-4.5	1.56E-04	4.09E-03	down
c34513_g1	5.368	99.455	4.19	1.56E-04	4.10E-03	up
c31968_g1	0.093	6.735	5.15	1.59E-04	4.15E-03	up
c15113_g1	7.65	146.357	4.24	1.59E-04	4.15E-03	up
c17787_g1	65.563	3.012	-4.4	1.59E-04	4.15E-03	down
c30316_g1	24.861	0.983	-4.53	1.60E-04	4.17E-03	down
c3074_g1	6.103	0.078	-5.12	1.60E-04	4.17E-03	down
c34794_g1	67.072	1160.788	4.11	1.61E-04	4.18E-03	up
c10228_g1	2.29	48.441	4.34	1.62E-04	4.21E-03	up
c36780_g1	2.344	45.791	4.23	1.62E-04	4.21E-03	up
c16040_g1	1.153	31.596	4.66	1.62E-04	4.21E-03	up
c3061_g1	0.789	16.47	4.22	1.64E-04	4.25E-03	up
c34614_g1	3.968	70.781	4.12	1.65E-04	4.26E-03	up
c4353_g1	6.095	105.298	4.09	1.66E-04	4.28E-03	up
c6424_g1	2.135	48.764	4.45	1.67E-04	4.30E-03	up
c31481_g1	2.243	40.839	4.13	1.68E-04	4.30E-03	up
c37250_g1	0	5.21	5.73	1.69E-04	4.30E-03	up
c6163_g1	0	8.455	6.42	1.69E-04	4.30E-03	up
c30351_g1	0	15.035	7.24	1.69E-04	4.30E-03	up
c7217_g2	0	5.611	5.84	1.69E-04	4.30E-03	up
c13154_g2	0	15.423	7.28	1.69E-04	4.30E-03	up
c2883_g1	0	8.804	6.48	1.69E-04	4.30E-03	up
c18780_g1	4.881	0	-5.64	1.69E-04	4.30E-03	down
c9963_g1	0.077	5.856	5.07	1.69E-04	4.30E-03	up
c1763_g1	5.059	90.366	4.13	1.69E-04	4.30E-03	up
c13190_g2	1.044	15.824	3.8	1.70E-04	4.31E-03	up
c6834_g1	7.503	131.658	4.12	1.71E-04	4.34E-03	up
c12479_g2	25.263	0.814	-4.79	1.72E-04	4.36E-03	down
c31767_g1	2.823	51.776	4.15	1.76E-04	4.43E-03	up
c13647_g1	10.195	174.928	4.09	1.76E-04	4.43E-03	up
c30656_g1	20.514	0.284	-5.75	1.76E-04	4.43E-03	down
c1213_g1	12.322	0.168	-5.53	1.76E-04	4.43E-03	down
c14449_g1	1.261	22.74	4.07	1.76E-04	4.43E-03	up
c10643_g1	0.294	15.863	5.34	1.77E-04	4.45E-03	up
c11217_g1	0.727	14.79	4.17	1.79E-04	4.48E-03	up
c30365_g1	15.679	271.035	4.1	1.80E-04	4.52E-03	up
c10624_g1	3.226	52.074	3.97	1.84E-04	4.59E-03	up
c7290_g1	3.14	54.414	4.07	1.84E-04	4.59E-03	up

seq_id	Fpkm of flower	Fpkm of leaf	log2FC (leaf/flower)	p-value	FDR	regulate
c8192_g1	1.578	28.144	4.07	1.84E-04	4.59E-03	up
c27906_g1	0.843	24.55	4.71	1.85E-04	4.59E-03	up
c2588_g1	5.136	99.868	4.25	1.85E-04	4.59E-03	up
c9469_g1	7.898	0	-6.32	1.85E-04	4.59E-03	down
c6593_g1	17.52	0	-7.46	1.85E-04	4.59E-03	down
c1688_g1	44.121	1.319	-4.96	1.86E-04	4.60E-03	down
c1492_g1	2.073	42.029	4.28	1.87E-04	4.63E-03	up
c2855_g1	18.92	0.621	-4.72	1.88E-04	4.64E-03	down
c32332_g1	19.176	0.633	-4.72	1.88E-04	4.64E-03	down
c6995_g1	73.832	2.883	-4.63	1.89E-04	4.67E-03	down
c8417_g1	25.364	1.047	-4.47	1.90E-04	4.67E-03	down
c9125_g1	10.62	149.602	3.8	1.90E-04	4.67E-03	up
c23853_g1	1.114	22.495	4.22	1.91E-04	4.68E-03	up
c9938_g1	0.665	13.161	4.12	1.91E-04	4.68E-03	up
c7668_g2	6.877	117.903	4.08	1.91E-04	4.68E-03	up
c14442_g1	0.077	5.326	4.94	1.92E-04	4.68E-03	up
c23329_g1	0.085	5.74	4.98	1.92E-04	4.68E-03	up
c36881_g1	0.232	16.457	5.64	1.92E-04	4.68E-03	up
c30132_g1	46.233	772.859	4.06	1.93E-04	4.69E-03	up
c9342_g2	19.384	0.44	-5.17	1.93E-04	4.69E-03	down
c10092_g2	47.54	1.694	-4.73	1.94E-04	4.71E-03	down
c8081_g1	11.587	193.57	4.05	1.96E-04	4.76E-03	up
c1706_g1	253.451	12.876	-4.29	1.97E-04	4.77E-03	down
c7885_g1	17.304	0.401	-5.12	2.01E-04	4.85E-03	down
c9690_g1	15.192	0.349	-5.09	2.01E-04	4.85E-03	down
c6652_g1	6.111	103.579	4.06	2.01E-04	4.85E-03	up
c6183_g1	1.307	24.46	4.13	2.01E-04	4.85E-03	up
c22626_g1	9.646	160.539	4.04	2.01E-04	4.85E-03	up
c8180_g1	1.16	21.913	4.13	2.03E-04	4.87E-03	up
c12778_g3	43.549	2.043	-4.35	2.03E-04	4.87E-03	down
c12420_g1	3.79	0	-5.28	2.03E-04	4.87E-03	down
c27806_g1	15.571	0.452	-4.83	2.04E-04	4.87E-03	down
c5592_g1	10.999	0.323	-4.71	2.04E-04	4.87E-03	down
c13595_g1	61.123	2.805	-4.4	2.04E-04	4.88E-03	down
c4587_g1	5.724	96.753	4.06	2.06E-04	4.92E-03	up
c22193_g1	11.719	192.924	4.03	2.09E-04	4.98E-03	up
c12426_g1	24.227	387.748	3.99	2.11E-04	5.02E-03	up
c17903_g1	7.488	122.738	4.02	2.14E-04	5.10E-03	up
c31314_g1	0.309	9.231	4.51	2.15E-04	5.10E-03	up
c10643_g2	1.098	20.181	4.08	2.16E-04	5.11E-03	up
c13242_g1	72.015	3.4	-4.36	2.16E-04	5.11E-03	down
c3999_g1	73.6	1185.364	4.01	2.17E-04	5.12E-03	up

seq_id	Fpkm of flower	Fpkm of leaf	log2FC (leaf/flower)	p-value	FDR	regulate
c8880_g1	0.681	13.484	4.12	2.18E-04	5.14E-03	up
c8106_g1	35.628	1.616	-4.38	2.18E-04	5.14E-03	down
c12096_g4	0.108	7.64	5.22	2.18E-04	5.14E-03	up
c3251_g1	0.464	16.082	4.84	2.18E-04	5.14E-03	up
c22282_g1	23.499	380.03	4.01	2.19E-04	5.15E-03	up
c17977_g1	32.124	1.448	-4.38	2.20E-04	5.17E-03	down
c35399_g1	21.983	0.995	-4.33	2.22E-04	5.20E-03	down
c655_g1	0.681	12.398	4	2.22E-04	5.20E-03	up
c10177_g1	22.386	0.918	-4.47	2.24E-04	5.21E-03	down
c9951_g1	0	12.812	7.01	2.24E-04	5.21E-03	up
c7332_g1	0	36.547	8.52	2.24E-04	5.21E-03	up
c31710_g1	0	8.028	6.34	2.24E-04	5.21E-03	up
c396_g1	5.268	0	-5.75	2.24E-04	5.21E-03	down
c30184_g1	45.978	2.249	-4.29	2.25E-04	5.22E-03	down
c22695_g1	21.264	343.108	4.01	2.25E-04	5.23E-03	up
c12329_g3	0.743	22.159	4.72	2.26E-04	5.23E-03	up
c11942_g1	0.387	12.488	4.69	2.26E-04	5.23E-03	up
c26420_g1	123.763	6.231	-4.29	2.26E-04	5.23E-03	down
c10243_g2	2.854	55.396	4.23	2.26E-04	5.23E-03	up
c783_g1	7.782	0.181	-4.81	2.27E-04	5.23E-03	down
c13161_g1	21.519	1.021	-4.27	2.27E-04	5.23E-03	down
c36423_g1	22.455	1.021	-4.33	2.27E-04	5.23E-03	down
c13154_g1	32.124	397.185	3.62	2.28E-04	5.24E-03	up
c34429_g1	147.958	7.382	-4.31	2.30E-04	5.28E-03	down
c17847_g1	24.188	384.581	3.99	2.32E-04	5.31E-03	up
c8547_g1	1.292	23.594	4.09	2.32E-04	5.31E-03	up
c9358_g1	12.717	0.556	-4.29	2.37E-04	5.41E-03	down
c15386_g1	16.298	0.388	-5.07	2.37E-04	5.41E-03	down
c9165_g1	1.199	19.573	3.92	2.37E-04	5.41E-03	up
c27292_g1	4.1	66.812	3.99	2.37E-04	5.41E-03	up
c26343_g1	40.532	640.864	3.98	2.38E-04	5.42E-03	up
c17912_g1	18.557	296.515	3.99	2.39E-04	5.42E-03	up
c26290_g1	1.942	34.854	4.1	2.39E-04	5.44E-03	up
c5567_g1	29.378	1.319	-4.38	2.41E-04	5.47E-03	down
c12098_g1	11.649	0.504	-4.28	2.43E-04	5.50E-03	down
c17178_g1	0	14.738	7.21	2.47E-04	5.57E-03	up
c12079_g1	8.671	0	-6.45	2.47E-04	5.57E-03	down
c12821_g1	20.266	0	-7.67	2.47E-04	5.57E-03	down
c30583_g1	6.583	0	-6.06	2.47E-04	5.57E-03	down
c31564_g1	6.32	0	-6	2.47E-04	5.57E-03	down
c12416_g1	0.828	13.833	3.91	2.48E-04	5.57E-03	up
c13502_g1	54.092	2.689	-4.28	2.48E-04	5.57E-03	down

seq_id	Fpkm of flower	Fpkm of leaf	log2FC (leaf/flower)	p-value	FDR	regulate
c11569_g2	6.119	100.683	4.02	2.48E-04	5.57E-03	up
c3022_g1	91.677	4.085	-4.45	2.49E-04	5.59E-03	down
c9904_g1	0.093	13.303	6.12	2.50E-04	5.59E-03	up
c9392_g1	13.691	0.646	-4.21	2.50E-04	5.59E-03	down
c8820_g1	43.379	1.797	-4.52	2.50E-04	5.59E-03	down
c14666_g1	0.495	14.505	4.62	2.51E-04	5.59E-03	up
c6379_g1	23.67	1.047	-4.37	2.51E-04	5.59E-03	down
c21788_g1	16.43	258.119	3.97	2.52E-04	5.60E-03	up
c944_g1	10.319	165.012	3.99	2.52E-04	5.60E-03	up
c13265_g1	38.428	597.31	3.95	2.55E-04	5.67E-03	up
c23775_g1	0.719	19.108	4.55	2.58E-04	5.72E-03	up
c10090_g1	2.514	39.598	3.92	2.61E-04	5.77E-03	up
c1121_g1	27.537	0.866	-4.84	2.61E-04	5.77E-03	down
c12794_g1	1.3	25.442	4.19	2.61E-04	5.77E-03	up
c26294_g1	822.551	44.201	-4.21	2.61E-04	5.77E-03	down
c4922_g1	79.897	4.137	-4.24	2.64E-04	5.82E-03	down
c13086_g3	0.248	6.968	4.34	2.64E-04	5.82E-03	up
c14300_g1	105.26	5.546	-4.22	2.66E-04	5.85E-03	down
c13477_g1	25.116	384.736	3.93	2.67E-04	5.85E-03	up
c6451_g1	467.058	25.093	-4.21	2.67E-04	5.85E-03	down
c4345_g1	5.221	86.876	4.03	2.67E-04	5.86E-03	up
c17664_g1	41.43	633.444	3.93	2.69E-04	5.88E-03	up
c12229_g1	0.766	15.371	4.16	2.70E-04	5.90E-03	up
c34910_g1	1.276	22.249	4.02	2.71E-04	5.90E-03	up
c8803_g1	60.938	3.038	-4.28	2.71E-04	5.90E-03	down
c32971_g1	46.821	1.487	-4.89	2.71E-04	5.90E-03	down
c3238_g1	62.616	957.573	3.93	2.72E-04	5.91E-03	up
c1728_g1	0	8.998	6.51	2.73E-04	5.94E-03	up
c26371_g1	0	6.167	5.97	2.73E-04	5.94E-03	up
c17699_g1	68.201	1036.149	3.92	2.75E-04	5.96E-03	up
c1538_g1	80.175	4.176	-4.23	2.75E-04	5.96E-03	down
c156_g1	9.77	150.481	3.93	2.76E-04	5.97E-03	up
c22910_g1	35.582	1.616	-4.38	2.78E-04	6.00E-03	down
c28435_g1	0.371	10.368	4.47	2.79E-04	6.02E-03	up
c18044_g1	52.104	1.668	-4.88	2.81E-04	6.05E-03	down
c4526_g1	0.425	10.342	4.31	2.81E-04	6.05E-03	up
c13033_g2	9.437	0.246	-4.78	2.81E-04	6.05E-03	down
c9593_g1	11.054	0.259	-4.96	2.81E-04	6.05E-03	down
c8513_g1	11.611	0.414	-4.51	2.84E-04	6.11E-03	down
c10021_g1	14.24	0.672	-4.22	2.87E-04	6.14E-03	down
c37223_g1	5.322	84.95	3.97	2.88E-04	6.16E-03	up
c9397_g1	1.818	28.597	3.9	2.91E-04	6.22E-03	up

seq_id	Fpkm of flower	Fpkm of leaf	log2FC (leaf/flower)	p-value	FDR	regulate
c22127_g1	458.371	25.093	-4.19	2.93E-04	6.26E-03	down
c13222_g3	22.455	0.569	-5.08	2.94E-04	6.27E-03	down
c31688_g1	18.688	0.297	-5.56	2.94E-04	6.27E-03	down
c27808_g1	0.356	9.89	4.45	2.94E-04	6.27E-03	up
c2287_g1	36.734	1.836	-4.25	2.96E-04	6.29E-03	down
c35270_g1	2.39	37.53	3.92	2.96E-04	6.29E-03	up
c2628_g1	0	14.738	7.21	3.03E-04	6.36E-03	up
c28268_g1	0	5.688	5.85	3.03E-04	6.36E-03	up
c14499_g1	0	8.675	6.46	3.03E-04	6.36E-03	up
c2487_g1	0	11.196	6.82	3.03E-04	6.36E-03	up
c36967_g1	0	4.408	5.49	3.03E-04	6.36E-03	up
c4596_g2	0	13.626	7.1	3.03E-04	6.36E-03	up
c27865_g1	7.843	0	-6.31	3.03E-04	6.36E-03	down
c10255_g1	7.72	0	-6.29	3.03E-04	6.36E-03	down
c10399_g1	3.605	0	-5.21	3.03E-04	6.36E-03	down
c6146_g1	3.55	0	-5.19	3.03E-04	6.36E-03	down
c9955_g1	10.976	163.021	3.88	3.05E-04	6.40E-03	up
c2378_g1	1.307	23.451	4.07	3.05E-04	6.40E-03	up
c13681_g1	10.141	152.834	3.9	3.07E-04	6.42E-03	up
c3127_g1	1.57	30.846	4.21	3.07E-04	6.42E-03	up
c1773_g1	2.127	32.475	3.87	3.08E-04	6.42E-03	up
c11566_g1	0.209	13.406	5.45	3.08E-04	6.42E-03	up
c35332_g1	0.766	24.692	4.84	3.08E-04	6.42E-03	up
c15466_g1	17.59	0.814	-4.27	3.08E-04	6.42E-03	down
c14479_g1	0.302	7.007	4.14	3.09E-04	6.42E-03	up
c22050_g1	10.218	154.58	3.91	3.09E-04	6.42E-03	up
c1545_g1	0.209	5.675	4.22	3.11E-04	6.45E-03	up
c8214_g1	150.001	7.977	-4.22	3.13E-04	6.49E-03	down
c2243_g1	32.774	1.706	-4.19	3.16E-04	6.54E-03	down
c10409_g1	14.403	0.582	-4.41	3.17E-04	6.56E-03	down
c122_g1	5.716	84.833	3.87	3.18E-04	6.57E-03	up
c8067_g1	0.859	14.492	3.93	3.19E-04	6.57E-03	up
c34623_g1	18.309	0.685	-4.55	3.19E-04	6.57E-03	down
c30574_g1	0.835	16.431	4.14	3.19E-04	6.57E-03	up
c12445_g1	1048.573	15300.735	3.87	3.20E-04	6.58E-03	up
c35812_g1	48.979	2.573	-4.2	3.21E-04	6.59E-03	down
c5540_g1	1.292	22.818	4.04	3.22E-04	6.59E-03	up
c30206_g1	314.443	4869.003	3.95	3.22E-04	6.59E-03	up
c5699_g1	0.394	9.748	4.32	3.22E-04	6.59E-03	up
c18823_g1	11.386	173.377	3.92	3.23E-04	6.62E-03	up
c4926_g1	0.534	10.355	4.04	3.24E-04	6.62E-03	up
c191_g1	13.297	0.44	-4.63	3.27E-04	6.68E-03	down

seq_id	Fpkm of flower	Fpkm of leaf	log2FC (leaf/flower)	p-value	FDR	regulate
c34936_g1	7.874	0.129	-5.12	3.28E-04	6.69E-03	down
c13523_g1	4.432	66.049	3.87	3.29E-04	6.69E-03	up
c30638_g1	3.543	54.155	3.9	3.29E-04	6.69E-03	up
c31106_g1	147.224	7.692	-4.24	3.30E-04	6.69E-03	down
c10593_g1	15.354	1.306	-3.46	3.30E-04	6.69E-03	down
c13284_g1	74.73	1083.168	3.86	3.31E-04	6.69E-03	up
c10036_g2	1.392	44.757	4.91	3.31E-04	6.69E-03	up
c13893_g1	0.193	6.063	4.39	3.31E-04	6.69E-03	up
c7761_g2	2.498	37.064	3.84	3.31E-04	6.69E-03	up
c17863_g1	4.045	60.257	3.86	3.32E-04	6.70E-03	up
c26247_g1	13.305	195.496	3.87	3.34E-04	6.73E-03	up
c151_g1	4.479	67.574	3.89	3.34E-04	6.73E-03	up
c8556_g1	5.043	77.064	3.91	3.36E-04	6.75E-03	up
c10516_g1	14.991	0.284	-5.3	3.37E-04	6.75E-03	down
c2239_g1	0	5.378	5.78	3.37E-04	6.75E-03	up
c37772_g1	0	6.697	6.09	3.37E-04	6.75E-03	up
c6169_g1	15.207	220.241	3.85	3.38E-04	6.77E-03	up
c2110_g1	6.969	0.233	-4.41	3.40E-04	6.80E-03	down
c10537_g1	27.939	1.357	-4.27	3.40E-04	6.80E-03	down
c12338_g1	15.254	0.737	-4.2	3.41E-04	6.81E-03	down
c22129_g1	63.413	3.193	-4.27	3.41E-04	6.81E-03	down
c9923_g1	6.002	87.134	3.84	3.43E-04	6.83E-03	up
c26738_g1	62.624	2.909	-4.38	3.44E-04	6.84E-03	down
c34672_g1	208.061	3097.524	3.9	3.44E-04	6.84E-03	up
c5739_g1	12.082	0.194	-5.37	3.47E-04	6.90E-03	down
c26090_g1	62.059	888.564	3.84	3.48E-04	6.90E-03	up
c35448_g1	12.601	0.336	-4.86	3.52E-04	6.99E-03	down
c19078_g1	7.727	0.259	-4.45	3.53E-04	7.00E-03	down
c35529_g1	6.057	88.699	3.85	3.55E-04	7.00E-03	up
c28558_g1	35.613	1.655	-4.35	3.55E-04	7.00E-03	down
c1557_g1	0.859	13.962	3.87	3.55E-04	7.00E-03	up
c1809_g1	0.193	13.109	5.49	3.56E-04	7.00E-03	up
c26492_g1	0.186	11.183	5.3	3.56E-04	7.00E-03	up
c30690_g1	0.062	3.697	4.55	3.56E-04	7.00E-03	up
c13190_g1	0.998	30.962	4.82	3.56E-04	7.00E-03	up
c4883_g1	31.761	1.319	-4.49	3.58E-04	7.03E-03	down
c2928_g1	30.399	1.487	-4.26	3.59E-04	7.04E-03	down
c12271_g1	31.064	1.5	-4.28	3.59E-04	7.04E-03	down
c3226_g1	38.15	543.297	3.83	3.63E-04	7.10E-03	up
c6987_g1	17.652	0.918	-4.12	3.64E-04	7.13E-03	down
c7915_g1	26.833	0.944	-4.69	3.65E-04	7.13E-03	down
c13241_g1	4.834	70.238	3.83	3.68E-04	7.18E-03	up

seq_id	Fpkm of flower	Fpkm of leaf	log2FC (leaf/flower)	p-value	FDR	regulate
c30684_g1	7.024	101.239	3.83	3.74E-04	7.28E-03	up
c17714_g1	16.074	227.816	3.82	3.74E-04	7.28E-03	up
c13048_g2	23.484	0	-7.88	3.75E-04	7.28E-03	down
c1808_g1	12.956	0	-7.03	3.75E-04	7.28E-03	down
c26009_g1	5.221	0	-5.73	3.75E-04	7.28E-03	down
c7398_g1	9.158	0.349	-4.37	3.80E-04	7.36E-03	down
c11309_g1	1.903	23.025	3.53	3.80E-04	7.36E-03	up
c30196_g1	75.867	1064.539	3.81	3.80E-04	7.36E-03	up
c14219_g1	2.421	38.396	3.93	3.82E-04	7.38E-03	up
c35462_g1	21.527	1.138	-4.13	3.82E-04	7.38E-03	down
c2305_g1	0.101	6.063	4.94	3.83E-04	7.39E-03	up
c31623_g1	1.044	31.764	4.8	3.83E-04	7.39E-03	up
c24008_g1	0.248	7.408	4.43	3.83E-04	7.39E-03	up
c5822_g1	7.078	103.462	3.85	3.85E-04	7.41E-03	up
c6114_g1	6.335	0.155	-4.66	3.87E-04	7.43E-03	down
c4804_g1	0.394	9.412	4.27	3.87E-04	7.43E-03	up
c11868_g1	0.835	12.825	3.79	3.89E-04	7.43E-03	up
c7128_g1	19.872	278.817	3.8	3.89E-04	7.43E-03	up
c3433_g1	5.678	0.09	-4.93	3.89E-04	7.43E-03	down
c7417_g2	0.681	17.763	4.52	3.89E-04	7.43E-03	up
c6994_g1	0.201	5.197	4.14	3.89E-04	7.43E-03	up
c9163_g1	0.425	11.286	4.44	3.89E-04	7.43E-03	up
c4509_g1	1.098	17.246	3.86	3.91E-04	7.43E-03	up
c35791_g1	25.673	0.995	-4.56	3.91E-04	7.43E-03	down
c398_g1	10.559	0.336	-4.61	3.91E-04	7.43E-03	down
c28541_g1	37.291	1.5	-4.55	3.91E-04	7.43E-03	down
c3178_g1	12.964	182.129	3.8	3.93E-04	7.46E-03	up
c7074_g1	5.105	72.797	3.81	4.01E-04	7.62E-03	up
c7122_g1	0.89	13.717	3.8	4.07E-04	7.70E-03	up
c14553_g1	5.709	89.19	3.94	4.07E-04	7.70E-03	up
c18133_g1	1.199	19.793	3.94	4.07E-04	7.70E-03	up
c6968_g1	21.767	0.931	-4.41	4.15E-04	7.84E-03	down
c18121_g1	227.979	13.561	-4.06	4.16E-04	7.85E-03	down
c15627_g1	72.834	4.227	-4.08	4.16E-04	7.85E-03	down
c19065_g1	0	7.731	6.29	4.19E-04	7.87E-03	up
c12563_g1	4.61	0	-5.56	4.19E-04	7.87E-03	down
c177_g1	8.192	0	-6.37	4.19E-04	7.87E-03	down
c9302_g1	19.647	1.086	-4.06	4.27E-04	8.02E-03	down
c6277_g1	64.829	3.684	-4.1	4.29E-04	8.04E-03	down
c15461_g1	17.257	0.608	-4.62	4.31E-04	8.08E-03	down
c11888_g3	30.012	1.06	-4.7	4.31E-04	8.08E-03	down
c31971_g1	10.056	140.191	3.79	4.33E-04	8.09E-03	up

seq_id	Fpkm of flower	Fpkm of leaf	log2FC (leaf/flower)	p-value	FDR	regulate
c10943_g1	2.986	42.21	3.78	4.34E-04	8.10E-03	up
c19221_g1	153.652	9.088	-4.06	4.34E-04	8.10E-03	down
c30405_g1	117.513	7.007	-4.05	4.35E-04	8.12E-03	down
c1493_g1	0.719	11.661	3.84	4.36E-04	8.13E-03	up
c12393_g1	21.658	1.073	-4.21	4.39E-04	8.17E-03	down
c4997_g1	20.104	274.422	3.76	4.41E-04	8.19E-03	up
c2017_g1	65.115	3.736	-4.09	4.41E-04	8.20E-03	down
c11924_g1	1.284	20.517	3.9	4.44E-04	8.23E-03	up
c34948_g1	0.402	11.571	4.54	4.47E-04	8.27E-03	up
c30544_g1	0.309	9.05	4.48	4.47E-04	8.27E-03	up
c7041_g2	51.826	2.78	-4.17	4.49E-04	8.31E-03	down
c30640_g1	8.462	0.297	-4.43	4.50E-04	8.31E-03	down
c17723_g1	99.095	1329.756	3.74	4.52E-04	8.34E-03	up
c35212_g1	32.55	1.771	-4.13	4.54E-04	8.37E-03	down
c11888_g2	16.182	0.802	-4.17	4.55E-04	8.38E-03	down
c34409_g1	97.757	1308.709	3.74	4.55E-04	8.38E-03	up
c4138_g1	58.656	3.258	-4.13	4.62E-04	8.48E-03	down
c11663_g1	2017.002	127.78	-3.98	4.62E-04	8.48E-03	down
c6280_g1	20.127	1.073	-4.11	4.63E-04	8.48E-03	down
c851_g1	0.503	9.916	4.05	4.67E-04	8.48E-03	up
c24716_g1	15.316	0.543	-4.58	4.69E-04	8.48E-03	down
c1678_g1	9.236	0.336	-4.42	4.69E-04	8.48E-03	down
c8795_g1	9.22	0.31	-4.51	4.69E-04	8.48E-03	down
c23296_g1	18.889	0.672	-4.62	4.69E-04	8.48E-03	down
c4210_g1	0	11.131	6.81	4.69E-04	8.48E-03	up
c17070_g1	0	8.054	6.35	4.69E-04	8.48E-03	up
c35778_g1	0	6.412	6.03	4.69E-04	8.48E-03	up
c34059_g1	6.683	0	-6.08	4.69E-04	8.48E-03	down
c19224_g1	4.927	0	-5.65	4.69E-04	8.48E-03	down
c2988_g1	8.323	0	-6.4	4.69E-04	8.48E-03	down
c5871_g1	12.67	0	-7	4.69E-04	8.48E-03	down
c11226_g1	21.388	0	-7.75	4.69E-04	8.48E-03	down
c10896_g1	6.126	0	-5.96	4.69E-04	8.48E-03	down
c28127_g1	0.232	5.352	4.04	4.69E-04	8.48E-03	up
c463_g1	4.734	63.98	3.73	4.69E-04	8.48E-03	up
c2017_g2	38.862	2.094	-4.15	4.73E-04	8.54E-03	down
c12096_g2	2.305	31.027	3.69	4.74E-04	8.55E-03	up
c10459_g1	15.849	0.918	-3.97	4.82E-04	8.67E-03	down
c1498_g1	0.186	10.666	5.23	4.83E-04	8.67E-03	up
c3649_g1	0.139	8.39	5.15	4.83E-04	8.67E-03	up
c2537_g1	0.263	7.434	4.38	4.83E-04	8.67E-03	up
c21691_g1	11.464	155.885	3.75	4.83E-04	8.67E-03	up

seq_id	Fpkm of flower	Fpkm of leaf	log2FC (leaf/flower)	p-value	FDR	regulate
c1349_g1	9.553	0.427	-4.2	4.84E-04	8.68E-03	down
c13536_g1	25.472	1.513	-3.99	4.85E-04	8.68E-03	down
c14965_g1	17.017	234.681	3.78	4.91E-04	8.79E-03	up
c10858_g1	24.76	0.724	-4.92	4.93E-04	8.80E-03	down
c1454_g1	7.302	0.194	-4.65	4.93E-04	8.80E-03	down
c26878_g1	8.671	0.246	-4.66	4.93E-04	8.80E-03	down
c6962_g1	6.041	0.09	-5.01	4.94E-04	8.80E-03	down
c9542_g1	1.609	23.736	3.8	4.94E-04	8.80E-03	up
c12210_g1	0.944	15.837	3.93	4.97E-04	8.84E-03	up
c22522_g1	49.76	2.999	-4.01	5.03E-04	8.95E-03	down
c10854_g1	0.634	13.342	4.19	5.10E-04	9.05E-03	up
c26212_g1	127.143	1655.993	3.7	5.11E-04	9.07E-03	up
c13813_g1	232.697	14.66	-3.98	5.12E-04	9.09E-03	down
c9128_g1	0.387	8.52	4.15	5.18E-04	9.18E-03	up
c11974_g1	0.093	5.184	4.77	5.23E-04	9.20E-03	up
c2623_g1	0.248	6.71	4.29	5.23E-04	9.20E-03	up
c3076_g1	0.201	5.559	4.23	5.23E-04	9.20E-03	up
c37218_g1	0.286	7.899	4.37	5.23E-04	9.20E-03	up
c21015_g1	0.387	10.653	4.46	5.23E-04	9.20E-03	up
c2042_g1	0	6.011	5.93	5.27E-04	9.20E-03	up
c14655_g1	0	2.922	4.92	5.27E-04	9.20E-03	up
c21659_g1	0	4.473	5.52	5.27E-04	9.20E-03	up
c2904_g1	0	5.818	5.89	5.27E-04	9.20E-03	up
c15605_g1	0	4.318	5.47	5.27E-04	9.20E-03	up
c15903_g1	0	4.15	5.41	5.27E-04	9.20E-03	up
c20409_g1	0	13.548	7.09	5.27E-04	9.20E-03	up
c32154_g1	0	10.11	6.67	5.27E-04	9.20E-03	up
c30241_g1	0	5.275	5.75	5.27E-04	9.20E-03	up
c11080_g1	2.576	0	-4.74	5.27E-04	9.20E-03	down
c26066_g1	32.055	415.388	3.69	5.27E-04	9.20E-03	up
c34647_g1	169.516	10.666	-3.98	5.31E-04	9.25E-03	down
c9319_g1	27.599	1.642	-3.99	5.31E-04	9.25E-03	down
c15319_g1	4.409	63.153	3.81	5.32E-04	9.26E-03	up
c22949_g1	12.546	165.969	3.72	5.35E-04	9.30E-03	up
c22751_g2	23.414	1.319	-4.05	5.36E-04	9.30E-03	down
c10447_g3	91.322	5.624	-4	5.39E-04	9.35E-03	down
c29973_g1	46.21	2.831	-3.98	5.40E-04	9.36E-03	down
c36538_g1	24.737	1.202	-4.25	5.40E-04	9.36E-03	down
c8484_g1	12.43	0.685	-4	5.41E-04	9.36E-03	down
c19945_g1	10.133	133.649	3.71	5.46E-04	9.44E-03	up
c11654_g1	27.537	1.202	-4.41	5.61E-04	9.67E-03	down
c8122_g1	24.497	1.073	-4.39	5.61E-04	9.67E-03	down

seq_id	Fpkm of flower	Fpkm of leaf	log2FC (leaf/flower)	p-value	FDR	regulate
c31115_g1	1.369	23.529	4.01	5.61E-04	9.67E-03	up
c4272_g1	49.683	3.103	-3.96	5.62E-04	9.68E-03	down
c294_g1	41.94	2.56	-3.98	5.63E-04	9.69E-03	down
c34509_g1	72.332	4.615	-3.94	5.64E-04	9.69E-03	down
c3965_g1	31.01	1.926	-3.94	5.64E-04	9.69E-03	down
c7113_g1	17.35	0.853	-4.19	5.67E-04	9.69E-03	down
c12728_g1	0.147	7.925	5.02	5.67E-04	9.69E-03	up
c6076_g1	0.162	8.52	5.04	5.67E-04	9.69E-03	up
c10036_g1	0.758	42.21	5.62	5.67E-04	9.69E-03	up
c19315_g1	0.077	4.176	4.59	5.67E-04	9.69E-03	up
c2691_g1	0.379	7.757	4.04	5.67E-04	9.69E-03	up
c8528_g1	7.093	0.388	-3.88	5.68E-04	9.70E-03	down
c5208_g1	15.316	0.465	-4.77	5.74E-04	9.77E-03	down
c26527_g1	7.016	0.207	-4.53	5.74E-04	9.77E-03	down
c18541_g1	23.206	1.293	-4.06	5.77E-04	9.82E-03	down
c18170_g1	20.939	266.898	3.67	5.77E-04	9.82E-03	up
c1783_g1	21.295	1.112	-4.14	5.81E-04	9.86E-03	down
c31872_g1	35.033	1.836	-4.18	5.81E-04	9.86E-03	down
c2881_g1	26.903	1.681	-3.92	5.82E-04	9.87E-03	down
c13086_g1	1.083	15.087	3.68	5.84E-04	9.89E-03	up
c12092_g2	18.487	1.008	-4.07	5.88E-04	9.95E-03	down
c12951_g1	28.636	1.706	-3.99	5.90E-04	9.95E-03	down
c9362_g2	1.122	17	3.81	5.91E-04	9.95E-03	up
c14744_g1	0	14.996	7.24	5.93E-04	9.95E-03	up
c19151_g1	0	5.378	5.78	5.93E-04	9.95E-03	up
c27753_g1	0	5.055	5.69	5.93E-04	9.95E-03	up
c3392_g1	0	5.869	5.9	5.93E-04	9.95E-03	up
c32190_g1	0	15.345	7.27	5.93E-04	9.95E-03	up
c15542_g1	3.597	0	-5.21	5.93E-04	9.95E-03	down
c11011_g1	4.069	0	-5.38	5.93E-04	9.95E-03	down
c6703_g1	4.038	0	-5.37	5.93E-04	9.95E-03	down
c11955_g1	4.951	0	-5.66	5.93E-04	9.95E-03	down
c8065_g1	14.859	0.284	-5.28	5.95E-04	9.97E-03	down
c37299_g1	16.615	0.827	-4.17	5.96E-04	9.98E-03	down
c31125_g1	24.312	1.151	-4.29	5.99E-04	1.00E-02	down
c17711_g1	120.421	1512.001	3.65	5.99E-04	1.00E-02	up
c7519_g1	1.725	23.645	3.7	6.04E-04	1.01E-02	up
c22061_g1	3.264	41.576	3.63	6.10E-04	1.02E-02	up
c13727_g1	40.432	2.469	-3.98	6.11E-04	1.02E-02	down
c23098_g1	45.336	2.909	-3.92	6.11E-04	1.02E-02	down
c30795_g1	28.396	1.745	-3.95	6.14E-04	1.02E-02	down
c30201_g1	25.797	324.595	3.65	6.15E-04	1.02E-02	up

seq_id	Fpkm of flower	Fpkm of leaf	log2FC (leaf/flower)	p-value	FDR	regulate
c3716_g1	21.264	1.008	-4.27	6.15E-04	1.02E-02	down
c23591_g1	2.003	28.752	3.78	6.23E-04	1.03E-02	up
c12703_g1	104.889	6.942	-3.9	6.31E-04	1.05E-02	down
c5253_g1	6.575	83.178	3.64	6.34E-04	1.05E-02	up
c1958_g1	0.356	8.119	4.17	6.35E-04	1.05E-02	up
c2582_g1	20.498	0.633	-4.81	6.36E-04	1.05E-02	down
c12114_g1	6.08	0.194	-4.39	6.36E-04	1.05E-02	down
c5662_g1	0.673	10.963	3.84	6.45E-04	1.06E-02	up
c5012_g1	0.959	14.247	3.76	6.46E-04	1.06E-02	up
c25991_g1	25.975	319.838	3.62	6.53E-04	1.07E-02	up
c238_g1	0.688	13.316	4.09	6.53E-04	1.07E-02	up
c10959_g1	0.874	14.234	3.88	6.53E-04	1.07E-02	up
c11655_g1	3.434	43.684	3.63	6.53E-04	1.07E-02	up
c14470_g1	368.249	24.576	-3.9	6.54E-04	1.07E-02	down
c8453_g2	58.13	2.34	-4.58	6.62E-04	1.08E-02	down
c460_g1	5.175	0.207	-4.1	6.62E-04	1.08E-02	down
c1459_g1	51.555	3.167	-3.98	6.63E-04	1.08E-02	down
c14161_g1	16.383	207.791	3.66	6.63E-04	1.08E-02	up
c8411_g1	50.286	3.116	-3.97	6.65E-04	1.08E-02	down
c30286_g1	172.85	11.402	-3.91	6.65E-04	1.08E-02	down
c18865_g1	6.776	84.523	3.62	6.67E-04	1.08E-02	up
c9441_g1	0.101	5.223	4.73	6.70E-04	1.08E-02	up
c4805_g1	0.317	16.587	5.32	6.70E-04	1.08E-02	up
c31404_g1	0.364	19.185	5.38	6.70E-04	1.08E-02	up
c7948_g1	0.255	6.787	4.28	6.70E-04	1.08E-02	up
c9874_g1	5.005	0	-5.67	6.71E-04	1.08E-02	down
c8497_g1	8.199	0	-6.37	6.71E-04	1.08E-02	down
c6934_g2	9.313	0	-6.56	6.71E-04	1.08E-02	down
c11522_g1	8.555	0	-6.44	6.71E-04	1.08E-02	down
c12512_g1	4.533	0	-5.53	6.71E-04	1.08E-02	down
c4087_g1	5.794	0	-5.88	6.71E-04	1.08E-02	down
c22026_g1	104.023	6.865	-3.9	6.72E-04	1.09E-02	down
c27221_g1	51.71	3.322	-3.92	6.82E-04	1.10E-02	down
c31243_g1	8.524	108.724	3.66	6.83E-04	1.10E-02	up
c15868_g1	6.273	83.153	3.71	6.84E-04	1.10E-02	up
c8679_g1	15.787	0.944	-3.93	6.85E-04	1.10E-02	down
c163_g1	17.721	92.099	2.37	6.98E-04	1.12E-02	up
c7697_g1	2.398	29.502	3.57	7.02E-04	1.13E-02	up
c13061_g1	0.449	8.158	3.91	7.06E-04	1.13E-02	up
c3996_g1	0.665	12.463	4.04	7.06E-04	1.13E-02	up
c32016_g1	35.11	2.133	-3.98	7.06E-04	1.13E-02	down
c22567_g1	5.051	62.287	3.6	7.07E-04	1.13E-02	up

seq_id	Fpkm of flower	Fpkm of leaf	log2FC (leaf/flower)	p-value	FDR	regulate
c26083_g1	32.263	2.094	-3.88	7.08E-04	1.13E-02	down
c7632_g1	72.494	2.392	-4.86	7.08E-04	1.13E-02	down
c13022_g1	17.729	0.569	-4.74	7.08E-04	1.13E-02	down
c9119_g1	0.92	13.393	3.73	7.08E-04	1.13E-02	up
c30647_g1	30.012	367.529	3.61	7.10E-04	1.13E-02	up
c270_g1	7.132	90.651	3.65	7.11E-04	1.13E-02	up
c10434_g2	12.345	141.535	3.51	7.12E-04	1.13E-02	up
c31213_g1	0.217	4.77	3.94	7.24E-04	1.15E-02	up
c18506_g1	0.302	6.723	4.09	7.24E-04	1.15E-02	up
c14694_g1	6.575	0.259	-4.22	7.25E-04	1.15E-02	down
c7023_g1	95.932	6.503	-3.86	7.25E-04	1.15E-02	down
c1525_g1	0.186	9.502	5.07	7.30E-04	1.16E-02	up
c355_g1	8.733	105.932	3.59	7.33E-04	1.16E-02	up
c8790_g1	1.253	11.26	3.07	7.35E-04	1.16E-02	up
c862_g2	0.588	9.373	3.78	7.35E-04	1.16E-02	up
c18092_g1	2.235	32.268	3.79	7.36E-04	1.16E-02	up
c34497_g1	116.894	1389.367	3.57	7.38E-04	1.17E-02	up
c23952_g1	30.84	2.056	-3.84	7.42E-04	1.17E-02	down
c18139_g1	191.778	13.044	-3.87	7.43E-04	1.17E-02	down
c30231_g1	131.382	1556.758	3.57	7.44E-04	1.17E-02	up
c6576_g1	11.881	0.543	-4.22	7.46E-04	1.17E-02	down
c10359_g1	17.381	1.086	-3.88	7.48E-04	1.18E-02	down
c12795_g1	191.229	13.678	-3.8	7.52E-04	1.18E-02	down
c26368_g1	1.601	22.986	3.76	7.54E-04	1.18E-02	up
c10727_g1	4.486	55.655	3.6	7.57E-04	1.18E-02	up
c18499_g1	15.246	181.25	3.56	7.59E-04	1.18E-02	up
c21902_g1	64.442	4.383	-3.85	7.60E-04	1.18E-02	down
c2336_g1	0	6.192	5.98	7.61E-04	1.18E-02	up
c7560_g1	0	15.449	7.28	7.61E-04	1.18E-02	up
c31786_g1	0	57.09	9.16	7.61E-04	1.18E-02	up
c3720_g1	0	8.869	6.49	7.61E-04	1.18E-02	up
c5516_g1	0	15.501	7.29	7.61E-04	1.18E-02	up
c7834_g1	0	34.582	8.44	7.61E-04	1.18E-02	up
c6808_g2	0	29.321	8.2	7.61E-04	1.18E-02	up
c6807_g1	0	5.016	5.68	7.61E-04	1.18E-02	up
c8638_g1	4.363	0	-5.48	7.61E-04	1.18E-02	down
c13025_g1	9.189	0	-6.54	7.61E-04	1.18E-02	down
c14246_g1	4.339	0	-5.47	7.61E-04	1.18E-02	down
c19376_g1	4.657	0	-5.57	7.61E-04	1.18E-02	down
c24318_g1	3.458	0	-5.15	7.61E-04	1.18E-02	down
c10989_g1	4.254	0	-5.44	7.61E-04	1.18E-02	down
c12923_g1	23.662	0	-7.89	7.61E-04	1.18E-02	down

seq_id	Fpkm of flower	Fpkm of leaf	log2FC (leaf/flower)	p-value	FDR	regulate
c12740_g1	7.217	0	-6.19	7.61E-04	1.18E-02	down
c99_g1	23.329	277.925	3.57	7.63E-04	1.18E-02	up
c34646_g1	15.826	189.382	3.57	7.69E-04	1.19E-02	up
c2626_g1	0.951	13.432	3.69	7.70E-04	1.19E-02	up
c14216_g1	8.284	98.744	3.56	7.74E-04	1.19E-02	up
c9934_g1	1.771	23.387	3.65	7.75E-04	1.19E-02	up
c6356_g1	6.103	73.353	3.57	7.78E-04	1.20E-02	up
c2995_g1	28.636	1.887	-3.85	7.79E-04	1.20E-02	down
c9748_g2	19.5	230.118	3.55	7.80E-04	1.20E-02	up
c8104_g1	46.844	3.064	-3.89	7.83E-04	1.20E-02	down
c36746_g1	6.103	74.995	3.6	7.84E-04	1.20E-02	up
c13141_g1	15.022	0.879	-3.95	7.87E-04	1.21E-02	down
c11556_g1	2.955	17.724	2.54	7.91E-04	1.21E-02	up
c26023_g1	15.478	185.232	3.57	7.93E-04	1.21E-02	up
c14153_g1	0.487	7.447	3.68	7.93E-04	1.21E-02	up
c5904_g1	102.182	7.11	-3.83	7.94E-04	1.21E-02	down
c3344_g1	0.108	5.288	4.7	7.97E-04	1.21E-02	up
c1982_g1	0.286	8.08	4.41	7.97E-04	1.21E-02	up
c29492_g1	0.286	7.059	4.21	7.97E-04	1.21E-02	up
c30424_g1	0.333	8.183	4.26	7.97E-04	1.21E-02	up
c22380_g1	8.532	102.053	3.56	7.99E-04	1.22E-02	up
c7752_g1	26.795	2.482	-3.38	8.06E-04	1.23E-02	down
c12490_g1	0.998	12.463	3.52	8.09E-04	1.23E-02	up
c9966_g1	13.869	172.692	3.63	8.21E-04	1.24E-02	up
c13384_g1	3380.087	243.472	-3.79	8.22E-04	1.24E-02	down
c30149_g1	106.134	1228.841	3.53	8.25E-04	1.25E-02	up
c4911_g1	1.702	26.283	3.87	8.25E-04	1.25E-02	up
c4021_g1	5.159	0.103	-4.7	8.28E-04	1.25E-02	down
c14182_g1	4.339	52.888	3.58	8.31E-04	1.26E-02	up
c12429_g1	28.427	4.68	-2.58	8.33E-04	1.26E-02	down
c33_g1	1.083	21.538	4.19	8.35E-04	1.26E-02	up
c33002_g1	0.719	14.234	4.13	8.35E-04	1.26E-02	up
c35474_g1	24.869	1.694	-3.8	8.38E-04	1.26E-02	down
c6687_g1	24.845	261.843	3.39	8.42E-04	1.27E-02	up
c8225_g1	38.73	2.676	-3.81	8.49E-04	1.27E-02	down
c10558_g1	16.708	1.086	-3.82	8.49E-04	1.27E-02	down
c35196_g1	66.925	4.706	-3.8	8.58E-04	1.27E-02	down
c26467_g1	138.576	9.851	-3.8	8.60E-04	1.27E-02	down
c11047_g2	2.042	24.227	3.51	8.62E-04	1.27E-02	up
c35333_g1	60.466	4.034	-3.87	8.62E-04	1.27E-02	down
c11190_g1	9.731	0.633	-3.75	8.63E-04	1.27E-02	down
c23514_g1	9.336	0.478	-4.03	8.66E-04	1.27E-02	down

seq_id	Fpkm of flower	Fpkm of leaf	log2FC (leaf/flower)	p-value	FDR	regulate
c4192_g1	0	9.05	6.52	8.66E-04	1.27E-02	up
c4100_g1	0	6.335	6.01	8.66E-04	1.27E-02	up
c15276_g1	0	9.567	6.59	8.66E-04	1.27E-02	up
c29530_g1	0	13.794	7.12	8.66E-04	1.27E-02	up
c32146_g1	0	43.218	8.76	8.66E-04	1.27E-02	up
c19660_g1	0	4.46	5.51	8.66E-04	1.27E-02	up
c682_g2	0	4.189	5.42	8.66E-04	1.27E-02	up
c224_g1	0	5.662	5.85	8.66E-04	1.27E-02	up
c16314_g1	0	3.439	5.15	8.66E-04	1.27E-02	up
c31085_g1	0	4.719	5.59	8.66E-04	1.27E-02	up
c4149_g1	0	7.744	6.29	8.66E-04	1.27E-02	up
c36222_g1	0	9.605	6.6	8.66E-04	1.27E-02	up
c6934_g1	18.843	0	-7.57	8.66E-04	1.27E-02	down
c12890_g1	9.29	0	-6.55	8.66E-04	1.27E-02	down
c6902_g1	6.869	0	-6.12	8.66E-04	1.27E-02	down
c11120_g1	21.179	0	-7.73	8.66E-04	1.27E-02	down
c32143_g1	4.958	0	-5.66	8.66E-04	1.27E-02	down
c10334_g1	4.401	0	-5.49	8.66E-04	1.27E-02	down
c15148_g1	3.821	0	-5.29	8.66E-04	1.27E-02	down
c10255_g2	6.312	0	-6	8.66E-04	1.27E-02	down
c11051_g1	8.091	0	-6.36	8.66E-04	1.27E-02	down
c30104_g1	23.298	1.513	-3.86	8.70E-04	1.27E-02	down
c14094_g1	0.147	6.981	4.84	8.71E-04	1.27E-02	up
c14063_g1	0.093	4.473	4.57	8.71E-04	1.27E-02	up
c16029_g1	0.255	6.063	4.12	8.71E-04	1.27E-02	up
c2425_g1	0.201	4.861	4.04	8.71E-04	1.27E-02	up
c4019_g1	0.193	4.279	3.9	8.71E-04	1.27E-02	up
c9718_g1	10.705	122.699	3.51	8.78E-04	1.28E-02	up
c11585_g1	11.077	0.336	-4.68	8.83E-04	1.29E-02	down
c3131_g1	114.102	1296.182	3.5	8.84E-04	1.29E-02	up
c13276_g1	13.97	159.608	3.5	8.84E-04	1.29E-02	up
c28690_g1	4.981	62.158	3.62	8.90E-04	1.30E-02	up
c12579_g2	1.802	21.641	3.51	8.90E-04	1.30E-02	up
c27444_g1	100.318	7.085	-3.8	8.99E-04	1.31E-02	down
c14601_g1	17.157	194.217	3.49	9.12E-04	1.33E-02	up
c20335_g1	2.119	39.663	4.16	9.17E-04	1.33E-02	up
c8409_g1	1.16	21.46	4.1	9.17E-04	1.33E-02	up
c22694_g1	0.688	12.618	4.01	9.17E-04	1.33E-02	up
c34608_g1	89.573	1007.462	3.49	9.18E-04	1.33E-02	up
c35310_g1	2.096	24.136	3.46	9.26E-04	1.34E-02	up
c23495_g1	89.171	6.141	-3.84	9.29E-04	1.34E-02	down
c5551_g1	2.344	28.803	3.56	9.29E-04	1.34E-02	up

seq_id	Fpkm of flower	Fpkm of leaf	log2FC (leaf/flower)	p-value	FDR	regulate
c12469_g1	25.209	1.706	-3.81	9.32E-04	1.35E-02	down
c4489_g1	16.329	0.517	-4.73	9.35E-04	1.35E-02	down
c7296_g1	0.611	11.726	4.06	9.35E-04	1.35E-02	up
c13165_g1	7.201	37.22	2.35	9.39E-04	1.35E-02	up
c31043_g1	111.294	7.977	-3.79	9.41E-04	1.36E-02	down
c13063_g1	33.625	385.524	3.52	9.45E-04	1.36E-02	up
c3666_g1	12.879	144.806	3.48	9.45E-04	1.36E-02	up
c9468_g1	7.642	86.01	3.48	9.49E-04	1.36E-02	up
c12567_g1	0.317	6.503	3.99	9.51E-04	1.37E-02	up
c3154_g1	4.015	45.726	3.48	9.52E-04	1.37E-02	up
c180_g1	0.425	10.032	4.27	9.54E-04	1.37E-02	up
c18446_g1	0.333	7.757	4.18	9.54E-04	1.37E-02	up
c28167_g1	1.3	31.195	4.48	9.54E-04	1.37E-02	up
c10806_g1	38.452	2.482	-3.9	9.54E-04	1.37E-02	down
c8841_g1	3.512	40.801	3.5	9.56E-04	1.37E-02	up
c4837_g1	65.648	4.615	-3.8	9.56E-04	1.37E-02	down
c15334_g1	2.189	28.054	3.62	9.68E-04	1.38E-02	up
c18013_g1	5.701	64.252	3.47	9.69E-04	1.38E-02	up
c30421_g1	17.876	200.383	3.48	9.70E-04	1.38E-02	up
c35425_g1	125.743	9.179	-3.76	9.74E-04	1.39E-02	down
c35444_g1	1.083	16.16	3.78	9.80E-04	1.39E-02	up
c28421_g1	2.46	35.81	3.81	9.83E-04	1.39E-02	up
c11010_g1	2.367	26.709	3.44	9.87E-04	1.39E-02	up
c1674_g1	0	7.847	6.31	9.90E-04	1.39E-02	up
c36301_g1	0	3.814	5.29	9.90E-04	1.39E-02	up
c1466_g1	0	5.21	5.73	9.90E-04	1.39E-02	up
c24355_g1	0	15.307	7.27	9.90E-04	1.39E-02	up
c6785_g1	0	6.645	6.08	9.90E-04	1.39E-02	up
c28481_g1	4.038	0	-5.37	9.90E-04	1.39E-02	down
c35913_g1	4.587	0	-5.55	9.90E-04	1.39E-02	down
c10183_g1	21.071	0	-7.73	9.90E-04	1.39E-02	down
c5207_g1	8.71	0	-6.46	9.90E-04	1.39E-02	down
c10520_g1	5.484	0	-5.8	9.90E-04	1.39E-02	down
c12358_g1	3.605	0	-5.21	9.90E-04	1.39E-02	down
c26447_g1	4.247	0	-5.44	9.90E-04	1.39E-02	down
c7844_g1	12.376	0	-6.96	9.90E-04	1.39E-02	down
c15153_g1	11.881	0.414	-4.54	9.90E-04	1.39E-02	down
c22866_g1	9.298	0.323	-4.47	9.90E-04	1.39E-02	down
c21695_g1	12.972	149.111	3.51	9.99E-04	1.40E-02	up
c8059_g1	7.689	0.375	-4.04	1.00E-03	1.40E-02	down
c10182_g2	1.671	24.421	3.79	1.00E-03	1.40E-02	up
c17660_g1	320.314	3530.766	3.46	1.00E-03	1.40E-02	up

seq_id	Fpkm of flower	Fpkm of leaf	log2FC (leaf/flower)	p-value	FDR	regulate
c14018_g1	366.431	27.213	-3.75	1.01E-03	1.41E-02	down
c26571_g1	2.947	39.12	3.69	1.01E-03	1.41E-02	up
c10578_g1	9.104	0.543	-3.84	1.01E-03	1.42E-02	down
c3366_g1	26.129	1.784	-3.8	1.02E-03	1.42E-02	down
c32525_g1	9.29	0.207	-4.93	1.02E-03	1.43E-02	down
c1241_g1	32.712	2.301	-3.77	1.03E-03	1.44E-02	down
c26662_g1	3.481	38.564	3.43	1.03E-03	1.44E-02	up
c22668_g1	9.437	104.057	3.45	1.03E-03	1.44E-02	up
c13729_g1	103.226	7.679	-3.73	1.04E-03	1.44E-02	down
c15604_g1	0.101	4.783	4.6	1.05E-03	1.46E-02	up
c10510_g2	5.152	56.922	3.44	1.05E-03	1.46E-02	up
c6190_g1	0.603	11.26	4.01	1.05E-03	1.46E-02	up
c1797_g1	854.582	9319.592	3.45	1.05E-03	1.46E-02	up
c18707_g1	6.799	78.072	3.5	1.05E-03	1.46E-02	up
c18617_g1	3.148	37.827	3.55	1.05E-03	1.46E-02	up
c30974_g1	49.923	3.503	-3.8	1.06E-03	1.46E-02	down
c3970_g1	1804.578	139.169	-3.7	1.06E-03	1.46E-02	down
c7476_g1	10.009	0.646	-3.76	1.06E-03	1.47E-02	down
c9208_g2	16.963	1.073	-3.86	1.07E-03	1.47E-02	down
c15516_g1	1.4	18.254	3.61	1.07E-03	1.47E-02	up
c10801_g1	456.298	4958.813	3.44	1.07E-03	1.47E-02	up
c35008_g1	22.556	247.855	3.45	1.07E-03	1.47E-02	up
c10349_g2	8.416	0.491	-3.85	1.07E-03	1.47E-02	down
c8525_g1	11.34	0.401	-4.51	1.07E-03	1.47E-02	down
c22054_g1	2.344	25.933	3.41	1.08E-03	1.48E-02	up
c6686_g1	36.03	2.508	-3.79	1.08E-03	1.48E-02	down
c3101_g1	143.317	10.717	-3.73	1.08E-03	1.49E-02	down
c5825_g1	7.596	83.566	3.44	1.09E-03	1.49E-02	up
c32373_g1	4.425	51.764	3.52	1.09E-03	1.49E-02	up
c2105_g1	15.13	1.034	-3.75	1.09E-03	1.50E-02	down
c5975_g2	201.718	2169.078	3.43	1.10E-03	1.50E-02	up
c4423_g1	9.236	0.207	-4.93	1.10E-03	1.50E-02	down
c26564_g1	0.797	15.863	4.15	1.10E-03	1.50E-02	up
c22430_g1	24.366	1.642	-3.81	1.10E-03	1.50E-02	down
c7940_g1	5.136	55.538	3.41	1.11E-03	1.51E-02	up
c8806_g1	4.015	45.455	3.47	1.11E-03	1.51E-02	up
c9117_g1	11.332	0.284	-4.9	1.11E-03	1.51E-02	down
c10811_g1	11.054	0.349	-4.63	1.11E-03	1.51E-02	down
c6117_g1	9.514	0.31	-4.55	1.11E-03	1.51E-02	down
c18117_g1	0.449	8.261	3.93	1.11E-03	1.51E-02	up
c1640_g1	1.369	17.75	3.6	1.11E-03	1.51E-02	up
c8342_g1	123.6	9.45	-3.7	1.12E-03	1.51E-02	down

seq_id	Fpkm of flower	Fpkm of leaf	log2FC (leaf/flower)	p-value	FDR	regulate
c11480_g1	93.998	6.748	-3.78	1.12E-03	1.51E-02	down
c652_g1	8.934	0.401	-4.17	1.12E-03	1.51E-02	down
c13410_g1	984.031	76.702	-3.68	1.12E-03	1.52E-02	down
c34844_g1	70.862	5.197	-3.74	1.12E-03	1.52E-02	down
c34879_g1	10.04	108.388	3.42	1.13E-03	1.52E-02	up
c27389_g1	1.238	15.268	3.52	1.13E-03	1.52E-02	up
c17994_g1	4.78	53.832	3.47	1.14E-03	1.52E-02	up
c18625_g1	0	3.646	5.23	1.14E-03	1.52E-02	up
c35907_g1	0	5.52	5.81	1.14E-03	1.52E-02	up
c28047_g1	0	2.663	4.79	1.14E-03	1.52E-02	up
c23227_g1	0	3.31	5.09	1.14E-03	1.52E-02	up
c23162_g1	0	19.34	7.6	1.14E-03	1.52E-02	up
c30575_g1	0	4.486	5.52	1.14E-03	1.52E-02	up
c28817_g1	0	7.511	6.25	1.14E-03	1.52E-02	up
c10153_g1	15.339	0	-7.27	1.14E-03	1.52E-02	down
c4391_g1	5.399	0	-5.78	1.14E-03	1.52E-02	down
c27140_g1	5.709	0	-5.86	1.14E-03	1.52E-02	down
c13175_g1	26.911	0	-8.08	1.14E-03	1.52E-02	down
c29258_g1	40.308	0	-8.66	1.14E-03	1.52E-02	down
c30841_g1	4.958	0	-5.66	1.14E-03	1.52E-02	down
c6352_g2	22.099	0	-7.79	1.14E-03	1.52E-02	down
c3793_g1	11.796	0.608	-4.07	1.15E-03	1.53E-02	down
c27106_g1	0.959	12.282	3.55	1.15E-03	1.53E-02	up
c8912_g1	18.735	1.293	-3.76	1.15E-03	1.53E-02	down
c3907_g1	70.305	746.68	3.41	1.15E-03	1.53E-02	up
c15125_g1	0.108	5.003	4.62	1.15E-03	1.53E-02	up
c19522_g1	0.394	18.241	5.21	1.15E-03	1.53E-02	up
c37339_g1	0.317	7.201	4.13	1.15E-03	1.53E-02	up
c4947_g1	0.843	19.418	4.37	1.15E-03	1.53E-02	up
c568_g1	0.356	8.132	4.17	1.15E-03	1.53E-02	up
c35922_g1	46.071	3.154	-3.83	1.15E-03	1.53E-02	down
c11354_g2	19.431	1.099	-4.03	1.16E-03	1.53E-02	down
c30623_g1	31.026	335.61	3.43	1.16E-03	1.53E-02	up
c11369_g2	8.277	0.427	-3.99	1.17E-03	1.54E-02	down
c10949_g1	9.421	94.93	3.32	1.17E-03	1.55E-02	up
c18090_g1	11.618	125.246	3.42	1.17E-03	1.55E-02	up
c12641_g1	1.872	22.546	3.52	1.17E-03	1.55E-02	up
c26105_g1	20.653	0.944	-4.31	1.17E-03	1.55E-02	down
c30488_g1	6.629	70.923	3.4	1.18E-03	1.55E-02	up
c13911_g1	8.176	87.031	3.4	1.18E-03	1.55E-02	up
c8433_g1	22.092	1.668	-3.65	1.18E-03	1.55E-02	down
c5690_g2	2.7	29.282	3.39	1.18E-03	1.56E-02	up

seq_id	Fpkm of flower	Fpkm of leaf	log2FC (leaf/flower)	p-value	FDR	regulate
c8982_g1	2.019	23.619	3.48	1.18E-03	1.56E-02	up
c10282_g1	5.925	0.181	-4.42	1.18E-03	1.56E-02	down
c12258_g1	1.74	19.224	3.39	1.18E-03	1.56E-02	up
c30401_g1	13.467	145.879	3.43	1.18E-03	1.56E-02	up
c1920_g1	7.874	0.401	-3.99	1.19E-03	1.56E-02	down
c9678_g1	1.098	12.553	3.4	1.19E-03	1.56E-02	up
c34493_g1	28.891	306.561	3.4	1.19E-03	1.57E-02	up
c30119_g1	96.976	7.278	-3.72	1.20E-03	1.57E-02	down
c17687_g1	1013.827	80.153	-3.66	1.20E-03	1.57E-02	down
c35297_g1	0.441	6.141	3.53	1.20E-03	1.57E-02	up
c3145_g1	12.198	129.176	3.39	1.21E-03	1.58E-02	up
c6395_g1	2.266	25.403	3.43	1.21E-03	1.58E-02	up
c12883_g1	1.57	17.931	3.43	1.21E-03	1.58E-02	up
c9712_g1	9.244	0.517	-3.92	1.21E-03	1.58E-02	down
c30576_g1	28.759	2.146	-3.68	1.21E-03	1.59E-02	down
c9462_g1	0.333	5.637	3.73	1.22E-03	1.59E-02	up
c1780_g1	42.11	2.896	-3.82	1.22E-03	1.59E-02	down
c6897_g1	30.593	2.249	-3.71	1.23E-03	1.60E-02	down
c35024_g1	12.384	134.115	3.43	1.23E-03	1.60E-02	up
c30742_g1	3.28	35.966	3.42	1.23E-03	1.60E-02	up
c13033_g3	12.446	0.75	-3.88	1.23E-03	1.60E-02	down
c870_g1	14.473	0.672	-4.24	1.24E-03	1.60E-02	down
c4426_g1	14.859	0.698	-4.23	1.24E-03	1.60E-02	down
c330_g1	21.535	1.525	-3.73	1.24E-03	1.61E-02	down
c34550_g1	8.315	88.918	3.4	1.24E-03	1.61E-02	up
c11664_g2	47.362	3.568	-3.69	1.25E-03	1.61E-02	down
c26324_g1	272.209	21.538	-3.65	1.26E-03	1.62E-02	down
c10420_g1	16.43	0.608	-4.55	1.26E-03	1.62E-02	down
c21705_g1	0.317	5.585	3.77	1.26E-03	1.62E-02	up
c14079_g1	3.434	37.077	3.4	1.27E-03	1.63E-02	up
c3392_g2	0.224	4.822	3.93	1.27E-03	1.63E-02	up
c13913_g1	0.425	9.424	4.18	1.27E-03	1.63E-02	up
c5570_g1	0.263	5.753	4.01	1.27E-03	1.63E-02	up
c12787_g1	0.797	10.704	3.59	1.27E-03	1.64E-02	up
c27486_g1	0.348	6.748	3.93	1.27E-03	1.64E-02	up
c6784_g1	9.661	0.504	-4.01	1.27E-03	1.64E-02	down
c30159_g1	56.436	4.344	-3.67	1.28E-03	1.64E-02	down
c10200_g1	3.767	38.344	3.31	1.28E-03	1.65E-02	up
c2522_g1	21.906	1.409	-3.87	1.29E-03	1.65E-02	down
c26485_g1	1.346	16.716	3.54	1.30E-03	1.67E-02	up
c4040_g1	13.684	1.008	-3.64	1.30E-03	1.67E-02	down
c5574_g1	45.251	473.435	3.38	1.30E-03	1.67E-02	up

seq_id	Fpkm of flower	Fpkm of leaf	log2FC (leaf/flower)	p-value	FDR	regulate
c12571_g3	0.464	7.757	3.8	1.30E-03	1.67E-02	up
c30463_g1	30.538	317.756	3.37	1.31E-03	1.67E-02	up
c9684_g1	3.605	0	-5.21	1.31E-03	1.67E-02	down
c5244_g1	3.055	0	-4.98	1.31E-03	1.67E-02	down
c1581_g1	4.076	0	-5.38	1.31E-03	1.67E-02	down
c9800_g1	20.87	1.228	-3.98	1.31E-03	1.67E-02	down
c34582_g1	22.378	230.57	3.36	1.31E-03	1.67E-02	up
c290_g1	10.017	0.53	-4.01	1.32E-03	1.68E-02	down
c9484_g1	1.006	11.532	3.39	1.32E-03	1.68E-02	up
c34899_g1	352.345	3612.509	3.36	1.33E-03	1.69E-02	up
c5411_g1	0.565	7.705	3.55	1.33E-03	1.69E-02	up
c18719_g1	45.583	3.426	-3.7	1.33E-03	1.69E-02	down
c13902_g1	12.848	131.917	3.35	1.33E-03	1.69E-02	up
c12306_g1	7.712	0.543	-3.6	1.34E-03	1.69E-02	down
c13766_g1	6.126	0.233	-4.22	1.34E-03	1.69E-02	down
c30229_g1	18.17	189.02	3.37	1.34E-03	1.70E-02	up
c14173_g1	58.571	4.486	-3.68	1.34E-03	1.70E-02	down
c4005_g1	10.953	113.404	3.36	1.35E-03	1.70E-02	up
c30214_g1	33.029	2.611	-3.61	1.35E-03	1.70E-02	down
c13069_g2	1.972	15.888	2.95	1.35E-03	1.70E-02	up
c10303_g1	1.47	20.025	3.68	1.35E-03	1.70E-02	up
c30912_g1	10.806	110.624	3.34	1.35E-03	1.70E-02	up
c1748_g1	3.829	40.607	3.37	1.35E-03	1.70E-02	up
c35654_g1	55.747	574.221	3.36	1.36E-03	1.71E-02	up
c6483_g1	25.139	1.357	-4.11	1.37E-03	1.72E-02	down
c11941_g2	5.77	0.116	-4.76	1.37E-03	1.73E-02	down
c9483_g1	5.825	0.142	-4.61	1.37E-03	1.73E-02	down
c35488_g1	4.935	0.116	-4.54	1.37E-03	1.73E-02	down
c1797_g2	508.959	41.382	-3.62	1.38E-03	1.73E-02	down
c11435_g1	31.436	1.784	-4.07	1.38E-03	1.73E-02	down
c7320_g1	23.213	1.357	-4	1.38E-03	1.73E-02	down
c1249_g1	0.425	7.369	3.83	1.39E-03	1.74E-02	up
c11477_g1	6.938	0.375	-3.89	1.39E-03	1.74E-02	down
c14057_g1	0.139	5.908	4.65	1.40E-03	1.74E-02	up
c13101_g2	0.139	5.882	4.65	1.40E-03	1.74E-02	up
c2627_g1	0.193	8.17	4.82	1.40E-03	1.74E-02	up
c24929_g1	0.209	9.037	4.89	1.40E-03	1.74E-02	up
c16402_g1	5.732	0.181	-4.38	1.40E-03	1.74E-02	down
c6713_g1	0.433	10.032	4.25	1.40E-03	1.74E-02	up
c9958_g1	0.596	12.682	4.2	1.40E-03	1.74E-02	up
c6997_g1	18.572	1.293	-3.74	1.40E-03	1.74E-02	down
c3209_g1	41.662	3.271	-3.63	1.40E-03	1.75E-02	down

seq_id	Fpkm of flower	Fpkm of leaf	log2FC (leaf/flower)	p-value	FDR	regulate
c12295_g1	16.081	1.254	-3.58	1.40E-03	1.75E-02	down
c18105_g1	3.821	42.132	3.43	1.41E-03	1.75E-02	up
c12588_g1	1492.464	117.67	-3.66	1.41E-03	1.75E-02	down
c2993_g1	1.284	14.725	3.42	1.41E-03	1.75E-02	up
c1426_g1	239.76	2417.915	3.33	1.42E-03	1.76E-02	up
c4234_g1	9.646	0.517	-3.98	1.42E-03	1.76E-02	down
c13007_g2	0.356	6.141	3.77	1.42E-03	1.76E-02	up
c6859_g1	5.152	53.561	3.35	1.42E-03	1.76E-02	up
c34441_g1	204.062	16.677	-3.61	1.43E-03	1.77E-02	down
c8452_g1	5.206	53.018	3.32	1.44E-03	1.78E-02	up
c6786_g3	90.942	7.24	-3.63	1.45E-03	1.79E-02	down
c8147_g1	12.384	124.884	3.32	1.45E-03	1.79E-02	up
c13331_g1	25.132	252.935	3.33	1.45E-03	1.79E-02	up
c703_g1	17.172	172.717	3.32	1.46E-03	1.80E-02	up
c11344_g1	20.344	1.138	-4.05	1.46E-03	1.80E-02	down
c13759_g1	5.136	54.103	3.37	1.46E-03	1.80E-02	up
c381_g1	7.147	75.448	3.38	1.47E-03	1.81E-02	up
c8431_g1	1.16	13.07	3.39	1.47E-03	1.81E-02	up
c12613_g1	6.923	0.103	-5.11	1.48E-03	1.82E-02	down
c7938_g1	5.206	0.129	-4.53	1.48E-03	1.82E-02	down
c18842_g1	0.309	5.662	3.82	1.48E-03	1.82E-02	up
c24924_g1	0.302	5.559	3.82	1.48E-03	1.82E-02	up
c21967_g1	719.921	59.934	-3.58	1.49E-03	1.82E-02	down
c21843_g1	25.588	256.62	3.32	1.50E-03	1.82E-02	up
c27429_g1	75.743	6.154	-3.6	1.50E-03	1.82E-02	down
c16184_g1	0	4.964	5.66	1.52E-03	1.82E-02	up
c24825_g1	0	5.753	5.87	1.52E-03	1.82E-02	up
c37341_g1	0	3.775	5.28	1.52E-03	1.82E-02	up
c16840_g1	0	6.167	5.97	1.52E-03	1.82E-02	up
c27838_g1	0	7.059	6.16	1.52E-03	1.82E-02	up
c1373_g2	0	3.788	5.28	1.52E-03	1.82E-02	up
c35744_g1	0	4.719	5.59	1.52E-03	1.82E-02	up
c3427_g1	0	5.275	5.75	1.52E-03	1.82E-02	up
c11492_g1	0	9.14	6.53	1.52E-03	1.82E-02	up
c6736_g1	38.359	0	-8.59	1.52E-03	1.82E-02	down
c12466_g1	7.031	0	-6.16	1.52E-03	1.82E-02	down
c415_g1	5.105	0	-5.7	1.52E-03	1.82E-02	down
c7117_g1	2.614	0	-4.76	1.52E-03	1.82E-02	down
c11854_g2	4.394	0	-5.49	1.52E-03	1.82E-02	down
c11806_g1	6.111	0	-5.96	1.52E-03	1.82E-02	down
c22426_g1	2.568	0	-4.74	1.52E-03	1.82E-02	down
c7621_g1	8.485	0	-6.42	1.52E-03	1.82E-02	down

seq_id	Fpkm of flower	Fpkm of leaf	log2FC (leaf/flower)	p-value	FDR	regulate
c11858_g1	9.8	0	-6.63	1.52E-03	1.82E-02	down
c4619_g1	3.388	0	-5.12	1.52E-03	1.82E-02	down
c13145_g2	7.047	0	-6.16	1.52E-03	1.82E-02	down
c8748_g1	3.837	0	-5.3	1.52E-03	1.82E-02	down
c12344_g1	8.617	0	-6.45	1.52E-03	1.82E-02	down
c15508_g1	7.403	0	-6.23	1.52E-03	1.82E-02	down
c32850_g1	42.799	0	-8.74	1.52E-03	1.82E-02	down
c1359_g1	7.201	0	-6.19	1.52E-03	1.82E-02	down
c18_g1	4.471	0	-5.51	1.52E-03	1.82E-02	down
c1486_g1	14.96	0	-7.23	1.52E-03	1.82E-02	down
c37855_g1	8.772	0	-6.47	1.52E-03	1.82E-02	down
c19345_g1	0.325	5.52	3.73	1.52E-03	1.82E-02	up
c10397_g1	0.309	5.249	3.71	1.52E-03	1.82E-02	up
c24539_g1	0.534	9.062	3.85	1.52E-03	1.82E-02	up
c16045_g1	1.153	13.794	3.47	1.52E-03	1.82E-02	up
c11523_g1	1.153	13.755	3.47	1.52E-03	1.82E-02	up
c5786_g2	20.181	202.516	3.32	1.52E-03	1.82E-02	up
c4278_g1	1.416	16.742	3.47	1.52E-03	1.82E-02	up
c10910_g1	12.438	0.608	-4.15	1.53E-03	1.83E-02	down
c4943_g1	7.619	0.427	-3.87	1.53E-03	1.83E-02	down
c29982_g1	1.74	23.296	3.67	1.53E-03	1.83E-02	up
c8152_g1	17.087	1.151	-3.78	1.54E-03	1.84E-02	down
c34253_g1	0.286	11.816	4.95	1.55E-03	1.84E-02	up
c12400_g1	0.155	6.244	4.64	1.55E-03	1.84E-02	up
c24554_g1	0.232	9.644	4.88	1.55E-03	1.84E-02	up
c35610_g1	6.451	0.207	-4.42	1.55E-03	1.84E-02	down
c36743_g1	7.201	0.233	-4.45	1.55E-03	1.84E-02	down
c22612_g1	5.825	0.181	-4.4	1.55E-03	1.84E-02	down
c4504_g1	0.294	5.999	3.95	1.55E-03	1.84E-02	up
c4042_g1	31.776	2.456	-3.64	1.56E-03	1.86E-02	down
c1752_g1	216.307	18.022	-3.58	1.57E-03	1.86E-02	down
c5779_g1	1.3	14.272	3.36	1.57E-03	1.86E-02	up
c18106_g1	126.207	10.459	-3.58	1.57E-03	1.86E-02	down
c26693_g1	3.001	33.729	3.45	1.58E-03	1.87E-02	up
c30660_g1	5.012	50.018	3.29	1.59E-03	1.88E-02	up
c9597_g1	233.03	19.353	-3.58	1.59E-03	1.89E-02	down
c12624_g1	3.79	37.594	3.28	1.60E-03	1.89E-02	up
c9240_g1	11.525	0.284	-4.92	1.61E-03	1.90E-02	down
c957_g1	0.572	10.239	3.94	1.61E-03	1.90E-02	up
c6196_g1	16.352	0.814	-4.17	1.61E-03	1.90E-02	down
c10133_g1	8.834	0.349	-4.31	1.62E-03	1.90E-02	down
c10319_g2	6.637	0.259	-4.23	1.62E-03	1.90E-02	down

seq_id	Fpkm of flower	Fpkm of leaf	log2FC (leaf/flower)	p-value	FDR	regulate
c20174_g1	10.079	0.401	-4.34	1.62E-03	1.90E-02	down
c18154_g1	4.765	0.194	-4.05	1.62E-03	1.90E-02	down
c9011_g1	12.856	126.151	3.28	1.62E-03	1.91E-02	up
c34551_g1	92.072	893.502	3.28	1.62E-03	1.91E-02	up
c30071_g1	36.897	2.288	-3.95	1.63E-03	1.92E-02	down
c936_g1	7.232	71.595	3.29	1.63E-03	1.92E-02	up
c22268_g1	172.255	14.518	-3.56	1.64E-03	1.93E-02	down
c7147_g1	3.597	37.504	3.35	1.64E-03	1.93E-02	up
c11427_g2	25.619	2.043	-3.59	1.65E-03	1.93E-02	down
c8706_g2	26.176	1.487	-4.05	1.65E-03	1.93E-02	down
c6111_g1	0.828	10.575	3.52	1.65E-03	1.93E-02	up
c27423_g1	2.127	27.524	3.63	1.65E-03	1.93E-02	up
c12956_g1	19.887	1.616	-3.54	1.65E-03	1.93E-02	down
c4617_g1	5.886	61.343	3.36	1.65E-03	1.93E-02	up
c18449_g1	43.526	424.722	3.28	1.65E-03	1.93E-02	up
c17721_g1	4.409	43.438	3.27	1.66E-03	1.94E-02	up
c30191_g1	54.301	524.952	3.27	1.66E-03	1.94E-02	up
c23301_g1	57.712	4.279	-3.72	1.67E-03	1.94E-02	down
c5989_g1	2.823	30.458	3.39	1.68E-03	1.96E-02	up
c12894_g2	0.449	6.71	3.63	1.68E-03	1.96E-02	up
c26270_g1	1.16	14.996	3.58	1.68E-03	1.96E-02	up
c22223_g1	32.325	2.547	-3.61	1.69E-03	1.96E-02	down
c6435_g1	5.987	59.184	3.28	1.69E-03	1.96E-02	up
c37484_g1	5.159	52.229	3.31	1.70E-03	1.97E-02	up
c35009_g1	270.747	2603.069	3.26	1.70E-03	1.97E-02	up
c13260_g1	17.984	173.506	3.26	1.70E-03	1.97E-02	up
c11433_g1	12.709	0.866	-3.73	1.70E-03	1.97E-02	down
c31897_g1	0.866	13.445	3.81	1.70E-03	1.97E-02	up
c5497_g1	11.355	109.513	3.26	1.71E-03	1.98E-02	up
c26402_g1	6.908	0.388	-3.84	1.71E-03	1.98E-02	down
c11164_g1	19.972	0.724	-4.61	1.71E-03	1.98E-02	down
c17728_g1	2.081	21.628	3.32	1.71E-03	1.98E-02	up
c27556_g1	4.587	0.155	-4.2	1.72E-03	1.98E-02	down
c8996_g1	13.521	1.086	-3.52	1.72E-03	1.98E-02	down
c30785_g1	6.969	0.452	-3.68	1.72E-03	1.99E-02	down
c7668_g1	28.953	277.86	3.26	1.73E-03	2.00E-02	up
c22326_g1	1.191	15.113	3.56	1.74E-03	2.00E-02	up
c22512_g1	5.438	0.129	-4.6	1.74E-03	2.00E-02	down
c24771_g1	14.256	0.349	-5	1.74E-03	2.00E-02	down
c10655_g1	1.446	14.699	3.26	1.75E-03	2.01E-02	up
c409_g1	77.476	6.464	-3.56	1.75E-03	2.01E-02	down
c12891_g1	19.129	1.461	-3.62	1.75E-03	2.01E-02	down

seq_id	Fpkm of flower	Fpkm of leaf	log2FC (leaf/flower)	p-value	FDR	regulate
c4334_g1	10.063	0.737	-3.6	1.75E-03	2.01E-02	down
c9060_g1	3.063	30.588	3.28	1.76E-03	2.01E-02	up
c12889_g1	4.734	45.739	3.25	1.76E-03	2.01E-02	up
c30138_g1	6.683	0	-6.08	1.76E-03	2.01E-02	down
c5319_g1	5.175	0	-5.72	1.76E-03	2.01E-02	down
c6786_g2	5.237	0	-5.74	1.76E-03	2.01E-02	down
c7964_g1	4.324	0	-5.47	1.76E-03	2.01E-02	down
c20369_g1	7.201	0	-6.19	1.76E-03	2.01E-02	down
c11018_g1	4.997	0	-5.67	1.76E-03	2.01E-02	down
c31515_g1	15.865	0	-7.32	1.76E-03	2.01E-02	down
c1427_g1	13.397	0.776	-3.95	1.77E-03	2.01E-02	down
c7682_g1	9.143	0.582	-3.76	1.77E-03	2.01E-02	down
c4964_g1	0.789	10.187	3.53	1.78E-03	2.02E-02	up
c5266_g1	1.733	21.848	3.58	1.78E-03	2.02E-02	up
c14381_g1	1.532	19.276	3.57	1.78E-03	2.02E-02	up
c26434_g1	15.053	1.099	-3.66	1.78E-03	2.02E-02	down
c1908_g1	12.593	0.84	-3.76	1.78E-03	2.02E-02	down
c9344_g1	32.263	2.34	-3.73	1.78E-03	2.02E-02	down
c12278_g1	36.471	346.935	3.25	1.78E-03	2.02E-02	up
c5692_g1	20.993	1.202	-4.02	1.79E-03	2.03E-02	down
c13552_g1	27.004	2.262	-3.52	1.80E-03	2.04E-02	down
c11954_g1	18.897	1.487	-3.58	1.81E-03	2.06E-02	down
c11804_g2	1.238	14.04	3.4	1.83E-03	2.07E-02	up
c6218_g1	36.301	345.784	3.25	1.83E-03	2.08E-02	up
c6312_g1	7.209	70.871	3.28	1.84E-03	2.09E-02	up
c10287_g2	19.988	1.5	-3.65	1.85E-03	2.09E-02	down
c36562_g1	11.788	119.958	3.34	1.85E-03	2.09E-02	up
c9165_g2	0.681	9.942	3.68	1.85E-03	2.09E-02	up
c10496_g1	10.466	0.621	-3.87	1.85E-03	2.09E-02	down
c9236_g1	0.565	6.981	3.41	1.85E-03	2.09E-02	up
c18103_g1	4.742	45.597	3.24	1.86E-03	2.09E-02	up
c28628_g1	3.55	40.633	3.48	1.86E-03	2.10E-02	up
c10644_g1	20.243	186.331	3.2	1.86E-03	2.10E-02	up
c12200_g2	12.693	1.034	-3.5	1.86E-03	2.10E-02	down
c18159_g1	10.914	0.802	-3.61	1.88E-03	2.11E-02	down
c18081_g1	6.474	0.168	-4.62	1.89E-03	2.12E-02	down
c31952_g1	18.216	0.478	-4.99	1.89E-03	2.12E-02	down
c14366_g1	0.41	7.162	3.83	1.89E-03	2.12E-02	up
c8299_g1	0.24	4.215	3.67	1.89E-03	2.12E-02	up
c8112_g2	0.766	13.264	3.95	1.89E-03	2.12E-02	up
c13765_g1	34.978	2.896	-3.55	1.89E-03	2.12E-02	down
c5377_g2	36.742	2.857	-3.64	1.90E-03	2.12E-02	down

seq_id	Fpkm of flower	Fpkm of leaf	log2FC (leaf/flower)	p-value	FDR	regulate
c12208_g1	41.932	3.09	-3.72	1.90E-03	2.12E-02	down
c2560_g1	9.011	0.478	-3.98	1.91E-03	2.12E-02	down
c278_g1	28.465	2.366	-3.53	1.91E-03	2.12E-02	down
c22559_g1	28.303	264.752	3.22	1.91E-03	2.12E-02	up
c38179_g1	0.309	12.398	4.93	1.91E-03	2.12E-02	up
c19527_g1	0.186	7.24	4.68	1.91E-03	2.12E-02	up
c6170_g1	0.263	10.459	4.86	1.91E-03	2.12E-02	up
c26012_g1	0.178	7.046	4.68	1.91E-03	2.12E-02	up
c15098_g1	4.355	0.142	-4.2	1.91E-03	2.12E-02	down
c33230_g1	3.86	0.129	-4.11	1.91E-03	2.12E-02	down
c37793_g1	0.263	5.132	3.85	1.91E-03	2.12E-02	up
c9862_g1	0.58	6.942	3.37	1.91E-03	2.12E-02	up
c6417_g1	0.34	6.671	3.94	1.91E-03	2.12E-02	up
c18712_g1	0.356	7.097	3.98	1.91E-03	2.12E-02	up
c8969_g2	17.42	1.319	-3.63	1.92E-03	2.13E-02	down
c10899_g1	15.238	1.28	-3.47	1.92E-03	2.13E-02	down
c9010_g1	12.686	0.931	-3.63	1.93E-03	2.14E-02	down
c3678_g1	8.841	0.685	-3.51	1.94E-03	2.15E-02	down
c35476_g1	115.78	9.916	-3.53	1.94E-03	2.15E-02	down
c11854_g1	23.917	1.926	-3.57	1.94E-03	2.15E-02	down
c10886_g3	6.42	64.252	3.3	1.95E-03	2.15E-02	up
c8453_g1	133.416	11.803	-3.49	1.95E-03	2.16E-02	down
c27268_g1	26.199	244.119	3.22	1.97E-03	2.17E-02	up
c19453_g1	0.425	6.723	3.7	1.97E-03	2.17E-02	up
c6525_g1	0.549	8.688	3.76	1.97E-03	2.17E-02	up
c14812_g1	1.624	18.164	3.41	1.97E-03	2.17E-02	up
c3237_g1	10.033	93.534	3.21	1.98E-03	2.18E-02	up
c7764_g1	17.458	1.267	-3.68	1.98E-03	2.18E-02	down
c18137_g1	18.007	167.133	3.21	1.99E-03	2.19E-02	up
c2488_g1	0.634	7.679	3.41	2.00E-03	2.20E-02	up
c11394_g1	9.22	0.737	-3.48	2.00E-03	2.20E-02	down
c12424_g2	2.119	19.961	3.18	2.01E-03	2.21E-02	up
c8706_g1	15.045	1.164	-3.58	2.01E-03	2.21E-02	down
c11628_g1	21.867	1.577	-3.71	2.02E-03	2.21E-02	down
c37046_g1	8.594	0.452	-3.98	2.02E-03	2.21E-02	down
c13507_g1	26.609	2.301	-3.48	2.03E-03	2.23E-02	down
c17853_g1	81.916	749.95	3.19	2.04E-03	2.23E-02	up
c2679_g1	0.487	7.977	3.78	2.05E-03	2.24E-02	up
c35213_g1	1.23	15.126	3.52	2.05E-03	2.24E-02	up
c6601_g1	0.248	5.107	3.9	2.05E-03	2.24E-02	up
c19017_g1	1.663	28.894	4.04	2.05E-03	2.24E-02	up
c11696_g1	13.475	1.06	-3.55	2.06E-03	2.24E-02	down

seq_id	Fpkm of flower	Fpkm of leaf	log2FC (leaf/flower)	p-value	FDR	regulate
c9970_g1	19.763	1.655	-3.5	2.06E-03	2.24E-02	down
c34560_g2	39.844	0	-8.64	2.06E-03	2.24E-02	down
c26235_g1	4.2	0	-5.43	2.06E-03	2.24E-02	down
c8742_g1	4.657	0	-5.57	2.06E-03	2.24E-02	down
c9458_g1	4.223	0	-5.43	2.06E-03	2.24E-02	down
c16083_g1	7.248	0	-6.2	2.06E-03	2.24E-02	down
c2727_g1	3.017	0	-4.96	2.06E-03	2.24E-02	down
c4083_g1	18.093	165.09	3.18	2.08E-03	2.26E-02	up
c2913_g1	46.867	4.163	-3.46	2.08E-03	2.26E-02	down
c31058_g1	0.371	4.396	3.25	2.08E-03	2.26E-02	up
c30966_g1	58.285	4.124	-3.79	2.09E-03	2.27E-02	down
c8179_g1	1.965	30.445	3.89	2.09E-03	2.27E-02	up
c7264_g1	1.524	16.354	3.34	2.10E-03	2.27E-02	up
c13049_g2	2.854	20.129	2.78	2.10E-03	2.27E-02	up
c12745_g1	29.943	275.689	3.2	2.10E-03	2.27E-02	up
c27433_g1	5.554	54.453	3.27	2.10E-03	2.28E-02	up
c21665_g1	33.261	2.78	-3.53	2.10E-03	2.28E-02	down
c26208_g1	49.381	2.159	-4.45	2.11E-03	2.28E-02	down
c18116_g1	4.455	0.194	-3.95	2.11E-03	2.28E-02	down
c7907_g1	8.563	0.362	-4.23	2.11E-03	2.28E-02	down
c999_g1	0.193	7.395	4.68	2.13E-03	2.29E-02	up
c28397_g1	0.155	5.882	4.55	2.13E-03	2.29E-02	up
c30821_g1	2.994	0.103	-3.93	2.13E-03	2.29E-02	down
c16110_g1	9.816	0.336	-4.51	2.13E-03	2.29E-02	down
c24329_g1	7.101	0.246	-4.38	2.13E-03	2.29E-02	down
c3454_g1	14.929	0.53	-4.58	2.13E-03	2.29E-02	down
c4983_g1	1.972	39.404	4.25	2.13E-03	2.29E-02	up
c11647_g1	0.325	6.477	3.95	2.13E-03	2.29E-02	up
c3800_g1	3.527	34.893	3.27	2.13E-03	2.29E-02	up
c13290_g1	9.166	85.518	3.21	2.13E-03	2.29E-02	up
c6079_g1	6.235	58.913	3.22	2.13E-03	2.29E-02	up
c10836_g1	2.661	27.718	3.33	2.13E-03	2.29E-02	up
c21789_g1	424.878	38.706	-3.45	2.14E-03	2.29E-02	down
c26281_g1	158.447	14.376	-3.45	2.15E-03	2.30E-02	down
c2167_g1	0.65	9.153	3.62	2.15E-03	2.30E-02	up
c2193_g1	0.418	5.908	3.54	2.15E-03	2.30E-02	up
c11711_g1	0.998	11.997	3.46	2.16E-03	2.31E-02	up
c11400_g1	0.603	7.472	3.43	2.16E-03	2.31E-02	up
c5712_g1	1.021	10.885	3.29	2.16E-03	2.31E-02	up
c3339_g1	0.627	7.408	3.37	2.16E-03	2.31E-02	up
c26962_g1	20.645	1.706	-3.52	2.17E-03	2.32E-02	down
c36225_g1	25.944	235.366	3.18	2.18E-03	2.32E-02	up

seq_id	Fpkm of flower	Fpkm of leaf	log2FC (leaf/flower)	p-value	FDR	regulate
c10266_g2	13.869	149.408	3.42	2.20E-03	2.34E-02	up
c1617_g1	10.412	94.413	3.17	2.21E-03	2.35E-02	up
c14401_g1	29.456	2.599	-3.45	2.21E-03	2.35E-02	down
c30490_g1	12.229	110.883	3.17	2.21E-03	2.36E-02	up
c1834_g1	213.816	1923.666	3.17	2.22E-03	2.36E-02	up
c3680_g1	19.384	176.389	3.18	2.22E-03	2.36E-02	up
c1135_g1	26.787	0.724	-5.03	2.23E-03	2.37E-02	down
c5303_g1	5.477	0.142	-4.53	2.23E-03	2.37E-02	down
c5130_g1	21.829	196.531	3.16	2.23E-03	2.37E-02	up
c23048_g1	6.861	63.36	3.19	2.23E-03	2.37E-02	up
c814_g1	0.913	10.653	3.41	2.24E-03	2.37E-02	up
c12484_g1	25.379	2.262	-3.43	2.25E-03	2.38E-02	down
c3001_g1	59.182	2.288	-4.63	2.26E-03	2.39E-02	down
c10954_g1	2.328	23.955	3.31	2.26E-03	2.39E-02	up
c8599_g2	1.261	18.319	3.76	2.27E-03	2.40E-02	up
c34682_g1	4.44	66.695	3.88	2.27E-03	2.40E-02	up
c1440_g1	139.458	12.812	-3.43	2.30E-03	2.43E-02	down
c11100_g1	11.618	103.915	3.15	2.30E-03	2.43E-02	up
c5532_g1	0.982	9.916	3.21	2.32E-03	2.45E-02	up
c9826_g1	2.119	19.676	3.16	2.32E-03	2.45E-02	up
c8874_g1	71.991	6.063	-3.55	2.32E-03	2.45E-02	down
c12701_g1	26.501	2.198	-3.53	2.33E-03	2.46E-02	down
c9313_g1	25.426	2.249	-3.44	2.34E-03	2.46E-02	down
c13081_g2	45.908	3.969	-3.5	2.34E-03	2.46E-02	down
c4898_g1	3.094	29.14	3.19	2.34E-03	2.46E-02	up
c27009_g1	0.866	8.739	3.19	2.35E-03	2.47E-02	up
c1676_g1	38.475	3.387	-3.47	2.35E-03	2.47E-02	down
c30862_g1	375.427	35.074	-3.42	2.36E-03	2.48E-02	down
c1861_g1	257.21	23.891	-3.42	2.37E-03	2.48E-02	down
c14805_g1	0.248	9.114	4.73	2.38E-03	2.48E-02	up
c4931_g1	0.131	4.938	4.45	2.38E-03	2.48E-02	up
c26669_g1	0.201	7.537	4.67	2.38E-03	2.48E-02	up
c2514_g1	6.807	0.246	-4.32	2.38E-03	2.48E-02	down
c11090_g1	2.777	0.103	-3.83	2.38E-03	2.48E-02	down
c16562_g1	15.579	0.569	-4.55	2.38E-03	2.48E-02	down
c60_g1	12.956	0.375	-4.78	2.38E-03	2.48E-02	down
c9677_g1	6.397	0.233	-4.29	2.38E-03	2.48E-02	down
c8305_g2	0.224	4.111	3.7	2.38E-03	2.48E-02	up
c706_g1	0.302	5.585	3.82	2.38E-03	2.48E-02	up
c3892_g1	0.193	3.542	3.64	2.38E-03	2.48E-02	up
c31241_g1	240.541	22.249	-3.43	2.38E-03	2.48E-02	down
c22066_g1	108.169	958.944	3.15	2.39E-03	2.48E-02	up

seq_id	Fpkm of flower	Fpkm of leaf	log2FC (leaf/flower)	p-value	FDR	regulate
c22731_g1	0.511	5.869	3.29	2.39E-03	2.48E-02	up
c7400_g1	5.214	47.019	3.15	2.39E-03	2.48E-02	up
c11401_g1	20.15	1.112	-4.06	2.40E-03	2.50E-02	down
c12192_g2	8.246	0.349	-4.22	2.40E-03	2.50E-02	down
c10884_g1	17.768	1.525	-3.46	2.41E-03	2.50E-02	down
c15003_g1	440.913	41.434	-3.41	2.41E-03	2.51E-02	down
c12076_g1	0.688	8.726	3.49	2.42E-03	2.51E-02	up
c38088_g1	5.43	0	-5.79	2.43E-03	2.51E-02	down
c10455_g2	10.218	0	-6.69	2.43E-03	2.51E-02	down
c8246_g1	4.076	0	-5.38	2.43E-03	2.51E-02	down
c7657_g1	8.207	0	-6.38	2.43E-03	2.51E-02	down
c26577_g1	2.336	0	-4.61	2.43E-03	2.51E-02	down
c4605_g1	3.628	0	-5.22	2.43E-03	2.51E-02	down
c35537_g1	9.143	0	-6.53	2.43E-03	2.51E-02	down
c15087_g1	11.046	0.297	-4.81	2.43E-03	2.51E-02	down
c13246_g1	70.939	619.934	3.13	2.43E-03	2.51E-02	up
c11006_g1	3.465	32.462	3.19	2.44E-03	2.51E-02	up
c10383_g2	14.225	125.22	3.13	2.44E-03	2.52E-02	up
c36064_g1	1.253	12.152	3.18	2.45E-03	2.52E-02	up
c13156_g2	4.981	33.432	2.72	2.45E-03	2.52E-02	up
c17899_g1	46.891	4.111	-3.48	2.45E-03	2.52E-02	down
c7912_g1	2.622	24.201	3.16	2.45E-03	2.53E-02	up
c30135_g1	63.22	554.57	3.13	2.46E-03	2.53E-02	up
c19962_g1	2.653	25.662	3.23	2.46E-03	2.53E-02	up
c30153_g1	157.999	1376.504	3.12	2.47E-03	2.53E-02	up
c34702_g1	9.46	82.997	3.12	2.47E-03	2.53E-02	up
c1253_g1	8.872	78.718	3.14	2.47E-03	2.53E-02	up
c34727_g1	107.79	10.006	-3.42	2.47E-03	2.53E-02	down
c1203_g1	6.505	0.465	-3.55	2.47E-03	2.53E-02	down
c31498_g1	0.572	6.645	3.33	2.47E-03	2.53E-02	up
c6185_g1	59.7	5.481	-3.42	2.50E-03	2.56E-02	down
c23993_g1	20.429	1.396	-3.78	2.50E-03	2.56E-02	down
c22330_g1	4.927	44.395	3.15	2.50E-03	2.56E-02	up
c18306_g1	6.606	64.187	3.26	2.52E-03	2.57E-02	up
c15537_g1	6.265	0.478	-3.46	2.52E-03	2.57E-02	down
c6677_g1	20.274	1.745	-3.47	2.52E-03	2.58E-02	down
c3146_g1	19.121	168.244	3.13	2.53E-03	2.58E-02	up
c30223_g1	24.667	214.1	3.11	2.54E-03	2.59E-02	up
c6305_g1	15.656	1.189	-3.61	2.54E-03	2.59E-02	down
c23165_g1	4.061	37.207	3.16	2.55E-03	2.60E-02	up
c35464_g1	1.354	13.975	3.28	2.56E-03	2.60E-02	up
c9368_g1	1.044	9.786	3.11	2.56E-03	2.61E-02	up

seq_id	Fpkm of flower	Fpkm of leaf	log2FC (leaf/flower)	p-value	FDR	regulate
c8405_g1	1.849	18.901	3.29	2.56E-03	2.61E-02	up
c36550_g1	4.951	48.506	3.27	2.57E-03	2.61E-02	up
c21936_g1	116.507	10.911	-3.4	2.60E-03	2.64E-02	down
c3539_g1	12.059	105.983	3.13	2.60E-03	2.64E-02	up
c10508_g1	3.922	0.181	-3.84	2.60E-03	2.64E-02	down
c4389_g1	50.789	2.366	-4.37	2.60E-03	2.64E-02	down
c8585_g1	23.585	1.9	-3.57	2.61E-03	2.65E-02	down
c9108_g1	8.091	0.672	-3.41	2.61E-03	2.65E-02	down
c4244_g1	3.04	27.899	3.16	2.62E-03	2.66E-02	up
c3156_g1	24.49	2.198	-3.42	2.63E-03	2.66E-02	down
c7565_g1	18.998	1.474	-3.6	2.63E-03	2.66E-02	down
c12972_g5	1.284	12.23	3.16	2.64E-03	2.67E-02	up
c96_g1	0.89	9.024	3.2	2.65E-03	2.67E-02	up
c8184_g1	0.518	5.275	3.12	2.65E-03	2.67E-02	up
c838_g1	0.449	7.434	3.78	2.65E-03	2.67E-02	up
c37427_g1	0.286	4.473	3.57	2.65E-03	2.67E-02	up
c9494_g1	0.418	6.438	3.66	2.65E-03	2.67E-02	up
c489_g1	10.404	90.198	3.1	2.66E-03	2.67E-02	up
c3435_g1	6.544	57.542	3.12	2.66E-03	2.67E-02	up
c12730_g1	15.811	1.267	-3.54	2.66E-03	2.67E-02	down
c5422_g1	0.24	14.027	5.38	2.66E-03	2.67E-02	up
c6890_g2	0.255	8.972	4.68	2.66E-03	2.67E-02	up
c15776_g1	0.232	8.093	4.63	2.66E-03	2.67E-02	up
c14041_g1	0.131	4.719	4.38	2.66E-03	2.67E-02	up
c1952_g1	5.84	0.168	-4.47	2.66E-03	2.67E-02	down
c12647_g1	4.231	0.155	-4.09	2.66E-03	2.67E-02	down
c19176_g1	10.234	0.388	-4.4	2.66E-03	2.67E-02	down
c22358_g1	3.69	0.142	-3.97	2.66E-03	2.67E-02	down
c21563_g1	1.423	25.921	4.09	2.66E-03	2.67E-02	up
c26024_g1	599.615	5133.496	3.1	2.66E-03	2.67E-02	up
c9863_g1	4.44	39.857	3.14	2.67E-03	2.67E-02	up
c13476_g1	238.042	22.818	-3.38	2.68E-03	2.68E-02	down
c30062_g1	13.475	116.248	3.1	2.68E-03	2.68E-02	up
c7663_g1	22.749	2.043	-3.41	2.69E-03	2.68E-02	down
c6374_g1	46.256	4.279	-3.4	2.69E-03	2.68E-02	down
c35166_g1	50.542	4.796	-3.37	2.69E-03	2.69E-02	down
c34468_g1	26.702	227.648	3.09	2.71E-03	2.70E-02	up
c13947_g1	322.441	31.26	-3.36	2.72E-03	2.71E-02	down
c1010_g1	1.439	13.523	3.15	2.75E-03	2.74E-02	up
c6846_g2	728.777	71.143	-3.35	2.75E-03	2.74E-02	down
c3595_g1	11.03	0.543	-4.11	2.76E-03	2.75E-02	down
c8257_g2	39.403	336.178	3.09	2.77E-03	2.76E-02	up

seq_id	Fpkm of flower	Fpkm of leaf	log2FC (leaf/flower)	p-value	FDR	regulate
c11946_g1	14.697	0.983	-3.77	2.79E-03	2.77E-02	down
c862_g1	1.663	18.914	3.43	2.79E-03	2.77E-02	up
c1300_g1	49.613	4.732	-3.36	2.79E-03	2.77E-02	down
c11856_g1	12.701	1.164	-3.34	2.80E-03	2.78E-02	down
c35568_g1	6.436	0.297	-4.04	2.80E-03	2.78E-02	down
c12823_g1	0.743	10.769	3.69	2.80E-03	2.78E-02	up
c11971_g1	9.831	0.789	-3.48	2.80E-03	2.78E-02	down
c26687_g1	49.884	425.873	3.09	2.80E-03	2.78E-02	up
c8411_g2	20.258	1.396	-3.77	2.80E-03	2.78E-02	down
c2655_g1	13.335	0.944	-3.69	2.80E-03	2.78E-02	down
c12738_g3	2.661	24.24	3.14	2.81E-03	2.78E-02	up
c1790_g1	28.574	2.741	-3.34	2.81E-03	2.78E-02	down
c10529_g1	15.207	1.345	-3.41	2.81E-03	2.78E-02	down
c17747_g1	32.72	276.994	3.08	2.87E-03	2.81E-02	up
c26172_g1	142.467	1198.02	3.07	2.87E-03	2.81E-02	up
c14100_g1	3.179	0	-5.04	2.87E-03	2.81E-02	down
c3077_g1	6.235	0	-5.99	2.87E-03	2.81E-02	down
c20966_g1	8.671	0	-6.45	2.87E-03	2.81E-02	down
c4861_g1	3.063	0	-4.98	2.87E-03	2.81E-02	down
c12505_g2	12.384	0	-6.96	2.87E-03	2.81E-02	down
c14190_g1	5.763	0	-5.87	2.87E-03	2.81E-02	down
c3986_g1	5.113	0	-5.7	2.87E-03	2.81E-02	down
c98_g1	5.36	0	-5.77	2.87E-03	2.81E-02	down
c12130_g1	3.442	0	-5.15	2.87E-03	2.81E-02	down
c33154_g1	10.497	0	-6.73	2.87E-03	2.81E-02	down
c37538_g1	6.088	0	-5.95	2.87E-03	2.81E-02	down
c582_g2	9.097	0	-6.52	2.87E-03	2.81E-02	down
c20258_g1	4.223	0	-5.43	2.87E-03	2.81E-02	down
c36716_g1	7.449	0	-6.24	2.87E-03	2.81E-02	down
c19050_g1	4.873	0	-5.64	2.87E-03	2.81E-02	down
c22888_g1	6.351	0	-6.01	2.87E-03	2.81E-02	down
c10308_g1	4.455	0	-5.51	2.87E-03	2.81E-02	down
c14807_g1	3.991	0	-5.35	2.87E-03	2.81E-02	down
c15001_g1	3.233	0	-5.06	2.87E-03	2.81E-02	down
c9123_g1	11.703	1.008	-3.41	2.87E-03	2.81E-02	down
c4360_g1	47.192	4.046	-3.51	2.88E-03	2.81E-02	down
c6365_g1	12.268	0.827	-3.74	2.88E-03	2.81E-02	down
c20877_g1	15.509	0.905	-3.96	2.89E-03	2.82E-02	down
c2091_g1	0.75	10.161	3.59	2.89E-03	2.82E-02	up
c10919_g1	6.884	0.194	-4.57	2.90E-03	2.82E-02	down
c23401_g1	9.893	0.284	-4.7	2.90E-03	2.82E-02	down
c35859_g1	37.825	320.031	3.08	2.90E-03	2.83E-02	up

seq_id	Fpkm of flower	Fpkm of leaf	log2FC (leaf/flower)	p-value	FDR	regulate
c3618_g1	2.065	17.905	3.06	2.91E-03	2.83E-02	up
c3491_g1	37.539	310.038	3.04	2.92E-03	2.84E-02	up
c1330_g1	2.8	23.555	3.03	2.93E-03	2.84E-02	up
c34824_g1	50.797	4.926	-3.34	2.94E-03	2.85E-02	down
c2917_g1	12.5	0.944	-3.59	2.94E-03	2.85E-02	down
c9090_g1	12.879	1.008	-3.55	2.94E-03	2.85E-02	down
c27220_g1	7.635	64.317	3.06	2.94E-03	2.85E-02	up
c7962_g1	1.516	10.95	2.77	2.94E-03	2.85E-02	up
c31854_g1	17.683	1.577	-3.41	2.94E-03	2.85E-02	down
c5274_g1	9.545	0.776	-3.46	2.97E-03	2.88E-02	down
c8114_g1	18.247	153.209	3.06	2.98E-03	2.89E-02	up
c3803_g1	6.002	50.329	3.05	2.99E-03	2.89E-02	up
c13175_g2	5.948	0.233	-4.18	2.99E-03	2.89E-02	down
c19536_g1	20.97	0.827	-4.51	2.99E-03	2.89E-02	down
c36287_g1	10.381	0.401	-4.39	2.99E-03	2.89E-02	down
c22063_g1	35.566	3.426	-3.34	2.99E-03	2.89E-02	down
c9510_g1	8.965	73.211	3.02	2.99E-03	2.89E-02	up
c3376_g1	9.584	82.377	3.09	3.01E-03	2.90E-02	up
c8868_g1	12.864	106.19	3.04	3.01E-03	2.90E-02	up
c6805_g1	6.103	50.639	3.03	3.01E-03	2.90E-02	up
c3400_g1	10.133	0.84	-3.44	3.01E-03	2.90E-02	down
c24598_g1	28.334	1.345	-4.3	3.01E-03	2.90E-02	down
c9928_g1	10.907	0.556	-4.07	3.01E-03	2.90E-02	down
c9824_g1	6.08	0.284	-4.01	3.01E-03	2.90E-02	down
c964_g1	40.385	3.465	-3.51	3.04E-03	2.92E-02	down
c34932_g1	0.812	8.726	3.27	3.04E-03	2.92E-02	up
c18850_g1	2.088	20.685	3.25	3.04E-03	2.92E-02	up
c8775_g1	7.109	59.831	3.06	3.06E-03	2.93E-02	up
c9977_g1	44.547	368.977	3.05	3.06E-03	2.93E-02	up
c26312_g1	199.691	19.948	-3.32	3.07E-03	2.94E-02	down
c9728_g1	1.493	18.72	3.56	3.07E-03	2.94E-02	up
c1804_g2	20.846	1.241	-3.97	3.07E-03	2.94E-02	down
c35554_g1	6.366	0.375	-3.77	3.07E-03	2.94E-02	down
c8698_g1	20.831	1.021	-4.22	3.07E-03	2.94E-02	down
c30985_g1	147.409	14.428	-3.34	3.08E-03	2.94E-02	down
c9310_g1	47.301	4.021	-3.52	3.08E-03	2.95E-02	down
c10093_g1	9.251	0.789	-3.39	3.09E-03	2.95E-02	down
c12598_g1	26.88	2.237	-3.53	3.10E-03	2.96E-02	down
c27390_g1	71.759	7.046	-3.33	3.10E-03	2.96E-02	down
c4634_g1	7.751	0.685	-3.32	3.10E-03	2.96E-02	down
c1668_g1	39.689	3.865	-3.33	3.11E-03	2.96E-02	down
c11494_g1	34.197	3.141	-3.4	3.11E-03	2.96E-02	down

seq_id	Fpkm of flower	Fpkm of leaf	log2FC (leaf/flower)	p-value	FDR	regulate
c6716_g1	6.343	53.005	3.04	3.12E-03	2.97E-02	up
c10193_g1	31.583	2.792	-3.45	3.14E-03	2.99E-02	down
c9864_g2	0.588	6.309	3.22	3.14E-03	2.99E-02	up
c35908_g1	1.23	13.174	3.32	3.15E-03	2.99E-02	up
c10755_g1	14.89	122.66	3.03	3.15E-03	2.99E-02	up
c10174_g1	1.423	15.126	3.32	3.15E-03	3.00E-02	up
c3303_g1	17.056	1.228	-3.69	3.16E-03	3.00E-02	down
c11891_g2	2.475	22.559	3.14	3.16E-03	3.00E-02	up
c8690_g2	7.789	66.075	3.07	3.16E-03	3.00E-02	up
c12581_g1	12.825	1.086	-3.45	3.16E-03	3.00E-02	down
c11807_g1	13.985	1.357	-3.27	3.17E-03	3.00E-02	down
c11923_g1	10.071	0.271	-4.78	3.18E-03	3.00E-02	down
c4279_g1	6.157	0.181	-4.48	3.18E-03	3.00E-02	down
c13156_g1	0.456	6.916	3.66	3.18E-03	3.00E-02	up
c13220_g9	1.044	15.876	3.8	3.18E-03	3.00E-02	up
c34987_g1	55.523	453.823	3.03	3.18E-03	3.01E-02	up
c30081_g1	69.911	571.234	3.03	3.20E-03	3.02E-02	up
c7605_g1	14.712	119.881	3.02	3.22E-03	3.04E-02	up
c7769_g1	189.164	18.991	-3.31	3.24E-03	3.06E-02	down
c4860_g1	28.744	233.737	3.02	3.25E-03	3.06E-02	up
c11424_g1	0.657	9.101	3.6	3.25E-03	3.06E-02	up
c6887_g1	7.418	46.424	2.63	3.25E-03	3.06E-02	up
c14353_g1	6.265	51.453	3.02	3.25E-03	3.06E-02	up
c3992_g1	5.685	0.44	-3.42	3.27E-03	3.08E-02	down
c11824_g2	12.098	0.646	-4.03	3.27E-03	3.08E-02	down
c4787_g1	7.085	0.543	-3.48	3.27E-03	3.08E-02	down
c1976_g1	123.221	12.437	-3.3	3.27E-03	3.08E-02	down
c2190_g1	0.572	7.989	3.59	3.28E-03	3.08E-02	up
c30475_g1	104.881	854.913	3.03	3.28E-03	3.08E-02	up
c4245_g1	19.825	164.922	3.05	3.28E-03	3.08E-02	up
c2432_g1	0.534	5.662	3.18	3.28E-03	3.08E-02	up
c4941_g1	46.117	3.258	-3.78	3.28E-03	3.08E-02	down
c5802_g1	3.017	25.029	3.01	3.29E-03	3.09E-02	up
c8056_g1	9.507	78.654	3.04	3.31E-03	3.10E-02	up
c11044_g2	22.115	1.991	-3.41	3.32E-03	3.10E-02	down
c11459_g2	7.31	59.65	3.01	3.33E-03	3.11E-02	up
c9298_g1	0.797	7.395	3.06	3.34E-03	3.12E-02	up
c1799_g1	49.165	4.926	-3.29	3.34E-03	3.12E-02	down
c10909_g1	2.947	26.218	3.11	3.35E-03	3.13E-02	up
c22198_g1	29.703	2.857	-3.33	3.36E-03	3.13E-02	down
c36261_g1	0.155	5.171	4.37	3.37E-03	3.13E-02	up
c16483_g1	0.302	10.226	4.68	3.37E-03	3.13E-02	up

seq_id	Fpkm of flower	Fpkm of leaf	log2FC (leaf/flower)	p-value	FDR	regulate
c15860_g1	0.255	8.584	4.61	3.37E-03	3.13E-02	up
c10036_g3	0.611	21.37	4.92	3.37E-03	3.13E-02	up
c5396_g1	6.142	0.246	-4.17	3.37E-03	3.13E-02	down
c9058_g1	4.409	0.129	-4.3	3.37E-03	3.13E-02	down
c10130_g1	8.346	0.323	-4.32	3.37E-03	3.13E-02	down
c8381_g1	20.769	0.646	-4.81	3.37E-03	3.13E-02	down
c2565_g1	0.503	8.623	3.85	3.37E-03	3.13E-02	up
c35708_g1	0.348	5.843	3.73	3.37E-03	3.13E-02	up
c481_g1	0.402	6.878	3.8	3.37E-03	3.13E-02	up
c19149_g1	0.271	4.564	3.65	3.37E-03	3.13E-02	up
c27535_g1	20.119	1.913	-3.33	3.38E-03	3.13E-02	down
c3055_g1	40.122	3.995	-3.3	3.38E-03	3.14E-02	down
c17812_g1	178.992	1443.457	3.01	3.39E-03	3.14E-02	up
c12845_g1	20.854	1.939	-3.36	3.39E-03	3.14E-02	down
c4202_g1	3.079	25.145	2.99	3.39E-03	3.14E-02	up
c10988_g1	1.253	13.574	3.34	3.39E-03	3.14E-02	up
c13003_g4	16.468	1.59	-3.29	3.40E-03	3.14E-02	down
c11315_g3	16.894	1.668	-3.26	3.40E-03	3.14E-02	down
c14194_g1	10.179	82.558	3.01	3.40E-03	3.14E-02	up
c8505_g1	23.492	2.301	-3.3	3.41E-03	3.14E-02	down
c10420_g2	4.192	0.31	-3.39	3.41E-03	3.14E-02	down
c9910_g2	3.094	28.855	3.18	3.42E-03	3.14E-02	up
c9592_g1	17.822	1.719	-3.3	3.42E-03	3.14E-02	down
c2416_g1	9.151	0	-6.53	3.42E-03	3.14E-02	down
c5807_g1	4.247	0	-5.44	3.42E-03	3.14E-02	down
c12103_g1	2.731	0	-4.82	3.42E-03	3.14E-02	down
c12326_g1	9.182	0	-6.54	3.42E-03	3.14E-02	down
c914_g1	5.19	0	-5.73	3.42E-03	3.14E-02	down
c7110_g1	7.256	0	-6.2	3.42E-03	3.14E-02	down
c23881_g1	9.599	0	-6.6	3.42E-03	3.14E-02	down
c22172_g1	3.411	0	-5.13	3.42E-03	3.14E-02	down
c28151_g1	2.769	0	-4.84	3.42E-03	3.14E-02	down
c33358_g1	4.943	0	-5.66	3.42E-03	3.14E-02	down
c7969_g1	7.58	0	-6.26	3.42E-03	3.14E-02	down
c10793_g1	27.676	1.926	-3.78	3.43E-03	3.14E-02	down
c35012_g1	8.764	70.91	3	3.43E-03	3.14E-02	up
c9224_g1	5.19	42.145	3	3.44E-03	3.15E-02	up
c6788_g1	2.599	23.051	3.1	3.45E-03	3.16E-02	up
c7790_g1	1.957	16.199	2.99	3.46E-03	3.17E-02	up
c16960_g1	1.199	14.996	3.54	3.47E-03	3.18E-02	up
c22483_g1	64.009	6.412	-3.3	3.48E-03	3.18E-02	down
c12378_g1	6.977	0.207	-4.53	3.49E-03	3.18E-02	down

seq_id	Fpkm of flower	Fpkm of leaf	log2FC (leaf/flower)	p-value	FDR	regulate
c11381_g1	4.332	0.129	-4.27	3.49E-03	3.18E-02	down
c13225_g1	0.534	12.307	4.29	3.49E-03	3.18E-02	up
c11185_g1	3.272	26.864	3	3.49E-03	3.18E-02	up
c18148_g1	38.699	308.9	2.99	3.49E-03	3.18E-02	up
c4559_g1	15.37	0.944	-3.89	3.49E-03	3.18E-02	down
c18868_g1	4.889	0.297	-3.65	3.49E-03	3.18E-02	down
c13509_g1	12.732	106.772	3.06	3.50E-03	3.18E-02	up
c15481_g1	7.805	64.226	3.02	3.50E-03	3.18E-02	up
c11090_g2	15.323	1.357	-3.4	3.50E-03	3.18E-02	down
c7291_g1	9.143	0.504	-3.94	3.50E-03	3.18E-02	down
c7311_g1	0.774	10.407	3.59	3.50E-03	3.18E-02	up
c38049_g1	0.82	11.053	3.6	3.50E-03	3.18E-02	up
c35101_g1	27.7	2.702	-3.31	3.51E-03	3.19E-02	down
c26136_g1	47.123	4.77	-3.28	3.51E-03	3.19E-02	down
c4679_g1	0.92	9.502	3.23	3.52E-03	3.19E-02	up
c27487_g1	17.087	1.435	-3.49	3.52E-03	3.19E-02	down
c30843_g1	8.099	65.041	2.99	3.53E-03	3.20E-02	up
c3982_g1	0.975	8.287	2.96	3.55E-03	3.22E-02	up
c461_g1	2.63	22.068	3.02	3.57E-03	3.23E-02	up
c5578_g1	1.787	16.263	3.12	3.57E-03	3.23E-02	up
c32088_g1	18.433	1.745	-3.33	3.58E-03	3.24E-02	down
c4895_g1	138.344	14.324	-3.26	3.58E-03	3.24E-02	down
c9991_g2	19.686	1.345	-3.78	3.59E-03	3.24E-02	down
c17969_g1	3.999	32.165	2.98	3.61E-03	3.26E-02	up
c7342_g1	6.258	0.517	-3.37	3.61E-03	3.26E-02	down
c2952_g1	2.429	20.051	2.99	3.62E-03	3.26E-02	up
c3729_g1	34.862	3.581	-3.25	3.62E-03	3.27E-02	down
c10286_g1	2.908	30.743	3.36	3.63E-03	3.27E-02	up
c2953_g1	41.716	4.279	-3.26	3.63E-03	3.27E-02	down
c7506_g1	2.483	21.111	3.04	3.64E-03	3.28E-02	up
c9672_g1	8.493	0.776	-3.29	3.65E-03	3.28E-02	down
c4212_g2	2.707	25.597	3.19	3.65E-03	3.28E-02	up
c17772_g1	38.707	306.884	2.98	3.65E-03	3.28E-02	up
c1350_g1	2.885	26.14	3.14	3.65E-03	3.28E-02	up
c8692_g1	11.471	0.814	-3.66	3.67E-03	3.30E-02	down
c6748_g1	7.395	0.53	-3.57	3.67E-03	3.30E-02	down
c10782_g1	19.493	1.396	-3.71	3.67E-03	3.30E-02	down
c10993_g2	11.866	93.081	2.96	3.68E-03	3.30E-02	up
c26087_g1	14.542	114.71	2.97	3.68E-03	3.30E-02	up
c27372_g1	29.146	2.792	-3.34	3.68E-03	3.30E-02	down
c34989_g1	27.352	215.25	2.97	3.69E-03	3.31E-02	up
c11776_g1	6.018	0.543	-3.25	3.69E-03	3.31E-02	down

seq_id	Fpkm of flower	Fpkm of leaf	log2FC (leaf/flower)	p-value	FDR	regulate
c5860_g1	10.783	1.164	-3.11	3.69E-03	3.31E-02	down
c12070_g1	20.096	1.538	-3.62	3.70E-03	3.31E-02	down
c9234_g1	6.675	54.349	3.01	3.70E-03	3.31E-02	up
c6179_g1	67.281	6.891	-3.27	3.73E-03	3.33E-02	down
c13787_g1	3.164	0.194	-3.47	3.73E-03	3.33E-02	down
c21962_g1	0.65	8.222	3.47	3.73E-03	3.33E-02	up
c7498_g1	6.598	53.806	3.01	3.73E-03	3.33E-02	up
c6044_g1	9.104	0.763	-3.41	3.74E-03	3.33E-02	down
c19088_g1	31.923	3.077	-3.33	3.74E-03	3.33E-02	down
c11242_g1	18.557	1.538	-3.51	3.79E-03	3.37E-02	down
c12718_g1	27.15	1.37	-4.21	3.79E-03	3.37E-02	down
c10201_g1	13.714	0.595	-4.31	3.79E-03	3.37E-02	down
c4089_g1	8.308	0.414	-4.03	3.79E-03	3.37E-02	down
c10411_g1	13.66	1.37	-3.23	3.79E-03	3.37E-02	down
c14733_g1	47.858	4.822	-3.28	3.80E-03	3.37E-02	down
c11144_g1	5.051	38.512	2.91	3.80E-03	3.37E-02	up
c588_g1	0.735	6.335	2.95	3.81E-03	3.37E-02	up
c3940_g1	2.181	20.439	3.17	3.81E-03	3.37E-02	up
c6352_g1	5.113	0.207	-4.09	3.81E-03	3.37E-02	down
c4935_g1	3.697	0.155	-3.9	3.81E-03	3.37E-02	down
c6234_g1	16.778	0.685	-4.43	3.81E-03	3.37E-02	down
c12582_g2	10.326	0.271	-4.81	3.81E-03	3.37E-02	down
c12244_g1	5.384	0.22	-4.1	3.81E-03	3.37E-02	down
c6977_g1	5.19	0.22	-4.05	3.81E-03	3.37E-02	down
c3852_g1	3.96	0.168	-3.92	3.81E-03	3.37E-02	down
c8578_g1	5.585	0.168	-4.41	3.81E-03	3.37E-02	down
c38545_g1	1.818	30.794	4.01	3.81E-03	3.37E-02	up
c8050_g1	0.812	8.261	3.2	3.81E-03	3.37E-02	up
c7599_g1	80.268	8.468	-3.23	3.82E-03	3.37E-02	down
c4120_g1	10.334	82.881	2.99	3.82E-03	3.38E-02	up
c36678_g1	0.611	8.649	3.62	3.83E-03	3.38E-02	up
c12400_g3	0.333	4.641	3.45	3.83E-03	3.38E-02	up
c3162_g1	0.248	3.426	3.34	3.83E-03	3.38E-02	up
c5519_g1	0.441	6.27	3.56	3.83E-03	3.38E-02	up
c10741_g1	18.673	144.56	2.95	3.84E-03	3.39E-02	up
c9416_g1	7.983	0.569	-3.59	3.85E-03	3.39E-02	down
c36275_g1	0.936	9.489	3.21	3.85E-03	3.39E-02	up
c17916_g1	18.371	145.504	2.98	3.86E-03	3.40E-02	up
c12757_g1	16.484	1.422	-3.45	3.87E-03	3.40E-02	down
c14418_g1	8.47	66.049	2.95	3.88E-03	3.41E-02	up
c14674_g1	15.254	1.422	-3.33	3.89E-03	3.41E-02	down
c12612_g1	10.961	0.892	-3.48	3.89E-03	3.41E-02	down

seq_id	Fpkm of flower	Fpkm of leaf	log2FC (leaf/flower)	p-value	FDR	regulate
c15174_g1	2.932	35.978	3.57	3.89E-03	3.41E-02	up
c2614_g1	16.886	130.249	2.94	3.90E-03	3.42E-02	up
c18045_g1	27.491	213.867	2.96	3.90E-03	3.42E-02	up
c4721_g1	14.759	1.435	-3.28	3.92E-03	3.44E-02	down
c31322_g1	15.347	1.099	-3.69	3.92E-03	3.44E-02	down
c26676_g1	17.621	1.254	-3.71	3.92E-03	3.44E-02	down
c13105_g3	30.461	3.193	-3.21	3.93E-03	3.44E-02	down
c4658_g1	1.671	14.88	3.08	3.93E-03	3.44E-02	up
c12299_g1	14.039	1.422	-3.22	3.93E-03	3.44E-02	down
c1873_g2	33.609	259.774	2.95	3.94E-03	3.44E-02	up
c17656_g1	59.282	6.205	-3.24	3.97E-03	3.47E-02	down
c36547_g1	1.911	24.149	3.59	3.98E-03	3.48E-02	up
c6715_g1	6.683	53.922	2.99	3.99E-03	3.48E-02	up
c36452_g1	56.83	457.468	3.01	3.99E-03	3.48E-02	up
c14049_g1	9.019	70.134	2.95	3.99E-03	3.48E-02	up
c11561_g1	8.88	0.814	-3.3	4.01E-03	3.49E-02	down
c12903_g1	12.964	1.267	-3.26	4.01E-03	3.49E-02	down
c4941_g2	214.984	23.051	-3.22	4.04E-03	3.52E-02	down
c12797_g1	9.058	0.892	-3.21	4.05E-03	3.52E-02	down
c14989_g1	118.418	12.385	-3.25	4.06E-03	3.53E-02	down
c1855_g1	135.474	1032.93	2.93	4.07E-03	3.54E-02	up
c6813_g1	13.312	1.267	-3.29	4.08E-03	3.54E-02	down
c4522_g1	9.274	0.259	-4.71	4.10E-03	3.55E-02	down
c830_g1	6.614	0.323	-3.99	4.10E-03	3.55E-02	down
c18403_g1	43.982	4.654	-3.21	4.10E-03	3.55E-02	down
c28601_g1	3.442	0	-5.15	4.10E-03	3.55E-02	down
c24114_g1	5.221	0	-5.73	4.10E-03	3.55E-02	down
c27355_g1	2.321	0	-4.6	4.10E-03	3.55E-02	down
c22152_g1	3.117	0	-5.01	4.10E-03	3.55E-02	down
c13200_g2	5.477	0	-5.8	4.10E-03	3.55E-02	down
c36819_g1	7.186	0	-6.19	4.10E-03	3.55E-02	down
c30988_g1	2.761	0	-4.84	4.10E-03	3.55E-02	down
c28195_g1	2.7	0	-4.81	4.10E-03	3.55E-02	down
c9342_g1	6.165	0.44	-3.54	4.11E-03	3.55E-02	down
c9950_g1	0.565	5.611	3.1	4.11E-03	3.55E-02	up
c11443_g1	31.722	2.405	-3.67	4.12E-03	3.56E-02	down
c26516_g1	148.763	15.811	-3.23	4.12E-03	3.56E-02	down
c21656_g1	27.762	211.398	2.92	4.13E-03	3.56E-02	up
c4651_g1	32.712	3.245	-3.29	4.14E-03	3.57E-02	down
c1508_g1	17.567	1.732	-3.27	4.15E-03	3.58E-02	down
c34803_g1	43.309	4.615	-3.2	4.15E-03	3.58E-02	down
c21739_g1	683.333	74.452	-3.2	4.16E-03	3.58E-02	down

seq_id	Fpkm of flower	Fpkm of leaf	log2FC (leaf/flower)	p-value	FDR	regulate
c1917_g1	8.014	0.621	-3.49	4.18E-03	3.60E-02	down
c5464_g1	2.003	16.108	2.95	4.21E-03	3.62E-02	up
c8136_g1	4.146	31.751	2.91	4.22E-03	3.62E-02	up
c17888_g1	158.687	17.142	-3.2	4.22E-03	3.62E-02	down
c4637_g1	0.394	4.344	3.17	4.22E-03	3.62E-02	up
c36308_g1	0.302	4.111	3.39	4.22E-03	3.62E-02	up
c22833_g1	0.248	3.322	3.3	4.22E-03	3.62E-02	up
c6154_g1	0.364	4.977	3.45	4.22E-03	3.62E-02	up
c8126_g1	5.152	0.427	-3.32	4.25E-03	3.64E-02	down
c4085_g1	0.688	6.205	3	4.25E-03	3.64E-02	up
c3741_g1	14.356	1.422	-3.25	4.25E-03	3.64E-02	down
c34972_g1	13.096	99.403	2.91	4.26E-03	3.65E-02	up
c7383_g1	13.188	0.814	-3.86	4.26E-03	3.65E-02	down
c9369_g1	21.403	2.159	-3.25	4.27E-03	3.65E-02	down
c7705_g1	39.341	297.317	2.91	4.27E-03	3.65E-02	up
c7627_g1	13.614	1.332	-3.26	4.30E-03	3.67E-02	down
c345_g1	0.534	5.262	3.08	4.30E-03	3.68E-02	up
c7262_g1	49.838	5.145	-3.25	4.32E-03	3.68E-02	down
c36950_g1	0.108	3.348	4.05	4.33E-03	3.68E-02	up
c22412_g1	0.155	4.874	4.29	4.33E-03	3.68E-02	up
c11948_g2	52.174	2.379	-4.4	4.33E-03	3.68E-02	down
c16727_g1	8.424	0.362	-4.21	4.33E-03	3.68E-02	down
c37149_g1	0.487	7.563	3.71	4.33E-03	3.68E-02	up
c24792_g1	0.503	7.899	3.73	4.33E-03	3.68E-02	up
c10727_g2	1.996	32.514	3.96	4.33E-03	3.68E-02	up
c2968_g1	1.663	11.377	2.7	4.33E-03	3.68E-02	up
c35447_g1	0.24	3.723	3.49	4.33E-03	3.68E-02	up
c32255_g1	0.456	7.123	3.7	4.33E-03	3.68E-02	up
c13720_g1	1430.985	160.488	-3.16	4.33E-03	3.68E-02	down
c5723_g1	15.989	1.357	-3.47	4.34E-03	3.69E-02	down
c37289_g1	4.154	38.034	3.16	4.34E-03	3.69E-02	up
c7469_g1	2.638	21.822	3	4.35E-03	3.69E-02	up
c26022_g1	55.461	5.999	-3.19	4.36E-03	3.70E-02	down
c6577_g1	22.115	2.353	-3.18	4.36E-03	3.70E-02	down
c17958_g1	4.749	36.987	2.94	4.37E-03	3.70E-02	up
c12618_g1	7.697	58.887	2.92	4.37E-03	3.70E-02	up
c11374_g1	15.486	1.176	-3.61	4.37E-03	3.70E-02	down
c6939_g1	11.649	0.879	-3.59	4.37E-03	3.70E-02	down
c30375_g1	1313.897	146.241	-3.17	4.38E-03	3.70E-02	down
c6159_g1	10.334	1.06	-3.17	4.41E-03	3.72E-02	down
c35032_g1	0.603	5.417	2.97	4.41E-03	3.72E-02	up
c6238_g1	2.174	19.172	3.08	4.41E-03	3.72E-02	up

seq_id	Fpkm of flower	Fpkm of leaf	log2FC (leaf/flower)	p-value	FDR	regulate
c2211_g1	1.245	12.126	3.18	4.41E-03	3.72E-02	up
c12161_g1	1.269	11.131	3.04	4.41E-03	3.72E-02	up
c25976_g1	25.812	195.147	2.91	4.43E-03	3.73E-02	up
c5728_g2	3.11	25.248	2.98	4.43E-03	3.74E-02	up
c13984_g1	0.572	7.24	3.45	4.44E-03	3.74E-02	up
c21453_g1	3.767	49.979	3.69	4.44E-03	3.74E-02	up
c462_g1	0.951	9.412	3.18	4.45E-03	3.75E-02	up
c13473_g1	244.865	1830.029	2.9	4.47E-03	3.76E-02	up
c12148_g1	20.104	145.97	2.85	4.48E-03	3.77E-02	up
c31884_g1	25.936	2.754	-3.19	4.50E-03	3.78E-02	down
c30377_g1	36.796	273.219	2.89	4.55E-03	3.82E-02	up
c910_g1	0.511	4.977	3.05	4.55E-03	3.82E-02	up
c30252_g1	13.947	1.293	-3.33	4.56E-03	3.82E-02	down
c477_g1	7.635	0.711	-3.25	4.56E-03	3.82E-02	down
c15806_g1	8.679	0.569	-3.71	4.56E-03	3.82E-02	down
c35030_g1	10.62	0.698	-3.75	4.56E-03	3.82E-02	down
c18979_g1	0.433	5.094	3.28	4.56E-03	3.82E-02	up
c35827_g1	0.495	5.831	3.32	4.56E-03	3.82E-02	up
c8690_g1	1.609	13.768	3.02	4.57E-03	3.82E-02	up
c1558_g1	101.431	11.26	-3.16	4.57E-03	3.82E-02	down
c17701_g1	18.603	139.079	2.9	4.57E-03	3.82E-02	up
c7748_g1	11.1	0.957	-3.41	4.59E-03	3.84E-02	down
c13398_g1	12.856	1.267	-3.24	4.59E-03	3.84E-02	down
c18401_g1	9.344	69.811	2.89	4.59E-03	3.84E-02	up
c14845_g1	12.724	96.714	2.92	4.60E-03	3.84E-02	up
c10547_g2	21.047	156.545	2.89	4.60E-03	3.84E-02	up
c26186_g1	10.195	76.223	2.89	4.62E-03	3.86E-02	up
c10072_g1	235.327	1520.715	2.69	4.63E-03	3.86E-02	up
c4846_g2	0.511	5.908	3.3	4.64E-03	3.86E-02	up
c9057_g1	12.33	0.84	-3.73	4.64E-03	3.86E-02	down
c1755_g2	12.384	0.944	-3.58	4.64E-03	3.86E-02	down
c26607_g1	7.356	0.556	-3.51	4.64E-03	3.86E-02	down
c19469_g1	0.936	9.218	3.17	4.64E-03	3.86E-02	up
c22262_g1	29.285	216.582	2.88	4.65E-03	3.87E-02	up
c10065_g1	4.641	35.604	2.91	4.67E-03	3.88E-02	up
c11843_g1	21.836	2.237	-3.23	4.67E-03	3.88E-02	down
c8098_g1	1.021	9.373	3.08	4.67E-03	3.88E-02	up
c4846_g1	12.082	89.914	2.89	4.68E-03	3.88E-02	up
c5924_g1	49.985	4.874	-3.33	4.70E-03	3.90E-02	down
c5597_g1	24.66	2.56	-3.22	4.71E-03	3.91E-02	down
c7742_g1	12.956	0.97	-3.61	4.73E-03	3.92E-02	down
c8000_g1	18.982	140.785	2.88	4.76E-03	3.94E-02	up

seq_id	Fpkm of flower	Fpkm of leaf	log2FC (leaf/flower)	p-value	FDR	regulate
c13562_g1	35.814	264.002	2.88	4.78E-03	3.96E-02	up
c5489_g1	7.689	0.698	-3.29	4.79E-03	3.96E-02	down
c365_g1	23.894	2.586	-3.16	4.79E-03	3.96E-02	down
c18392_g1	8.571	64.161	2.89	4.80E-03	3.97E-02	up
c8703_g1	25.534	2.728	-3.18	4.80E-03	3.97E-02	down
c11890_g1	15.169	1.629	-3.14	4.81E-03	3.98E-02	down
c10859_g1	2.166	5.65	1.34	4.81E-03	3.98E-02	up
c35051_g1	6.699	0.362	-3.88	4.82E-03	3.98E-02	down
c10766_g1	11.069	0.595	-4.01	4.82E-03	3.98E-02	down
c6147_g1	29.27	3.232	-3.14	4.83E-03	3.98E-02	down
c26255_g1	20.676	151.231	2.86	4.84E-03	3.98E-02	up
c26653_g1	4.595	37.969	3.02	4.84E-03	3.98E-02	up
c5708_g1	4.37	32.139	2.85	4.85E-03	3.98E-02	up
c14492_g1	38.552	4.266	-3.15	4.85E-03	3.98E-02	down
c3113_g1	28.156	205.813	2.87	4.85E-03	3.98E-02	up
c3136_g1	6.985	51.479	2.86	4.87E-03	3.98E-02	up
c7843_g1	9.104	68.415	2.9	4.88E-03	3.98E-02	up
c5281_g1	13.088	98.782	2.91	4.88E-03	3.98E-02	up
c16463_g1	9.027	0.608	-3.69	4.89E-03	3.98E-02	down
c26995_g1	23.639	2.534	-3.17	4.90E-03	3.98E-02	down
c12092_g1	30.716	3.232	-3.21	4.92E-03	3.98E-02	down
c13683_g1	0.619	6.994	3.3	4.93E-03	3.98E-02	up
c11576_g1	14.163	1.073	-3.6	4.93E-03	3.98E-02	down
c16043_g1	5.763	58.008	3.31	4.93E-03	3.98E-02	up
c4285_g1	1.222	11.881	3.18	4.93E-03	3.98E-02	up
c24759_g1	0.178	5.391	4.3	4.93E-03	3.98E-02	up
c34510_g1	0.124	3.749	4.1	4.93E-03	3.98E-02	up
c120_g1	0.248	7.459	4.44	4.93E-03	3.98E-02	up
c13101_g1	0.124	3.568	4.03	4.93E-03	3.98E-02	up
c8830_g1	4.347	0.194	-3.92	4.93E-03	3.98E-02	down
c36033_g1	3.976	0.181	-3.86	4.93E-03	3.98E-02	down
c11698_g1	3.914	0.168	-3.9	4.93E-03	3.98E-02	down
c7463_g1	11.997	0.543	-4.23	4.93E-03	3.98E-02	down
c26163_g1	4.525	0.194	-3.98	4.93E-03	3.98E-02	down
c19130_g1	0.286	4.24	3.49	4.93E-03	3.98E-02	up
c32483_g1	0.58	8.701	3.69	4.93E-03	3.98E-02	up
c10511_g1	0.48	7.136	3.64	4.93E-03	3.98E-02	up
c14547_g1	0.302	4.46	3.5	4.93E-03	3.98E-02	up
c36691_g1	1.408	21.564	3.84	4.93E-03	3.98E-02	up
c19947_g1	0.317	4.719	3.53	4.93E-03	3.98E-02	up
c31987_g1	0.418	6.192	3.6	4.93E-03	3.98E-02	up
c36028_g1	0.58	8.701	3.69	4.93E-03	3.98E-02	up

seq_id	Fpkm of flower	Fpkm of leaf	log2FC (leaf/flower)	p-value	FDR	regulate
c21676_g1	228.675	1661.694	2.86	4.93E-03	3.98E-02	up
c31184_g1	11.394	83.45	2.86	4.94E-03	3.98E-02	up
c17959_g1	27.854	202.995	2.86	4.95E-03	3.98E-02	up
c1467_g1	0.719	6.839	3.08	4.95E-03	3.98E-02	up
c564_g1	4.858	36.948	2.9	4.95E-03	3.98E-02	up
c11742_g1	18.827	1.849	-3.28	4.95E-03	3.98E-02	down
c8169_g1	7.387	0.556	-3.51	4.96E-03	3.98E-02	down
c160_g1	1.733	15.1	3.05	4.96E-03	3.98E-02	up
c14754_g1	18.216	133.585	2.87	4.96E-03	3.98E-02	up
c36369_g1	6.149	0.504	-3.37	4.96E-03	3.98E-02	down
c1518_g1	1.222	11.558	3.14	4.96E-03	3.98E-02	up
c12797_g2	8.671	0	-6.45	4.96E-03	3.98E-02	down
c19717_g1	4.564	0	-5.54	4.96E-03	3.98E-02	down
c13649_g1	6.776	0	-6.1	4.96E-03	3.98E-02	down
c6127_g1	3.968	0	-5.35	4.96E-03	3.98E-02	down
c28584_g1	2.723	0	-4.82	4.96E-03	3.98E-02	down
c34009_g1	6.057	0	-5.94	4.96E-03	3.98E-02	down
c69_g1	3.21	0	-5.05	4.96E-03	3.98E-02	down
c26394_g1	2.839	0	-4.88	4.96E-03	3.98E-02	down
c7930_g1	6.923	0	-6.13	4.96E-03	3.98E-02	down
c7497_g1	7.287	0	-6.21	4.96E-03	3.98E-02	down
c13087_g2	4.796	0	-5.61	4.96E-03	3.98E-02	down
c6751_g1	2.676	0	-4.79	4.96E-03	3.98E-02	down
c20058_g1	6.149	0	-5.97	4.96E-03	3.98E-02	down
c1187_g1	3.171	0	-5.03	4.96E-03	3.98E-02	down
c10410_g1	2.205	0	-4.53	4.96E-03	3.98E-02	down
c13151_g2	9.878	0	-6.64	4.96E-03	3.98E-02	down
c8529_g1	4.796	0	-5.61	4.96E-03	3.98E-02	down
c1491_g1	3.566	0	-5.2	4.96E-03	3.98E-02	down
c409_g2	11.99	0	-6.92	4.96E-03	3.98E-02	down
c1209_g1	5.778	0	-5.88	4.96E-03	3.98E-02	down
c9126_g1	0.495	4.266	2.88	4.96E-03	3.98E-02	up
c35491_g1	17.636	129.047	2.86	4.97E-03	3.99E-02	up
c19641_g1	60.095	6.386	-3.21	4.97E-03	3.99E-02	down
c18087_g1	14.852	108.569	2.86	4.98E-03	3.99E-02	up
c18431_g1	16.422	119.467	2.86	4.99E-03	4.00E-02	up
c21786_g1	407.126	2943.009	2.85	4.99E-03	4.00E-02	up
c30570_g1	64.009	7.214	-3.13	4.99E-03	4.00E-02	down
c29995_g1	43.797	318.002	2.86	5.00E-03	4.00E-02	up
c11604_g3	3.535	26.283	2.86	5.00E-03	4.00E-02	up
c5673_g1	19.825	2.146	-3.15	5.01E-03	4.01E-02	down
c10356_g1	329.611	37.607	-3.13	5.02E-03	4.01E-02	down

seq_id	Fpkm of flower	Fpkm of leaf	log2FC (leaf/flower)	p-value	FDR	regulate
c34664_g1	597.751	68.622	-3.12	5.03E-03	4.02E-02	down
c11966_g2	3.697	27.058	2.84	5.03E-03	4.02E-02	up
c14857_g1	36.518	3.84	-3.22	5.04E-03	4.02E-02	down
c9126_g2	4.131	30.769	2.87	5.09E-03	4.06E-02	up
c14854_g1	9.12	0.879	-3.24	5.09E-03	4.06E-02	down
c35324_g1	26.068	2.896	-3.13	5.10E-03	4.07E-02	down
c12191_g1	8.632	0.802	-3.28	5.11E-03	4.07E-02	down
c10948_g3	33.323	3.4	-3.26	5.11E-03	4.07E-02	down
c4356_g1	4.27	31.311	2.85	5.11E-03	4.07E-02	up
c13458_g1	287.888	32.953	-3.12	5.11E-03	4.07E-02	down
c26615_g1	259.724	1870.933	2.85	5.11E-03	4.07E-02	up
c34929_g1	7	0.724	-3.11	5.13E-03	4.08E-02	down
c30221_g1	27.236	195.858	2.84	5.13E-03	4.08E-02	up
c2279_g1	1.075	9.735	3.07	5.13E-03	4.08E-02	up
c22099_g1	30.902	223.977	2.85	5.14E-03	4.08E-02	up
c444_g1	77.483	8.752	-3.13	5.16E-03	4.09E-02	down
c27516_g1	32.232	3.607	-3.12	5.16E-03	4.09E-02	down
c18730_g1	0.309	4.021	3.33	5.16E-03	4.09E-02	up
c36059_g1	0.611	8.028	3.51	5.16E-03	4.09E-02	up
c248_g1	0.611	11.053	3.97	5.16E-03	4.09E-02	up
c12246_g1	25.426	2.12	-3.52	5.18E-03	4.11E-02	down
c3861_g1	4.123	29.799	2.82	5.21E-03	4.13E-02	up
c10630_g1	14.225	87.315	2.61	5.22E-03	4.13E-02	up
c10463_g1	35.296	3.904	-3.14	5.22E-03	4.13E-02	down
c13275_g1	101.315	11.532	-3.12	5.23E-03	4.13E-02	down
c9560_g1	7.503	0.401	-3.92	5.23E-03	4.13E-02	down
c8692_g2	9.166	1.151	-2.89	5.24E-03	4.13E-02	down
c3228_g1	0.364	3.426	2.93	5.24E-03	4.13E-02	up
c26884_g1	0.603	5.727	3.05	5.24E-03	4.13E-02	up
c26234_g1	4.37	0.297	-3.49	5.24E-03	4.14E-02	down
c20458_g1	0.611	7.072	3.33	5.24E-03	4.14E-02	up
c6810_g1	14.318	103.243	2.84	5.25E-03	4.14E-02	up
c17891_g1	12.253	88.608	2.84	5.26E-03	4.14E-02	up
c3495_g1	26.617	191.437	2.84	5.27E-03	4.15E-02	up
c18624_g1	117.265	13.445	-3.12	5.28E-03	4.16E-02	down
c6118_g1	9.135	65.545	2.83	5.29E-03	4.16E-02	up
c15947_g1	11.603	85.312	2.87	5.29E-03	4.16E-02	up
c7105_g1	38.599	4.408	-3.1	5.30E-03	4.17E-02	down
c7552_g1	27.97	2.754	-3.3	5.33E-03	4.19E-02	down
c6821_g2	5.786	41.292	2.81	5.33E-03	4.19E-02	up
c1258_g1	16.491	1.732	-3.18	5.34E-03	4.19E-02	down
c10214_g1	4.27	33.031	2.92	5.34E-03	4.19E-02	up

seq_id	Fpkm of flower	Fpkm of leaf	log2FC (leaf/flower)	p-value	FDR	regulate
c7262_g2	21.434	2.275	-3.18	5.36E-03	4.21E-02	down
c7819_g1	7.379	53.897	2.85	5.36E-03	4.21E-02	up
c12686_g1	6.049	44.149	2.85	5.38E-03	4.22E-02	up
c12465_g3	15.726	3.129	-2.29	5.38E-03	4.22E-02	down
c14084_g1	255.315	29.605	-3.1	5.38E-03	4.22E-02	down
c12494_g1	11.185	0.944	-3.43	5.42E-03	4.24E-02	down
c6551_g1	12.717	0.659	-4.08	5.42E-03	4.24E-02	down
c2010_g1	12.384	1.112	-3.36	5.42E-03	4.24E-02	down
c7558_g2	12.167	86.385	2.82	5.45E-03	4.26E-02	up
c12824_g2	14.674	1.564	-3.15	5.45E-03	4.26E-02	down
c21782_g2	125.503	883.341	2.81	5.45E-03	4.26E-02	up
c34620_g1	48.531	342.785	2.82	5.47E-03	4.27E-02	up
c35233_g1	11.904	85.143	2.83	5.48E-03	4.28E-02	up
c14780_g1	60.234	6.8	-3.13	5.49E-03	4.29E-02	down
c12343_g1	13.382	1.319	-3.25	5.50E-03	4.29E-02	down
c18623_g1	105.137	12.217	-3.09	5.51E-03	4.29E-02	down
c35999_g1	0.719	6.684	3.05	5.51E-03	4.30E-02	up
c35151_g1	61.696	7.149	-3.09	5.52E-03	4.30E-02	down
c13474_g1	251.548	1777.244	2.82	5.53E-03	4.31E-02	up
c15360_g1	49.018	5.391	-3.16	5.55E-03	4.32E-02	down
c11644_g1	7.395	52.475	2.81	5.55E-03	4.32E-02	up
c11007_g1	12.175	1.332	-3.1	5.56E-03	4.32E-02	down
c27500_g1	37.848	4.24	-3.13	5.56E-03	4.32E-02	down
c17777_g1	785.082	5507.359	2.81	5.57E-03	4.33E-02	up
c12889_g2	0.549	5.882	3.2	5.57E-03	4.33E-02	up
c7794_g1	0.975	8.442	2.99	5.57E-03	4.33E-02	up
c14140_g1	155.098	18.177	-3.09	5.58E-03	4.33E-02	down
c18155_g1	19.988	2.275	-3.08	5.59E-03	4.34E-02	down
c5521_g1	6.915	0.621	-3.28	5.60E-03	4.35E-02	down
c11209_g1	21.86	2.301	-3.19	5.61E-03	4.35E-02	down
c15703_g1	0.557	6.283	3.28	5.63E-03	4.36E-02	up
c10019_g1	2.97	24.55	3.01	5.64E-03	4.36E-02	up
c26286_g1	3.868	0.181	-3.82	5.64E-03	4.36E-02	down
c1167_g1	6.258	0.284	-4.05	5.64E-03	4.36E-02	down
c9857_g1	10.736	0.582	-3.99	5.64E-03	4.36E-02	down
c22252_g1	38.792	4.447	-3.1	5.64E-03	4.36E-02	down
c35905_g1	9.352	76.702	3.02	5.65E-03	4.37E-02	up
c26454_g1	55.732	6.309	-3.12	5.66E-03	4.37E-02	down
c3857_g1	36.092	4.008	-3.14	5.66E-03	4.37E-02	down
c9968_g2	8.841	0.879	-3.19	5.69E-03	4.39E-02	down
c1812_g1	6.057	0.336	-3.82	5.69E-03	4.39E-02	down
c6066_g1	7.836	0.44	-3.88	5.69E-03	4.39E-02	down

seq_id	Fpkm of flower	Fpkm of leaf	log2FC (leaf/flower)	p-value	FDR	regulate
c2060_g1	0.394	6.955	3.84	5.69E-03	4.39E-02	up
c1590_g1	9.553	67.264	2.8	5.70E-03	4.39E-02	up
c7974_g1	2.576	19.043	2.84	5.71E-03	4.40E-02	up
c8164_g1	17.822	128.116	2.84	5.71E-03	4.40E-02	up
c23364_g1	1.694	22.081	3.63	5.73E-03	4.40E-02	up
c2414_g1	0.418	5.339	3.39	5.73E-03	4.40E-02	up
c8082_g1	0.24	2.999	3.19	5.73E-03	4.40E-02	up
c21978_g1	0.333	4.253	3.33	5.73E-03	4.40E-02	up
c34573_g1	0.433	5.494	3.39	5.73E-03	4.40E-02	up
c22631_g1	1.423	18.422	3.6	5.73E-03	4.40E-02	up
c7220_g1	0.317	3.995	3.3	5.73E-03	4.40E-02	up
c20131_g1	0.828	10.588	3.53	5.73E-03	4.40E-02	up
c34943_g1	13.281	1.461	-3.1	5.77E-03	4.43E-02	down
c30273_g1	221.69	1537.133	2.79	5.80E-03	4.45E-02	up
c17859_g1	193.31	1339.077	2.79	5.80E-03	4.45E-02	up
c26038_g1	7.658	54.026	2.8	5.81E-03	4.45E-02	up
c28064_g1	38.923	4.111	-3.21	5.81E-03	4.45E-02	down
c22603_g1	102.182	11.894	-3.09	5.82E-03	4.46E-02	down
c15214_g1	4.053	28.687	2.79	5.83E-03	4.46E-02	up
c30322_g1	28.148	196.195	2.8	5.85E-03	4.48E-02	up
c12430_g1	15.323	1.771	-3.04	5.86E-03	4.48E-02	down
c25973_g1	153.829	1062.548	2.79	5.86E-03	4.48E-02	up
c25975_g1	199.243	1373.892	2.79	5.92E-03	4.52E-02	up
c12013_g1	18.796	1.849	-3.28	5.93E-03	4.53E-02	down
c31848_g1	74.281	8.584	-3.1	5.93E-03	4.53E-02	down
c12457_g2	6.026	43.27	2.82	5.94E-03	4.54E-02	up
c35254_g1	35.752	246.639	2.78	5.96E-03	4.55E-02	up
c829_g1	2.228	17.996	2.96	5.97E-03	4.55E-02	up
c4396_g1	0.913	7.511	2.91	5.98E-03	4.56E-02	up
c23077_g1	0.982	8.067	2.92	5.98E-03	4.56E-02	up
c35494_g1	34.437	3.749	-3.17	6.01E-03	4.58E-02	down
c4358_g1	24.93	2.831	-3.09	6.02E-03	4.58E-02	down
c27049_g1	9.205	0.737	-3.47	6.03E-03	4.58E-02	down
c11155_g2	1.911	14.945	2.9	6.04E-03	4.58E-02	up
c18065_g1	0.82	6.399	2.82	6.04E-03	4.58E-02	up
c21687_g1	27.267	189.33	2.79	6.05E-03	4.58E-02	up
c10052_g3	2.003	0	-4.39	6.05E-03	4.58E-02	down
c2260_g1	14.604	0	-7.2	6.05E-03	4.58E-02	down
c26684_g1	2.738	0	-4.83	6.05E-03	4.58E-02	down
c20493_g1	6.103	0	-5.95	6.05E-03	4.58E-02	down
c38444_g1	8.253	0	-6.38	6.05E-03	4.58E-02	down
c24200_g1	3.682	0	-5.24	6.05E-03	4.58E-02	down

seq_id	Fpkm of flower	Fpkm of leaf	log2FC (leaf/flower)	p-value	FDR	regulate
c12024_g1	9.406	0	-6.57	6.05E-03	4.58E-02	down
c34846_g1	2.421	0	-4.66	6.05E-03	4.58E-02	down
c11389_g1	16.058	0	-7.34	6.05E-03	4.58E-02	down
c2860_g1	43.719	4.887	-3.14	6.05E-03	4.58E-02	down
c30392_g1	19.268	132.718	2.78	6.05E-03	4.58E-02	up
c16244_g1	1.153	12.799	3.36	6.06E-03	4.58E-02	up
c1986_g1	2.638	29.708	3.44	6.06E-03	4.58E-02	up
c1453_g1	0.456	5.003	3.2	6.06E-03	4.58E-02	up
c3309_g1	14.503	1.655	-3.06	6.07E-03	4.59E-02	down
c9612_g1	1.122	10.045	3.05	6.08E-03	4.59E-02	up
c3358_g2	14.666	103.98	2.82	6.10E-03	4.60E-02	up
c10985_g1	26.006	3.064	-3.04	6.10E-03	4.60E-02	down
c592_g1	82.101	9.114	-3.16	6.10E-03	4.60E-02	down
c35795_g1	25.07	173.506	2.79	6.11E-03	4.61E-02	up
c27258_g1	6.938	48.493	2.79	6.12E-03	4.61E-02	up
c5588_g1	7.356	0.776	-3.09	6.14E-03	4.63E-02	down
c12511_g1	18.402	126.086	2.77	6.15E-03	4.63E-02	up
c10547_g1	11.974	81.756	2.76	6.17E-03	4.65E-02	up
c14898_g1	137.199	16.044	-3.09	6.18E-03	4.65E-02	down
c8607_g1	3.202	23.93	2.86	6.20E-03	4.66E-02	up
c8988_g1	8.424	0.737	-3.35	6.20E-03	4.66E-02	down
c641_g1	2.645	0.155	-3.43	6.21E-03	4.66E-02	down
c36608_g1	14.078	0.814	-3.96	6.21E-03	4.66E-02	down
c20632_g1	1.037	8.416	2.9	6.21E-03	4.67E-02	up
c9354_g1	15.88	1.836	-3.05	6.22E-03	4.67E-02	down
c31008_g1	236.805	28.661	-3.04	6.23E-03	4.68E-02	down
c10995_g1	483.309	50.316	-3.26	6.23E-03	4.68E-02	down
c26551_g1	4.216	29.23	2.76	6.26E-03	4.70E-02	up
c22365_g1	5.461	38.422	2.79	6.27E-03	4.70E-02	up
c22210_g1	4.254	29.683	2.77	6.30E-03	4.72E-02	up
c30186_g1	0.34	3.581	3.06	6.32E-03	4.73E-02	up
c30585_g1	0.812	7.343	3.03	6.32E-03	4.73E-02	up
c12776_g4	6.157	42.055	2.75	6.33E-03	4.74E-02	up
c4458_g1	10.976	1.241	-3.05	6.33E-03	4.74E-02	down
c10707_g1	2.39	17.22	2.8	6.35E-03	4.75E-02	up
c29978_g1	0.278	3.361	3.19	6.37E-03	4.75E-02	up
c11312_g1	0.634	8.274	3.51	6.37E-03	4.75E-02	up
c15729_g1	0.472	5.727	3.35	6.37E-03	4.75E-02	up
c25292_g1	2.088	26.438	3.6	6.37E-03	4.75E-02	up
c7192_g1	57.728	357.083	2.63	6.37E-03	4.75E-02	up
c1707_g1	15.393	1.655	-3.14	6.39E-03	4.77E-02	down
c10802_g1	9.684	1.073	-3.06	6.41E-03	4.78E-02	down

seq_id	Fpkm of flower	Fpkm of leaf	log2FC (leaf/flower)	p-value	FDR	regulate
c30244_g1	7.751	52.294	2.74	6.43E-03	4.79E-02	up
c10262_g1	6.938	51.841	2.88	6.44E-03	4.79E-02	up
c9699_g1	1.485	10.963	2.8	6.44E-03	4.79E-02	up
c22208_g1	59.398	7.085	-3.05	6.44E-03	4.79E-02	down
c26854_g1	106.954	12.579	-3.08	6.45E-03	4.79E-02	down
c14380_g1	46.249	5.481	-3.05	6.47E-03	4.79E-02	down
c9260_g1	0.17	4.822	4.19	6.47E-03	4.79E-02	up
c35784_g1	0.155	4.421	4.15	6.47E-03	4.79E-02	up
c19316_g1	0.147	4.124	4.1	6.47E-03	4.79E-02	up
c28447_g1	7.047	0.336	-4.03	6.47E-03	4.79E-02	down
c18517_g1	3.303	0.155	-3.74	6.47E-03	4.79E-02	down
c30760_g1	3.527	0.168	-3.76	6.47E-03	4.79E-02	down
c23903_g1	11.03	0.53	-4.14	6.47E-03	4.79E-02	down
c12843_g3	5.856	0.284	-3.96	6.47E-03	4.79E-02	down
c22498_g1	3.728	0.181	-3.77	6.47E-03	4.79E-02	down
c36284_g1	8.014	0.388	-4.06	6.47E-03	4.79E-02	down
c33537_g1	1.648	24.162	3.79	6.47E-03	4.79E-02	up
c36536_g1	0.255	3.568	3.37	6.47E-03	4.79E-02	up
c8162_g2	0.557	7.925	3.61	6.47E-03	4.79E-02	up
c18467_g1	18.789	128.09	2.76	6.48E-03	4.80E-02	up
c27317_g1	3.991	28.131	2.79	6.49E-03	4.80E-02	up
c5049_g1	8.122	0.724	-3.32	6.49E-03	4.80E-02	down
c7600_g1	3.396	23.49	2.75	6.50E-03	4.81E-02	up
c10385_g1	27.97	183.603	2.71	6.51E-03	4.81E-02	up
c27505_g1	1.044	7.783	2.78	6.52E-03	4.81E-02	up
c981_g1	3.179	25.882	2.99	6.52E-03	4.81E-02	up
c12972_g2	1.183	9.243	2.86	6.52E-03	4.81E-02	up
c30833_g1	4.223	29.14	2.76	6.53E-03	4.82E-02	up
c30106_g1	91.933	619.585	2.75	6.54E-03	4.82E-02	up
c13928_g1	16.437	110.443	2.74	6.55E-03	4.83E-02	up
c9539_g1	5.693	38.978	2.75	6.56E-03	4.83E-02	up
c4075_g1	18.077	1.887	-3.19	6.56E-03	4.83E-02	down
c9577_g1	16.19	1.926	-3.01	6.57E-03	4.84E-02	down
c29998_g1	38.622	4.68	-3.02	6.58E-03	4.84E-02	down
c35379_g1	5.794	39.417	2.75	6.58E-03	4.84E-02	up
c18218_g1	55.067	371.847	2.75	6.60E-03	4.85E-02	up
c7990_g1	1.648	13.006	2.91	6.61E-03	4.86E-02	up
c5621_g1	2.174	15.85	2.81	6.62E-03	4.86E-02	up
c4130_g1	87.446	10.394	-3.06	6.62E-03	4.86E-02	down
c8119_g1	18.789	126.487	2.74	6.63E-03	4.86E-02	up
c18669_g1	10.381	1.138	-3.08	6.63E-03	4.86E-02	down
c2184_g1	5.097	45.985	3.15	6.67E-03	4.90E-02	up

seq_id	Fpkm of flower	Fpkm of leaf	log2FC (leaf/flower)	p-value	FDR	regulate
c26109_g1	124.343	828.669	2.74	6.68E-03	4.90E-02	up
c7753_g1	133.3	16.302	-3.02	6.70E-03	4.91E-02	down
c548_g1	6.621	0.659	-3.15	6.71E-03	4.92E-02	down
c12585_g2	1.663	12.178	2.8	6.72E-03	4.92E-02	up
c2015_g1	20.784	139.195	2.74	6.72E-03	4.92E-02	up
c6876_g1	10.86	1.073	-3.22	6.73E-03	4.92E-02	down
c3485_g1	13.258	1.448	-3.11	6.73E-03	4.92E-02	down
c22131_g1	10.845	0.918	-3.43	6.74E-03	4.92E-02	down
c13901_g1	5.159	47.42	3.18	6.74E-03	4.92E-02	up
c18964_g1	1.083	9.605	3.04	6.74E-03	4.92E-02	up
c10042_g1	0.681	6.037	2.97	6.74E-03	4.92E-02	up
c27539_g1	0.657	5.831	2.97	6.74E-03	4.92E-02	up
c30571_g1	5.314	35.991	2.74	6.77E-03	4.94E-02	up
c4756_g1	5.6	37.504	2.72	6.77E-03	4.94E-02	up
c7043_g1	3.334	0.194	-3.55	6.78E-03	4.94E-02	down
c2066_g1	10.535	70.548	2.73	6.80E-03	4.96E-02	up
c11263_g2	33.339	215.393	2.69	6.80E-03	4.96E-02	up
c21929_g1	20.46	2.443	-3.02	6.81E-03	4.97E-02	down
c30005_g1	38.165	253.297	2.73	6.82E-03	4.97E-02	up
c26411_g1	525.528	3491.245	2.73	6.83E-03	4.97E-02	up
c34994_g1	7.434	49.863	2.73	6.83E-03	4.97E-02	up
c5676_g1	16.29	1.9	-3.03	6.84E-03	4.98E-02	down
