# Supplementary material for: De novo transcriptome analysis of Tibetan medicinal plant Dysphania schraderiana
Source: Genet Mol Biol. 2019 Jun 13;42(2):480–7. doi: 10.1590/1678-4685-GMB-2018-0033 (PMC6726160; doi:10.1590/1678-4685-GMB-2018-0033)
Supplement: Supplementary file 5 [file 1415-4757-GMB-1678-4685-GMB-2018-0033-20190513-suppl4.pdf]

Supplementary Material to "*De novo* transcriptome analysis of Tibetan medicinal plant *Dysphania schraderiana*"

Table S4 - KEGG annotation of DEGs.

PathWay	Pathway_definition	No.of DEGs
path:ko00940	Phenylpropanoid biosynthesis	159
path:ko00500	Starch and sucrose metabolism	113
path:ko00360	Phenylalanine metabolism	106
path:ko01200	Carbon metabolism	105
path:ko01230	Biosynthesis of amino acids	93
path:ko05145	Toxoplasmosis	79
path:ko05162	Measles	79
path:ko04075	Plant hormone signal transduction	74
path:ko05164	Influenza A	70
path:ko04141	Protein processing in endoplasmic reticulum	66
path:ko04626	Plant-pathogen interaction	60
path:ko00040	Pentose and glucuronate interconversions	58
path:ko03010	Ribosome	58
path:ko00010	Glycolysis / Gluconeogenesis	56
path:ko05169	Epstein-Barr virus infection	54
path:ko05133	Pertussis	54
path:ko00630	Glyoxylate and dicarboxylate metabolism	52
path:ko05152	Tuberculosis	51
path:ko00710	Carbon fixation in photosynthetic organisms	51
path:ko00480	Glutathione metabolism	50
path:ko05134	Legionellosis	49
path:ko00460	Cyanoamino acid metabolism	48
path:ko00190	Oxidative phosphorylation	48
path:ko04210	Apoptosis	46
path:ko04620	Toll-like receptor signaling pathway	46
path:ko05142	Chagas disease (American trypanosomiasis)	46
path:ko04064	NF-kappa B signaling pathway	46
path:ko04722	Neurotrophin signaling pathway	46
path:ko05140	Leishmaniasis	46
path:ko04612	Antigen processing and presentation	45
path:ko04144	Endocytosis	43
path:ko05204	Chemical carcinogenesis	43
path:ko00980	Metabolism of xenobiotics by cytochrome P450	43
path:ko00982	Drug metabolism - cytochrome P450	40
path:ko04111	Cell cycle - yeast	37
path:ko00680	Methane metabolism	37
path:ko04110	Cell cycle	36
path:ko04151	PI3K-Akt signaling pathway	35
path:ko05016	Huntington's disease	35
path:ko05034	Alcoholism	34

PathWay	Pathway_definition	No.of DEGs
path:ko04915	Estrogen signaling pathway	34
path:ko05322	Systemic lupus erythematosus	33
path:ko03040	Spliceosome	33
path:ko04010	MAPK signaling pathway	33
path:ko00030	Pentose phosphate pathway	32
path:ko00230	Purine metabolism	32
path:ko00941	Flavonoid biosynthesis	30
path:ko00330	Arginine and proline metabolism	30
path:ko05012	Parkinson's disease	29
path:ko00520	Amino sugar and nucleotide sugar metabolism	29
path:ko04146	Peroxisome	29
path:ko04152	AMPK signaling pathway	29
path:ko00270	Cysteine and methionine metabolism	29
path:ko00051	Fructose and mannose metabolism	28
path:ko00260	Glycine, serine and threonine metabolism	28
path:ko00195	Photosynthesis	27
path:ko04666	Fc gamma R-mediated phagocytosis	26
path:ko05010	Alzheimer's disease	26
path:ko04145	Phagosome	25
path:ko00240	Pyrimidine metabolism	25
path:ko00620	Pyruvate metabolism	25
path:ko00052	Galactose metabolism	25
path:ko04113	Meiosis - yeast	25
path:ko00945	Stilbenoid, diarylheptanoid and gingerol biosynthesis	24
path:ko03013	RNA transport	24
path:ko03030	DNA replication	24
path:ko04810	Regulation of actin cytoskeleton	24
path:ko00062	Fatty acid elongation	23
path:ko04932	Non-alcoholic fatty liver disease (NAFLD)	21
path:ko00053	Ascorbate and aldarate metabolism	21
path:ko04910	Insulin signaling pathway	21
path:ko00910	Nitrogen metabolism	21
path:ko05146	Amoebiasis	21
path:ko05203	Viral carcinogenesis	20
path:ko04974	Protein digestion and absorption	20
path:ko04922	Glucagon signaling pathway	20
path:ko04724	Glutamatergic synapse	20
path:ko00380	Tryptophan metabolism	19
path:ko04512	ECM-receptor interaction	19
path:ko04611	Platelet activation	19
path:ko04510	Focal adhesion	19
path:ko03440	Homologous recombination	18
path:ko04068	FoxO signaling pathway	18
path:ko05231	Choline metabolism in cancer	18
path:ko03430	Mismatch repair	17

PathWay	Pathway_definition	No.of DEGs
path:ko04727	GABAergic synapse	17
path:ko00562	Inositol phosphate metabolism	16
path:ko04142	Lysosome	16
path:ko04150	mTOR signaling pathway	16
path:ko00564	Glycerophospholipid metabolism	16
path:ko04070	Phosphatidylinositol signaling system	15
path:ko00561	Glycerolipid metabolism	15
path:ko00250	Alanine, aspartate and glutamate metabolism	15
path:ko04112	Cell cycle - Caulobacter	14
path:ko00130	Ubiquinone and other terpenoid-quinone biosynthesis	14
path:ko01212	Fatty acid metabolism	14
path:ko00960	Tropane, piperidine and pyridine alkaloid biosynthesis	14
path:ko05230	Central carbon metabolism in cancer	14
path:ko03420	Nucleotide excision repair	14
path:ko03460	Fanconi anemia pathway	14
path:ko03320	PPAR signaling pathway	14
path:ko04920	Adipocytokine signaling pathway	13
path:ko04914	Progesterone-mediated oocyte maturation	13
path:ko02020	Two-component system	13
path:ko00670	One carbon pool by folate	13
path:ko04540	Gap junction	13
path:ko05130	Pathogenic Escherichia coli infection	13
path:ko04721	Synaptic vesicle cycle	13
path:ko05410	Hypertrophic cardiomyopathy (HCM)	13
path:ko04710	Circadian rhythm	13
path:ko00073	Cutin, suberine and wax biosynthesis	13
path:ko04140	Regulation of autophagy	13
path:ko04921	Oxytocin signaling pathway	12
path:ko00340	Histidine metabolism	12
path:ko00900	Terpenoid backbone biosynthesis	11
path:ko03018	RNA degradation	11
path:ko01040	Biosynthesis of unsaturated fatty acids	11
path:ko04712	Circadian rhythm - plant	11
path:ko00906	Carotenoid biosynthesis	11
path:ko00280	Valine, leucine and isoleucine degradation	11
path:ko05202	Transcriptional misregulation in cancer	11
path:ko04071	Sphingolipid signaling pathway	11
path:ko05206	MicroRNAs in cancer	10
path:ko05166	HTLV-I infection	10
path:ko00196	Photosynthesis - antenna proteins	10
path:ko04066	HIF-1 signaling pathway	10
path:ko04973	Carbohydrate digestion and absorption	9
path:ko00903	Limonene and pinene degradation	9
path:ko04014	Ras signaling pathway	9
path:ko04024	cAMP signaling pathway	9

PathWay	Pathway_definition	No.of DEGs
path:ko04114	Oocyte meiosis	9
path:ko00565	Ether lipid metabolism	9
path:ko04912	GnRH signaling pathway	9
path:ko00400	Phenylalanine, tyrosine and tryptophan biosynthesis	9
path:ko00020	Citrate cycle (TCA cycle)	9
path:ko05131	Shigellosis	8
path:ko00071	Fatty acid degradation	8
path:ko04930	Type II diabetes mellitus	8
path:ko00410	beta-Alanine metabolism	8
path:ko04360	Axon guidance	8
path:ko05205	Proteoglycans in cancer	8
path:ko00100	Steroid biosynthesis	8
path:ko00350	Tyrosine metabolism	8
path:ko00592	alpha-Linolenic acid metabolism	8
path:ko00760	Nicotinate and nicotinamide metabolism	7
path:ko04130	SNARE interactions in vesicular transport	7
path:ko04120	Ubiquitin mediated proteolysis	7
path:ko00310	Lysine degradation	7
path:ko05132	Salmonella infection	7
path:ko00750	Vitamin B6 metabolism	7
path:ko04115	p53 signaling pathway	7
path:ko04962	Vasopressin-regulated water reabsorption	6
path:ko04340	Hedgehog signaling pathway	6
path:ko00904	Diterpenoid biosynthesis	6
path:ko03020	RNA polymerase	6
path:ko04020	Calcium signaling pathway	6
path:ko04919	Thyroid hormone signaling pathway	6
path:ko01051	Biosynthesis of ansamycins	6
path:ko04520	Adherens junction	6
path:ko04723	Retrograde endocannabinoid signaling	6
path:ko05033	Nicotine addiction	6
path:ko05100	Bacterial invasion of epithelial cells	6
path:ko05161	Hepatitis B	6
path:ko00625	Chloroalkane and chloroalkene degradation	6
path:ko00290	Valine, leucine and isoleucine biosynthesis	6
path:ko05032	Morphine addiction	6
path:ko00920	Sulfur metabolism	6
path:ko03070	Bacterial secretion system	5
path:ko00531	Glycosaminoglycan degradation	5
path:ko01210	2-Oxocarboxylic acid metabolism	5
path:ko00950	Isoquinoline alkaloid biosynthesis	5
path:ko00860	Porphyrin and chlorophyll metabolism	5
path:ko05014	Amyotrophic lateral sclerosis (ALS)	5
path:ko00401	Novobiocin biosynthesis	5
path:ko00140	Steroid hormone biosynthesis	5

PathWay	Pathway_definition	No.of DEGs
path:ko05200	Pathways in cancer	5
path:ko00061	Fatty acid biosynthesis	5
path:ko02010	ABC transporters	5
path:ko03410	Base excision repair	4
path:ko05020	Prion diseases	4
path:ko00909	Sesquiterpenoid and triterpenoid biosynthesis	4
path:ko00970	Aminoacyl-tRNA biosynthesis	4
path:ko04976	Bile secretion	4
path:ko00603	Glycosphingolipid biosynthesis - globo series	4
path:ko04260	Cardiac muscle contraction	4
path:ko00740	Riboflavin metabolism	4
path:ko04623	Cytosolic DNA-sensing pathway	4
path:ko00908	Zeatin biosynthesis	4
path:ko00604	Glycosphingolipid biosynthesis - ganglio series	4
path:ko00511	Other glycan degradation	4
path:ko00830	Retinol metabolism	3
path:ko00624	Polycyclic aromatic hydrocarbon degradation	3
path:ko05323	Rheumatoid arthritis	3
path:ko00591	Linoleic acid metabolism	3
path:ko00790	Folate biosynthesis	3
path:ko04012	ErbB signaling pathway	3
path:ko05110	Vibrio cholerae infection	3
path:ko05217	Basal cell carcinoma	3
path:ko04913	Ovarian steroidogenesis	3
path:ko03008	Ribosome biogenesis in eukaryotes	3
path:ko00627	Aminobenzoate degradation	3
path:ko04350	TGF-beta signaling pathway	3
path:ko00770	Pantothenate and CoA biosynthesis	3
path:ko05221	Acute myeloid leukemia	3
path:ko00363	Bisphenol degradation	3
path:ko03060	Protein export	2
path:ko03050	Proteasome	2
path:ko00450	Selenocompound metabolism	2
path:ko00261	Monobactam biosynthesis	2
path:ko04918	Thyroid hormone synthesis	2
path:ko04970	Salivary secretion	2
path:ko05210	Colorectal cancer	2
path:ko05340	Primary immunodeficiency	2
path:ko00905	Brassinosteroid biosynthesis	2
path:ko01220	Degradation of aromatic compounds	2
path:ko00232	Caffeine metabolism	2
path:ko04977	Vitamin digestion and absorption	1
path:ko04261	Adrenergic signaling in cardiomyocytes	1
path:ko00660	C5-Branched dibasic acid metabolism	1
path:ko00983	Drug metabolism - other enzymes	1

PathWay	Pathway_definition	No.of DEGs
path:ko00300	Lysine biosynthesis	1
path:ko04622	RIG-I-like receptor signaling pathway	1
path:ko00623	Toluene degradation	1
path:ko04310	Wnt signaling pathway	1
path:ko04668	TNF signaling pathway	1
path:ko00730	Thiamine metabolism	1
path:ko00364	Fluorobenzoate degradation	1
path:ko00902	Monoterpenoid biosynthesis	1
path:ko05414	Dilated cardiomyopathy	1
path:ko03015	mRNA surveillance pathway	1
path:ko00361	Chlorocyclohexane and chlorobenzene degradation	1
path:ko04390	Hippo signaling pathway	1
path:ko04015	Rap1 signaling pathway	1
path:ko00720	Carbon fixation pathways in prokaryotes	1
path:ko04978	Mineral absorption	1
path:ko00521	Streptomycin biosynthesis	1
