# Supplementary material for: De novo transcriptome analysis of Tibetan medicinal plant Dysphania schraderiana
Source: Genet Mol Biol. 2019 Jun 13;42(2):480–7. doi: 10.1590/1678-4685-GMB-2018-0033 (PMC6726160; doi:10.1590/1678-4685-GMB-2018-0033)
Supplement: Supplementary file 8 [file 1415-4757-GMB-1678-4685-GMB-2018-0033-20190513-suppl5.pdf]

Supplementary Material to "*De novo* transcriptome analysis of Tibetan medicinal plant *Dysphania schraderiana*"

Table S5 - List of predicted transcription factors.

TF Family	Family Type	Sequence Acc.	Fpkm of flower	Fpkm of leaf	significant
A20-like	putative novel transcription regulator	c27597_g1	2.243	4.046	no
A20-like	putative novel transcription regulator	c13695_g1	16.391	33.664	no
A20-like	putative novel transcription regulator	c14012_g1	155.415	81.472	no
A20-like	putative novel transcription regulator	c580_g1	12.013	5.21	no
A20-like	putative novel transcription regulator	c12728_g1	0.147	7.925	yes
A20-like	putative novel transcription regulator	c35013_g1	0	0	no
A20-like	putative novel transcription regulator	c8537_g2	0.774	0	no
A20-like	putative novel transcription regulator	c18040_g1	293.674	238.146	no
A20-like	putative novel transcription regulator	c30047_g1	125.496	193.066	no
A20-like	putative novel transcription regulator	c30829_g1	53.984	124.703	no
ABTB	chromatin remodeling & transcription regulator	c34809_g1	13.219	21.939	no
AP2-EREBP	transcription factor	c21353_g1	3.636	9.373	no
AP2-EREBP	transcription factor	c22208_g1	59.398	7.085	yes
AP2-EREBP	transcription factor	c22038_g1	22.03	12.45	no
AP2-EREBP	transcription factor	c20511_g1	2.8	1.5	no
AP2-EREBP	transcription factor	c20460_g1	1.075	2.288	no
AP2-EREBP	transcription factor	c7708_g2	6.049	3.4	no
AP2-EREBP	transcription factor	c28520_g1	0.828	1.189	no
AP2-EREBP	transcription factor	c31498_g1	0.572	6.645	yes
AP2-EREBP	transcription factor	c31380_g1	11.054	16.315	no
AP2-EREBP	transcription factor	c32255_g1	0.456	7.123	yes
AP2-EREBP	transcription factor	c11702_g1	12.709	10.394	no
AP2-EREBP	transcription factor	c28381_g1	0.727	1.383	no

TF Family	Family Type	Sequence Acc.	Fpkm of flower	Fpkm of leaf	significant
AP2-EREBP	transcription factor	c11808_g1	11.804	8.093	no
AP2-EREBP	transcription factor	c11927_g1	15.068	0.194	yes
AP2-EREBP	transcription factor	c13356_g1	1.624	0.776	no
AP2-EREBP	transcription factor	c29026_g1	1.709	0	no
AP2-EREBP	transcription factor	c14103_g1	28.589	30.756	no
AP2-EREBP	transcription factor	c964_g1	40.385	3.465	yes
AP2-EREBP	transcription factor	c529_g1	13.745	0.478	yes
AP2-EREBP	transcription factor	c9407_g1	18.038	33.871	no
AP2-EREBP	transcription factor	c6096_g1	2.924	9.774	no
AP2-EREBP	transcription factor	c6212_g1	17.296	5.843	no
AP2-EREBP	transcription factor	c23222_g1	22.95	36.353	no
AP2-EREBP	transcription factor	c12956_g1	19.887	1.616	yes
AP2-EREBP	transcription factor	c13122_g1	119.462	44.317	no
AP2-EREBP	transcription factor	c10319_g1	13.846	1.357	no
AP2-EREBP	transcription factor	c10319_g2	6.637	0.259	yes
AP2-EREBP	transcription factor	c1487_g1	12.887	17.117	no
AP2-EREBP	transcription factor	c11126_g1	58.532	86.165	no
AP2-EREBP	transcription factor	c36302_g1	6.931	2.405	no
AP2-EREBP	transcription factor	c36829_g1	42.373	25.546	no
AP2-EREBP	transcription factor	c2504_g1	478.823	247.531	no
AP2-EREBP	transcription factor	c32896_g1	13.15	15.759	no
AP2-EREBP	transcription factor	c6374_g1	46.256	4.279	yes
AP2-EREBP	transcription factor	c32661_g1	14.627	30.626	no
AP2-EREBP	transcription factor	c4195_g1	103.605	94.154	no
AP2-EREBP	transcription factor	c34505_g1	14.364	11.506	no

TF Family	Family Type	Sequence Acc.	Fpkm of flower	Fpkm of leaf	significant
AP2-EREBP	transcription factor	c34418_g1	109.329	299.398	no
AP2-EREBP	transcription factor	c26693_g1	3.001	33.729	yes
AP2-EREBP	transcription factor	c6742_g1	30.02	13.393	no
AP2-EREBP	transcription factor	c6246_g1	8.547	4.667	no
AP2-EREBP	transcription factor	c23389_g1	22.78	40.193	no
AP2-EREBP	transcription factor	c8996_g1	13.521	1.086	yes
AP2-EREBP	transcription factor	c9091_g1	12.013	12.45	no
AP2-EREBP	transcription factor	c18779_g1	2.15	0.608	no
AP2-EREBP	transcription factor	c19497_g1	59.669	28.765	no
AP2-EREBP	transcription factor	c19724_g1	2.87	1.241	no
AP2-EREBP	transcription factor	c19545_g1	2.351	0	no
AP2-EREBP	transcription factor	c17692_g1	186.062	670.146	no
AP2-EREBP	transcription factor	c17876_g1	0.882	3.297	no
AP2-EREBP	transcription factor	c17728_g1	2.081	21.628	yes
AP2-EREBP	transcription factor	c18093_g1	1.191	7.783	no
AP2-EREBP	transcription factor	c7023_g1	95.932	6.503	yes
AP2-EREBP	transcription factor	c25757_g1	1.137	0.814	no
AP2-EREBP	transcription factor	c25717_g1	8.045	0	no
AP2-EREBP	transcription factor	c37578_g1	2.731	0.349	no
AP2-EREBP	transcription factor	c36224_g1	2.986	2.909	no
AP2-EREBP	transcription factor	c14838_g1	48.414	14.014	no
AP2-EREBP	transcription factor	c14628_g1	2.754	3.891	no
AP2-EREBP	transcription factor	c14780_g1	60.234	6.8	yes
AP2-EREBP	transcription factor	c14379_g1	3.512	4.344	no
ARF	transcription factor	c21703_g1	63.993	62.235	no

TF Family	Family Type	Sequence Acc.	Fpkm of flower	Fpkm of leaf	significant
ARF	transcription factor	c266_g1	37.98	19.353	no
ARF	transcription factor	c9519_g1	36.657	13.329	no
ARF	transcription factor	c5995_g1	5.407	17.078	no
ARF	transcription factor	c12399_g1	18.758	6.361	no
ARF	transcription factor	c12399_g2	0.131	6.024	yes
ARF	transcription factor	c12399_g3	9.87	6.296	no
ARF	transcription factor	c3928_g1	44.47	78.886	no
ARF	transcription factor	c1607_g1	6.513	2.068	no
ARF	transcription factor	c2579_g1	13.343	13.704	no
ARF	transcription factor	c4393_g1	12.956	16.044	no
ARF	transcription factor	c34758_g1	49.528	46.308	no
ARF	transcription factor	c17675_g1	69.601	30.975	no
ARF	transcription factor	c30336_g1	46.713	58.266	no
ARID	transcription factor & chromatin remodeling	c7534_g1	21.744	6.115	no
ARID	transcription factor & chromatin remodeling	c8027_g1	57.147	14.208	no
ARID	transcription factor & chromatin remodeling	c9041_g1	48.763	34.776	no
ARID	transcription factor & chromatin remodeling	c18471_g1	1.601	1.9	no
ARID	transcription factor & chromatin remodeling	c19102_g1	2.367	1.164	no
ARID	transcription factor & chromatin remodeling	c7018_g1	13.792	17.647	no
ARID-HMG	transcription factor & chromatin remodeling	c11893_g2	26.756	38.9	no
ARID-HMG	transcription factor & chromatin remodeling	c5253_g1	6.575	83.178	yes
AS2-LOB	transcription factor	c22180_g1	5.422	0.879	no
AS2-LOB	transcription factor	c21993_g1	1.485	0.608	no
AS2-LOB	transcription factor	c7547_g1	58.524	71.065	no
AS2-LOB	transcription factor	c31964_g1	2.228	0	no

TF Family	Family Type	Sequence Acc.	Fpkm of flower	Fpkm of leaf	significant
AS2-LOB	transcription factor	c31564_g1	6.32	0	yes
AS2-LOB	transcription factor	c11544_g1	9.909	15.578	no
AS2-LOB	transcription factor	c9961_g1	4.804	4.99	no
AS2-LOB	transcription factor	c5501_g1	3.133	0	no
AS2-LOB	transcription factor	c5489_g1	7.689	0.698	yes
AS2-LOB	transcription factor	c6077_g1	3.156	4.512	no
AS2-LOB	transcription factor	c22970_g1	35.102	4.538	no
AS2-LOB	transcription factor	c3623_g1	27.328	0.763	yes
AS2-LOB	transcription factor	c38504_g1	2.413	0	no
AS2-LOB	transcription factor	c18346_g1	9.197	12.256	no
AS2-LOB	transcription factor	c35447_g1	0.24	3.723	yes
AS2-LOB	transcription factor	c36171_g1	10.775	16.987	no
AS2-LOB	transcription factor	c17053_g1	1.276	0	no
AUX-IAA	transcription factor interactor and regulator	c7970_g1	19.307	34.634	no
AUX-IAA	transcription factor interactor and regulator	c28864_g1	0	2.262	no
AUX-IAA	transcription factor interactor and regulator	c13777_g1	60.837	73.004	no
AUX-IAA	transcription factor interactor and regulator	c410_g1	29.502	41.279	no
AUX-IAA	transcription factor interactor and regulator	c621_g1	1.609	2.418	no
AUX-IAA	transcription factor interactor and regulator	c5924_g2	210.25	48.751	no
AUX-IAA	transcription factor interactor and regulator	c5689_g1	26.818	32.721	no
AUX-IAA	transcription factor interactor and regulator	c1909_g1	13.769	50.393	no
AUX-IAA	transcription factor interactor and regulator	c2270_g1	10.296	0.659	no
AUX-IAA	transcription factor interactor and regulator	c2961_g1	36.928	44.731	no
AUX-IAA	transcription factor interactor and regulator	c1708_g1	127.236	34.453	no
AUX-IAA	transcription factor interactor and regulator	c34941_g1	8.253	28.622	no

TF Family	Family Type	Sequence Acc.	Fpkm of flower	Fpkm of leaf	significant
AUX-IAA	transcription factor interactor and regulator	c34383_g1	181.065	246.859	no
AUX-IAA	transcription factor interactor and regulator	c34930_g1	3.365	21.137	no
AUX-IAA	transcription factor interactor and regulator	c26450_g1	55.067	31.389	no
AUX-IAA	transcription factor interactor and regulator	c8535_g1	14.689	26.451	no
AUX-IAA	transcription factor interactor and regulator	c20263_g1	5.77	3.633	no
AUX-IAA	transcription factor interactor and regulator	c18053_g1	165.95	38.797	no
AUX-IAA	transcription factor interactor and regulator	c35386_g1	51.416	6.322	no
AUX-IAA	transcription factor interactor and regulator	c16423_g1	25.085	40.258	no
B3-Domain	transcription factor	c21939_g1	2.019	0.827	no
B3-Domain	transcription factor	c20891_g1	3.496	1.112	no
B3-Domain	transcription factor	c7915_g1	26.833	0.944	yes
B3-Domain	transcription factor	c8046_g1	5.709	5.288	no
B3-Domain	transcription factor	c12144_g1	9.886	3.555	no
B3-Domain	transcription factor	c28949_g1	2.228	0	no
B3-Domain	transcription factor	c11888_g1	10.76	2.482	no
B3-Domain	transcription factor	c11888_g2	16.182	0.802	yes
B3-Domain	transcription factor	c11888_g3	30.012	1.06	yes
B3-Domain	transcription factor	c11661_g1	2.104	2.314	no
B3-Domain	transcription factor	c11820_g1	18.278	0.621	yes
B3-Domain	transcription factor	c11787_g1	5.314	12.075	no
B3-Domain	transcription factor	c430_g1	1.098	1.021	no
B3-Domain	transcription factor	c10177_g1	22.386	0.918	yes
B3-Domain	transcription factor	c6171_g1	14.89	2.198	no
B3-Domain	transcription factor	c6117_g1	9.514	0.31	yes
B3-Domain	transcription factor	c22778_g1	0.201	1.422	no

TF Family	Family Type	Sequence Acc.	Fpkm of flower	Fpkm of leaf	significant
B3-Domain	transcription factor	c1391_g1	1.439	6.192	no
B3-Domain	transcription factor	c12644_g1	6.969	0.659	no
B3-Domain	transcription factor	c12643_g1	12.268	1.991	no
B3-Domain	transcription factor	c12944_g1	18.58	1.862	no
B3-Domain	transcription factor	c10536_g1	6.745	1.021	no
B3-Domain	transcription factor	c10465_g1	25.039	0.724	yes
B3-Domain	transcription factor	c11091_g1	9.46	4.835	no
B3-Domain	transcription factor	c4134_g1	4.022	0.569	no
B3-Domain	transcription factor	c3198_g1	3.264	0	no
B3-Domain	transcription factor	c2855_g1	18.92	0.621	yes
B3-Domain	transcription factor	c3077_g1	6.235	0	yes
B3-Domain	transcription factor	c3055_g1	40.122	3.995	yes
B3-Domain	transcription factor	c2986_g1	14.937	2.185	no
B3-Domain	transcription factor	c2176_g1	1.446	0	no
B3-Domain	transcription factor	c33098_g1	2.073	0	no
B3-Domain	transcription factor	c4581_g1	2.498	0	no
B3-Domain	transcription factor	c4605_g1	3.628	0	yes
B3-Domain	transcription factor	c35247_g1	1.872	2.094	no
B3-Domain	transcription factor	c6280_g1	20.127	1.073	yes
B3-Domain	transcription factor	c34867_g1	2.576	0	no
B3-Domain	transcription factor	c26558_g1	8.222	1.073	no
B3-Domain	transcription factor	c26878_g1	8.671	0.246	yes
B3-Domain	transcription factor	c26429_g1	3.272	0.271	no
B3-Domain	transcription factor	c15747_g1	0.379	2.43	no
B3-Domain	transcription factor	c15764_g1	2.847	0	no

TF Family	Family Type	Sequence Acc.	Fpkm of flower	Fpkm of leaf	significant
B3-Domain	transcription factor	c16235_g1	2.344	0	no
B3-Domain	transcription factor	c9103_g1	14.086	0.065	yes
B3-Domain	transcription factor	c8964_g1	6.459	3.529	no
B3-Domain	transcription factor	c9032_g2	15.06	6.477	no
B3-Domain	transcription factor	c8843_g1	25.116	3.659	no
B3-Domain	transcription factor	c9117_g1	11.332	0.284	yes
B3-Domain	transcription factor	c18780_g1	4.881	0	yes
B3-Domain	transcription factor	c25893_g1	3.527	0	no
B3-Domain	transcription factor	c30559_g1	3.071	0.621	no
B3-Domain	transcription factor	c30184_g1	45.978	2.249	yes
B3-Domain	transcription factor	c17098_g1	6.474	0	no
BED-type(Zn)	transcription factor/ chromatin remodeling	c12072_g1	14.356	15.164	no
BED-type(Zn)	transcription factor/ chromatin remodeling	c11498_g1	8.184	4.021	no
BED-type(Zn)	transcription factor/ chromatin remodeling	c10075_g2	4.1	4.279	no
BED-type(Zn)	transcription factor/ chromatin remodeling	c5367_g1	7.488	2.288	no
BED-type(Zn)	transcription factor/ chromatin remodeling	c22634_g1	3.295	2.418	no
BED-type(Zn)	transcription factor/ chromatin remodeling	c10523_g1	13.366	7.24	no
BED-type(Zn)	transcription factor/ chromatin remodeling	c1994_g1	14.983	9.295	no
BED-type(Zn)	transcription factor/ chromatin remodeling	c9137_g1	8.346	3.361	no
BED-type(Zn)	transcription factor/ chromatin remodeling	c18209_g1	46.326	21.305	no
BES/BZR	transcription factor	c3385_g1	2.104	2.767	no
BES/BZR	transcription factor	c2155_g1	2.143	1.332	no
BES/BZR	transcription factor	c34425_g1	21.983	20.504	no
BES/BZR	transcription factor	c34561_g1	58.254	52.811	no
BES/BZR	transcription factor	c26055_g1	37.338	38.9	no

TF Family	Family Type	Sequence Acc.	Fpkm of flower	Fpkm of leaf	significant
bHLH	transcription factor	c22105_g1	2.112	0	no
bHLH	transcription factor	c20632_g1	1.037	8.416	yes
bHLH	transcription factor	c30226_g1	38.56	88.259	no
bHLH	transcription factor	c7477_g1	61.239	14.285	no
bHLH	transcription factor	c7912_g1	2.622	24.201	yes
bHLH	transcription factor	c8049_g1	12.941	17.311	no
bHLH	transcription factor	c8008_g1	13.722	33.031	no
bHLH	transcription factor	c7667_g1	9.553	6.658	no
bHLH	transcription factor	c31494_g1	25.039	14.932	no
bHLH	transcription factor	c31357_g1	1.942	0	no
bHLH	transcription factor	c28885_g1	1.276	0	no
bHLH	transcription factor	c11696_g1	13.475	1.06	yes
bHLH	transcription factor	c11948_g1	7.55	0.672	no
bHLH	transcription factor	c11948_g3	34.22	0.892	yes
bHLH	transcription factor	c27924_g1	1.145	0.827	no
bHLH	transcription factor	c28011_g1	0.487	1.409	no
bHLH	transcription factor	c27681_g1	3.009	11.402	no
bHLH	transcription factor	c28010_g1	2.529	5.456	no
bHLH	transcription factor	c13679_g1	39.155	60.723	no
bHLH	transcription factor	c14252_g1	35.272	56.922	no
bHLH	transcription factor	c14022_g1	19.701	23.49	no
bHLH	transcription factor	c13344_g1	35.535	55.138	no
bHLH	transcription factor	c911_g1	13.49	19.03	no
bHLH	transcription factor	c191_g1	13.297	0.44	yes
bHLH	transcription factor	c440_g1	0	4.447	no

TF Family	Family Type	Sequence Acc.	Fpkm of flower	Fpkm of leaf	significant
bHLH	transcription factor	c10230_g1	13.018	1.952	no
bHLH	transcription factor	c5802_g1	3.017	25.029	yes
bHLH	transcription factor	c1216_g1	8.176	0	yes
bHLH	transcription factor	c5803_g1	22.44	10.006	no
bHLH	transcription factor	c22937_g1	5.67	34.854	no
bHLH	transcription factor	c22965_g1	26.702	28.17	no
bHLH	transcription factor	c13010_g3	0.572	3.878	no
bHLH	transcription factor	c13059_g1	6.451	7.757	no
bHLH	transcription factor	c12899_g1	9.491	33.561	no
bHLH	transcription factor	c12510_g1	1.276	81.317	yes
bHLH	transcription factor	c10407_g1	16.035	7.472	no
bHLH	transcription factor	c1827_g1	0.394	46.618	yes
bHLH	transcription factor	c10675_g1	13.861	3.116	no
bHLH	transcription factor	c11271_g1	27.181	12.269	no
bHLH	transcription factor	c10778_g1	4.486	12.45	no
bHLH	transcription factor	c3974_g1	6.969	11.48	no
bHLH	transcription factor	c3968_g1	4.858	1.112	no
bHLH	transcription factor	c3490_g1	31.993	16.367	no
bHLH	transcription factor	c36445_g1	4.293	1.06	no
bHLH	transcription factor	c36819_g1	7.186	0	yes
bHLH	transcription factor	c1516_g1	31.915	15.307	no
bHLH	transcription factor	c3053_g1	22.27	19.961	no
bHLH	transcription factor	c1277_g1	10.551	2.754	no
bHLH	transcription factor	c2119_g1	21.202	64.252	no
bHLH	transcription factor	c2591_g1	38.397	12.475	no

TF Family	Family Type	Sequence Acc.	Fpkm of flower	Fpkm of leaf	significant
bHLH	transcription factor	c2727_g1	3.017	0	yes
bHLH	transcription factor	c3030_g1	11.255	24.912	no
bHLH	transcription factor	c4444_g1	12.253	79.093	no
bHLH	transcription factor	c4959_g1	25.774	29.463	no
bHLH	transcription factor	c4615_g1	42.203	55.707	no
bHLH	transcription factor	c4427_g1	30.616	17.802	no
bHLH	transcription factor	c4776_g1	17.652	13.923	no
bHLH	transcription factor	c4892_g1	2.22	0	no
bHLH	transcription factor	c4598_g1	11.959	2.301	no
bHLH	transcription factor	c35200_g1	77.476	122.919	no
bHLH	transcription factor	c34881_g1	40.385	27.989	no
bHLH	transcription factor	c26848_g1	34.948	16.173	no
bHLH	transcription factor	c26592_g1	3.264	154.218	yes
bHLH	transcription factor	c26596_g1	13.451	11.364	no
bHLH	transcription factor	c15950_g1	1.238	0	no
bHLH	transcription factor	c6270_g1	11.061	12.269	no
bHLH	transcription factor	c6337_g1	22.973	7.485	no
bHLH	transcription factor	c24179_g1	2.514	0.879	no
bHLH	transcription factor	c6723_g1	9.947	6.412	no
bHLH	transcription factor	c8686_g2	6.761	2.611	no
bHLH	transcription factor	c9064_g1	6.436	1.306	no
bHLH	transcription factor	c8914_g1	12.686	8.636	no
bHLH	transcription factor	c8753_g1	10.62	21.797	no
bHLH	transcription factor	c9045_g1	5.453	18.888	no
bHLH	transcription factor	c6852_g1	9.739	0.866	no

TF Family	Family Type	Sequence Acc.	Fpkm of flower	Fpkm of leaf	significant
bHLH	transcription factor	c18545_g1	84.901	13.652	no
bHLH	transcription factor	c6968_g1	21.767	0.931	yes
bHLH	transcription factor	c17394_g1	0.951	1.81	no
bHLH	transcription factor	c17816_g1	45.56	48.118	no
bHLH	transcription factor	c18126_g1	35.249	39.366	no
bHLH	transcription factor	c17985_g1	22.796	18.345	no
bHLH	transcription factor	c26167_g1	24.521	28.791	no
bHLH	transcription factor	c37790_g1	0	2.831	no
bHLH	transcription factor	c37979_g1	1.222	1.306	no
bHLH	transcription factor	c35723_g1	2.483	2.909	no
bHLH	transcription factor	c24867_g1	2.56	0	no
bHLH	transcription factor	c30513_g1	14.387	18.384	no
bHLH	transcription factor	c30105_g1	11.595	19.34	no
bHLH	transcription factor	c16773_g1	4.231	0.22	no
bHLH	transcription factor	c14351_g1	0.766	4.202	no
bHLH	transcription factor	c14615_g1	117.776	73.482	no
bHLH	transcription factor	c15011_g1	37.461	30.251	no
bHLH	transcription factor	c14548_g1	8.184	36.017	no
Bromodomain	chromatin remodeler	c22116_g1	9.677	12.475	no
Bromodomain	chromatin remodeler	c21759_g1	18.642	20.439	no
Bromodomain	chromatin remodeler	c7916_g1	22.231	8.726	no
Bromodomain	chromatin remodeler	c11812_g1	8.826	5.779	no
Bromodomain	chromatin remodeler	c11295_g1	12.809	12.734	no
Bromodomain	chromatin remodeler	c6770_g1	40.757	88.737	no
Bromodomain	chromatin remodeler	c10114_g1	8.339	5.96	no

TF Family	Family Type	Sequence Acc.	Fpkm of flower	Fpkm of leaf	significant
Bromodomain	chromatin remodeler	c10235_g1	4.061	10.588	no
Bromodomain	chromatin remodeler	c5598_g1	2.189	11.079	no
Bromodomain	chromatin remodeler	c22295_g1	15.277	20.426	no
Bromodomain	chromatin remodeler	c22457_g1	3.976	17.763	no
Bromodomain	chromatin remodeler	c12937_g2	12.5	9.774	no
Bromodomain	chromatin remodeler	c12690_g2	2.645	7.821	no
Bromodomain	chromatin remodeler	c1292_g1	25.201	28.868	no
Bromodomain	chromatin remodeler	c11061_g1	10.458	9.334	no
Bromodomain	chromatin remodeler	c10457_g1	18.634	10.666	no
Bromodomain	chromatin remodeler	c3506_g1	28.93	25.3	no
Bromodomain	chromatin remodeler	c2975_g1	15.927	17.75	no
Bromodomain	chromatin remodeler	c8571_g1	6.142	3.814	no
Bromodomain	chromatin remodeler	c19787_g1	27.29	28.454	no
Bromodomain	chromatin remodeler	c17806_g1	1.802	6.542	no
BTB-POZ	chromatin remodeling & transcription regulator	c7618_g2	8.888	7.033	no
BTB-POZ	chromatin remodeling & transcription regulator	c7618_g3	1.856	2.986	no
BTB-POZ	chromatin remodeling & transcription regulator	c7536_g1	8.184	16.832	no
BTB-POZ	chromatin remodeling & transcription regulator	c7848_g1	30.175	6.632	no
BTB-POZ	chromatin remodeling & transcription regulator	c7380_g1	10.69	3.646	no
BTB-POZ	chromatin remodeling & transcription regulator	c22473_g1	35.528	40.038	no
BTB-POZ	chromatin remodeling & transcription regulator	c22856_g1	0.82	2.34	no
BTB-POZ	chromatin remodeling & transcription regulator	c12368_g2	19.833	28.739	no
BTB-POZ	chromatin remodeling & transcription regulator	c12427_g1	7.998	9.45	no
BTB-POZ	chromatin remodeling & transcription regulator	c10361_g1	12.941	7.653	no
BTB-POZ	chromatin remodeling & transcription regulator	c10661_g1	1.006	4.034	no

TF Family	Family Type	Sequence Acc.	Fpkm of flower	Fpkm of leaf	significant
BTB-POZ	chromatin remodeling & transcription regulator	c10665_g1	8.13	2.327	no
BTB-POZ	chromatin remodeling & transcription regulator	c3128_g1	25.928	18.138	no
BTB-POZ	chromatin remodeling & transcription regulator	c36766_g1	2.127	1.034	no
BTB-POZ	chromatin remodeling & transcription regulator	c36858_g1	1.106	1.59	no
BTB-POZ	chromatin remodeling & transcription regulator	c4919_g1	19.106	5.675	no
BTB-POZ	chromatin remodeling & transcription regulator	c35144_g1	10.497	16.418	no
BTB-POZ	chromatin remodeling & transcription regulator	c26335_g1	13.243	15.888	no
BTB-POZ	chromatin remodeling & transcription regulator	c9158_g1	26.594	47.407	no
BTB-POZ	chromatin remodeling & transcription regulator	c8756_g1	6.049	7.64	no
BTB-POZ	chromatin remodeling & transcription regulator	c8448_g1	12.013	25.597	no
BTB-POZ	chromatin remodeling & transcription regulator	c9007_g1	16.313	17.375	no
BTB-POZ	chromatin remodeling & transcription regulator	c8393_g1	14.372	8.765	no
BTB-POZ	chromatin remodeling & transcription regulator	c8841_g1	3.512	40.801	yes
BTB-POZ	chromatin remodeling & transcription regulator	c26229_g1	51.88	47.82	no
BTB-POZ	chromatin remodeling & transcription regulator	c25977_g1	20.637	25.184	no
BTB-POZ	chromatin remodeling & transcription regulator	c36080_g1	0.642	2.792	no
BTB-POZ	chromatin remodeling & transcription regulator	c24737_g1	4.131	1.487	no
BTB-POZ	chromatin remodeling & transcription regulator	c30121_g1	45.483	24.072	no
BTB-POZ	chromatin remodeling & transcription regulator	c30487_g1	28.233	21.137	no
BTB-POZ	chromatin remodeling & transcription regulator	c17115_g1	1.872	1.81	no
BTB-POZ-MA	chromatin remodeling & transcription regulator	c21977_g1	62.732	46.566	no
BTB-POZ-MA	chromatin remodeling & transcription regulator	c13966_g1	16.646	45.946	no
BTB-POZ-MA	chromatin remodeling & transcription regulator	c6637_g1	26.199	32.63	no
bZIP	transcription factor	c21808_g1	13.776	11.687	no
bZIP	transcription factor	c7262_g1	49.838	5.145	yes

TF Family	Family Type	Sequence Acc.	Fpkm of flower	Fpkm of leaf	significant
bZIP	transcription factor	c7262_g2	21.434	2.275	yes
bZIP	transcription factor	c11982_g1	9.452	2.508	no
bZIP	transcription factor	c11529_g3	3.589	5.068	no
bZIP	transcription factor	c27349_g1	0	3.206	no
bZIP	transcription factor	c14042_g1	28.752	44.291	no
bZIP	transcription factor	c14068_g1	22.146	16.845	no
bZIP	transcription factor	c13429_g1	20.506	33.496	no
bZIP	transcription factor	c13424_g2	3.14	80.373	yes
bZIP	transcription factor	c823_g1	26.184	7.059	no
bZIP	transcription factor	c793_g1	0.433	3.594	no
bZIP	transcription factor	c9982_g2	21.999	25.455	no
bZIP	transcription factor	c9507_g1	22.486	5.081	no
bZIP	transcription factor	c5934_g2	30.469	38.564	no
bZIP	transcription factor	c22277_g1	5.376	0.698	no
bZIP	transcription factor	c22877_g1	30.848	26.851	no
bZIP	transcription factor	c12404_g6	22.099	36.664	no
bZIP	transcription factor	c12430_g1	15.323	1.771	yes
bZIP	transcription factor	c13107_g9	54.548	35.707	no
bZIP	transcription factor	c10924_g3	22.695	24.175	no
bZIP	transcription factor	c10992_g1	4.138	1.047	no
bZIP	transcription factor	c10331_g1	21.945	27.239	no
bZIP	transcription factor	c7092_g1	51.849	85.234	no
bZIP	transcription factor	c3447_g1	1.091	4.305	no
bZIP	transcription factor	c3773_g1	22.393	14.208	no
bZIP	transcription factor	c3028_g1	16.329	34.543	no

TF Family	Family Type	Sequence Acc.	Fpkm of flower	Fpkm of leaf	significant
bZIP	transcription factor	c2952_g1	2.429	20.051	yes
bZIP	transcription factor	c3107_g1	24.126	5.74	no
bZIP	transcription factor	c4679_g1	0.92	9.502	yes
bZIP	transcription factor	c4422_g1	15.687	3.529	no
bZIP	transcription factor	c34530_g1	121.21	62.533	no
bZIP	transcription factor	c34812_g1	43.75	84.432	no
bZIP	transcription factor	c34804_g1	8.176	26.089	no
bZIP	transcription factor	c34566_g1	18.201	18.409	no
bZIP	transcription factor	c6283_g1	63.367	112.008	no
bZIP	transcription factor	c6361_g1	26.23	19.831	no
bZIP	transcription factor	c26527_g1	7.016	0.207	yes
bZIP	transcription factor	c15310_g1	2.645	1.138	no
bZIP	transcription factor	c23330_g1	0.843	1.241	no
bZIP	transcription factor	c9042_g1	12.647	8.364	no
bZIP	transcription factor	c18373_g1	9.971	3.995	no
bZIP	transcription factor	c18188_g1	1.942	41.744	yes
bZIP	transcription factor	c17922_g1	49.358	21.745	no
bZIP	transcription factor	c37484_g1	5.159	52.229	yes
bZIP	transcription factor	c35887_g1	2.452	4.085	no
bZIP	transcription factor	c24319_g1	2.746	1.474	no
bZIP	transcription factor	c30587_g1	81.506	38.176	no
bZIP	transcription factor	c31163_g1	1.88	1.383	no
bZIP	transcription factor	c30936_g1	10.945	10.006	no
bZIP	transcription factor	c30319_g1	2.506	0	no
bZIP	transcription factor	c16857_g1	1.261	0	no

TF Family	Family Type	Sequence Acc.	Fpkm of flower	Fpkm of leaf	significant
bZIP	transcription factor	c14646_g1	43.92	20.271	no
C2C2-CO-like	transcription factor	c22035_g1	3.465	2.107	no
C2C2-CO-like	transcription factor	c7540_g1	19.09	38.318	no
C2C2-CO-like	transcription factor	c11747_g1	7.712	6.218	no
C2C2-CO-like	transcription factor	c12190_g1	22.324	3.361	no
C2C2-CO-like	transcription factor	c9727_g1	47.888	62.3	no
C2C2-CO-like	transcription factor	c9468_g1	7.642	86.01	yes
C2C2-CO-like	transcription factor	c5906_g1	6.691	11.17	no
C2C2-CO-like	transcription factor	c10357_g1	9.522	8.158	no
C2C2-CO-like	transcription factor	c3907_g1	70.305	746.68	yes
C2C2-CO-like	transcription factor	c36914_g1	1.238	2.611	no
C2C2-CO-like	transcription factor	c3048_g1	21.256	75.706	no
C2C2-CO-like	transcription factor	c2751_g1	9.251	14.26	no
C2C2-CO-like	transcription factor	c32541_g1	4.015	160.216	yes
C2C2-CO-like	transcription factor	c6876_g1	10.86	1.073	yes
C2C2-CO-like	transcription factor	c26385_g1	19.114	22.055	no
C2C2-CO-like	transcription factor	c17950_g1	193.991	357.057	no
C2C2-CO-like	transcription factor	c6914_g1	24.722	77.141	no
C2C2-CO-like	transcription factor	c26018_g1	0.487	2.392	no
C2C2-Dof	transcription factor	c8163_g1	3.171	15.229	no
C2C2-Dof	transcription factor	c8163_g2	0.541	4.874	no
C2C2-Dof	transcription factor	c30035_g1	42.853	149.667	no
C2C2-Dof	transcription factor	c8188_g1	40.207	22.844	no
C2C2-Dof	transcription factor	c8188_g2	12.013	10.459	no
C2C2-Dof	transcription factor	c7758_g1	3.512	10.148	no

TF Family	Family Type	Sequence Acc.	Fpkm of flower	Fpkm of leaf	significant
C2C2-Dof	transcription factor	c27593_g1	3.357	2.831	no
C2C2-Dof	transcription factor	c28232_g1	20.521	3.038	no
C2C2-Dof	transcription factor	c13424_g1	19.121	39.909	no
C2C2-Dof	transcription factor	c13854_g1	60.342	1.06	yes
C2C2-Dof	transcription factor	c514_g1	9.978	10.148	no
C2C2-Dof	transcription factor	c9509_g2	6.567	6.438	no
C2C2-Dof	transcription factor	c10249_g1	29.78	31.803	no
C2C2-Dof	transcription factor	c10249_g2	9.097	21.163	no
C2C2-Dof	transcription factor	c22668_g1	9.437	104.057	yes
C2C2-Dof	transcription factor	c22770_g1	37.229	12.282	no
C2C2-Dof	transcription factor	c4068_g1	2.854	18.758	no
C2C2-Dof	transcription factor	c4911_g1	1.702	26.283	yes
C2C2-Dof	transcription factor	c34972_g1	13.096	99.403	yes
C2C2-Dof	transcription factor	c26649_g1	13.204	26.683	no
C2C2-Dof	transcription factor	c9093_g2	54.634	72.952	no
C2C2-Dof	transcription factor	c18528_g1	7.263	39.909	no
C2C2-Dof	transcription factor	c18212_g1	2.104	11.157	no
C2C2-Dof	transcription factor	c35799_g1	29.347	10.239	no
C2C2-Dof	transcription factor	c17060_g1	2.119	2.301	no
C2C2-GATA	transcription factor	c32134_g1	0.526	1.655	no
C2C2-GATA	transcription factor	c12225_g1	9.762	13.484	no
C2C2-GATA	transcription factor	c11545_g1	11.742	3.84	no
C2C2-GATA	transcription factor	c9863_g1	4.44	39.857	yes
C2C2-GATA	transcription factor	c9446_g1	5.917	6.671	no
C2C2-GATA	transcription factor	c5394_g1	20.142	22.753	no

TF Family	Family Type	Sequence Acc.	Fpkm of flower	Fpkm of leaf	significant
C2C2-GATA	transcription factor	c5401_g1	63.398	74.995	no
C2C2-GATA	transcription factor	c5498_g1	24.242	49.217	no
C2C2-GATA	transcription factor	c22318_g1	88.382	3.245	yes
C2C2-GATA	transcription factor	c12933_g3	4.92	12.191	no
C2C2-GATA	transcription factor	c11085_g1	21.071	14.26	no
C2C2-GATA	transcription factor	c11031_g1	9.197	2.818	no
C2C2-GATA	transcription factor	c10716_g1	21.133	30.937	no
C2C2-GATA	transcription factor	c10558_g1	16.708	1.086	yes
C2C2-GATA	transcription factor	c1083_g1	57.913	36.147	no
C2C2-GATA	transcription factor	c2069_g1	1.942	0	no
C2C2-GATA	transcription factor	c5151_g1	49.83	36.573	no
C2C2-GATA	transcription factor	c34569_g1	122.146	133.171	no
C2C2-GATA	transcription factor	c26894_g1	27.282	41.305	no
C2C2-GATA	transcription factor	c9112_g1	8.671	22.133	no
C2C2-YABBY	transcription factor	c3463_g1	49.582	85.118	no
C2C2-YABBY	transcription factor	c3972_g1	72.131	2.056	yes
C2C2-YABBY	transcription factor	c4955_g1	38.916	5.262	no
C2C2-YABBY	transcription factor	c8558_g2	67.095	165.348	no
C2C2-YABBY	transcription factor	c6867_g1	37.129	97.748	no
C2H2	transcription factor	c21798_g1	40.022	36.509	no
C2H2	transcription factor	c22122_g1	32.82	35.836	no
C2H2	transcription factor	c21630_g1	0	3.4	no
C2H2	transcription factor	c21727_g1	28.063	15.152	no
C2H2	transcription factor	c21853_g1	15.818	29.127	no
C2H2	transcription factor	c21828_g1	1.199	39.12	yes

TF Family	Family Type	Sequence Acc.	Fpkm of flower	Fpkm of leaf	significant
C2H2	transcription factor	c20520_g1	0.681	1.991	no
C2H2	transcription factor	c20938_g1	3.063	0	no
C2H2	transcription factor	c7968_g1	12.825	26.489	no
C2H2	transcription factor	c7997_g1	28.179	42.468	no
C2H2	transcription factor	c7767_g1	3.349	103.217	yes
C2H2	transcription factor	c7909_g1	17.017	14.389	no
C2H2	transcription factor	c7735_g1	18.673	20.892	no
C2H2	transcription factor	c7935_g1	30.716	33.147	no
C2H2	transcription factor	c7309_g1	6.691	0.879	no
C2H2	transcription factor	c7687_g1	5.144	18.513	no
C2H2	transcription factor	c29648_g1	0	1.629	no
C2H2	transcription factor	c7508_g1	3.69	8.83	no
C2H2	transcription factor	c29480_g1	1.702	0	no
C2H2	transcription factor	c7400_g1	5.214	47.019	yes
C2H2	transcription factor	c8094_g1	3.543	4.447	no
C2H2	transcription factor	c7238_g1	15.176	10.756	no
C2H2	transcription factor	c7361_g1	20.498	9.722	no
C2H2	transcription factor	c7745_g2	7.395	13.057	no
C2H2	transcription factor	c7486_g1	36.634	77.257	no
C2H2	transcription factor	c7488_g1	0.425	145.284	yes
C2H2	transcription factor	c31386_g1	28.922	39.702	no
C2H2	transcription factor	c31903_g1	25.209	69.346	no
C2H2	transcription factor	c31418_g1	27.576	20.142	no
C2H2	transcription factor	c28601_g1	3.442	0	yes
C2H2	transcription factor	c31884_g1	25.936	2.754	yes

TF Family	Family Type	Sequence Acc.	Fpkm of flower	Fpkm of leaf	significant
C2H2	transcription factor	c31910_g1	0	2.159	no
C2H2	transcription factor	c7228_g1	12.987	22.727	no
C2H2	transcription factor	c12122_g1	9.491	3.271	no
C2H2	transcription factor	c11371_g1	11.177	7.485	no
C2H2	transcription factor	c12263_g1	15.896	8.08	no
C2H2	transcription factor	c11296_g1	17.249	30.238	no
C2H2	transcription factor	c11329_g1	7.457	8.222	no
C2H2	transcription factor	c12022_g2	8.934	8.145	no
C2H2	transcription factor	c11900_g1	9.236	2.573	no
C2H2	transcription factor	c12084_g1	43.766	27.368	no
C2H2	transcription factor	c11953_g1	18.851	9.334	no
C2H2	transcription factor	c12176_g1	19.361	16.095	no
C2H2	transcription factor	c12042_g1	9.994	4.059	no
C2H2	transcription factor	c11395_g2	13.482	25.985	no
C2H2	transcription factor	c11311_g1	7.503	5.132	no
C2H2	transcription factor	c11380_g1	16.94	5.249	no
C2H2	transcription factor	c12213_g1	14.712	13.91	no
C2H2	transcription factor	c12049_g1	4.734	10.226	no
C2H2	transcription factor	c11315_g2	11.788	11.17	no
C2H2	transcription factor	c12211_g1	8.447	16.483	no
C2H2	transcription factor	c11559_g1	8.547	28.454	no
C2H2	transcription factor	c6986_g1	25.472	21.965	no
C2H2	transcription factor	c12169_g3	8.362	4.641	no
C2H2	transcription factor	c12014_g2	22.239	18.345	no
C2H2	transcription factor	c11762_g2	20.761	18.022	no

TF Family	Family Type	Sequence Acc.	Fpkm of flower	Fpkm of leaf	significant
C2H2	transcription factor	c11768_g1	25.271	11.648	no
C2H2	transcription factor	c11547_g1	10.149	12.902	no
C2H2	transcription factor	c11547_g3	7.302	2.469	no
C2H2	transcription factor	c11504_g1	19.609	8.326	no
C2H2	transcription factor	c11298_g2	10.999	8.597	no
C2H2	transcription factor	c11677_g2	14.681	18.332	no
C2H2	transcription factor	c27487_g1	17.087	1.435	yes
C2H2	transcription factor	c27805_g1	12.183	14.337	no
C2H2	transcription factor	c28047_g1	0	2.663	yes
C2H2	transcription factor	c27769_g1	15.757	27.795	no
C2H2	transcription factor	c27763_g1	4.78	1.655	no
C2H2	transcription factor	c13972_g1	20.8	19.586	no
C2H2	transcription factor	c13610_g1	71.388	69.126	no
C2H2	transcription factor	c13993_g1	24.281	7.291	no
C2H2	transcription factor	c14015_g1	55.283	84.743	no
C2H2	transcription factor	c14142_g1	16.638	32.32	no
C2H2	transcription factor	c28790_g1	2.498	2.043	no
C2H2	transcription factor	c13684_g1	243.318	152.834	no
C2H2	transcription factor	c13728_g1	24.513	23.503	no
C2H2	transcription factor	c13437_g1	29.146	173.441	no
C2H2	transcription factor	c13354_g1	20.924	25.313	no
C2H2	transcription factor	c14064_g1	152.607	6.554	yes
C2H2	transcription factor	c13877_g1	13.668	22.236	no
C2H2	transcription factor	c13418_g1	17.984	14.764	no
C2H2	transcription factor	c13415_g1	38.869	17.66	no

TF Family	Family Type	Sequence Acc.	Fpkm of flower	Fpkm of leaf	significant
C2H2	transcription factor	c405_g1	11.286	28.816	no
C2H2	transcription factor	c41_g1	11.688	1.474	no
C2H2	transcription factor	c798_g1	2.282	2.586	no
C2H2	transcription factor	c865_g1	4.881	7.485	no
C2H2	transcription factor	c746_g1	4.78	19.831	no
C2H2	transcription factor	c213_g1	29.819	10.73	no
C2H2	transcription factor	c139_g1	25.843	9.243	no
C2H2	transcription factor	c670_g1	37.438	24.033	no
C2H2	transcription factor	c441_g1	36.216	12.101	no
C2H2	transcription factor	c327_g1	8.029	14.441	no
C2H2	transcription factor	c370_g1	15.362	9.709	no
C2H2	transcription factor	c392_g1	13.49	13.872	no
C2H2	transcription factor	c271_g1	10.682	4.525	no
C2H2	transcription factor	c9649_g1	44.617	19.857	no
C2H2	transcription factor	c9514_g1	8.393	14.285	no
C2H2	transcription factor	c9601_g1	22.13	48.816	no
C2H2	transcription factor	c9716_g1	13.923	4.344	no
C2H2	transcription factor	c9843_g2	2.661	22.365	no
C2H2	transcription factor	c1093_g1	9.824	4.279	no
C2H2	transcription factor	c9602_g1	17.304	3.607	no
C2H2	transcription factor	c1233_g1	7.147	2.379	no
C2H2	transcription factor	c7012_g1	24.296	17.336	no
C2H2	transcription factor	c2003_g1	8.71	2.107	no
C2H2	transcription factor	c9466_g1	17.064	4.434	no
C2H2	transcription factor	c9840_g1	5.09	2.43	no

TF Family	Family Type	Sequence Acc.	Fpkm of flower	Fpkm of leaf	significant
C2H2	transcription factor	c9858_g1	18.023	6.037	no
C2H2	transcription factor	c9305_g1	25.905	46.773	no
C2H2	transcription factor	c9366_g1	7.348	2.521	no
C2H2	transcription factor	c9922_g1	17.559	12.346	no
C2H2	transcription factor	c9954_g1	7.843	5.507	no
C2H2	transcription factor	c9886_g4	21.674	24.576	no
C2H2	transcription factor	c9741_g1	13.498	12.876	no
C2H2	transcription factor	c5783_g1	23.136	19.974	no
C2H2	transcription factor	c5749_g1	32.047	16.574	no
C2H2	transcription factor	c5359_g1	5.291	24.408	no
C2H2	transcription factor	c6115_g2	3.21	0.646	no
C2H2	transcription factor	c6017_g1	13.56	16.018	no
C2H2	transcription factor	c5735_g1	11.108	6.244	no
C2H2	transcription factor	c5473_g1	3.953	25.093	no
C2H2	transcription factor	c6176_g1	6.683	3.284	no
C2H2	transcription factor	c5875_g1	6.08	6.296	no
C2H2	transcription factor	c5698_g1	17.288	4.111	no
C2H2	transcription factor	c6191_g1	0.657	3.943	no
C2H2	transcription factor	c5750_g1	16.368	29.657	no
C2H2	transcription factor	c1921_g1	21.976	43.554	no
C2H2	transcription factor	c22768_g1	4.03	1.513	no
C2H2	transcription factor	c22905_g1	1.98	1.215	no
C2H2	transcription factor	c22455_g1	88.784	134.89	no
C2H2	transcription factor	c22571_g1	17.265	8.507	no
C2H2	transcription factor	c22443_g1	5.647	3.141	no

TF Family	Family Type	Sequence Acc.	Fpkm of flower	Fpkm of leaf	significant
C2H2	transcription factor	c22532_g1	2.707	2.107	no
C2H2	transcription factor	c1794_g1	10.04	14.247	no
C2H2	transcription factor	c12869_g1	5.972	7.964	no
C2H2	transcription factor	c12725_g1	7.952	11.816	no
C2H2	transcription factor	c12573_g1	2.158	7.343	no
C2H2	transcription factor	c12736_g1	5.678	7.576	no
C2H2	transcription factor	c12729_g2	11.386	41.938	no
C2H2	transcription factor	c13078_g5	4.981	12.075	no
C2H2	transcription factor	c12392_g1	9.831	6.128	no
C2H2	transcription factor	c12539_g2	17.249	37.233	no
C2H2	transcription factor	c13007_g6	57.38	126.539	no
C2H2	transcription factor	c12675_g3	22.958	25.895	no
C2H2	transcription factor	c12994_g3	14.991	26.748	no
C2H2	transcription factor	c13204_g1	16.677	17.866	no
C2H2	transcription factor	c6285_g1	6.606	4.977	no
C2H2	transcription factor	c12331_g1	15.757	4.008	no
C2H2	transcription factor	c12864_g1	22.811	38.836	no
C2H2	transcription factor	c12672_g1	23.329	17.336	no
C2H2	transcription factor	c1870_g1	4.185	6.916	no
C2H2	transcription factor	c12544_g3	2.259	3.09	no
C2H2	transcription factor	c12633_g1	22.896	21.034	no
C2H2	transcription factor	c12836_g1	13.057	3.943	no
C2H2	transcription factor	c1393_g1	3.086	2.78	no
C2H2	transcription factor	c13036_g1	26.423	12.825	no
C2H2	transcription factor	c13015_g1	0.425	16.806	yes

TF Family	Family Type	Sequence Acc.	Fpkm of flower	Fpkm of leaf	significant
C2H2	transcription factor	c11172_g1	32.349	8.351	no
C2H2	transcription factor	c10658_g1	13.931	29.928	no
C2H2	transcription factor	c10408_g1	2.166	7.744	no
C2H2	transcription factor	c11157_g1	7.813	26.774	no
C2H2	transcription factor	c10928_g1	12.291	8.222	no
C2H2	transcription factor	c10843_g1	4.355	7.938	no
C2H2	transcription factor	c10619_g1	1.114	3.84	no
C2H2	transcription factor	c11089_g1	6.66	2.482	no
C2H2	transcription factor	c10528_g2	7.65	14.983	no
C2H2	transcription factor	c10790_g1	9.824	14.428	no
C2H2	transcription factor	c11221_g1	10.883	14.492	no
C2H2	transcription factor	c11201_g1	6.993	15.177	no
C2H2	transcription factor	c1996_g1	11.154	13.523	no
C2H2	transcription factor	c11168_g2	11.866	5.262	no
C2H2	transcription factor	c10564_g1	27.591	18.19	no
C2H2	transcription factor	c11114_g1	6.513	3.788	no
C2H2	transcription factor	c10846_g1	7.464	6.167	no
C2H2	transcription factor	c10544_g1	9.878	6.735	no
C2H2	transcription factor	c11098_g1	18.642	52.759	no
C2H2	transcription factor	c10326_g1	14.279	10.161	no
C2H2	transcription factor	c10777_g1	13.869	8.985	no
C2H2	transcription factor	c10840_g2	6.049	7.576	no
C2H2	transcription factor	c10598_g2	3.287	6.309	no
C2H2	transcription factor	c10309_g1	13.753	2.159	no
C2H2	transcription factor	c11002_g1	14.449	9.864	no

TF Family	Family Type	Sequence Acc.	Fpkm of flower	Fpkm of leaf	significant
C2H2	transcription factor	c10725_g1	10.69	5.921	no
C2H2	transcription factor	c11129_g1	38.181	34.621	no
C2H2	transcription factor	c11173_g1	8.083	16.729	no
C2H2	transcription factor	c1153_g2	11.727	10.859	no
C2H2	transcription factor	c3465_g2	1.145	5.481	no
C2H2	transcription factor	c3383_g1	17.164	2.922	no
C2H2	transcription factor	c4036_g1	1.586	0.672	no
C2H2	transcription factor	c1962_g1	1.586	1.81	no
C2H2	transcription factor	c3606_g1	50.936	33.535	no
C2H2	transcription factor	c3196_g1	7.194	4.809	no
C2H2	transcription factor	c3461_g1	2.823	9.58	no
C2H2	transcription factor	c4050_g1	20.088	5.391	no
C2H2	transcription factor	c3133_g1	20.452	18.319	no
C2H2	transcription factor	c3228_g1	0.364	3.426	yes
C2H2	transcription factor	c3913_g1	15.439	12.863	no
C2H2	transcription factor	c3197_g2	7.627	5.313	no
C2H2	transcription factor	c4008_g1	16.832	16.47	no
C2H2	transcription factor	c3397_g1	9.05	4.046	no
C2H2	transcription factor	c3263_g1	19.292	23.387	no
C2H2	transcription factor	c36329_g1	1.547	0.595	no
C2H2	transcription factor	c37072_g1	0	4.279	no
C2H2	transcription factor	c36917_g1	0.565	4.008	no
C2H2	transcription factor	c37227_g1	1.787	1.345	no
C2H2	transcription factor	c3019_g1	22.254	21.654	no
C2H2	transcription factor	c2379_g1	5.012	7.072	no

TF Family	Family Type	Sequence Acc.	Fpkm of flower	Fpkm of leaf	significant
C2H2	transcription factor	c2431_g1	4.556	10.536	no
C2H2	transcription factor	c2253_g1	8.741	18.203	no
C2H2	transcription factor	c2409_g1	1.369	3.581	no
C2H2	transcription factor	c2933_g1	25.464	28.61	no
C2H2	transcription factor	c2071_g1	16.793	15.061	no
C2H2	transcription factor	c1845_g1	6.938	22.159	no
C2H2	transcription factor	c2446_g1	5.183	23.762	no
C2H2	transcription factor	c1199_g1	18.727	19.987	no
C2H2	transcription factor	c6881_g1	55.229	36.909	no
C2H2	transcription factor	c32838_g1	1.4	32.514	yes
C2H2	transcription factor	c33170_g1	1.957	0.246	no
C2H2	transcription factor	c32927_g1	1.996	1.435	no
C2H2	transcription factor	c6775_g1	42.188	9.709	no
C2H2	transcription factor	c33244_g1	4.107	5.068	no
C2H2	transcription factor	c4786_g1	41.538	63.787	no
C2H2	transcription factor	c4731_g1	1.524	5.921	no
C2H2	transcription factor	c4727_g1	9.004	12.695	no
C2H2	transcription factor	c4930_g1	20.645	9.748	no
C2H2	transcription factor	c6856_g2	25.402	151.296	no
C2H2	transcription factor	c5179_g1	1.4	4.383	no
C2H2	transcription factor	c4628_g1	29.146	6.011	no
C2H2	transcription factor	c4473_g1	17.613	8.675	no
C2H2	transcription factor	c4849_g1	13.428	13.471	no
C2H2	transcription factor	c4869_g1	12.995	8.442	no
C2H2	transcription factor	c5017_g1	10.907	3.568	no

TF Family	Family Type	Sequence Acc.	Fpkm of flower	Fpkm of leaf	significant
C2H2	transcription factor	c5001_g1	42.257	28.338	no
C2H2	transcription factor	c4808_g1	22.339	17.776	no
C2H2	transcription factor	c5158_g1	16.747	22.016	no
C2H2	transcription factor	c4604_g1	0	3.154	no
C2H2	transcription factor	c6304_g1	2.63	3.84	no
C2H2	transcription factor	c4670_g1	16.375	28.778	no
C2H2	transcription factor	c4483_g1	9.708	6.399	no
C2H2	transcription factor	c5004_g1	23.414	80.283	no
C2H2	transcription factor	c34709_g1	1.106	3.723	no
C2H2	transcription factor	c34519_g1	7.666	2.443	no
C2H2	transcription factor	c34680_g1	44.771	74.155	no
C2H2	transcription factor	c34400_g1	271.984	55.254	no
C2H2	transcription factor	c34896_g1	168.867	76.986	no
C2H2	transcription factor	c35116_g1	22.494	49.747	no
C2H2	transcription factor	c34635_g1	26.957	48.596	no
C2H2	transcription factor	c35167_g1	12.268	13.729	no
C2H2	transcription factor	c34630_g1	0	3.943	no
C2H2	transcription factor	c34748_g1	6.358	9.062	no
C2H2	transcription factor	c35195_g1	3.628	1.293	no
C2H2	transcription factor	c34790_g1	42.513	39.805	no
C2H2	transcription factor	c34399_g1	4.084	17.168	no
C2H2	transcription factor	c35159_g1	28.899	23.632	no
C2H2	transcription factor	c34917_g1	30.353	25.39	no
C2H2	transcription factor	c34553_g1	49.412	31.441	no
C2H2	transcription factor	c34724_g1	1.222	1.732	no

TF Family	Family Type	Sequence Acc.	Fpkm of flower	Fpkm of leaf	significant
C2H2	transcription factor	c34949_g1	5.778	2.883	no
C2H2	transcription factor	c34441_g1	204.062	16.677	yes
C2H2	transcription factor	c26377_g1	1.408	2.56	no
C2H2	transcription factor	c26637_g1	22.362	32.204	no
C2H2	transcription factor	c26895_g1	0	1.991	no
C2H2	transcription factor	c26724_g1	16.383	21.344	no
C2H2	transcription factor	c26711_g1	0	3.31	no
C2H2	transcription factor	c26489_g1	99.15	124.871	no
C2H2	transcription factor	c26670_g1	21.937	54.284	no
C2H2	transcription factor	c26559_g1	24.335	16.38	no
C2H2	transcription factor	c26762_g1	4.123	2.831	no
C2H2	transcription factor	c26862_g1	60.218	109.758	no
C2H2	transcription factor	c26701_g1	158.579	203.913	no
C2H2	transcription factor	c38484_g1	0	2.56	no
C2H2	transcription factor	c15537_g1	6.265	0.478	yes
C2H2	transcription factor	c15703_g1	0.557	6.283	yes
C2H2	transcription factor	c15931_g1	1.671	2.521	no
C2H2	transcription factor	c15866_g1	0	3.335	no
C2H2	transcription factor	c15203_g1	12.902	14.027	no
C2H2	transcription factor	c15467_g1	3.148	1.008	no
C2H2	transcription factor	c23915_g1	2.081	5.908	no
C2H2	transcription factor	c6491_g1	8.973	10.187	no
C2H2	transcription factor	c23779_g1	1.315	0	no
C2H2	transcription factor	c23676_g1	4.672	3.284	no
C2H2	transcription factor	c9089_g1	16.7	21.473	no

TF Family	Family Type	Sequence Acc.	Fpkm of flower	Fpkm of leaf	significant
C2H2	transcription factor	c8778_g1	10.071	9.554	no
C2H2	transcription factor	c8294_g1	5.77	6.839	no
C2H2	transcription factor	c8761_g1	29.37	14.738	no
C2H2	transcription factor	c8774_g1	10.056	6.748	no
C2H2	transcription factor	c9014_g1	9.669	3.038	no
C2H2	transcription factor	c8814_g1	7.697	4.654	no
C2H2	transcription factor	c7031_g1	9.986	15.578	no
C2H2	transcription factor	c8796_g1	8.957	10.821	no
C2H2	transcription factor	c9030_g1	12.539	14.415	no
C2H2	transcription factor	c8304_g1	4.541	23.658	no
C2H2	transcription factor	c8526_g1	51.71	44.162	no
C2H2	transcription factor	c8631_g1	15.354	6.761	no
C2H2	transcription factor	c8652_g1	3.52	8.532	no
C2H2	transcription factor	c8959_g1	7.07	3.503	no
C2H2	transcription factor	c8848_g1	0.054	13.497	yes
C2H2	transcription factor	c8380_g1	7.464	5.288	no
C2H2	transcription factor	c9186_g1	34.576	16.147	no
C2H2	transcription factor	c9195_g1	5.979	4.111	no
C2H2	transcription factor	c8415_g1	12.647	74.685	no
C2H2	transcription factor	c6241_g1	19.168	3.736	no
C2H2	transcription factor	c8587_g1	8.222	5.365	no
C2H2	transcription factor	c9131_g1	14.527	8.558	no
C2H2	transcription factor	c9077_g1	29.077	47.497	no
C2H2	transcription factor	c8645_g1	5.531	4.693	no
C2H2	transcription factor	c8946_g1	36.201	27.549	no

TF Family	Family Type	Sequence Acc.	Fpkm of flower	Fpkm of leaf	significant
C2H2	transcription factor	c8880_g1	0.681	13.484	yes
C2H2	transcription factor	c18384_g1	20.421	53.108	no
C2H2	transcription factor	c18887_g1	65.486	92.978	no
C2H2	transcription factor	c19197_g1	2.576	2.469	no
C2H2	transcription factor	c18805_g1	31.211	13.613	no
C2H2	transcription factor	c18381_g1	5.886	3.775	no
C2H2	transcription factor	c18376_g1	33.362	23.076	no
C2H2	transcription factor	c18285_g1	12.183	44.976	no
C2H2	transcription factor	c18517_g1	3.303	0.155	yes
C2H2	transcription factor	c19124_g1	1.477	4.15	no
C2H2	transcription factor	c18368_g1	2.305	5.52	no
C2H2	transcription factor	c19404_g1	2.065	2.948	no
C2H2	transcription factor	c19989_g1	1.16	1.228	no
C2H2	transcription factor	c19557_g1	0.503	0.724	no
C2H2	transcription factor	c19492_g1	0	6.335	no
C2H2	transcription factor	c6694_g1	13.614	21.137	no
C2H2	transcription factor	c19807_g1	11.974	46.528	no
C2H2	transcription factor	c19505_g1	3.024	0	no
C2H2	transcription factor	c19275_g1	7.364	4.266	no
C2H2	transcription factor	c18186_g1	38.042	21.435	no
C2H2	transcription factor	c17710_g1	12.423	15.979	no
C2H2	transcription factor	c17740_g1	11.835	22.107	no
C2H2	transcription factor	c17674_g1	84.437	50.044	no
C2H2	transcription factor	c18133_g1	1.199	19.793	yes
C2H2	transcription factor	c17818_g1	30.593	30.264	no

TF Family	Family Type	Sequence Acc.	Fpkm of flower	Fpkm of leaf	significant
C2H2	transcription factor	c18257_g1	21.728	34.621	no
C2H2	transcription factor	c17745_g1	3.922	15.126	no
C2H2	transcription factor	c18058_g1	3.929	0.724	no
C2H2	transcription factor	c25958_g1	4.239	4.654	no
C2H2	transcription factor	c26095_g1	14.774	35.203	no
C2H2	transcription factor	c25373_g1	0	4.008	no
C2H2	transcription factor	c25996_g1	16.159	21.034	no
C2H2	transcription factor	c33869_g1	1.052	1.564	no
C2H2	transcription factor	c6536_g1	17.698	14.208	no
C2H2	transcription factor	c33396_g1	0	1.047	no
C2H2	transcription factor	c33341_g1	0	0	no
C2H2	transcription factor	c33453_g1	0	2.87	no
C2H2	transcription factor	c38170_g1	0.627	3.62	no
C2H2	transcription factor	c37247_g1	4.069	16.108	no
C2H2	transcription factor	c37520_g1	19.16	19.133	no
C2H2	transcription factor	c37461_g1	1.222	2.482	no
C2H2	transcription factor	c38071_g1	0	3.62	no
C2H2	transcription factor	c36064_g1	1.253	12.152	yes
C2H2	transcription factor	c35791_g1	25.673	0.995	yes
C2H2	transcription factor	c35778_g1	0	6.412	yes
C2H2	transcription factor	c35546_g1	3.21	4.602	no
C2H2	transcription factor	c35410_g1	246.342	78.085	no
C2H2	transcription factor	c35862_g1	1.949	3.684	no
C2H2	transcription factor	c35314_g1	1.153	2.469	no
C2H2	transcription factor	c35540_g1	26.833	14.867	no

TF Family	Family Type	Sequence Acc.	Fpkm of flower	Fpkm of leaf	significant
C2H2	transcription factor	c24482_g1	0	3.943	no
C2H2	transcription factor	c24911_g1	0.75	4.77	no
C2H2	transcription factor	c24349_g1	30.384	12.837	no
C2H2	transcription factor	c24622_g1	3.233	0	no
C2H2	transcription factor	c24368_g1	0.193	3.878	no
C2H2	transcription factor	c24240_g1	5.492	30.497	no
C2H2	transcription factor	c24924_g1	0.302	5.559	yes
C2H2	transcription factor	c30361_g1	8.656	27.407	no
C2H2	transcription factor	c30574_g1	0.835	16.431	yes
C2H2	transcription factor	c31266_g1	3.999	0	no
C2H2	transcription factor	c30957_g1	7.898	1.254	no
C2H2	transcription factor	c30945_g1	27.475	44.046	no
C2H2	transcription factor	c30895_g1	19.748	34.104	no
C2H2	transcription factor	c30549_g1	23.43	18.306	no
C2H2	transcription factor	c30515_g1	16.074	45.235	no
C2H2	transcription factor	c30585_g1	0.812	7.343	yes
C2H2	transcription factor	c30456_g1	42.219	23.865	no
C2H2	transcription factor	c31042_g1	35.226	74.517	no
C2H2	transcription factor	c30609_g1	21.519	54.362	no
C2H2	transcription factor	c31153_g1	13.274	42.921	no
C2H2	transcription factor	c31028_g1	0.797	2.237	no
C2H2	transcription factor	c16371_g1	0	0	no
C2H2	transcription factor	c16386_g1	0.967	1.37	no
C2H2	transcription factor	c15087_g1	11.046	0.297	yes
C2H2	transcription factor	c14617_g1	0.217	2.754	no

TF Family	Family Type	Sequence Acc.	Fpkm of flower	Fpkm of leaf	significant
C2H2	transcription factor	c14664_g1	1.617	11.183	no
C2H2	transcription factor	c14824_g1	2.986	3.284	no
C2H2	transcription factor	c14918_g1	30.647	20.491	no
C2H2	transcription factor	c14343_g1	37.608	22.999	no
C3H	transcription factor	c22186_g1	22.045	18.849	no
C3H	transcription factor	c21702_g1	62.036	210.932	no
C3H	transcription factor	c7302_g1	23.12	27.045	no
C3H	transcription factor	c7242_g1	17.033	11.958	no
C3H	transcription factor	c32027_g1	1.725	5.119	no
C3H	transcription factor	c31344_g1	3.164	0	no
C3H	transcription factor	c11637_g1	93.859	57.71	no
C3H	transcription factor	c11973_g1	9.46	8.468	no
C3H	transcription factor	c11830_g1	16.662	25.559	no
C3H	transcription factor	c11449_g1	15.3	9.295	no
C3H	transcription factor	c12267_g1	9.344	13.135	no
C3H	transcription factor	c11822_g1	14.434	11.596	no
C3H	transcription factor	c11290_g1	3.295	1.021	no
C3H	transcription factor	c14263_g1	2.87	3.646	no
C3H	transcription factor	c13318_g1	29.1	30.471	no
C3H	transcription factor	c190_g1	16.36	16.522	no
C3H	transcription factor	c182_g1	63.127	37.413	no
C3H	transcription factor	c10135_g1	12.276	8.442	no
C3H	transcription factor	c9269_g1	58.292	49.165	no
C3H	transcription factor	c10242_g1	15.285	7.421	no
C3H	transcription factor	c1624_g1	17.373	18.655	no

TF Family	Family Type	Sequence Acc.	Fpkm of flower	Fpkm of leaf	significant
C3H	transcription factor	c22351_g1	1.841	0.517	no
C3H	transcription factor	c22395_g1	20.908	57.232	no
C3H	transcription factor	c12979_g2	21.55	12.734	no
C3H	transcription factor	c12357_g1	3.218	9.36	no
C3H	transcription factor	c12526_g2	3.318	8.791	no
C3H	transcription factor	c12639_g2	2.56	7.447	no
C3H	transcription factor	c10659_g1	2.676	4.499	no
C3H	transcription factor	c10305_g1	7.735	11.687	no
C3H	transcription factor	c11265_g2	2.785	14.053	no
C3H	transcription factor	c10507_g1	5.407	5.675	no
C3H	transcription factor	c3225_g1	49.861	49.579	no
C3H	transcription factor	c36618_g1	3.442	0.375	no
C3H	transcription factor	c3014_g1	92.907	33.845	no
C3H	transcription factor	c2708_g1	11.262	4.822	no
C3H	transcription factor	c4558_g1	12.469	24.124	no
C3H	transcription factor	c4524_g1	8.13	3.426	no
C3H	transcription factor	c34876_g1	123.392	18.022	no
C3H	transcription factor	c34507_g1	0.464	0.983	no
C3H	transcription factor	c6644_g1	11.827	14.906	no
C3H	transcription factor	c23439_g1	4.904	3.503	no
C3H	transcription factor	c23493_g1	4.285	8.494	no
C3H	transcription factor	c23349_g1	5.438	25.959	no
C3H	transcription factor	c8892_g1	7.488	2.262	no
C3H	transcription factor	c8961_g1	2.297	8.972	no
C3H	transcription factor	c8787_g1	52.893	14.453	no

TF Family	Family Type	Sequence Acc.	Fpkm of flower	Fpkm of leaf	significant
C3H	transcription factor	c8574_g1	4.192	13.251	no
C3H	transcription factor	c8332_g1	33.168	20.051	no
C3H	transcription factor	c9066_g1	11.239	3.077	no
C3H	transcription factor	c8302_g1	21.767	10.575	no
C3H	transcription factor	c8298_g1	28.806	37.013	no
C3H	transcription factor	c8298_g2	15.2	68.247	no
C3H	transcription factor	c8944_g1	12.067	9.256	no
C3H	transcription factor	c19258_g1	1.795	1.706	no
C3H	transcription factor	c7158_g2	18.518	27.187	no
C3H	transcription factor	c26264_g1	29.85	168.671	no
C3H	transcription factor	c38009_g1	0.394	3.064	no
C3H	transcription factor	c35306_g1	6.467	1.797	no
C3H	transcription factor	c30379_g1	3.326	1.706	no
C3H	transcription factor	c14277_g1	22.424	23.219	no
C3H-WRC/GR	transcription factor interactor and regulator	c22064_g1	14.48	85.531	no
C3H-WRC/GR	transcription factor interactor and regulator	c7722_g1	19.802	8.222	no
C3H-WRC/GR	transcription factor interactor and regulator	c27437_g1	44.23	7.744	no
C3H-WRC/GR	transcription factor interactor and regulator	c5587_g1	4.131	3.516	no
C3H-WRC/GR	transcription factor interactor and regulator	c22710_g1	1.106	5.352	no
C3H-WRC/GR	transcription factor interactor and regulator	c3405_g1	6.343	0	yes
C3H-WRC/GR	transcription factor interactor and regulator	c2359_g1	42.613	8.52	no
C3H-WRC/GR	transcription factor interactor and regulator	c6475_g1	26.632	5.507	no
C3H-WRC/GR	transcription factor interactor and regulator	c6987_g1	17.652	0.918	yes
C3H-WRC/GR	transcription factor interactor and regulator	c6987_g2	50.348	7.886	no
C3H-WRC/GR	transcription factor interactor and regulator	c17649_g1	1.369	3.62	no

TF Family	Family Type	Sequence Acc.	Fpkm of flower	Fpkm of leaf	significant
C3H-WRC/GR	transcription factor interactor and regulator	c24375_g1	0.874	2.495	no
C3H-WRC/GR	transcription factor interactor and regulator	c30899_g1	1.609	4.357	no
CCHC(Zn)	transcription factor interactor and regulator	c21753_g1	0	3.064	no
CCHC(Zn)	transcription factor interactor and regulator	c21982_g1	23.499	18.797	no
CCHC(Zn)	transcription factor interactor and regulator	c7224_g1	5.291	4.15	no
CCHC(Zn)	transcription factor interactor and regulator	c7480_g1	2.15	9.037	no
CCHC(Zn)	transcription factor interactor and regulator	c7330_g1	12.16	10.213	no
CCHC(Zn)	transcription factor interactor and regulator	c8195_g2	44.33	18.746	no
CCHC(Zn)	transcription factor interactor and regulator	c28314_g1	4.177	7.796	no
CCHC(Zn)	transcription factor interactor and regulator	c11416_g2	4.154	3.116	no
CCHC(Zn)	transcription factor interactor and regulator	c12101_g1	8.563	3.982	no
CCHC(Zn)	transcription factor interactor and regulator	c12109_g1	13.544	16.729	no
CCHC(Zn)	transcription factor interactor and regulator	c12109_g2	26.733	26.451	no
CCHC(Zn)	transcription factor interactor and regulator	c11397_g1	14.766	4.059	no
CCHC(Zn)	transcription factor interactor and regulator	c11882_g2	10.822	1.448	no
CCHC(Zn)	transcription factor interactor and regulator	c11685_g1	31.111	30.277	no
CCHC(Zn)	transcription factor interactor and regulator	c27998_g1	4.734	6.141	no
CCHC(Zn)	transcription factor interactor and regulator	c13814_g1	5.005	0	no
CCHC(Zn)	transcription factor interactor and regulator	c13340_g1	222.27	91.271	no
CCHC(Zn)	transcription factor interactor and regulator	c14085_g1	0.348	1.5	no
CCHC(Zn)	transcription factor interactor and regulator	c9397_g1	1.818	28.597	yes
CCHC(Zn)	transcription factor interactor and regulator	c10105_g1	9.46	7.395	no
CCHC(Zn)	transcription factor interactor and regulator	c9303_g1	4.842	3.529	no
CCHC(Zn)	transcription factor interactor and regulator	c9548_g1	11.146	5.171	no
CCHC(Zn)	transcription factor interactor and regulator	c9317_g1	219.478	172.989	no

TF Family	Family Type	Sequence Acc.	Fpkm of flower	Fpkm of leaf	significant
CCHC(Zn)	transcription factor interactor and regulator	c10113_g1	11.324	5.184	no
CCHC(Zn)	transcription factor interactor and regulator	c1704_g1	17.164	20.646	no
CCHC(Zn)	transcription factor interactor and regulator	c1713_g1	8.764	2.922	no
CCHC(Zn)	transcription factor interactor and regulator	c5566_g1	55.562	124.91	no
CCHC(Zn)	transcription factor interactor and regulator	c23184_g1	20.924	5.107	no
CCHC(Zn)	transcription factor interactor and regulator	c22357_g1	11.425	2.495	no
CCHC(Zn)	transcription factor interactor and regulator	c1878_g1	53.829	80.14	no
CCHC(Zn)	transcription factor interactor and regulator	c13006_g1	33.663	38.797	no
CCHC(Zn)	transcription factor interactor and regulator	c12694_g1	32.093	15.294	no
CCHC(Zn)	transcription factor interactor and regulator	c12393_g1	21.658	1.073	yes
CCHC(Zn)	transcription factor interactor and regulator	c12580_g1	15.316	11.39	no
CCHC(Zn)	transcription factor interactor and regulator	c12634_g1	18.51	16.742	no
CCHC(Zn)	transcription factor interactor and regulator	c10362_g1	10.899	36.69	no
CCHC(Zn)	transcription factor interactor and regulator	c11137_g1	2.205	1.939	no
CCHC(Zn)	transcription factor interactor and regulator	c10561_g1	46.396	33.238	no
CCHC(Zn)	transcription factor interactor and regulator	c10350_g1	11.208	10.614	no
CCHC(Zn)	transcription factor interactor and regulator	c10326_g3	2.622	2.599	no
CCHC(Zn)	transcription factor interactor and regulator	c1765_g1	22.726	17.556	no
CCHC(Zn)	transcription factor interactor and regulator	c1934_g1	1.199	1.345	no
CCHC(Zn)	transcription factor interactor and regulator	c3644_g1	11.278	3.141	no
CCHC(Zn)	transcription factor interactor and regulator	c3772_g1	1.957	0	no
CCHC(Zn)	transcription factor interactor and regulator	c4013_g1	21.163	28.752	no
CCHC(Zn)	transcription factor interactor and regulator	c3448_g1	4.022	4.37	no
CCHC(Zn)	transcription factor interactor and regulator	c36780_g1	2.344	45.791	yes
CCHC(Zn)	transcription factor interactor and regulator	c2950_g1	32.325	30.988	no

TF Family	Family Type	Sequence Acc.	Fpkm of flower	Fpkm of leaf	significant
CCHC(Zn)	transcription factor interactor and regulator	c3081_g1	44.052	18.203	no
CCHC(Zn)	transcription factor interactor and regulator	c2711_g1	2.452	1.758	no
CCHC(Zn)	transcription factor interactor and regulator	c2427_g1	27.56	39.702	no
CCHC(Zn)	transcription factor interactor and regulator	c2735_g1	1.555	0.608	no
CCHC(Zn)	transcription factor interactor and regulator	c2006_g1	27.321	12.32	no
CCHC(Zn)	transcription factor interactor and regulator	c32719_g1	0.325	0.931	no
CCHC(Zn)	transcription factor interactor and regulator	c34409_g1	97.757	1308.709	yes
CCHC(Zn)	transcription factor interactor and regulator	c34390_g1	0	7.136	yes
CCHC(Zn)	transcription factor interactor and regulator	c8489_g1	30.801	13.342	no
CCHC(Zn)	transcription factor interactor and regulator	c9071_g1	14.109	16.677	no
CCHC(Zn)	transcription factor interactor and regulator	c8484_g1	12.43	0.685	yes
CCHC(Zn)	transcription factor interactor and regulator	c19005_g1	20.019	14.079	no
CCHC(Zn)	transcription factor interactor and regulator	c18643_g1	12.639	3.62	no
CCHC(Zn)	transcription factor interactor and regulator	c18958_g1	11.703	4.46	no
CCHC(Zn)	transcription factor interactor and regulator	c6629_g1	2.336	5.378	no
CCHC(Zn)	transcription factor interactor and regulator	c17845_g1	187.539	78.873	no
CCHC(Zn)	transcription factor interactor and regulator	c17784_g1	65.602	31.531	no
CCHC(Zn)	transcription factor interactor and regulator	c26170_g1	158.973	124.082	no
CCHC(Zn)	transcription factor interactor and regulator	c6814_g1	8.369	11.674	no
CCHC(Zn)	transcription factor interactor and regulator	c31106_g1	147.224	7.692	yes
CG1-CAMTA	transcription factor	c8144_g1	11.378	13.187	no
CG1-CAMTA	transcription factor	c11880_g1	10.357	8.054	no
CG1-CAMTA	transcription factor	c494_g1	13.962	16.897	no
CG1-CAMTA	transcription factor	c6964_g1	23.151	19.495	no
CHROMO-DO	chromatin remodeling & transcriptional activation	c29398_g1	0	4.008	no

TF Family	Family Type	Sequence Acc.	Fpkm of flower	Fpkm of leaf	significant
CHROMO-DO	chromatin remodeling & transcriptional activation	c32204_g1	1.214	6.891	no
CHROMO-DO	chromatin remodeling & transcriptional activation	c13664_g1	74.668	32.708	no
CHROMO-DO	chromatin remodeling & transcriptional activation	c1671_g1	10.806	34.479	no
CHROMO-DO	chromatin remodeling & transcriptional activation	c12778_g2	10.845	1.603	no
CHROMO-DO	chromatin remodeling & transcriptional activation	c12778_g3	43.549	2.043	yes
CHROMO-DO	chromatin remodeling & transcriptional activation	c12488_g1	16.306	6.296	no
CHROMO-DO	chromatin remodeling & transcriptional activation	c10775_g1	8.54	13.342	no
CHROMO-DO	chromatin remodeling & transcriptional activation	c10690_g1	9.893	2.521	no
CHROMO-DO	chromatin remodeling & transcriptional activation	c3965_g1	31.01	1.926	yes
CHROMO-DO	chromatin remodeling & transcriptional activation	c36434_g1	2.321	1.681	no
CHROMO-DO	chromatin remodeling & transcriptional activation	c4565_g1	23.36	11.415	no
CHROMO-DO	chromatin remodeling & transcriptional activation	c15756_g1	1.222	8.145	no
CHROMO-DO	chromatin remodeling & transcriptional activation	c8692_g2	9.166	1.151	yes
CHROMO-DO	chromatin remodeling & transcriptional activation	c8977_g1	5.198	8.093	no
CW-Zn	putative transcription factor & chromatin remodelin	c31317_g1	0.232	2.65	no
CW-Zn	putative transcription factor & chromatin remodelin	c11844_g1	8.416	1.551	no
CW-Zn	putative transcription factor & chromatin remodelin	c22341_g1	1.733	1.228	no
CW-Zn	putative transcription factor & chromatin remodelin	c13107_g2	7.062	15.901	no
CW-Zn	putative transcription factor & chromatin remodelin	c10694_g1	9.174	8.765	no
CW-Zn	putative transcription factor & chromatin remodelin	c1278_g1	0.719	3.568	no
CW-Zn	putative transcription factor & chromatin remodelin	c34469_g1	128.512	63.205	no
CW-Zn	putative transcription factor & chromatin remodelin	c17984_g1	55.067	17.026	no
CW-Zn-B3/VA	transcription factor & chromatin remodeling	c12141_g1	7.65	8.132	no
CW-Zn-B3/VA	transcription factor & chromatin remodeling	c8852_g1	10.381	6.49	no
DDT	transcription factor & chromatin remodeling	c20439_g1	1.044	1.513	no

TF Family	Family Type	Sequence Acc.	Fpkm of flower	Fpkm of leaf	significant
DDT	transcription factor & chromatin remodeling	c11524_g1	9.916	3.361	no
DDT	transcription factor & chromatin remodeling	c12857_g2	1.307	5.081	no
DDT	transcription factor & chromatin remodeling	c3994_g1	18.982	12.825	no
DDT	transcription factor & chromatin remodeling	c30762_g1	0.65	2.159	no
DICER	post-transcriptional gene silencing	c31351_g1	1.323	1.241	no
DICER	post-transcriptional gene silencing	c22391_g1	2.792	0.905	no
DICER	post-transcriptional gene silencing	c12281_g1	7.905	6.076	no
E2F-DP	transcription factor	c9916_g2	27.638	61.834	no
E2F-DP	transcription factor	c3219_g1	16.197	33.47	no
E2F-DP	transcription factor	c2784_g1	5.051	8.894	no
E2F-DP	transcription factor	c6306_g1	57.186	14.466	no
E2F-DP	transcription factor	c19123_g1	16.623	15.242	no
EIL	transcription factor	c11512_g2	1.818	11.661	no
EIL	transcription factor	c12018_g1	5.422	11.351	no
EIL	transcription factor	c17982_g1	40.393	66.476	no
FAR	transcription factor	c20353_g1	2.823	1.383	no
FAR	transcription factor	c8029_g1	1.709	2.973	no
FAR	transcription factor	c11365_g1	3.922	4.434	no
FAR	transcription factor	c10129_g1	6.59	6.154	no
FAR	transcription factor	c12531_g1	12.809	10.549	no
FAR	transcription factor	c10797_g1	9.762	6.774	no
FAR	transcription factor	c36659_g1	1.462	3.439	no
FAR	transcription factor	c15858_g1	0	2.133	no
FAR	transcription factor	c15533_g1	1.508	2.172	no
FAR	transcription factor	c23246_g1	4.347	6.412	no

TF Family	Family Type	Sequence Acc.	Fpkm of flower	Fpkm of leaf	significant
FAR	transcription factor	c23325_g1	1.632	1.732	no
FAR	transcription factor	c8434_g1	6.49	15.733	no
FAR	transcription factor	c6778_g2	11.572	16.121	no
FAR	transcription factor	c36227_g1	1.052	0.504	no
FAR	transcription factor	c35465_g1	1.88	1.487	no
FAR	transcription factor	c14932_g1	1.416	1.513	no
FHA-SMAD	transcription factor interactor and regulator	c21866_g1	18.967	19.224	no
FHA-SMAD	transcription factor interactor and regulator	c7292_g1	12.593	7.847	no
FHA-SMAD	transcription factor interactor and regulator	c11496_g1	19.415	8.507	no
FHA-SMAD	transcription factor interactor and regulator	c12216_g1	11.742	7.55	no
FHA-SMAD	transcription factor interactor and regulator	c9976_g1	13.947	6.011	no
FHA-SMAD	transcription factor interactor and regulator	c5819_g1	17.768	57.529	no
FHA-SMAD	transcription factor interactor and regulator	c22760_g1	1.315	2.004	no
FHA-SMAD	transcription factor interactor and regulator	c12283_g1	2.437	4.822	no
FHA-SMAD	transcription factor interactor and regulator	c7192_g1	57.728	357.083	yes
FHA-SMAD	transcription factor interactor and regulator	c26630_g1	2.924	0.53	no
FHA-SMAD	transcription factor interactor and regulator	c6619_g1	9.398	8.106	no
FHA-SMAD	transcription factor interactor and regulator	c9140_g1	8.787	4.15	no
FHA-SMAD	transcription factor interactor and regulator	c8269_g3	15.602	11.26	no
FHA-SMAD	transcription factor interactor and regulator	c35371_g1	24.064	34.66	no
FYR	chromatin remodeling & transcription regulator	c22603_g1	102.182	11.894	yes
FYR	chromatin remodeling & transcription regulator	c12340_g1	8.176	11.105	no
FYR	chromatin remodeling & transcription regulator	c32319_g1	0	0	no
FYR	chromatin remodeling & transcription regulator	c7119_g1	19.98	20.866	no
FYR	chromatin remodeling & transcription regulator	c20204_g1	0.82	0.879	no

TF Family	Family Type	Sequence Acc.	Fpkm of flower	Fpkm of leaf	significant
FYR	chromatin remodeling & transcription regulator	c26119_g1	1.214	2.418	no
GAGA-Binding	transcription factor	c11981_g1	11.75	5.546	no
GAGA-Binding	transcription factor	c4474_g1	31.142	13.846	no
GAGA-Binding	transcription factor	c34781_g1	96.806	32.617	no
GAGA-Binding	transcription factor	c26292_g1	28.427	26.606	no
GARP-G2-like	transcription factor	c14000_g1	28.899	11.312	no
GARP-G2-like	transcription factor	c9285_g2	14.349	21.848	no
GARP-G2-like	transcription factor	c8704_g1	0.263	10.51	yes
GARP-G2-like	transcription factor	c30732_g1	44.029	60.529	no
GeBP	transcription factor	c18275_g1	75.271	54.388	no
GeBP	transcription factor	c26084_g1	17.296	34.285	no
GRAS	transcription factor	c21821_g1	43.456	56.379	no
GRAS	transcription factor	c30194_g1	21.38	20.672	no
GRAS	transcription factor	c21086_g1	4.943	0	no
GRAS	transcription factor	c13874_g1	18.479	53.224	no
GRAS	transcription factor	c14214_g1	3.79	77.025	yes
GRAS	transcription factor	c13760_g1	10.589	13.82	no
GRAS	transcription factor	c13598_g1	14.619	40.232	no
GRAS	transcription factor	c150_g1	3.496	8.455	no
GRAS	transcription factor	c462_g1	0.951	9.412	yes
GRAS	transcription factor	c9274_g1	2.792	7.576	no
GRAS	transcription factor	c12442_g1	10.357	11.583	no
GRAS	transcription factor	c1297_g1	1.222	7.175	no
GRAS	transcription factor	c1920_g1	7.874	0.401	yes
GRAS	transcription factor	c11240_g2	1.632	1.5	no

TF Family	Family Type	Sequence Acc.	Fpkm of flower	Fpkm of leaf	significant
GRAS	transcription factor	c1620_g1	13.39	15.979	no
GRAS	transcription factor	c3134_g1	45.019	24.537	no
GRAS	transcription factor	c36875_g1	0.178	4.24	no
GRAS	transcription factor	c5090_g1	47.254	73.832	no
GRAS	transcription factor	c34674_g1	25.503	42.481	no
GRAS	transcription factor	c35080_g1	3.465	0	no
GRAS	transcription factor	c26348_g1	49.907	54.556	no
GRAS	transcription factor	c26963_g1	36.456	5.624	no
GRAS	transcription factor	c8746_g2	4.409	8.946	no
GRAS	transcription factor	c7148_g1	7.387	33.302	no
GRAS	transcription factor	c18968_g1	8.934	17.569	no
GRAS	transcription factor	c19246_g1	0	0	no
GRAS	transcription factor	c17655_g1	34.499	46.437	no
GRAS	transcription factor	c25580_g1	3.373	0	no
GRAS	transcription factor	c30755_g1	2.994	10.006	no
GRAS	transcription factor	c14742_g1	4.339	0	no
GRF	transcription factor	c27607_g1	0	3.749	no
GRF	transcription factor	c9703_g1	11.959	2.534	no
GRF	transcription factor	c32523_g1	10.829	0.814	no
GRF	transcription factor	c24418_g1	0	2.004	no
Hap2/NF-YA	transcription factor	c22157_g1	10.945	34.492	no
Hap2/NF-YA	transcription factor	c12624_g1	3.79	37.594	yes
Hap2/NF-YA	transcription factor	c3937_g1	18.263	22.055	no
Hap2/NF-YA	transcription factor	c36787_g1	0.874	4.512	no
Hap2/NF-YA	transcription factor	c2346_g1	73.948	51.065	no

TF Family	Family Type	Sequence Acc.	Fpkm of flower	Fpkm of leaf	significant
Hap2/NF-YA	transcription factor	c4785_g1	33.633	29.683	no
Hap2/NF-YA	transcription factor	c7235_g1	72.587	92.383	no
Hap3/NF-YB	transcription factor	c30258_g1	21.844	17.285	no
Hap3/NF-YB	transcription factor	c21150_g1	5.33	3.154	no
Hap3/NF-YB	transcription factor	c7301_g1	102.746	213.402	no
Hap3/NF-YB	transcription factor	c31681_g1	36.897	29.773	no
Hap3/NF-YB	transcription factor	c12260_g1	3471.795	138.394	yes
Hap3/NF-YB	transcription factor	c6428_g1	0	0	no
Hap3/NF-YB	transcription factor	c11663_g1	2017.002	127.78	yes
Hap3/NF-YB	transcription factor	c11422_g1	1.207	4.938	no
Hap3/NF-YB	transcription factor	c13410_g1	984.031	76.702	yes
Hap3/NF-YB	transcription factor	c14018_g1	366.431	27.213	yes
Hap3/NF-YB	transcription factor	c13306_g1	3030.457	1561.8	no
Hap3/NF-YB	transcription factor	c13384_g1	3380.087	243.472	yes
Hap3/NF-YB	transcription factor	c13582_g1	25.642	15.294	no
Hap3/NF-YB	transcription factor	c393_g1	14.333	6.451	no
Hap3/NF-YB	transcription factor	c352_g1	41.569	88.026	no
Hap3/NF-YB	transcription factor	c49_g1	47.448	1.5	yes
Hap3/NF-YB	transcription factor	c9597_g1	233.03	19.353	yes
Hap3/NF-YB	transcription factor	c6134_g1	111.928	37.594	no
Hap3/NF-YB	transcription factor	c1814_g1	5.268	4.15	no
Hap3/NF-YB	transcription factor	c5502_g1	12.013	9.98	no
Hap3/NF-YB	transcription factor	c5841_g1	15.099	11.17	no
Hap3/NF-YB	transcription factor	c6036_g1	8.648	12.062	no
Hap3/NF-YB	transcription factor	c13199_g1	28.172	25.054	no

TF Family	Family Type	Sequence Acc.	Fpkm of flower	Fpkm of leaf	significant
Hap3/NF-YB	transcription factor	c13144_g1	4915.922	660.243	no
Hap3/NF-YB	transcription factor	c12519_g1	34.267	13.923	no
Hap3/NF-YB	transcription factor	c13039_g1	13.119	9.929	no
Hap3/NF-YB	transcription factor	c12588_g1	1492.464	117.67	yes
Hap3/NF-YB	transcription factor	c10952_g1	27.669	3.426	no
Hap3/NF-YB	transcription factor	c10995_g1	483.309	50.316	yes
Hap3/NF-YB	transcription factor	c3970_g1	1804.578	139.169	yes
Hap3/NF-YB	transcription factor	c36284_g1	8.014	0.388	yes
Hap3/NF-YB	transcription factor	c36738_g1	1.122	0	no
Hap3/NF-YB	transcription factor	c36538_g1	24.737	1.202	yes
Hap3/NF-YB	transcription factor	c3022_g1	91.677	4.085	yes
Hap3/NF-YB	transcription factor	c3001_g1	59.182	2.288	yes
Hap3/NF-YB	transcription factor	c4607_g1	83.308	38.642	no
Hap3/NF-YB	transcription factor	c35061_g1	572.937	22.508	yes
Hap3/NF-YB	transcription factor	c26276_g1	101.834	108.272	no
Hap3/NF-YB	transcription factor	c26294_g1	822.551	44.201	yes
Hap3/NF-YB	transcription factor	c23557_g1	185.203	3.917	yes
Hap3/NF-YB	transcription factor	c18359_g1	475.775	177.565	no
Hap3/NF-YB	transcription factor	c19971_g1	2.723	4.072	no
Hap3/NF-YB	transcription factor	c19441_g1	0	1.099	no
Hap3/NF-YB	transcription factor	c17957_g1	52.615	21.861	no
Hap3/NF-YB	transcription factor	c17510_g1	0	0	no
Hap3/NF-YB	transcription factor	c17971_g1	519.889	130.314	no
Hap3/NF-YB	transcription factor	c17855_g1	460.344	69.346	no
Hap3/NF-YB	transcription factor	c25954_g1	273.353	95.938	no

TF Family	Family Type	Sequence Acc.	Fpkm of flower	Fpkm of leaf	significant
Hap3/NF-YB	transcription factor	c33970_g1	0.65	1.887	no
Hap3/NF-YB	transcription factor	c34083_g1	0	0	no
Hap3/NF-YB	transcription factor	c34201_g1	1.276	0	no
Hap3/NF-YB	transcription factor	c30172_g1	1579.129	283.769	no
Hap3/NF-YB	transcription factor	c35634_g1	20.9	23.102	no
Hap3/NF-YB	transcription factor	c30429_g1	54.773	21.047	no
Hap3/NF-YB	transcription factor	c30375_g1	1313.897	146.241	yes
Hap3/NF-YB	transcription factor	c31023_g1	237.485	9.218	yes
Hap3/NF-YB	transcription factor	c30964_g1	13.127	11.053	no
Hap3/NF-YB	transcription factor	c16839_g1	0.379	3.258	no
Hap3/NF-YB	transcription factor	c14400_g1	317.73	12.217	yes
HD-SAD	transcription factor & lipid binding	c7939_g1	40.316	24.214	no
HD-SAD	transcription factor & lipid binding	c11315_g3	16.894	1.668	yes
HD-SAD	transcription factor & lipid binding	c10437_g1	29.579	4.163	no
HD-SAD	transcription factor & lipid binding	c29975_g1	101.563	44.175	no
HD-SAD	transcription factor & lipid binding	c29998_g1	38.622	4.68	yes
HD-ZIP	transcription factor	c28138_g1	1.377	0	no
HD-ZIP	transcription factor	c409_g1	77.476	6.464	yes
HD-ZIP	transcription factor	c6024_g1	56.923	27.718	no
HD-ZIP	transcription factor	c8812_g1	16.863	31.376	no
HD-ZIP	transcription factor	c8726_g1	112.044	269.871	no
HD-ZIP	transcription factor	c18077_g1	4.548	0.336	no
HD-ZIP	transcription factor	c35557_g1	12.964	24.279	no
HD-ZIP	transcription factor	c24619_g1	0	8.442	no
HD-ZIP	transcription factor	c30385_g1	61.951	37.116	no

TF Family	Family Type	Sequence Acc.	Fpkm of flower	Fpkm of leaf	significant
HMG	chromatin remodeling & transcriptional activation	c21857_g1	1190.637	577.854	no
HMG	chromatin remodeling & transcriptional activation	c7682_g1	9.143	0.582	yes
HMG	chromatin remodeling & transcriptional activation	c12114_g1	6.08	0.194	yes
HMG	chromatin remodeling & transcriptional activation	c12194_g1	11.873	4.525	no
HMG	chromatin remodeling & transcriptional activation	c568_g1	0.356	8.132	yes
HMG	chromatin remodeling & transcriptional activation	c1062_g1	0.998	2.844	no
HMG	chromatin remodeling & transcriptional activation	c13042_g1	145.561	99.804	no
HMG	chromatin remodeling & transcriptional activation	c1799_g1	49.165	4.926	yes
HMG	chromatin remodeling & transcriptional activation	c3911_g1	248.291	6.8	yes
HMG	chromatin remodeling & transcriptional activation	c1797_g2	508.959	41.382	yes
HMG	chromatin remodeling & transcriptional activation	c24161_g1	0	1.099	no
HMG	chromatin remodeling & transcriptional activation	c17643_g1	68.657	34.104	no
Homeodomain-	transcription regulator	c21829_g1	1.725	2.198	no
Homeodomain-	transcription regulator	c32201_g1	1.957	1.383	no
Homeodomain-	transcription regulator	c12137_g1	16.739	15.798	no
Homeodomain-	transcription regulator	c6024_g2	85.365	16.431	no
Homeodomain-	transcription regulator	c12791_g1	338.886	102.351	no
Homeodomain-	transcription regulator	c1077_g1	3.125	6.18	no
Homeodomain-	transcription regulator	c13103_g1	15.803	39.585	no
Homeodomain-	transcription regulator	c23418_g1	1.477	3.4	no
Homeodomain-	transcription regulator	c37915_g1	11.611	4.68	no
Homeodomain-	transcription regulator	c30770_g1	3.713	3.749	no
Homeodomain-	transcription regulator	c17173_g1	2.313	0	no
Homeodomain-	transcription regulator	c15232_g1	1.292	1.875	no
Homeodomain-	chromatin remodeling & transcription regulator	c9398_g1	22.625	12.475	no

TF Family	Family Type	Sequence Acc.	Fpkm of flower	Fpkm of leaf	significant
Homeodomain-	chromatin remodeling & transcription regulator	c12328_g1	2.955	1.525	no
Homeodomain-	transcription factor	c22206_g1	20.599	15.514	no
Homeodomain-	transcription factor	c13706_g1	48.167	19.25	no
Homeodomain-	transcription factor	c439_g1	19.926	16.509	no
Homeodomain-	transcription factor	c6230_g1	7.843	44.02	no
Homeodomain-	transcription factor	c1144_g1	49.443	101.187	no
Homeodomain-	transcription factor	c5497_g1	11.355	109.513	yes
Homeodomain-	transcription factor	c35048_g1	20.444	20.077	no
Homeodomain-	transcription factor	c30122_g1	0.89	54.052	yes
Homeodomain-	transcription factor	c13558_g1	1.617	3.736	no
Homeodomain-	transcription factor	c3068_g1	54.162	129.292	no
Homeodomain-	transcription factor	c33140_g1	18.34	10.911	no
Homeodomain-	transcription factor	c6652_g1	6.111	103.579	yes
Homeodomain-	transcription factor	c27221_g1	51.71	3.322	yes
Homeodomain-	transcription factor	c8286_g1	14.163	0.31	yes
Homeodomain-	transcription factor	c18243_g1	72.796	1.81	yes
Homeodomain-	transcription factor	c16390_g1	1.067	2.314	no
Homobox-WO	transcription factor	c21354_g1	3.079	0	no
Homobox-WO	transcription factor	c21790_g1	192.397	188.89	no
Homobox-WO	transcription factor	c7974_g1	2.576	19.043	yes
Homobox-WO	transcription factor	c7483_g1	10.172	8.83	no
Homobox-WO	transcription factor	c31955_g1	3.086	0	no
Homobox-WO	transcription factor	c11558_g1	9.05	7.692	no
Homobox-WO	transcription factor	c12014_g1	61.626	305.061	no
Homobox-WO	transcription factor	c27365_g1	2.034	0	no

TF Family	Family Type	Sequence Acc.	Fpkm of flower	Fpkm of leaf	significant
Homobox-WO	transcription factor	c485_g1	15.857	0	yes
Homobox-WO	transcription factor	c9800_g1	20.87	1.228	yes
Homobox-WO	transcription factor	c5886_g2	3.744	106.992	yes
Homobox-WO	transcription factor	c23189_g1	1.408	0.827	no
Homobox-WO	transcription factor	c11188_g1	6.931	0.078	yes
Homobox-WO	transcription factor	c3319_g1	35.528	17.647	no
Homobox-WO	transcription factor	c3917_g1	21.527	18.345	no
Homobox-WO	transcription factor	c2514_g2	7.952	0.401	no
Homobox-WO	transcription factor	c2238_g1	39.72	11.196	no
Homobox-WO	transcription factor	c2945_g1	15.138	7.576	no
Homobox-WO	transcription factor	c2245_g1	22.478	24.938	no
Homobox-WO	transcription factor	c4144_g1	25.302	124.806	no
Homobox-WO	transcription factor	c8367_g1	12.84	7.227	no
Homobox-WO	transcription factor	c9233_g1	17.891	15.255	no
Homobox-WO	transcription factor	c18427_g1	1.895	0.155	no
Homobox-WO	transcription factor	c18259_g1	67.776	72.241	no
Homobox-WO	transcription factor	c26103_g1	2.143	0.569	no
Homobox-WO	transcription factor	c33769_g1	2.584	0	no
Homobox-WO	transcription factor	c35882_g1	19.059	20.051	no
HSA	chromatin remodeler	c12412_g1	7.194	12.708	no
HSF-type-DNA	transcription factor	c8156_g1	15.896	3.555	no
HSF-type-DNA	transcription factor	c12124_g1	18.557	28.377	no
HSF-type-DNA	transcription factor	c13941_g1	2.274	0.853	no
HSF-type-DNA	transcription factor	c461_g1	2.63	22.068	yes
HSF-type-DNA	transcription factor	c9624_g1	17.157	4.059	no

TF Family	Family Type	Sequence Acc.	Fpkm of flower	Fpkm of leaf	significant
HSF-type-DNA	transcription factor	c5704_g1	7.534	2.43	no
HSF-type-DNA	transcription factor	c6470_g1	46.697	5.714	no
HSF-type-DNA	transcription factor	c12610_g1	13.916	7.951	no
HSF-type-DNA	transcription factor	c12610_g2	12.09	11.829	no
HSF-type-DNA	transcription factor	c2470_g1	4.827	10.847	no
HSF-type-DNA	transcription factor	c4740_g1	4.804	3.62	no
HSF-type-DNA	transcription factor	c15684_g1	5.337	2.094	no
HSF-type-DNA	transcription factor	c6984_g1	3.187	93.805	yes
HSF-type-DNA	transcription factor	c8870_g1	10.311	12.734	no
HSF-type-DNA	transcription factor	c18278_g1	10.133	3.271	no
HSF-type-DNA	transcription factor	c18031_g1	16.909	21.215	no
HSF-type-DNA	transcription factor	c30745_g1	6.382	5.197	no
HSF-type-DNA	transcription factor	c16563_g1	1.663	0.711	no
ISWI	chromatin remodeling	c7179_g1	31.776	15.449	no
JmjC	transcription factor & chromatin remodeling &Met	c7738_g1	15.757	6.632	no
JmjC	transcription factor & chromatin remodeling &Met	c11469_g2	10.968	1.241	no
JmjC	transcription factor & chromatin remodeling &Met	c12156_g1	4.981	4.719	no
JmjC	transcription factor & chromatin remodeling &Met	c28041_g1	4.154	1.164	no
JmjC	transcription factor & chromatin remodeling &Met	c6884_g1	6.791	9.502	no
JmjC	transcription factor & chromatin remodeling &Met	c9931_g1	22.571	9.088	no
JmjC	transcription factor & chromatin remodeling &Met	c9638_g1	24.915	17.155	no
JmjC	transcription factor & chromatin remodeling &Met	c13073_g1	19.91	11.39	no
JmjC	transcription factor & chromatin remodeling &Met	c12544_g1	20.653	25.248	no
JmjC	transcription factor & chromatin remodeling &Met	c12436_g1	2.676	8.39	no
JmjC	transcription factor & chromatin remodeling &Met	c10326_g2	1.957	1.849	no

TF Family	Family Type	Sequence Acc.	Fpkm of flower	Fpkm of leaf	significant
JmjC	transcription factor & chromatin remodeling &Met	c7093_g1	13.359	4.305	no
JmjC	transcription factor & chromatin remodeling &Met	c9202_g1	8.052	7.033	no
JmjC	transcription factor & chromatin remodeling &Met	c6976_g1	9.507	4.279	no
JmjC	transcription factor & chromatin remodeling &Met	c19385_g1	0.797	1.629	no
JmjC	transcription factor & chromatin remodeling &Met	c25874_g1	1.802	1.034	no
JmjC	transcription factor & chromatin remodeling &Met	c36048_g1	2.243	11.415	no
JmjC	transcription factor & chromatin remodeling &Met	c30693_g1	1.47	1.926	no
JmjC	transcription factor & chromatin remodeling &Met	c16380_g1	2.514	0	no
JmjC-ARID	transcription factor & chromatin remodeling	c23193_g1	1.377	1.345	no
JmjN	transcription factor & chromatin remodeling	c23377_g1	0.627	4.396	no
JmjN	transcription factor & chromatin remodeling	c37784_g1	5.19	3.878	no
Lambda-DB	putative novel transcription factor	c13862_g1	435.258	475.593	no
Lambda-DB	putative novel transcription factor	c18270_g1	33.486	93.754	no
LFY	transcription factor	c7014_g1	43.588	28.661	no
LIM	transcription factor interactor and regulator	c21074_g1	0	4.215	no
LIM	transcription factor interactor and regulator	c32064_g1	1.446	1.06	no
LIM	transcription factor interactor and regulator	c12034_g1	5.337	21.163	no
LIM	transcription factor interactor and regulator	c27530_g1	48.144	19.082	no
LIM	transcription factor interactor and regulator	c665_g1	11.007	25.623	no
LIM	transcription factor interactor and regulator	c5683_g1	5.538	11.816	no
LIM	transcription factor interactor and regulator	c22862_g1	0	2.249	no
LIM	transcription factor interactor and regulator	c23140_g1	18.394	13.936	no
LIM	transcription factor interactor and regulator	c12592_g1	4.943	12.695	no
LIM	transcription factor interactor and regulator	c10467_g1	18.201	7.886	no
LIM	transcription factor interactor and regulator	c10839_g1	9.909	1.952	no

TF Family	Family Type	Sequence Acc.	Fpkm of flower	Fpkm of leaf	significant
LIM	transcription factor interactor and regulator	c11270_g1	7.225	2.715	no
LIM	transcription factor interactor and regulator	c10854_g1	0.634	13.342	yes
LIM	transcription factor interactor and regulator	c2604_g1	0	3.749	no
LIM	transcription factor interactor and regulator	c4680_g1	19.09	59.882	no
LIM	transcription factor interactor and regulator	c9123_g1	11.703	1.008	yes
LIM	transcription factor interactor and regulator	c8445_g1	46.473	52.565	no
LIM	transcription factor interactor and regulator	c18984_g1	0.379	2.159	no
LIM	transcription factor interactor and regulator	c6949_g1	0.673	5.546	no
LIM	transcription factor interactor and regulator	c26089_g1	14.001	17.737	no
LIM	transcription factor interactor and regulator	c38076_g1	1.477	0	no
LIM	transcription factor interactor and regulator	c24919_g1	0	4.111	no
LIM	transcription factor interactor and regulator	c30459_g1	100.101	56.857	no
LIM	transcription factor interactor and regulator	c31202_g1	3.218	1.357	no
LisH	transcription factor interactor and regulator	c21865_g1	93.99	53.897	no
LisH	transcription factor interactor and regulator	c7437_g1	8.246	7.925	no
LisH	transcription factor interactor and regulator	c12212_g1	7.016	11.558	no
LisH	transcription factor interactor and regulator	c10002_g1	17.28	11.079	no
LisH	transcription factor interactor and regulator	c9343_g2	46.388	45.868	no
LisH	transcription factor interactor and regulator	c10089_g1	58.648	66.152	no
LisH	transcription factor interactor and regulator	c5249_g1	19.725	15.876	no
LisH	transcription factor interactor and regulator	c5667_g1	24.613	17.724	no
LisH	transcription factor interactor and regulator	c5746_g1	34.948	12.85	no
LisH	transcription factor interactor and regulator	c6205_g1	5.105	4.189	no
LisH	transcription factor interactor and regulator	c23175_g1	41.499	10.381	no
LisH	transcription factor interactor and regulator	c1414_g2	18.116	17.983	no

TF Family	Family Type	Sequence Acc.	Fpkm of flower	Fpkm of leaf	significant
LisH	transcription factor interactor and regulator	c10282_g1	5.925	0.181	yes
LisH	transcription factor interactor and regulator	c6467_g1	14.449	15.488	no
LisH	transcription factor interactor and regulator	c5143_g1	47.386	25.352	no
LisH	transcription factor interactor and regulator	c7059_g2	31.32	18.862	no
LisH	transcription factor interactor and regulator	c26331_g1	0	6.037	no
LisH	transcription factor interactor and regulator	c7052_g1	24.582	12.863	no
LisH	transcription factor interactor and regulator	c35604_g1	1.547	2.65	no
LisH	transcription factor interactor and regulator	c30015_g1	30.631	29.489	no
MADS-MIKC	transcription factor	c7759_g1	17.961	33.574	no
MADS-MIKC	transcription factor	c8104_g1	46.844	3.064	yes
MADS-MIKC	transcription factor	c8125_g1	36.518	25.365	no
MADS-MIKC	transcription factor	c27857_g1	4.804	0.879	no
MADS-MIKC	transcription factor	c13885_g1	93.658	3.297	yes
MADS-MIKC	transcription factor	c652_g1	8.934	0.401	yes
MADS-MIKC	transcription factor	c435_g1	10.442	3.982	no
MADS-MIKC	transcription factor	c10196_g1	84.584	124.613	no
MADS-MIKC	transcription factor	c5707_g1	39.179	6.542	no
MADS-MIKC	transcription factor	c23128_g1	29.394	27.368	no
MADS-MIKC	transcription factor	c12841_g1	13.66	10.446	no
MADS-MIKC	transcription factor	c12795_g1	191.229	13.678	yes
MADS-MIKC	transcription factor	c10673_g1	118.704	112.034	no
MADS-MIKC	transcription factor	c10447_g3	91.322	5.624	yes
MADS-MIKC	transcription factor	c2017_g1	65.115	3.736	yes
MADS-MIKC	transcription factor	c2017_g2	38.862	2.094	yes
MADS-MIKC	transcription factor	c1538_g1	80.175	4.176	yes

TF Family	Family Type	Sequence Acc.	Fpkm of flower	Fpkm of leaf	significant
MADS-MIKC	transcription factor	c6379_g1	23.67	1.047	yes
MADS-MIKC	transcription factor	c4951_g1	39.31	4.809	no
MADS-MIKC	transcription factor	c8803_g1	60.938	3.038	yes
MADS-MIKC	transcription factor	c26151_g1	94.199	472.904	no
MADS-MIKC	transcription factor	c31046_g1	36.309	10.084	no
MADS-type1	transcription factor	c31515_g1	15.865	0	yes
MADS-type1	transcription factor	c902_g1	5.995	8.869	no
MADS-type1	transcription factor	c5153_g1	5.097	0	no
MADS-type1	transcription factor	c15508_g1	7.403	0	yes
MADS-type1	transcription factor	c24109_g1	1.578	1.706	no
MADS-type1	transcription factor	c34070_g1	0.596	0	no
MBF1	transcription factor	c18047_g1	5.399	0	no
MBF1	transcription factor	c25307_g1	0	1.409	no
MYB	transcription factor	c11595_g1	20.9	10.859	no
MYB	transcription factor	c645_g1	3.373	0.401	no
MYB	transcription factor	c6184_g1	24.064	13.018	no
MYB	transcription factor	c22967_g1	3.04	0.323	no
MYB	transcription factor	c3223_g1	12.616	16.768	no
MYB	transcription factor	c1773_g1	2.127	32.475	yes
MYB	transcription factor	c37024_g1	1.655	0.517	no
MYB	transcription factor	c2786_g1	11.572	15.514	no
MYB	transcription factor	c3069_g1	5.562	2.107	no
MYB	transcription factor	c2963_g1	8.748	7.266	no
MYB	transcription factor	c32305_g1	3.28	2.418	no
MYB	transcription factor	c34813_g1	2.143	0.646	no

TF Family	Family Type	Sequence Acc.	Fpkm of flower	Fpkm of leaf	significant
MYB	transcription factor	c23417_g1	14.96	22.779	no
MYB	transcription factor	c9219_g1	6.962	8.39	no
MYB/SANT	transcription factor	c9676_g1	14.163	24.602	no
MYB/SANT	transcription factor	c9586_g1	7.093	7.097	no
MYB/SANT	transcription factor	c5717_g1	12.044	38.564	no
MYB/SANT	transcription factor	c1224_g1	12.121	7.951	no
MYB/SANT	transcription factor	c12300_g1	5.763	9.308	no
MYB/SANT	transcription factor	c13208_g1	14.442	16.832	no
MYB/SANT	transcription factor	c10494_g1	13.645	15.798	no
MYB/SANT	transcription factor	c10435_g1	20.328	11.467	no
MYB/SANT	transcription factor	c11122_g1	12.461	7.033	no
MYB/SANT	transcription factor	c4450_g1	13.83	10.006	no
MYB/SANT	transcription factor	c34488_g1	18.913	20.672	no
MYB/SANT	transcription factor	c6861_g1	38.397	26.735	no
MYB/SANT	transcription factor	c8530_g2	7.472	244.881	yes
MYB/SANT	transcription factor	c17884_g1	35.11	231.553	no
MYB/SANT	transcription factor	c14889_g1	8.578	18.358	no
MYB-HB-like	transcription factor	c21934_g1	5.515	28.997	no
MYB-HB-like	transcription factor	c22082_g1	20.545	4.964	no
MYB-HB-like	transcription factor	c30204_g1	36.092	67.212	no
MYB-HB-like	transcription factor	c30118_g1	139.937	657.955	no
MYB-HB-like	transcription factor	c7331_g1	5.422	10.058	no
MYB-HB-like	transcription factor	c7752_g1	26.795	2.482	yes
MYB-HB-like	transcription factor	c8016_g1	6.66	0.983	no
MYB-HB-like	transcription factor	c7503_g1	20.545	9.437	no

TF Family	Family Type	Sequence Acc.	Fpkm of flower	Fpkm of leaf	significant
MYB-HB-like	transcription factor	c7503_g2	32.318	4.034	no
MYB-HB-like	transcription factor	c31555_g1	2.591	261.106	yes
MYB-HB-like	transcription factor	c31427_g1	9.754	22.197	no
MYB-HB-like	transcription factor	c12265_g1	3.69	84.058	yes
MYB-HB-like	transcription factor	c11811_g1	5.159	5.908	no
MYB-HB-like	transcription factor	c27364_g1	9.777	14.389	no
MYB-HB-like	transcription factor	c27936_g1	2.205	0.53	no
MYB-HB-like	transcription factor	c13643_g1	22.347	33.044	no
MYB-HB-like	transcription factor	c13808_g1	147.603	258.456	no
MYB-HB-like	transcription factor	c13294_g1	41.569	133.817	no
MYB-HB-like	transcription factor	c13871_g1	2.065	0.892	no
MYB-HB-like	transcription factor	c13857_g1	0	8.313	yes
MYB-HB-like	transcription factor	c355_g1	8.733	105.932	yes
MYB-HB-like	transcription factor	c419_g1	18.363	5.947	no
MYB-HB-like	transcription factor	c28385_g1	3.055	4.589	no
MYB-HB-like	transcription factor	c9681_g1	40.076	21.473	no
MYB-HB-like	transcription factor	c9531_g1	63.931	14.609	no
MYB-HB-like	transcription factor	c9820_g1	1.106	66.527	yes
MYB-HB-like	transcription factor	c9270_g1	9.754	7.123	no
MYB-HB-like	transcription factor	c9270_g2	6.01	5.843	no
MYB-HB-like	transcription factor	c10052_g2	1.895	3.646	no
MYB-HB-like	transcription factor	c10052_g3	2.003	0	yes
MYB-HB-like	transcription factor	c9502_g1	29.78	3.077	no
MYB-HB-like	transcription factor	c5567_g1	29.378	1.319	yes
MYB-HB-like	transcription factor	c2038_g1	0.634	2.249	no

TF Family	Family Type	Sequence Acc.	Fpkm of flower	Fpkm of leaf	significant
MYB-HB-like	transcription factor	c5877_g1	18.332	8.468	no
MYB-HB-like	transcription factor	c5616_g1	5.546	5.701	no
MYB-HB-like	transcription factor	c5728_g2	3.11	25.248	yes
MYB-HB-like	transcription factor	c1702_g1	26.833	18.241	no
MYB-HB-like	transcription factor	c5716_g1	6.18	12.437	no
MYB-HB-like	transcription factor	c23151_g1	1.965	2.87	no
MYB-HB-like	transcription factor	c22515_g1	6.32	3.361	no
MYB-HB-like	transcription factor	c22960_g1	5.353	3.995	no
MYB-HB-like	transcription factor	c12875_g1	58.865	42.3	no
MYB-HB-like	transcription factor	c12441_g1	6.389	2.663	no
MYB-HB-like	transcription factor	c12441_g2	10.52	11.558	no
MYB-HB-like	transcription factor	c1662_g1	22.966	30.665	no
MYB-HB-like	transcription factor	c6839_g1	9.089	4.292	no
MYB-HB-like	transcription factor	c12622_g1	42.721	24.33	no
MYB-HB-like	transcription factor	c12385_g4	4.022	9.877	no
MYB-HB-like	transcription factor	c12554_g1	12.067	11.402	no
MYB-HB-like	transcription factor	c1546_g1	18.34	9.269	no
MYB-HB-like	transcription factor	c12274_g1	62.384	37.801	no
MYB-HB-like	transcription factor	c10927_g3	10.45	28.907	no
MYB-HB-like	transcription factor	c11263_g1	34.275	130.055	no
MYB-HB-like	transcription factor	c10755_g1	14.89	122.66	yes
MYB-HB-like	transcription factor	c11225_g1	15.934	5.856	no
MYB-HB-like	transcription factor	c10416_g1	44.052	91.607	no
MYB-HB-like	transcription factor	c3569_g1	2.498	1.681	no
MYB-HB-like	transcription factor	c3883_g1	19.353	15.63	no

TF Family	Family Type	Sequence Acc.	Fpkm of flower	Fpkm of leaf	significant
MYB-HB-like	transcription factor	c1524_g1	6.281	5.158	no
MYB-HB-like	transcription factor	c2316_g1	3.171	0	no
MYB-HB-like	transcription factor	c2808_g1	7.55	9.36	no
MYB-HB-like	transcription factor	c2561_g1	4.339	10.084	no
MYB-HB-like	transcription factor	c3000_g1	3.257	16.095	no
MYB-HB-like	transcription factor	c2160_g1	17.659	27.136	no
MYB-HB-like	transcription factor	c32774_g1	0	1.629	no
MYB-HB-like	transcription factor	c7145_g1	19.036	2.676	no
MYB-HB-like	transcription factor	c4259_g1	3.844	1.164	no
MYB-HB-like	transcription factor	c35017_g1	0.333	50.393	yes
MYB-HB-like	transcription factor	c34838_g1	41.221	117.955	no
MYB-HB-like	transcription factor	c34822_g1	93.464	73.095	no
MYB-HB-like	transcription factor	c34821_g1	15.254	18.099	no
MYB-HB-like	transcription factor	c26509_g1	19.701	18.642	no
MYB-HB-like	transcription factor	c6937_g1	0.596	4.693	no
MYB-HB-like	transcription factor	c16086_g1	2.413	3.4	no
MYB-HB-like	transcription factor	c23736_g1	0	2.922	no
MYB-HB-like	transcription factor	c23301_g1	57.712	4.279	yes
MYB-HB-like	transcription factor	c8406_g1	11.015	8.3	no
MYB-HB-like	transcription factor	c8749_g1	6.219	3.051	no
MYB-HB-like	transcription factor	c8503_g1	9.406	5.805	no
MYB-HB-like	transcription factor	c8819_g1	31.691	31.544	no
MYB-HB-like	transcription factor	c6542_g1	2.297	4.68	no
MYB-HB-like	transcription factor	c18669_g1	10.381	1.138	yes
MYB-HB-like	transcription factor	c19520_g1	2.274	0	no

TF Family	Family Type	Sequence Acc.	Fpkm of flower	Fpkm of leaf	significant
MYB-HB-like	transcription factor	c19774_g1	0	4.072	no
MYB-HB-like	transcription factor	c18095_g1	1.841	2.12	no
MYB-HB-like	transcription factor	c18137_g1	18.007	167.133	yes
MYB-HB-like	transcription factor	c17673_g1	22.362	21.965	no
MYB-HB-like	transcription factor	c33789_g1	0.743	1.086	no
MYB-HB-like	transcription factor	c34174_g1	3.257	0	no
MYB-HB-like	transcription factor	c37720_g1	4.076	0.646	no
MYB-HB-like	transcription factor	c30228_g1	62.686	86.527	no
MYB-HB-like	transcription factor	c37452_g1	3.906	2.288	no
MYB-HB-like	transcription factor	c36069_g1	14.496	11.777	no
MYB-HB-like	transcription factor	c24512_g1	0.843	3.71	no
MYB-HB-like	transcription factor	c25228_g1	4.432	1.267	no
MYB-HB-like	transcription factor	c30616_g1	25.433	61.589	no
MYB-HB-like	transcription factor	c16305_g1	0	3.206	no
MYB-HB-like	transcription factor	c14638_g1	34.073	28.713	no
MYB-HB-like	transcription factor	c14356_g1	1.934	2.237	no
NAM	transcription factor	c8189_g1	4.486	5.456	no
NAM	transcription factor	c7559_g1	3.937	2.249	no
NAM	transcription factor	c31800_g1	0.348	1.965	no
NAM	transcription factor	c31737_g1	13.8	14.26	no
NAM	transcription factor	c12110_g3	2.127	2.146	no
NAM	transcription factor	c12110_g5	2.065	1.823	no
NAM	transcription factor	c27661_g1	2.645	3.103	no
NAM	transcription factor	c27602_g1	0.657	0	no
NAM	transcription factor	c14201_g1	0.147	3.633	no

TF Family	Family Type	Sequence Acc.	Fpkm of flower	Fpkm of leaf	significant
NAM	transcription factor	c13983_g1	20.46	30.226	no
NAM	transcription factor	c65_g1	60.86	143.423	no
NAM	transcription factor	c769_g1	45.336	162.647	no
NAM	transcription factor	c28542_g1	0.549	3.723	no
NAM	transcription factor	c9646_g1	56.065	1.538	yes
NAM	transcription factor	c5650_g1	2.723	1.28	no
NAM	transcription factor	c5766_g1	13.312	18.552	no
NAM	transcription factor	c5443_g1	2.955	0.388	no
NAM	transcription factor	c5873_g1	3.14	6.49	no
NAM	transcription factor	c6050_g1	18.216	0.259	yes
NAM	transcription factor	c23077_g1	0.982	8.067	yes
NAM	transcription factor	c1993_g1	13.281	19.095	no
NAM	transcription factor	c10573_g1	24.227	20.607	no
NAM	transcription factor	c4097_g1	0.348	2.275	no
NAM	transcription factor	c4063_g1	2.251	4.783	no
NAM	transcription factor	c4025_g1	7.116	7.046	no
NAM	transcription factor	c4025_g2	6.188	5.934	no
NAM	transcription factor	c4754_g1	59.878	78.835	no
NAM	transcription factor	c5078_g1	0.905	4.874	no
NAM	transcription factor	c34687_g1	58.903	61.821	no
NAM	transcription factor	c34872_g1	24.923	7.175	no
NAM	transcription factor	c34832_g1	28.04	41.382	no
NAM	transcription factor	c26404_g1	57.147	98.356	no
NAM	transcription factor	c7139_g1	5.368	6.386	no
NAM	transcription factor	c8980_g1	2.22	14.208	no

TF Family	Family Type	Sequence Acc.	Fpkm of flower	Fpkm of leaf	significant
NAM	transcription factor	c6576_g1	11.881	0.543	yes
NAM	transcription factor	c18490_g1	3.218	2.702	no
NAM	transcription factor	c19933_g1	0.75	1.616	no
NAM	transcription factor	c19577_g1	0	0.931	no
NAM	transcription factor	c25439_g1	0.719	4.641	no
NAM	transcription factor	c33584_g1	0.804	5.908	no
NAM	transcription factor	c33557_g1	0	0	no
NAM	transcription factor	c6574_g1	1.006	25.52	yes
NAM	transcription factor	c35330_g1	0	2.663	no
NAM	transcription factor	c35679_g1	3.991	0.22	no
NAM	transcription factor	c14404_g1	37.268	24.576	no
NAM	transcription factor	c15202_g1	0.379	3.271	no
Nin-like	transcription factor	c27464_g1	23.105	9.024	no
Nin-like	transcription factor	c9841_g1	1.524	31.583	yes
Nin-like	transcription factor	c10592_g1	7.147	9.192	no
Nin-like	transcription factor	c36746_g1	6.103	74.995	yes
Nin-like	transcription factor	c34617_g1	2.986	81.162	yes
Nin-like	transcription factor	c35559_g1	10.512	47.562	no
PAZ-Argonaute	post-transcriptional gene silencing	c7599_g1	80.268	8.468	yes
PAZ-Argonaute	post-transcriptional gene silencing	c11683_g1	9.244	5.753	no
PAZ-Argonaute	post-transcriptional gene silencing	c28442_g1	2.096	0	no
PAZ-Argonaute	post-transcriptional gene silencing	c12160_g1	12.864	1.719	no
PAZ-Argonaute	post-transcriptional gene silencing	c14105_g1	2.468	1.021	no
PAZ-Argonaute	post-transcriptional gene silencing	c12383_g1	250.883	53.573	no
PAZ-Argonaute	post-transcriptional gene silencing	c12599_g1	2.638	4.641	no

TF Family	Family Type	Sequence Acc.	Fpkm of flower	Fpkm of leaf	significant
PAZ-Argonaute	post-transcriptional gene silencing	c1790_g1	28.574	2.741	yes
PAZ-Argonaute	post-transcriptional gene silencing	c12771_g2	11.533	5.119	no
PAZ-Argonaute	post-transcriptional gene silencing	c10420_g2	4.192	0.31	yes
PAZ-Argonaute	post-transcriptional gene silencing	c2877_g2	5.012	3.788	no
PAZ-Argonaute	post-transcriptional gene silencing	c34572_g1	124.289	37.142	no
PAZ-Argonaute	post-transcriptional gene silencing	c38771_g1	1.679	0	no
PHD	chromatin regulator	c21660_g1	33.052	49.307	no
PHD	chromatin regulator	c22155_g1	48.492	36.328	no
PHD	chromatin regulator	c22107_g1	26.501	25.921	no
PHD	chromatin regulator	c30244_g1	7.751	52.294	yes
PHD	chromatin regulator	c30065_g1	1.16	4.719	no
PHD	chromatin regulator	c8158_g1	9.785	15.746	no
PHD	chromatin regulator	c7351_g1	17.814	10.536	no
PHD	chromatin regulator	c12158_g1	5.554	5.701	no
PHD	chromatin regulator	c12251_g1	7.55	11.157	no
PHD	chromatin regulator	c11868_g1	0.835	12.825	yes
PHD	chromatin regulator	c12172_g1	7.697	4.9	no
PHD	chromatin regulator	c11607_g2	13.56	9.929	no
PHD	chromatin regulator	c12184_g1	17.01	8.817	no
PHD	chromatin regulator	c11751_g2	3.264	5.662	no
PHD	chromatin regulator	c11346_g2	1.269	3.516	no
PHD	chromatin regulator	c27802_g1	1.431	3.374	no
PHD	chromatin regulator	c27416_g1	19.671	11.635	no
PHD	chromatin regulator	c13725_g1	34.313	26.709	no
PHD	chromatin regulator	c285_g1	17.126	7.266	no

TF Family	Family Type	Sequence Acc.	Fpkm of flower	Fpkm of leaf	significant
PHD	chromatin regulator	c504_g1	3.883	0.711	no
PHD	chromatin regulator	c9424_g1	4.935	2.469	no
PHD	chromatin regulator	c10186_g2	8.586	12.01	no
PHD	chromatin regulator	c10172_g1	4.556	2.379	no
PHD	chromatin regulator	c9476_g2	29.989	28.661	no
PHD	chromatin regulator	c9616_g1	2.723	7.796	no
PHD	chromatin regulator	c9971_g1	2.839	5.585	no
PHD	chromatin regulator	c9418_g1	17.404	11.053	no
PHD	chromatin regulator	c1705_g1	14.991	11.364	no
PHD	chromatin regulator	c2010_g1	12.384	1.112	yes
PHD	chromatin regulator	c10142_g1	12.129	23.451	no
PHD	chromatin regulator	c9565_g1	8.168	9.696	no
PHD	chromatin regulator	c5673_g1	19.825	2.146	yes
PHD	chromatin regulator	c5192_g1	3.605	0.323	no
PHD	chromatin regulator	c5692_g1	20.993	1.202	yes
PHD	chromatin regulator	c5940_g1	4.44	2.857	no
PHD	chromatin regulator	c5693_g1	6.002	12.282	no
PHD	chromatin regulator	c6099_g1	9.878	17.285	no
PHD	chromatin regulator	c2024_g1	2.638	5.934	no
PHD	chromatin regulator	c23098_g1	45.336	2.909	yes
PHD	chromatin regulator	c1287_g1	2.127	1.293	no
PHD	chromatin regulator	c22864_g1	1.06	6.477	no
PHD	chromatin regulator	c22948_g1	9.182	2.301	no
PHD	chromatin regulator	c22266_g1	6.01	16.095	no
PHD	chromatin regulator	c12949_g1	11.324	8.959	no

TF Family	Family Type	Sequence Acc.	Fpkm of flower	Fpkm of leaf	significant
PHD	chromatin regulator	c1503_g2	2.761	5.275	no
PHD	chromatin regulator	c12804_g1	4.579	9.605	no
PHD	chromatin regulator	c1358_g1	1.16	3.633	no
PHD	chromatin regulator	c12942_g1	5.067	3.904	no
PHD	chromatin regulator	c12974_g1	128.984	50.445	no
PHD	chromatin regulator	c10313_g2	14.604	13.833	no
PHD	chromatin regulator	c10825_g1	5.152	9.062	no
PHD	chromatin regulator	c10799_g1	19.508	10.459	no
PHD	chromatin regulator	c10371_g1	8.841	9.657	no
PHD	chromatin regulator	c11087_g1	14.256	5.262	no
PHD	chromatin regulator	c10498_g1	19.462	16.341	no
PHD	chromatin regulator	c11212_g1	17.528	16.018	no
PHD	chromatin regulator	c3479_g1	35.698	10.523	no
PHD	chromatin regulator	c3126_g1	30.144	43.839	no
PHD	chromatin regulator	c1943_g1	3.512	2.844	no
PHD	chromatin regulator	c3853_g1	6.598	1.642	no
PHD	chromatin regulator	c3979_g2	17.59	24.266	no
PHD	chromatin regulator	c3874_g1	9.963	53.199	no
PHD	chromatin regulator	c3444_g1	3.032	2.624	no
PHD	chromatin regulator	c36619_g1	12.771	13.742	no
PHD	chromatin regulator	c2288_g1	12.361	1.706	no
PHD	chromatin regulator	c2994_g1	1.029	48.57	yes
PHD	chromatin regulator	c2716_g2	0.982	0	no
PHD	chromatin regulator	c2191_g1	10.241	24.02	no
PHD	chromatin regulator	c2085_g1	1.725	0	no

TF Family	Family Type	Sequence Acc.	Fpkm of flower	Fpkm of leaf	significant
PHD	chromatin regulator	c7213_g1	22.03	9.489	no
PHD	chromatin regulator	c4634_g1	7.751	0.685	yes
PHD	chromatin regulator	c4456_g1	5.399	6.723	no
PHD	chromatin regulator	c4303_g1	21.914	15.953	no
PHD	chromatin regulator	c5100_g1	13.869	16.936	no
PHD	chromatin regulator	c34679_g1	0.139	5.365	yes
PHD	chromatin regulator	c34660_g1	123.817	78.835	no
PHD	chromatin regulator	c35002_g1	90.687	15.371	no
PHD	chromatin regulator	c34478_g1	110.451	41.641	no
PHD	chromatin regulator	c26902_g1	51.168	26.696	no
PHD	chromatin regulator	c26622_g1	3.976	13.807	no
PHD	chromatin regulator	c15798_g1	2.019	0.724	no
PHD	chromatin regulator	c24034_g1	81.653	16.729	no
PHD	chromatin regulator	c23960_g1	0.402	2.883	no
PHD	chromatin regulator	c6715_g1	6.683	53.922	yes
PHD	chromatin regulator	c8277_g1	5.152	10.265	no
PHD	chromatin regulator	c8372_g1	30.863	12.695	no
PHD	chromatin regulator	c9208_g2	16.963	1.073	yes
PHD	chromatin regulator	c8770_g1	17.837	35.604	no
PHD	chromatin regulator	c9214_g1	9.901	16.393	no
PHD	chromatin regulator	c8879_g1	12.299	6.735	no
PHD	chromatin regulator	c8734_g2	1.74	3.891	no
PHD	chromatin regulator	c6462_g1	10.164	3.568	no
PHD	chromatin regulator	c18488_g1	36.611	43.438	no
PHD	chromatin regulator	c20058_g1	6.149	0	yes

TF Family	Family Type	Sequence Acc.	Fpkm of flower	Fpkm of leaf	significant
PHD	chromatin regulator	c19901_g1	1.593	0	no
PHD	chromatin regulator	c6974_g1	9.971	4.331	no
PHD	chromatin regulator	c17776_g1	26.253	31.712	no
PHD	chromatin regulator	c38090_g1	1.3	5.766	no
PHD	chromatin regulator	c35483_g1	1.856	0	no
PHD	chromatin regulator	c36103_g1	0.967	6.27	no
PHD	chromatin regulator	c25222_g1	1.253	1.008	no
PHD	chromatin regulator	c16377_g1	5.275	5.908	no
PHD	chromatin regulator	c15112_g1	0.982	1.383	no
PLATZ	putative novel transcription repressor	c32080_g1	1.091	0	no
PLATZ	putative novel transcription repressor	c11543_g1	16.669	4.072	no
PLATZ	putative novel transcription repressor	c84_g1	1.872	2.999	no
PLATZ	putative novel transcription repressor	c23135_g1	4.409	96.313	yes
PLATZ	putative novel transcription repressor	c23130_g1	3.674	0	no
PLATZ	putative novel transcription repressor	c18865_g1	6.776	84.523	yes
RAV	transcription factor	c12982_g2	7.201	18.358	no
RAV	transcription factor	c34413_g1	10.249	34.298	no
RB	putative novel transcription repressor	c10022_g1	28.558	14.686	no
RR-A-type	response regulator and putative novel transcription f	c22054_g1	2.344	25.933	yes
RR-A-type	response regulator and putative novel transcription f	c11999_g1	10.087	14.208	no
RR-A-type	response regulator and putative novel transcription f	c10222_g1	43	22.327	no
RR-A-type	response regulator and putative novel transcription f	c6979_g1	21.156	23.749	no
RR-A-type	response regulator and putative novel transcription f	c1877_g1	16.631	48.79	no
RR-A-type	response regulator and putative novel transcription f	c10963_g1	7.666	2.418	no
RR-A-type	response regulator and putative novel transcription f	c2888_g1	41.043	221.831	no

TF Family	Family Type	Sequence Acc.	Fpkm of flower	Fpkm of leaf	significant
RR-A-type	response regulator and putative novel transcription f	c1156_g1	26.501	7.77	no
RR-A-type	response regulator and putative novel transcription f	c4327_g1	12.206	22.701	no
RR-A-type	response regulator and putative novel transcription f	c4810_g1	6.815	1.629	no
RR-A-type	response regulator and putative novel transcription f	c6803_g1	3.906	2.379	no
RR-A-type	response regulator and putative novel transcription f	c8475_g1	9.081	3.219	no
RR-A-type	response regulator and putative novel transcription f	c9105_g1	12.817	2.663	no
RR-A-type	response regulator and putative novel transcription f	c17725_g1	0	5.223	no
RR-A-type	response regulator and putative novel transcription f	c25711_g1	0	3.594	no
RR-A-type	response regulator and putative novel transcription f	c33295_g1	1.338	0	no
RR-A-type	response regulator and putative novel transcription f	c30373_g1	10.682	43.942	no
RR-A-type	response regulator and putative novel transcription f	c16923_g1	1.949	0	no
RR-A-type	response regulator and putative novel transcription f	c14689_g1	2.027	0	no
S1Fa-like	transcription factor	c34923_g1	99.815	28.08	no
SAP	transcription regulator	c20333_g1	2.205	1.176	no
SAP	transcription regulator	c32173_g1	1.709	0.983	no
SAP	transcription regulator	c10144_g1	10.149	9.347	no
SAP	transcription regulator	c10178_g1	15.115	12.385	no
SAP	transcription regulator	c12332_g2	15.169	5.96	no
SAP	transcription regulator	c26893_g1	16.112	35.086	no
SAP	transcription regulator	c8362_g1	14.465	2.107	no
SAP	transcription regulator	c31161_g1	55.245	28.92	no
SBP	transcription factor	c7570_g1	12.693	20.853	no
SBP	transcription factor	c7356_g1	13.993	5.119	no
SBP	transcription factor	c29198_g1	4.92	6.632	no
SBP	transcription factor	c13518_g1	131.104	63.877	no

TF Family	Family Type	Sequence Acc.	Fpkm of flower	Fpkm of leaf	significant
SBP	transcription factor	c592_g1	82.101	9.114	yes
SBP	transcription factor	c9902_g1	12.918	10.678	no
SBP	transcription factor	c9481_g1	60.853	63.993	no
SBP	transcription factor	c10143_g2	118.139	15.294	no
SBP	transcription factor	c22352_g1	125.449	98.563	no
SBP	transcription factor	c12585_g1	11.061	14.725	no
SBP	transcription factor	c3519_g1	42.443	1.383	yes
SBP	transcription factor	c34731_g1	32.155	24.86	no
SBP	transcription factor	c23585_g1	5.152	5.352	no
SBP	transcription factor	c30970_g1	68.704	18.991	no
SBP	transcription factor	c30929_g1	36.719	54.026	no
SET	chromatin remodeling	c27339_g1	29.564	10.562	no
SET	chromatin remodeling	c13303_g1	11.812	4.021	no
SET	chromatin remodeling	c358_g1	5.662	3.322	no
SET	chromatin remodeling	c9903_g1	16.027	1.875	no
SET	chromatin remodeling	c10055_g1	13.552	10.51	no
SET	chromatin remodeling	c9371_g1	5.763	3.387	no
SET	chromatin remodeling	c23026_g1	1.648	3.62	no
SET	chromatin remodeling	c12848_g2	18.17	8.235	no
SET	chromatin remodeling	c12916_g2	6.111	7.55	no
SET	chromatin remodeling	c1307_g1	7.054	1.306	no
SET	chromatin remodeling	c12692_g2	7.526	12.178	no
SET	chromatin remodeling	c1339_g1	3.79	0.388	no
SET	chromatin remodeling	c12607_g2	16.785	10.821	no
SET	chromatin remodeling	c12527_g2	13.544	3.284	no

TF Family	Family Type	Sequence Acc.	Fpkm of flower	Fpkm of leaf	significant
SET	chromatin remodeling	c12699_g1	12.121	22.236	no
SET	chromatin remodeling	c10800_g1	26.624	8.08	no
SET	chromatin remodeling	c11230_g1	10.682	7.059	no
SET	chromatin remodeling	c10367_g1	13.281	35.578	no
SET	chromatin remodeling	c36317_g1	4.68	4.473	no
SET	chromatin remodeling	c5101_g1	4.223	2.094	no
SET	chromatin remodeling	c26370_g1	20.614	5.184	no
SET	chromatin remodeling	c6521_g1	8.934	7.55	no
SET	chromatin remodeling	c8379_g1	6.142	1.823	no
SET	chromatin remodeling	c8912_g1	18.735	1.293	yes
SET	chromatin remodeling	c9094_g1	7.905	0.246	no
SET	chromatin remodeling	c9094_g2	3.086	1.642	no
SET	chromatin remodeling	c9101_g1	26.57	24.136	no
SET	chromatin remodeling	c8640_g1	9.429	24.033	no
SET	chromatin remodeling	c18682_g1	5.028	0.582	no
SET	chromatin remodeling	c18124_g1	2.87	2.043	no
SET	chromatin remodeling	c33941_g1	6.002	0	no
SET	chromatin remodeling	c7210_g1	12.74	5.236	no
SET	chromatin remodeling	c35307_g1	1.887	0.246	no
SNF2	chromatin remodeling	c30136_g1	2.297	0.556	no
SNF2	chromatin remodeling	c7740_g1	39.89	45.015	no
SNF2	chromatin remodeling	c12195_g1	12.655	3.503	no
SNF2	chromatin remodeling	c11599_g1	10.605	14.531	no
SNF2	chromatin remodeling	c7120_g1	6.714	6.813	no
SNF2	chromatin remodeling	c12169_g1	2.22	3.038	no

TF Family	Family Type	Sequence Acc.	Fpkm of flower	Fpkm of leaf	significant
SNF2	chromatin remodeling	c11276_g1	1.918	5.223	no
SNF2	chromatin remodeling	c27565_g1	1.965	2.521	no
SNF2	chromatin remodeling	c27995_g1	1.818	0.646	no
SNF2	chromatin remodeling	c13631_g1	1.392	1.345	no
SNF2	chromatin remodeling	c9981_g1	12.593	14.777	no
SNF2	chromatin remodeling	c1326_g1	2.854	0.181	no
SNF2	chromatin remodeling	c9368_g1	1.044	9.786	yes
SNF2	chromatin remodeling	c9368_g2	10.566	23.128	no
SNF2	chromatin remodeling	c5293_g1	0.951	1.086	no
SNF2	chromatin remodeling	c1681_g1	16.53	15.785	no
SNF2	chromatin remodeling	c23233_g1	0	5.055	no
SNF2	chromatin remodeling	c13191_g1	25.681	20.491	no
SNF2	chromatin remodeling	c10625_g1	1.006	5.21	no
SNF2	chromatin remodeling	c2113_g1	11.556	17.13	no
SNF2	chromatin remodeling	c34625_g1	0.24	3.116	no
SNF2	chromatin remodeling	c15359_g1	1.725	2.224	no
SNF2	chromatin remodeling	c15788_g1	0.657	1.913	no
SNF2	chromatin remodeling	c15697_g1	1.323	2.702	no
SNF2	chromatin remodeling	c8633_g2	7.155	6.748	no
SNF2	chromatin remodeling	c9151_g1	10.187	4.421	no
SNF2	chromatin remodeling	c19099_g1	3.906	1.513	no
SNF2	chromatin remodeling	c19474_g1	1.183	0.672	no
SNF2	chromatin remodeling	c6776_g2	2.514	4.46	no
SNF2	chromatin remodeling	c25764_g1	2.127	1.577	no
SNF2	chromatin remodeling	c34055_g1	3.999	1.474	no

TF Family	Family Type	Sequence Acc.	Fpkm of flower	Fpkm of leaf	significant
SNF2	chromatin remodeling	c35949_g1	1.346	2.456	no
SNF2	chromatin remodeling	c35621_g1	1.679	0.853	no
SNF2	chromatin remodeling	c24615_g1	0.333	1.422	no
SNF2	chromatin remodeling	c14905_g1	2.266	0	no
ssDNA-binding	transcription factor	c21936_g1	116.507	10.911	yes
ssDNA-binding	transcription factor	c34435_g1	67.83	55.875	no
ssDNA-binding	transcription factor	c35222_g1	77.669	12.385	no
ssDNA-binding	transcription factor	c18370_g1	50.905	22.43	no
SSXT	putative novel transcription factor	c3998_g1	427.794	14.777	yes
SSXT	putative novel transcription factor	c34622_g1	22.656	18.409	no
SSXT	putative novel transcription factor	c14809_g1	43.719	19.741	no
STAT	transcription factor	c3795_g1	5.167	3.18	no
STY-LRP1	transcription factor	c553_g1	14.635	1.81	no
STY-LRP1	transcription factor	c1613_g1	13.947	4.24	no
STY-LRP1	transcription factor	c4489_g1	16.329	0.517	yes
STY-LRP1	transcription factor	c4751_g1	12.43	10.433	no
STY-LRP1	transcription factor	c26367_g1	30.051	17.892	no
STY-LRP1	transcription factor	c30640_g1	8.462	0.297	yes
SWIB-Plus-3	chromatin remodeling	c10187_g2	2.885	1.06	no
TAZ	transcription factor and/or regulators	c12448_g2	17.002	24.136	no
TAZ	transcription factor and/or regulators	c16707_g1	0.681	1.978	no
TCP	transcription factor	c8175_g1	62.462	100.967	no
TCP	transcription factor	c31784_g1	1.609	0.284	no
TCP	transcription factor	c27375_g1	40.865	47.769	no
TCP	transcription factor	c14245_g1	15.741	6.386	no

TF Family	Family Type	Sequence Acc.	Fpkm of flower	Fpkm of leaf	significant
TCP	transcription factor	c324_g1	4.718	3.995	no
TCP	transcription factor	c9528_g1	21.457	53.25	no
TCP	transcription factor	c9864_g1	2.785	10.019	no
TCP	transcription factor	c9864_g2	0.588	6.309	yes
TCP	transcription factor	c1990_g1	0.975	3.503	no
TCP	transcription factor	c22838_g1	9.978	5.585	no
TCP	transcription factor	c1834_g2	23.569	71.608	no
TCP	transcription factor	c2131_g1	11.974	5.029	no
TCP	transcription factor	c2351_g1	7.72	15.772	no
TCP	transcription factor	c3052_g1	18.928	11.971	no
TCP	transcription factor	c9183_g1	3.171	45.429	yes
TCP	transcription factor	c30631_g1	7.062	16.599	no
TCP	transcription factor	c15213_g1	1.16	0	no
Tc-PD	transcription coactivator	c30106_g1	91.933	619.585	yes
Tc-PD	transcription coactivator	c6553_g1	41.414	54.103	no
Tesmin	putative novel transcription factor	c11347_g2	1.408	3.141	no
Tesmin	putative novel transcription factor	c11856_g1	12.701	1.164	yes
Tesmin	putative novel transcription factor	c12271_g2	5.306	1.332	no
Tesmin	putative novel transcription factor	c9349_g1	15.641	3.116	no
Tesmin	putative novel transcription factor	c23262_g1	0.719	2.792	no
Tesmin	putative novel transcription factor	c3518_g1	4.285	10.666	no
TIFY	transcription factor	c5911_g1	48.461	26.825	no
TIFY	transcription factor	c23058_g1	2.359	0.478	no
TIFY	transcription factor	c23017_g1	3.581	0	no
TIFY	transcription factor	c11135_g1	23.654	11.325	no

TF Family	Family Type	Sequence Acc.	Fpkm of flower	Fpkm of leaf	significant
TIFY	transcription factor	c35230_g1	25.279	21.719	no
TIFY	transcription factor	c17909_g1	46.001	30.419	no
TIFY	transcription factor	c26157_g1	279.402	149.059	no
TIFY	transcription factor	c30111_g1	347.549	535.54	no
TTF-type(Zn)	transcription factor and/or regulators	c8181_g1	20.297	14.971	no
TTF-type(Zn)	transcription factor and/or regulators	c12805_g1	21.063	11.532	no
TTF-type(Zn)	transcription factor and/or regulators	c6954_g1	2.088	4.072	no
TUBBY	transcription factor	c30104_g1	23.298	1.513	yes
TUBBY	transcription factor	c13764_g1	68.054	40.801	no
TUBBY	transcription factor	c594_g1	26.624	38.008	no
TUBBY	transcription factor	c3973_g1	33.973	25.416	no
TUBBY	transcription factor	c14377_g1	33.261	41.176	no
WD40-like	transcription factor	c21650_g1	88.452	11.907	no
WD40-like	transcription factor	c22173_g1	8.485	4.137	no
WD40-like	transcription factor	c21961_g1	59.104	47.471	no
WD40-like	transcription factor	c22016_g1	65.177	18.125	no
WD40-like	transcription factor	c21505_g1	2.313	0	no
WD40-like	transcription factor	c20927_g1	1.524	0	no
WD40-like	transcription factor	c20309_g1	2.042	1.241	no
WD40-like	transcription factor	c20680_g1	2.576	3.258	no
WD40-like	transcription factor	c21222_g1	1.609	0	no
WD40-like	transcription factor	c7654_g1	22.231	8.856	no
WD40-like	transcription factor	c30211_g1	29.819	14.544	no
WD40-like	transcription factor	c7764_g1	17.458	1.267	yes
WD40-like	transcription factor	c8091_g1	32.797	10.342	no

TF Family	Family Type	Sequence Acc.	Fpkm of flower	Fpkm of leaf	significant
WD40-like	transcription factor	c7545_g1	14.109	35.772	no
WD40-like	transcription factor	c7852_g1	23.236	14.893	no
WD40-like	transcription factor	c7563_g1	21.945	7.977	no
WD40-like	transcription factor	c32174_g1	1.663	4.77	no
WD40-like	transcription factor	c31666_g1	34.855	8.765	no
WD40-like	transcription factor	c31529_g1	0.804	0	no
WD40-like	transcription factor	c31760_g1	2.715	0.271	no
WD40-like	transcription factor	c28998_g1	0	1.293	no
WD40-like	transcription factor	c31469_g1	63.521	25.158	no
WD40-like	transcription factor	c32259_g1	3.427	0	no
WD40-like	transcription factor	c11961_g2	28.032	15.177	no
WD40-like	transcription factor	c12114_g3	10.559	5.132	no
WD40-like	transcription factor	c12114_g2	8.865	1.758	no
WD40-like	transcription factor	c11729_g2	6.211	3.853	no
WD40-like	transcription factor	c11597_g1	5.608	8.145	no
WD40-like	transcription factor	c12104_g1	11.788	4.757	no
WD40-like	transcription factor	c11286_g1	14.21	7.214	no
WD40-like	transcription factor	c11780_g1	6.969	5.029	no
WD40-like	transcription factor	c12128_g1	11.34	0.931	no
WD40-like	transcription factor	c12128_g2	7.109	1.81	no
WD40-like	transcription factor	c11518_g1	13.622	10.653	no
WD40-like	transcription factor	c11392_g1	10.334	9.605	no
WD40-like	transcription factor	c28866_g1	1.315	0	no
WD40-like	transcription factor	c11432_g1	6.536	27.343	no
WD40-like	transcription factor	c11707_g1	6.908	15.85	no

TF Family	Family Type	Sequence Acc.	Fpkm of flower	Fpkm of leaf	significant
WD40-like	transcription factor	c11407_g2	4.587	3.025	no
WD40-like	transcription factor	c12048_g1	5.422	7.033	no
WD40-like	transcription factor	c27631_g1	3.117	2.599	no
WD40-like	transcription factor	c28137_g1	3.218	0	no
WD40-like	transcription factor	c27534_g1	2.003	3.09	no
WD40-like	transcription factor	c13501_g1	42.76	17.091	no
WD40-like	transcription factor	c13364_g1	104.773	38.267	no
WD40-like	transcription factor	c13799_g1	4.417	1.603	no
WD40-like	transcription factor	c13395_g1	13.97	25.623	no
WD40-like	transcription factor	c13612_g1	20.212	20.051	no
WD40-like	transcription factor	c14124_g1	24.675	8.403	no
WD40-like	transcription factor	c13843_g1	93.325	15.876	no
WD40-like	transcription factor	c14183_g1	1.423	2.301	no
WD40-like	transcription factor	c13656_g1	38.181	10.523	no
WD40-like	transcription factor	c13311_g1	77.058	47.73	no
WD40-like	transcription factor	c107_g2	4.177	5.727	no
WD40-like	transcription factor	c200_g1	2.351	44.369	yes
WD40-like	transcription factor	c89_g1	44.787	44.692	no
WD40-like	transcription factor	c109_g1	37.871	27.317	no
WD40-like	transcription factor	c717_g1	27.444	19.754	no
WD40-like	transcription factor	c658_g1	18.99	2.301	no
WD40-like	transcription factor	c446_g1	10.11	2.973	no
WD40-like	transcription factor	c87_g1	12.307	1.952	no
WD40-like	transcription factor	c9512_g1	7.542	4.24	no
WD40-like	transcription factor	c10108_g1	11.966	7.808	no

TF Family	Family Type	Sequence Acc.	Fpkm of flower	Fpkm of leaf	significant
WD40-like	transcription factor	c10126_g1	30.577	107.923	no
WD40-like	transcription factor	c9969_g1	21.411	26.05	no
WD40-like	transcription factor	c6561_g1	5.391	5.21	no
WD40-like	transcription factor	c10170_g1	17.01	6.024	no
WD40-like	transcription factor	c9984_g1	12.539	9.114	no
WD40-like	transcription factor	c10153_g2	5.538	2.611	no
WD40-like	transcription factor	c7000_g1	11.301	9.463	no
WD40-like	transcription factor	c9592_g1	17.822	1.719	yes
WD40-like	transcription factor	c9568_g1	23.043	66.98	no
WD40-like	transcription factor	c9697_g1	15.2	2.03	no
WD40-like	transcription factor	c10198_g1	12.16	11.558	no
WD40-like	transcription factor	c9401_g1	6.946	8.235	no
WD40-like	transcription factor	c5897_g1	23.995	11.273	no
WD40-like	transcription factor	c5913_g1	26.771	38.474	no
WD40-like	transcription factor	c5665_g1	16.035	8.545	no
WD40-like	transcription factor	c5647_g2	7.573	8.726	no
WD40-like	transcription factor	c5733_g1	25.696	29.153	no
WD40-like	transcription factor	c5996_g1	30.43	27.239	no
WD40-like	transcription factor	c5996_g2	3.303	5.404	no
WD40-like	transcription factor	c5640_g1	37.044	24.641	no
WD40-like	transcription factor	c5664_g1	16.143	5.326	no
WD40-like	transcription factor	c23281_g1	4.703	1.383	no
WD40-like	transcription factor	c22561_g1	1.872	3.943	no
WD40-like	transcription factor	c22400_g1	28.991	12.695	no
WD40-like	transcription factor	c22801_g1	1.849	0.323	no

TF Family	Family Type	Sequence Acc.	Fpkm of flower	Fpkm of leaf	significant
WD40-like	transcription factor	c22539_g1	0	1.59	no
WD40-like	transcription factor	c13051_g1	8.903	6.542	no
WD40-like	transcription factor	c13051_g2	25.286	8.817	no
WD40-like	transcription factor	c12965_g1	5.569	6.942	no
WD40-like	transcription factor	c13155_g1	21.983	7.149	no
WD40-like	transcription factor	c13160_g1	138.622	109.603	no
WD40-like	transcription factor	c13001_g1	24.343	6.348	no
WD40-like	transcription factor	c12542_g2	14.859	7.627	no
WD40-like	transcription factor	c12932_g1	20.893	18.254	no
WD40-like	transcription factor	c12689_g1	7.155	22.029	no
WD40-like	transcription factor	c1228_g1	17.605	23.18	no
WD40-like	transcription factor	c12937_g1	1.671	2.34	no
WD40-like	transcription factor	c1585_g1	4.463	10.872	no
WD40-like	transcription factor	c12661_g3	3.427	8.093	no
WD40-like	transcription factor	c12834_g3	15.354	8.158	no
WD40-like	transcription factor	c13164_g1	70.39	142.828	no
WD40-like	transcription factor	c12902_g1	9.916	10.808	no
WD40-like	transcription factor	c13220_g3	37.369	18.125	no
WD40-like	transcription factor	c12761_g1	13.923	5.986	no
WD40-like	transcription factor	c12761_g2	5.082	2.935	no
WD40-like	transcription factor	c12888_g1	6.08	0	yes
WD40-like	transcription factor	c12513_g2	9.514	5.792	no
WD40-like	transcription factor	c13008_g1	7.294	8.675	no
WD40-like	transcription factor	c12432_g1	5.268	4.576	no
WD40-like	transcription factor	c12782_g2	1.903	3.258	no

TF Family	Family Type	Sequence Acc.	Fpkm of flower	Fpkm of leaf	significant
WD40-like	transcription factor	c12437_g2	7.704	3.801	no
WD40-like	transcription factor	c12810_g1	7.488	8.287	no
WD40-like	transcription factor	c12813_g1	7.735	4.202	no
WD40-like	transcription factor	c13168_g1	23.461	13.652	no
WD40-like	transcription factor	c1832_g1	40.548	24.473	no
WD40-like	transcription factor	c13033_g1	56.088	0.724	yes
WD40-like	transcription factor	c13033_g2	9.437	0.246	yes
WD40-like	transcription factor	c13033_g3	12.446	0.75	yes
WD40-like	transcription factor	c13091_g1	55.539	51.414	no
WD40-like	transcription factor	c12310_g2	24.203	5.895	no
WD40-like	transcription factor	c13187_g1	30.136	12.372	no
WD40-like	transcription factor	c12304_g1	21.225	10.834	no
WD40-like	transcription factor	c10285_g1	32.495	14.247	no
WD40-like	transcription factor	c1719_g1	15.718	6.464	no
WD40-like	transcription factor	c10569_g1	7.209	6.852	no
WD40-like	transcription factor	c10400_g1	17.891	12.85	no
WD40-like	transcription factor	c10997_g1	11.904	2.004	no
WD40-like	transcription factor	c10566_g1	5.036	9.799	no
WD40-like	transcription factor	c11117_g1	13.622	7.666	no
WD40-like	transcription factor	c10366_g1	7.72	14.285	no
WD40-like	transcription factor	c11057_g1	18.719	8.455	no
WD40-like	transcription factor	c10409_g1	14.403	0.582	yes
WD40-like	transcription factor	c11198_g1	8.168	4.305	no
WD40-like	transcription factor	c11198_g2	4.912	3.93	no
WD40-like	transcription factor	c1541_g1	18.541	9.799	no

TF Family	Family Type	Sequence Acc.	Fpkm of flower	Fpkm of leaf	significant
WD40-like	transcription factor	c10470_g1	6.993	4.111	no
WD40-like	transcription factor	c10814_g1	7.836	14.828	no
WD40-like	transcription factor	c11073_g1	32.789	25.197	no
WD40-like	transcription factor	c10804_g1	14.434	22.857	no
WD40-like	transcription factor	c10804_g2	4.44	12.398	no
WD40-like	transcription factor	c10861_g1	8.222	6.425	no
WD40-like	transcription factor	c3194_g1	7.874	2.34	no
WD40-like	transcription factor	c3828_g1	20.197	6.981	no
WD40-like	transcription factor	c4044_g1	1.207	2.159	no
WD40-like	transcription factor	c3729_g1	34.862	3.581	yes
WD40-like	transcription factor	c3201_g1	43.959	19.754	no
WD40-like	transcription factor	c4124_g1	36.27	4.757	no
WD40-like	transcription factor	c3594_g1	3.945	3.167	no
WD40-like	transcription factor	c4093_g1	1.4	2.857	no
WD40-like	transcription factor	c3361_g1	25.781	31.402	no
WD40-like	transcription factor	c1831_g1	28.396	5.119	no
WD40-like	transcription factor	c3205_g1	14.728	13.536	no
WD40-like	transcription factor	c1831_g2	8.47	4.913	no
WD40-like	transcription factor	c37225_g1	2.622	0	no
WD40-like	transcription factor	c36634_g1	1.214	0.853	no
WD40-like	transcription factor	c2350_g1	26.996	9.45	no
WD40-like	transcription factor	c3040_g1	24.737	9.373	no
WD40-like	transcription factor	c2159_g1	14.186	10.885	no
WD40-like	transcription factor	c2104_g1	22.084	7.227	no
WD40-like	transcription factor	c2931_g1	6.838	13.626	no

TF Family	Family Type	Sequence Acc.	Fpkm of flower	Fpkm of leaf	significant
WD40-like	transcription factor	c2070_g1	5.917	1.564	no
WD40-like	transcription factor	c2075_g1	17.528	12.475	no
WD40-like	transcription factor	c2440_g1	50.441	24.835	no
WD40-like	transcription factor	c2324_g1	38.846	16.742	no
WD40-like	transcription factor	c2213_g1	50.023	11.131	no
WD40-like	transcription factor	c6408_g1	9.661	5.856	no
WD40-like	transcription factor	c32842_g1	0.766	0	no
WD40-like	transcription factor	c4181_g1	13.451	16.548	no
WD40-like	transcription factor	c4409_g1	34.692	17.013	no
WD40-like	transcription factor	c7198_g1	17.404	12.165	no
WD40-like	transcription factor	c4675_g1	4.038	5.662	no
WD40-like	transcription factor	c4940_g1	8.354	2.275	no
WD40-like	transcription factor	c4466_g1	63.893	18.771	no
WD40-like	transcription factor	c4413_g1	9.87	7.899	no
WD40-like	transcription factor	c4368_g1	1.957	1.422	no
WD40-like	transcription factor	c4736_g1	24.814	18.991	no
WD40-like	transcription factor	c4274_g1	25.944	14.285	no
WD40-like	transcription factor	c34756_g1	70.955	14.751	no
WD40-like	transcription factor	c34936_g1	7.874	0.129	yes
WD40-like	transcription factor	c34535_g1	143.039	70.936	no
WD40-like	transcription factor	c34517_g1	16.43	7.136	no
WD40-like	transcription factor	c6888_g1	8.988	0.233	yes
WD40-like	transcription factor	c35187_g1	23.824	4.887	no
WD40-like	transcription factor	c35123_g1	13.923	10.95	no
WD40-like	transcription factor	c34801_g1	35.342	21.797	no

TF Family	Family Type	Sequence Acc.	Fpkm of flower	Fpkm of leaf	significant
WD40-like	transcription factor	c34598_g1	27.383	17.931	no
WD40-like	transcription factor	c34824_g1	50.797	4.926	yes
WD40-like	transcription factor	c7063_g1	7.464	8.429	no
WD40-like	transcription factor	c27233_g1	4.394	4.887	no
WD40-like	transcription factor	c26702_g1	108.95	67.303	no
WD40-like	transcription factor	c27023_g1	5.028	1.862	no
WD40-like	transcription factor	c6958_g1	15.076	20.956	no
WD40-like	transcription factor	c7164_g1	41.878	21.034	no
WD40-like	transcription factor	c15991_g1	1.191	0	no
WD40-like	transcription factor	c15294_g1	13.188	32.01	no
WD40-like	transcription factor	c15591_g1	17.296	25.468	no
WD40-like	transcription factor	c7058_g1	12.585	11.441	no
WD40-like	transcription factor	c24114_g1	5.221	0	yes
WD40-like	transcription factor	c23875_g1	0.642	1.875	no
WD40-like	transcription factor	c6997_g1	18.572	1.293	yes
WD40-like	transcription factor	c24263_g1	2.854	0	no
WD40-like	transcription factor	c8861_g1	20.684	10.743	no
WD40-like	transcription factor	c9161_g1	10.04	34.931	no
WD40-like	transcription factor	c8292_g1	6.049	0.802	no
WD40-like	transcription factor	c6772_g1	5.384	0.983	no
WD40-like	transcription factor	c6940_g1	30.624	13.807	no
WD40-like	transcription factor	c8823_g1	12.307	13.755	no
WD40-like	transcription factor	c6750_g1	36.069	86.889	no
WD40-like	transcription factor	c8511_g1	54.634	27.911	no
WD40-like	transcription factor	c8511_g2	56.946	8.248	no

TF Family	Family Type	Sequence Acc.	Fpkm of flower	Fpkm of leaf	significant
WD40-like	transcription factor	c8971_g1	11.618	21.745	no
WD40-like	transcription factor	c6999_g2	30.871	13.471	no
WD40-like	transcription factor	c17929_g1	35.845	13.691	no
WD40-like	transcription factor	c17967_g1	46.867	27.588	no
WD40-like	transcription factor	c17391_g1	0.634	1.823	no
WD40-like	transcription factor	c26235_g1	4.2	0	yes
WD40-like	transcription factor	c25377_g1	1.88	0.672	no
WD40-like	transcription factor	c7086_g1	9.352	14.091	no
WD40-like	transcription factor	c34173_g1	14.72	6.231	no
WD40-like	transcription factor	c37704_g1	1.222	0	no
WD40-like	transcription factor	c37495_g1	2.893	5.288	no
WD40-like	transcription factor	c29996_g1	926.883	422.783	no
WD40-like	transcription factor	c37420_g1	0.549	1.241	no
WD40-like	transcription factor	c35458_g1	0	1.164	no
WD40-like	transcription factor	c24507_g1	1.361	1.926	no
WD40-like	transcription factor	c31269_g1	2.251	0	no
WD40-like	transcription factor	c31186_g1	18.704	12.592	no
WD40-like	transcription factor	c30405_g1	117.513	7.007	yes
WD40-like	transcription factor	c30827_g1	33.764	6.929	no
WD40-like	transcription factor	c30744_g1	20.057	23.206	no
WD40-like	transcription factor	c16396_g1	2.058	0.491	no
WD40-like	transcription factor	c29898_g1	2.854	0	no
WD40-like	transcription factor	c14938_g1	0	3.491	no
WD40-like	transcription factor	c15153_g1	11.881	0.414	yes
WD40-like	transcription factor	c14710_g1	1.253	3.245	no

TF Family	Family Type	Sequence Acc.	Fpkm of flower	Fpkm of leaf	significant
WD40-like	transcription factor	c14385_g1	24.884	17.97	no
WRKY	transcription factor	c22210_g1	4.254	29.683	yes
WRKY	transcription factor	c7678_g1	12.106	11.092	no
WRKY	transcription factor	c7996_g1	19.98	50.432	no
WRKY	transcription factor	c32183_g1	0.24	2.883	no
WRKY	transcription factor	c11852_g1	21.218	41.098	no
WRKY	transcription factor	c27687_g1	0	4.344	no
WRKY	transcription factor	c6384_g1	8.354	22.637	no
WRKY	transcription factor	c14175_g1	18.394	23.4	no
WRKY	transcription factor	c9496_g1	0.596	16.276	yes
WRKY	transcription factor	c1748_g1	3.829	40.607	yes
WRKY	transcription factor	c1340_g1	6.938	8.83	no
WRKY	transcription factor	c13049_g2	2.854	20.129	yes
WRKY	transcription factor	c1812_g1	6.057	0.336	yes
WRKY	transcription factor	c10458_g1	1.617	3.077	no
WRKY	transcription factor	c10458_g2	2.553	4.667	no
WRKY	transcription factor	c10709_g1	8.54	2.883	no
WRKY	transcription factor	c10941_g1	2.947	15.436	no
WRKY	transcription factor	c11162_g1	67.876	280.498	no
WRKY	transcription factor	c10631_g2	7.163	19.224	no
WRKY	transcription factor	c3350_g2	2.127	4.589	no
WRKY	transcription factor	c1207_g1	23.964	9.799	no
WRKY	transcription factor	c3918_g1	16.074	10.148	no
WRKY	transcription factor	c36698_g1	1.044	2.65	no
WRKY	transcription factor	c3061_g1	0.789	16.47	yes

TF Family	Family Type	Sequence Acc.	Fpkm of flower	Fpkm of leaf	significant
WRKY	transcription factor	c4690_g1	22.71	3.335	no
WRKY	transcription factor	c5036_g1	2.468	9.463	no
WRKY	transcription factor	c16195_g1	1.261	0.711	no
WRKY	transcription factor	c23681_g1	0.642	2.288	no
WRKY	transcription factor	c8584_g2	5.484	1.034	no
WRKY	transcription factor	c8330_g2	46.419	24.434	no
WRKY	transcription factor	c7142_g1	2.63	0.569	no
WRKY	transcription factor	c20084_g1	0.719	4.189	no
WRKY	transcription factor	c25271_g1	0.41	4.745	no
WRKY	transcription factor	c36093_g1	1.238	0	no
WRKY	transcription factor	c24574_g1	2.901	0	no
WRKY	transcription factor	c24403_g1	0	2.521	no
YEATS	putative novel transcription factor	c4594_g1	48.569	16.25	no
Znf-B	transcription factor interactor and regulator	c22188_g1	3.017	56.392	yes
Znf-B	transcription factor interactor and regulator	c20689_g1	27.367	2.353	no
Znf-B	transcription factor interactor and regulator	c32251_g1	2.29	0.362	no
Znf-B	transcription factor interactor and regulator	c27517_g1	2.251	6.981	no
Znf-B	transcription factor interactor and regulator	c14122_g1	0.944	3.167	no
Znf-B	transcription factor interactor and regulator	c9554_g1	10.659	5.624	no
Znf-B	transcription factor interactor and regulator	c10264_g1	128.783	331.356	no
Znf-B	transcription factor interactor and regulator	c5688_g1	1.849	3.09	no
Znf-B	transcription factor interactor and regulator	c6008_g1	30.461	41.525	no
Znf-B	transcription factor interactor and regulator	c12776_g5	73.322	324.802	no
Znf-B	transcription factor interactor and regulator	c11254_g1	35.342	161.134	no
Znf-B	transcription factor interactor and regulator	c3823_g1	0.835	6.593	no

TF Family	Family Type	Sequence Acc.	Fpkm of flower	Fpkm of leaf	significant
Znf-B	transcription factor interactor and regulator	c23435_g1	2.707	1.642	no
Znf-B	transcription factor interactor and regulator	c6993_g1	2.614	3.672	no
Znf-B	transcription factor interactor and regulator	c8374_g1	2.599	50.6	yes
Znf-LSD	transcription factor	c5898_g1	6.273	4.37	no
Znf-LSD	transcription factor	c2947_g1	4.858	10.756	no
Znf-LSD	transcription factor	c3002_g1	17.482	24.408	no
Znf-LSD	transcription factor	c34775_g1	41.476	134.192	no
Znf-LSD	transcription factor	c26612_g1	9.066	14.932	no
