# Supplementary material for: De novo transcriptome analysis of Tibetan medicinal plant Dysphania schraderiana
Source: Genet Mol Biol. 2019 Jun 13;42(2):480–7. doi: 10.1590/1678-4685-GMB-2018-0033 (PMC6726160; doi:10.1590/1678-4685-GMB-2018-0033)
Supplement: Supplementary file 9 [file 1415-4757-GMB-1678-4685-GMB-2018-0033-20190513-suppl6.pdf]

Supplementary Material to "*De novo* transcriptome analysis of Tibetan medicinal plant *Dysphania schraderiana*"

Table S6 - Unigenes involved in terpenoid backbone biosynthesis.

Enzyme	Gene name	Unigene	FPKM of Flower	FPKM of Leaf
acetyl-CoA acetyltransferase (AACT)	ACCT1	c12287_g1	48.824	16.793
	ACCT2	c26468_g1	0	4.757
	ACCT3	c9396_g2	16.221	10.213
hydroxymethylglutaryl-CoA synthase (HMGS)	HMGS	c26201_g1	52.87	12.152
hydroxymethylglutaryl-CoA reductase (HMGR)	HMGR1	c1164_g1	79.804	63.037
	HMGR2	c35918_g1	7.704	5.701
	HMGR3	c5175_g1	3.21	1.9
mevalonate kinase (MVK)	MVK1	c23215_g1	0	0
	MVK2	c37695_g1	1.3	0
	MVK3	c8234_g1	17.304	4.37
phosphomevalonate kinase (PMK)	PMK	c7079_g1	9.824	6.218
diphosphomevalonate decarboxylase (MVD)	MVD	c14561_g1	45.545	11.493
1-deoxy-D-xylulose-5-phosphate synthase (DXS)	DXS1	c18241_g1	32.766	52.345
	DXS2	c31669_g1	0.789	0.75
	DXS3	c9596_g1	25.588	60.037
	DXS4	c9876_g1	6.103	3.322
1-deoxy-D-xylulose-5-phosphate reductoisomerase (DXR)	DXR	c7994_g2	35.682	105.957
2-C-methyl-D-erythritol 4-phosphate cytidylyltransferase (MCT)	MCT	c13625_g1	24.505	68.311
4-diphosphocytidyl-2-C-methyl-D-erythritol kinase (CMK)	CMK	c9363_g1	15.548	27.2
2-C-methyl-D-erythritol 2,4-cyclodiphosphate synthase (MDS)	MDS	c17650_g1	44.74	226.433
(E)-4-hydroxy-3-methylbut-2-enyl-diphosphate synthase (HDS)	HDS	c5584_g1	13.699	84.613
isopentenyl-diphosphate Delta-isomerase (IDI)	IDI	c5584_g1	13.699	84.613
geranyl-diphosphate synthase (GPP synthase)	GPPS	c1893_g1	14.697	13.303
farnesyl diphosphate synthase (FPP synthase)	FPPS1	c12747_g1	17.211	4.408
	FPPS2	c13053_g1	71.821	19.883
	FPPS3	c24092_g1	0	0
geranylgeranyl diphosphate synthase (GGPP synthase)	GGPS1	c23572_g1	9.29	19.108
	GGPS2	c8327_g1	18.642	59.947
