# Supplementary material for: De novo transcriptome analysis of Tibetan medicinal plant Dysphania schraderiana
Source: Genet Mol Biol. 2019 Jun 13;42(2):480–7. doi: 10.1590/1678-4685-GMB-2018-0033 (PMC6726160; doi:10.1590/1678-4685-GMB-2018-0033)
Supplement: Supplementary file 10 [file 1415-4757-GMB-1678-4685-GMB-2018-0033-20190513-suppl7.pdf]

Supplementary Material to "*De novo* transcriptome analysis of Tibetan medicinal plant *Dysphania schraderiana*"

Table S7 - Unigenes annotated to mono-TPS and sesqui-TPS in *D. schraderiana*.

Terpene synthase	KEGG Entry	Annotation	Unigene ID	FPKM of Flower	FPKM of Leaf
Monoterpene	K15086	(3S)-linalool synthase	c12969_g1	7.287	4.564
	K15086	(3S)-linalool synthase	c13138_g1	22.386	5.805
	K15086	(3S)-linalool synthase	c25824_g1	0.897	0
	K15086	(3S)-linalool synthase	c36373_g1	9.097	2.133
	K15086	(3S)-linalool synthase	c37018_g1	2.839	2.068
	K15095	(+)-neomenthol dehydrogenase	c12686_g1*	6.049	44.149
	K15095	(+)-neomenthol dehydrogenase	c12686_g2	12.562	19.172
	K15095	(+)-neomenthol dehydrogenase	c28456_g1	16.097	2.418
	K15095	(+)-neomenthol dehydrogenase	c31738_g1	1.276	3.103
	K15095	(+)-neomenthol dehydrogenase	c23328_g1	0.58	1.926
Sesquiterpene	K14181	valencene/7-epi-alpha-selinene synthase	c4002_g1	6.559	1.487
	K14181	valencene/7-epi-alpha-selinene synthase	c7257_g1	14.248	1.254
	K14181	valencene/7-epi-alpha-selinene synthase	c32093_g1	1.632	1.396
	K15803	(-)-germacrene D synthase	c19835_g1	4.425	0
	K15803	(-)-germacrene D synthase	c31927_g1	2.854	4.279
	K15803	(-)-germacrene D synthase	c7041_g1	7.178	1.836
	K15803	(-)-germacrene D synthase	c7041_g2*	51.826	2.78
	K15891	NAD^+-dependent farnesol dehydrogenase	c800_g1	6.374	3.284

Asterisks (*) represent significantly differential expression between flowers and leaves.
